# Supplementary material for: The effect of seasonal temperatures on the physiology of the overwintered honey bee
Source: PLoS One. 2024 Dec 9;19(12):e0315062. doi: 10.1371/journal.pone.0315062 (PMC11627422; doi:10.1371/journal.pone.0315062)
Supplement: S1 Table — (PDF) [file pone.0315062.s001.pdf]

S1 Table. Survival of honey bee colonies at the end of the overwintering.

Group	control			25			35		
Replication	1	2	3	1	2	3	1	2	3
February 5th	+	+	+	+	+	+	+	+	+
February 27	+	+	+	+	+	+	+	+	+

+ Survival
