# Supplementary material for: The effect of seasonal temperatures on the physiology of the overwintered honey bee
Source: PLoS One. 2024 Dec 9;19(12):e0315062. doi: 10.1371/journal.pone.0315062 (PMC11627422; doi:10.1371/journal.pone.0315062)
Supplement: S2 Table — (PDF) [file pone.0315062.s002.pdf]

S2 Table. PCR primer sequences.

	Locus		Primers, 5`-3`	Amplicon length (bp)	GC (%)	TM (^{o}C)	NCBI Reference Sequence	Efficiency (%)
1	*vg*	F	GCA GAA TAC ATG GAC GGT GT	146	50	58.8	NM_001011578.1	92.36
2		R	GAA CAG TCT TCG GAA GCT TG		50			
3	*ilp1*	F	CGA TAG TCC TGG TCG GTT TG	237	55	60.2	XM_026442144.1	86.96
4		R	CAA GCT GAG CAT AGC TGC AC		55			
5	*ilp2*	F	TTC CAG AAA TGG AGA TGG ATG	166	43	60.1	XM_026444890.1	89.44
		R	TAG GAG CGC AAC TCC TCT GT					
6					55			
7	*TOR1*	F	AAC AAC TGT TGC TGA CGG TG	153	50	58.7	XM_006566642.3	90.54
8		R	GTT GCA GTC CAG GCT TTT TG		50			
9	*JHAMT*	F	TTG GAC ATA GGT TGC GGA CC	302	55	60.0	NM_001327967.1	89.20
10		R	AAT CCT TTT CCT CCT GGC CG		55			
11	*HSP 70*	F	ATC AAC CTG GCG TCT TGA TTC	104	48	60.7	NM_001160072.2	90.93
12		R	AGG TGC AGG TGG AAT GCC AG		60			
13	*RpL32*	F	AAG TTC ATT CGT CAC CAG AG	205	45	57.9	XM_016914656.2	89.53
14		R	CTT CCA GTT CCT TGA CAT TAT G		41			
