# Supplementary figures and images for: The effect of seasonal temperatures on the physiology of the overwintered honey bee
Source: PLoS One. 2024 Dec 9;19(12):e0315062. doi: 10.1371/journal.pone.0315062 (PMC11627422; doi:10.1371/journal.pone.0315062)

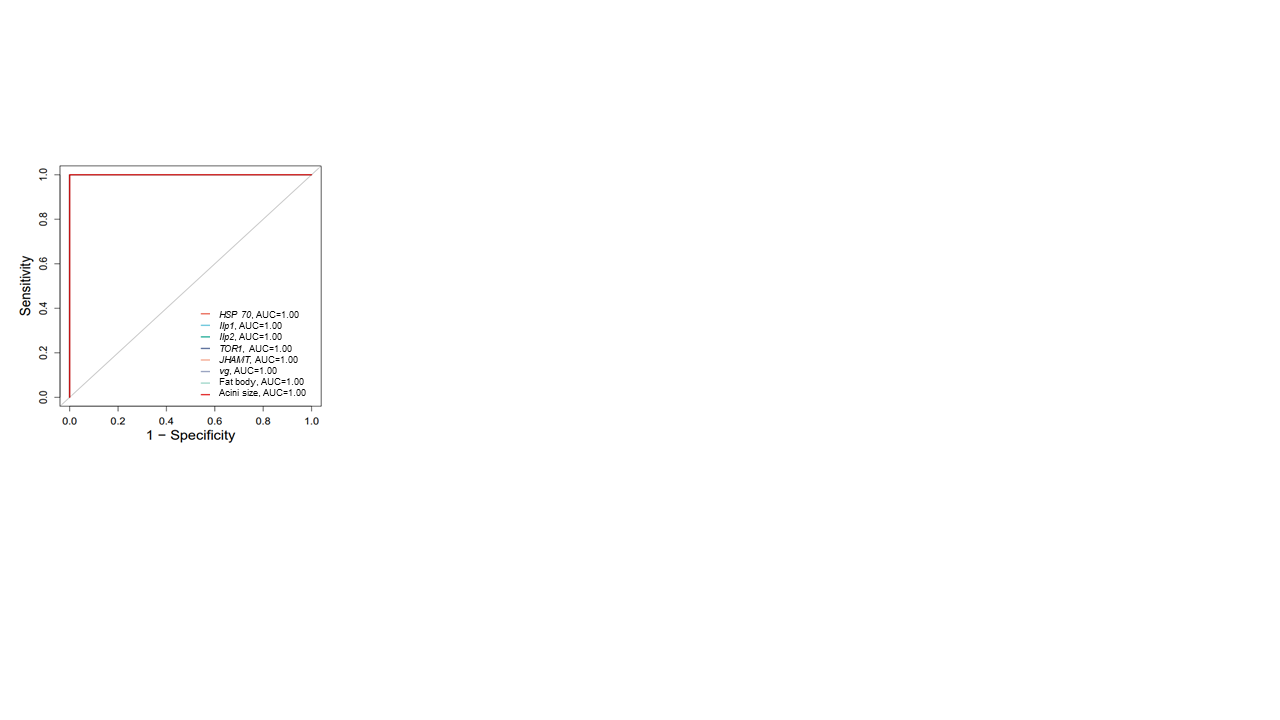

Supplement: S1 Fig — (TIF) [file pone.0315062.s003.tif]

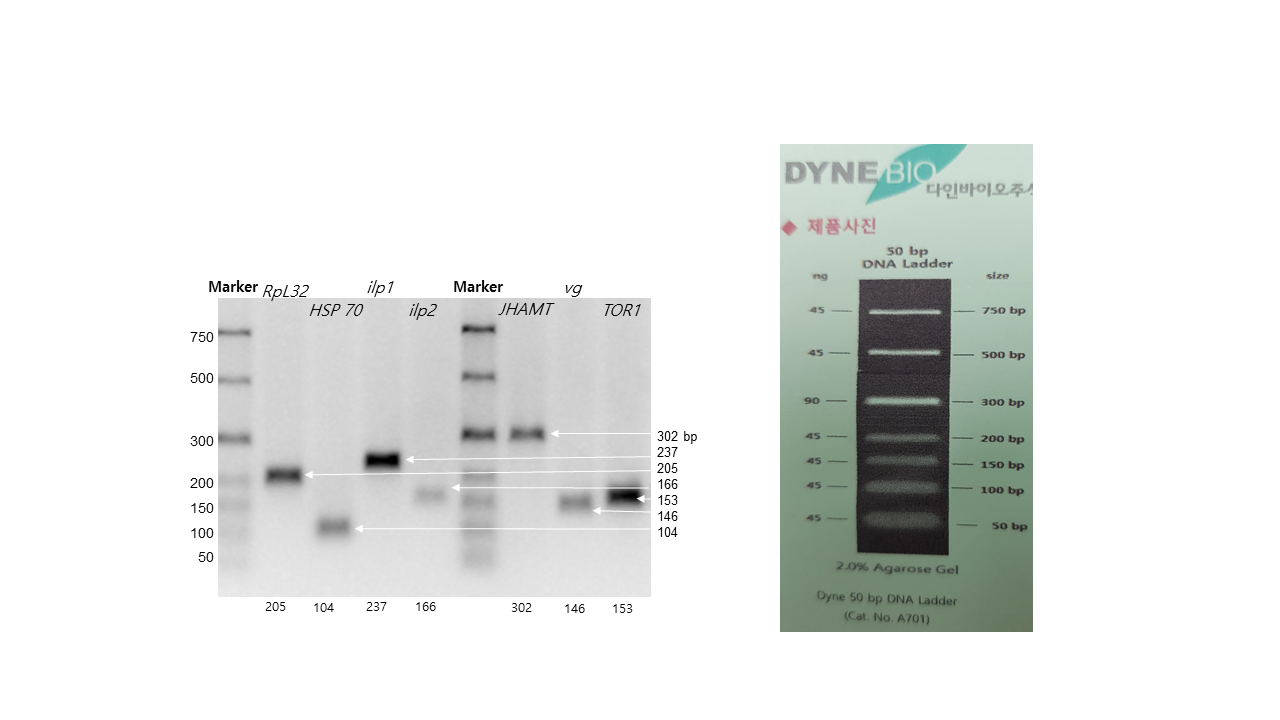

Supplement: S2 Fig — (TIF) [file pone.0315062.s004.tif]
