# Supplementary material for: The effect of seasonal temperatures on the physiology of the overwintered honey bee
Source: PLoS One. 2024 Dec 9;19(12):e0315062. doi: 10.1371/journal.pone.0315062 (PMC11627422; doi:10.1371/journal.pone.0315062)
Supplement: Attachment — Submitted filename: PLOS-ONED-24-13353_reviewer.pdf [file pone.0315062.s012.pdf]

PLOS ONE

The effect of seasonal temperatures on the aging of the overwintered honey bee

--Manuscript Draft--

**Manuscript Number:**	PONE-D-24-13353
**Article Type:**	Research Article
**Full Title:**	The effect of seasonal temperatures on the aging of the overwintered honey bee
**Short Title:**	Aging of honey bees at the end of winter
**Corresponding Author:**	Hyung Wook Kwon<br>Incheon National University<br>Incheon, KOREA, REPUBLIC OF
**Keywords:**	honey bee; aging; vitellogenin; acinus size; molecular markers; climate warming
**Abstract:**	Honey bee workers' survival hinges on an annual cycle, marked by extended longevity in winter workers (long-lived), approximately 10 times longer than in their summer workers (short-lived). While previous studies indicate temperature's impact on longevity, the underlying molecular mechanism remains elusive. In our study, we maintained constant temperatures in rooms at 25°C (Group 25) and 35°C (Group 35) around hives from June to October, simulating climate warming, with a control group experiencing natural conditions. Colony performance was assessed in August and September, and in February, workers were examined for physiological traits (Acinus size and Fat body weight) and expression of known task-related molecular markers (vg and JHAMT), along with expected markers (ilp1, ipl2, TOR1, and HSP70). Our findings suggest that temperature decreases around winter worker broods from Group 25 in autumn lead to their slower physiological aging in winter compared to Group 35 workers, supported by physiology and task-related markers. Furthermore, we observed a relation between aging and HSP70 and TOR1 genes, which may serve as additional markers, necessitating further research. This approach could enhance molecular aging identification in insect models, advancing climate warming-related research.
**Order of Authors:**	Hyung Wook Kwon
	Olga Frunze
	Yumi Yun
	Hyun Jee Kim
	Ravil R. Garafutdinov
	Young-Eun Na
**Additional Information:**	
**Question**	**Response**
**Financial Disclosure**<br><br>Enter a financial disclosure statement that describes the sources of funding for the work included in this submission. Review the submission guidelines for detailed requirements. View published research articles from *PLOS ONE* for specific examples.<br><br>This statement is required for submission and **will appear in the published article** if the submission is accepted. Please make sure it is accurate.	Yes<br>Hyung Wook Kwon received.<br>This work was carried out with the support of the Cooperative Research Program for Agriculture Science & Technology Development (Project No. RS-2023-00232749) and the Priority Research Centers Program through the National Research Foundation of Korea (NRF) funded by the Ministry of Education (2020R1A6A1A03041954).

Powered by Editorial Manager® and ProduXion Manager® from Aries Systems Corporation

**Unfunded studies**<br>Enter: *The author(s) received no specific funding for this work.*<br><br>**Funded studies**<br>Enter a statement with the following details:<br>• Initials of the authors who received each award<br>• Grant numbers awarded to each author<br>• The full name of each funder<br>• URL of each funder website<br>• Did the sponsors or funders play any role in the study design, data collection and analysis, decision to publish, or preparation of the manuscript?<br>• **NO** - Include this sentence at the end of your statement: *The funders had no role in study design, data collection and analysis, decision to publish, or preparation of the manuscript.*<br>• **YES** - Specify the role(s) played.<br><br>* typeset	
**Competing Interests**<br><br>Use the instructions below to enter a competing interest statement for this submission. On behalf of all authors, disclose any competing interests that could be perceived to bias this work—acknowledging all financial support and any other relevant financial or non-financial competing interests.<br><br>This statement is required for submission and **will appear in the published article** if the submission is accepted. Please make sure it is accurate and that any funding sources listed in your Funding Information later in the submission form are also declared in your Financial Disclosure statement.<br><br>View published research articles from *PLOS ONE* for specific examples.	The authors have declared that no competing interests exist.

**NO authors have competing interests**<br><br>Enter: *The authors have declared that no competing interests exist.*<br><br>**Authors with competing interests**<br><br>Enter competing interest details beginning with this statement:<br><br>*I have read the journal's policy and the authors of this manuscript have the following competing interests: [insert competing interests here]*<br><br>* typeset	
**Ethics Statement**<br><br>Enter an ethics statement for this submission. This statement is required if the study involved:<br><br>• Human participants<br>• Human specimens or tissue<br>• Vertebrate animals or cephalopods<br>• Vertebrate embryos or tissues<br>• Field research<br><br>Write "N/A" if the submission does not require an ethics statement.<br><br>General guidance is provided below. Consult the submission guidelines for detailed instructions. **Make sure that all information entered here is included in the Methods section of the manuscript.**	N/A

**Format for specific study types**<br><br>**Human Subject Research (involving human participants and/or tissue)**<br>• Give the name of the institutional review board or ethics committee that approved the study<br>• Include the approval number and/or a statement indicating approval of this research<br>• Indicate the form of consent obtained (written/oral) or the reason that consent was not obtained (e.g. the data were analyzed anonymously)<br><br>**Animal Research (involving vertebrate animals, embryos or tissues)**<br>• Provide the name of the Institutional Animal Care and Use Committee (IACUC) or other relevant ethics board that reviewed the study protocol, and indicate whether they approved this research or granted a formal waiver of ethical approval<br>• Include an approval number if one was obtained<br>• If the study involved *non-human primates*, add *additional details* about animal welfare and steps taken to ameliorate suffering<br>• If anesthesia, euthanasia, or any kind of animal sacrifice is part of the study, include briefly which substances and/or methods were applied<br><br>**Field Research**<br><br>Include the following details if this study involves the collection of plant, animal, or other materials from a natural setting:<br>• Field permit number<br>• Name of the institution or relevant body that granted permission	
**Data Availability**<br><br>Authors are required to make all data underlying the findings described fully available, without restriction, and from the time of publication. PLOS allows rare exceptions to address legal and ethical concerns. See the PLOS Data Policy and FAQ for detailed information.	Yes - all data are fully available without restriction

A Data Availability Statement describing where the data can be found is required at submission. Your answers to this question constitute the Data Availability Statement and **will be published in the article**, if accepted.<br><br>**Important:** Stating 'data available on request from the author' is not sufficient. If your data are only available upon request, select 'No' for the first question and explain your exceptional situation in the text box.<br><br>Do the authors confirm that all data underlying the findings described in their manuscript are fully available without restriction?	
**Describe where the data may be found in full sentences. If you are copying our sample text, replace any instances of XXX with the appropriate details.**<br><br>• If the data are **held or will be held in a public repository**, include URLs, accession numbers or DOIs. If this information will only be available after acceptance, indicate this by ticking the box below. For example: *All XXX files are available from the XXX database (accession number(s) XXX, XXX.).*<br>• If the data are all contained **within the manuscript and/or Supporting Information files**, enter the following: *All relevant data are within the manuscript and its Supporting Information files.*<br>• If neither of these applies but you are able to provide **details of access elsewhere**, with or without limitations, please do so. For example:<br><br>*Data cannot be shared publicly because of [XXX]. Data are available from the XXX Institutional Data Access / Ethics Committee (contact via XXX) for researchers who meet the criteria for access to confidential data.*<br><br>*The data underlying the results presented in the study are available from (include the name of the third party*	All relevant data are within the manuscript and its Supporting Information files.

and contact information or URL).<br>• This text is appropriate if the data are owned by a third party and authors do not have permission to share the data.<br><br>* typeset	
Additional data availability information:	

Manuscript
Click here to access/download;Manuscript;Manuscript.docx

Research article

The effect of seasonal temperatures on the aging of the overwintered honey bee

Olga Frunze[&1], Yumi Yun[&1], Hyun Jee Kim[1], Ravil R. Garafutdinov[2], Young-Eun Na[3]*, and Hyung-Wook Kwon[1]*

[1]Department of Life Sciences & Convergence Research Center for Insect Vectors (CRCIV), Incheon National University, 119 Academy-ro, Yeonsu-gu, Incheon, Republic of Korea 22012; frunzeon@gmail (O.F.); yym8804@naver.com (Y.Y.); beamed79@hanmail.net (H.J.K.)

[2]Institute of Biochemistry and Genetics, Ufa Federal Research Center, Russian Academy of Sciences, 450054, prosp. Oktyabrya, 71, Ufa, Bashkortostan, Russian Federation garafutdinovr@gmail.com (R.R.G.)

[3]Agro-materials Industry Division, Rural Development Administration (RDA), Wanju 55365, Republic of Korea

[&]These authors contributed equally to this work

*Correspondence: hwkwon@inu.ac.kr (H.-W.K.) youngman99@korea.kr (Y.-E.N.)

Short title: **Aging of honey bees at the end of winter**

Abstract

Honey bee workers' survival hinges on an annual cycle, marked by extended longevity in winter workers (long-lived), approximately 10 times longer than in their summer workers (short-lived). While previous studies indicate temperature's impact on longevity, the underlying molecular mechanism remains elusive. In our study, we maintained constant temperatures in rooms at 25°C (Group 25) and 35°C (Group 35) around hives from June to October, simulating climate warming, with a control group experiencing natural conditions. Colony performance was assessed in August and September, and in February, workers were examined for physiological traits (Acinus size and Fat body weight) and expression of known task-related molecular markers (*vg* and *JHAMT*), along with expected markers (*ilp1*, *ipl2*, *TOR1*, and *HSP70*). Our findings suggest that temperature decreases around winter worker broods from Group 25 in autumn lead to their slower physiological aging in winter compared to Group 35 workers, supported by physiology and task-related markers. Furthermore, we observed a relation between aging and *HSP70* and *TOR1* genes, which may serve as additional markers, necessitating further research. This approach could enhance molecular aging identification in insect models, advancing climate warming-related research.

Introduction

Honey bees play a crucial role in human life as producers of health and medicinal products, pollinators on agricultural farms, and contributors to ecosystems worldwide. However, the loss of honey bees has been reported over the last two decades, particularly at the end of the overwintering period (Döke et al., 2015; Hristov et al., 2020; Insolia et al., 2022; Gray et al., 2023). Various factors contribute to honey bee winter mortality, and currently, we do not have effective methods to prevent it.

To advance in this direction, it is necessary to elucidate the processes involved in the transition from short-lived workers to long-lived ones. This transition has been associated with a decrease in pollen availability (Crailsheim, 1986; Dellinger, 2023), cessation of brood rearing (Fruri et al., 1982; Münch et al., 2013), and declining ambient temperatures (Simpson, 1961; Medrzycki et al., 2012). Although seems honey bees maintain a consistent brood microclimate (Abou-Shaara et al., 2017; Bencsik et al., 2023), climate warming has been shown to significantly increase brood temperature in summer (Coulibaly et al., 2022), and a similar effect can be expected in fall, although this has not been thoroughly studied yet. With the onset of fall environmental triggers, the transition of honey bees is initiated, leading to the development of winter workers with prolonged longevity, which can reach up to 8 months (Döke et al., 2015). It exhibits the

classic insect diapause phenotype characterized by altered hormonal profiles and reduced metabolic activity (Fluri et al., 1982; Winston, 1987; Hartfelder and Engels, 1998; Rodriges and Flatt, 2016; Bresnahan et al., 2022). In honey bees, this phenomenon is well-described, manifesting as increased Acinus size (hypopharyngeal gland, HG), Fat body weight, and vitellogenin (vg) levels, alongside decreased juvenile hormone (JH) levels (Fluri et al., 1977; Döke et al., 2015; Knoll et al., 2020). However, unlike other insects during diapause (Denlinger, 2023), honey bees require a pure carbohydrate diet, as their metabolism facilitates the heating of the winter cluster by vibrating the wing muscles (Brejcha et al., 2023). The duration of diapause is crucial for successful honey bee overwintering (Denlinger, 2023). Furthermore, at the end of winter, diapause nurses gradually transition to nursing brood care and foraging physiology (Döke et al., 2015). The last transition is characterized by several physiological and molecular changes, including a decrease in Acinus size (Corby-Harris and Snyder, 2018) and Fat body mass (Corby-Harris et al., 2019), as well as reductions in vg levels and increases in juvenile hormone (JH) levels (Fluri et al., 1977; Amdam and Omholt, 2002). These changes contribute to seasonal effects and behavioral activities (Ali et al., 2019), inevitably leading to reduced longevity and increased aging (Alaux et al., 2018; Diebel and Rockwood, 2021).

Alongside the well-known task-related molecular marker genes *vg* and

hormone JH (Elekonich et al., 2001; Amdam et al., 2005; Guidugli et al., 2005), other potential markers such as *JHAMT, ilp1*, *ilp2*, *TOR1*, and *HSP70* genes were examined in this study. The enzyme involved in JH biosynthesis, *Juvenile Hormone Acid Methyltransferase (JHAMT)*, exhibits changes in expression direction similar to JH titer and can serve as an alternative marker to JH (Goodman and Granger, 2005; Li et al., 2013; Egi and Sakamoto, 2022). A positive correlation with JH was observed in the *insulin-like peptide*, *ilp1*, whose expression increases in the brains of foragers and decreases during continued foraging (Corona et al., 2007; Ament et al., 2008). The *insulin-like peptide, ilp2* gene is expected to have task-related expression as it is highly expressed in nurse tissue and plays a role in increasing cell size and number in individual organs (Zhang et al., 2022). Additionally, the *TOR1* gene, a nutrient-sensing kinase, along with the *ilp1* and *ilp2* genes, participates in the mobilization of nutritional resources (autophagy) from tissues such as the fat body and glands in *Drosophila* (Barth et al., 2010). Moreover, the *TOR* pathway is implicated in disease and nutrition in insects (Patel et al., 2007; Laplante and Sabatini, 2012; Johnson et al., 2013), and mammals (Zoncu et al., 2011), and it also regulates longevity extension in social insects (Rodrigues and Flatt, 2016). Lastly, the stress marker *heat shock protein 70 (HSP70)* was chosen as it is detected at low levels in nurses but shows increased expression in foragers (Sario˘glu-Bozkurt et al., 2022).

While all these markers are expected to be important for identifying aging in honey bees, testing them together requires further investigation.

This research aimed to simulate the influence of climate warming on colony performance in early fall and compare the aging of overwintered *Apis mellifera ligustica* honey bees. The experiment considered ambient temperatures of 25°C (Group 25) and 35°C (Group 35), which simulated the average and high summer temperatures in Incheon (Republic of Korea), respectively, while natural conditions were maintained in the Group control. The temperature experiments lasted throughout the previous summer, September, and October, with the colonies overwintering under the same conditions. Colony performance, including brood microclimate, was investigated in August and September because that month honey bee transformation from summer to winter physiology started in the Republic of Korea. The aging of honey bees at the end of diapause (broodless period) in February was determined by eight markers, including two physiological markers (Acinus size and Fat body weight) and six molecular gene markers related to the task (*vg* and *JHAMT*), nutrition (*ilp1*, *ilp2*, and *TOR1*), and stress response (*HSP70*). Our findings suggest that temperature decreases around winter worker broods from Group 25 in the fall lead to their slower physiological aging in winter compared to Group 35 workers, supported by physiology and task-related markers. Furthermore, we observed a relation between aging and

HSP70 and *TOR1* genes, which may serve as additional markers, necessitating further research. This approach could enhance the molecular method for identifying aging in insect models, thereby advancing research related to climate warming.

Results

Dynamics of ambient temperature in summer and fall months

The daily temperature in the first experimental room was set at 25°C for five months, and there were no significant differences compared to the natural daily average temperature in the summer months or September ($p > 0.05$). However, it was significantly greater than the average daily temperature in October 2022 ($p < 0.05$). The daily temperature in the second room was set at 35°C for five months, which was significantly greater than the average daily temperature during the summer months, September, and October ($p < 0.05$) (**Fig 6E**).

Brood microclimate and colony development in August and September

In this study, we evaluated the performance of the colony using temperature, humidity, CO_2, number of larvae, capped brood area, and honey bees' measurements from August 08 to September 18, 2022, when short-lived honey bees commenced rearing larvae that would develop into long-lived honey bees for the overwintering period. We hypothesized that the regulation of the nest

climate inside colonies influences the physiology of winter honey bees and the success of overwintering.

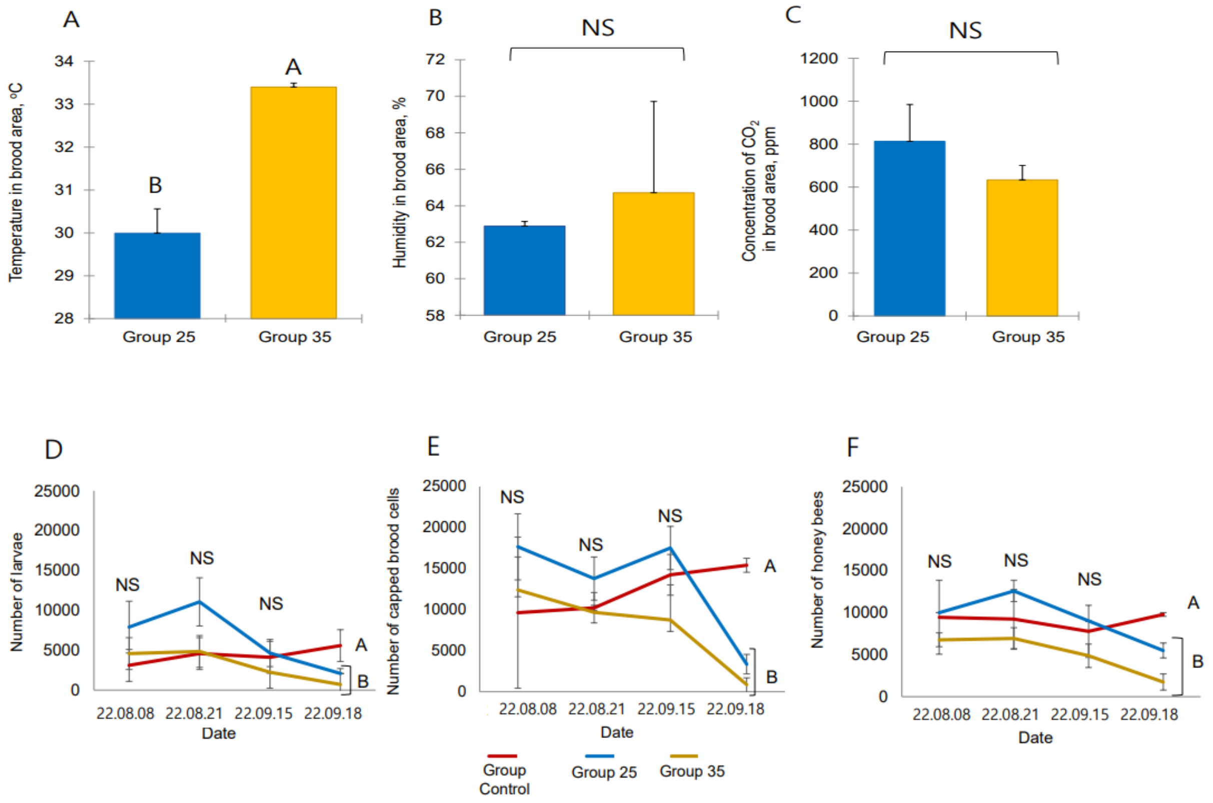

Fig 1. Colony performance in honey bees treated under different ambient temperatures (08 August – 18 September 2022). (A) Temperature in brood area (°C). **(B)** Humidity in brood area (%). **(C)** Concentration of CO_2 in brood area (ppm). **(D)** Number of larvae. **(E)** Number of capped brood cells. **(F)** Number of honey bees. Statistical significance was determined using one-way ANOVA and Duncan post hoc tests ($p < 0.05$, indicated by different letters above the columns); bars in the graphs represent the mean ± SD.

SD – Standard Deviation, NS – No Significance. Honey bee colonies in Group 25 were maintained in room conditions at 25°C; colonies in Group 35 were kept in room conditions at 35°C, while Group Control colonies were maintained under natural conditions.

The mean and standard deviation of the nest temperature for honey bee

colonies kept at 25 and 35°C were 29.995 ± 0.982 and 33.398 ± 0.154 °C, respectively. Similarly, the nest humidities for these groups were 62.889 ± 0.435 0.435 and 64.717 ± 8.650 %, and the nest CO_2 concentrations were 813.553 ± 296.841 and 633.298 ± 116.959 ppm, respectively. Importantly, the nest temperature inside the honey bee colonies kept at 35°C was significantly greater than that inside the honey bee colonies kept at 25°C ($p < 0.05$) (**Fig 1A**). However, nest humidity, CO_2 concentration, number of larvae, capped brood cells, and honey bees were not significantly differing ($p > 0.05$) (**Fig 1B, C, D, E, and F**). The colonies in the Group control, however, exhibited significantly ($p < 0.05$) larger capped brood area, more larvae, and a higher number of honey bees on September 18 compared to colonies in Groups 25 and 35. This observation suggests the importance of day-night and seasonal temperature fluctuations in the fall for colony development, especially as the colony prepares for overwintering.

Molecular marker recognition in overwintered western honey bees

The molecular markers recognition related to the biological age of long-lived honey bees, which were approximately 4.5 months old, was verified through a dataset comprising two physiological (Acinus size and Fat body weight) and six molecular markers (*vg*, *TOR1*, *JHAMT*, *ilp1*, *ilp2*, and *HSP70*) using Data Mining methods, including Discriminant Analysis (DA), Agglomerative Hierarchical Clustering (AHC), and Elastic Net Regression.

DA was performed to plot the honey bee Groups control, 25 and 35 and to identify the optimal combination of features that effectively distinguishes them. The Wilcoxon Lambda test within the DA yielded a statistically significant result ($p < 0.05$), indicating that the model successfully differentiated between groups (**Fig 2A**). The observed Wilks' lambda value of 0.000 signifies a robust separation of the Groups control, 25, and 35, demonstrating a high level of discriminative power. A comparison revealed significant differences between Group 25 and Group 35 ($F(2,8) = 19.37$, $p < 0.05$; **Fig 2A**).

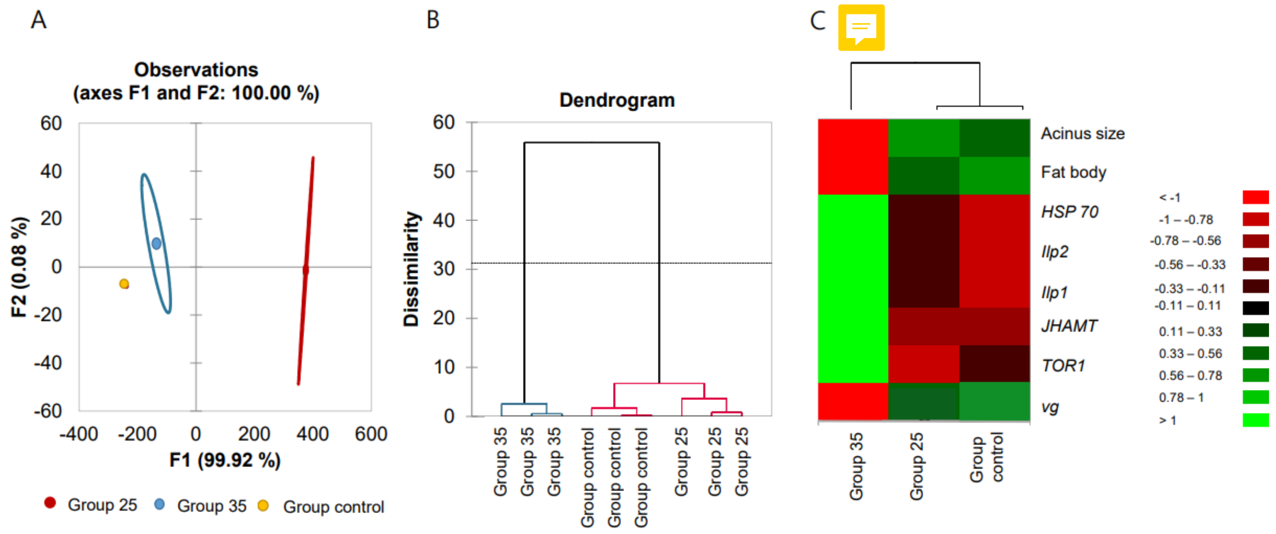

Fig 2. The discrimination of long-lived honey bees at the end of winter.
(A) Discriminant Analysis. **(B)** Agglomerative Hierarchical Clustering (AHC). **(C)** Heatmap with cluster analysis based on ddCt values, Acinus size (mm^2), and Fat body weight (mg). Honey bee colonies in Group 25 were maintained in room conditions at 25°C; colonies in Group 35 were kept in room conditions at 35°C, while Group Control colonies were maintained under natural conditions.

The Fisher distances (FDs) in the DA between the Control, Group 25, and Group 35 honey bees were significantly different (FD = 27511.10; $p < 0.05$ and FD = 19131.16; $p < 0.05$, respectively). Moreover, the FD density was also significantly different between the honey bees from Group control and Group 25 (FD = 783.659; $p < 0.05$). The honey bees were separated along the primary discriminant axis F1 in 99.98% of the samples, indicating that the gene expression of *vg*, *HSP70*, and *JHAMT* significantly contributed to this separation.

Subsequently, AHC was employed to identify natural groupings without labeled information using a dissimilarity measure. The evaluation metric for clustering algorithms, the Silhouette index between clusters 1 and 2, was 0.657, indicating successful clustering, albeit with moderate density **(Fig 2B, C).**

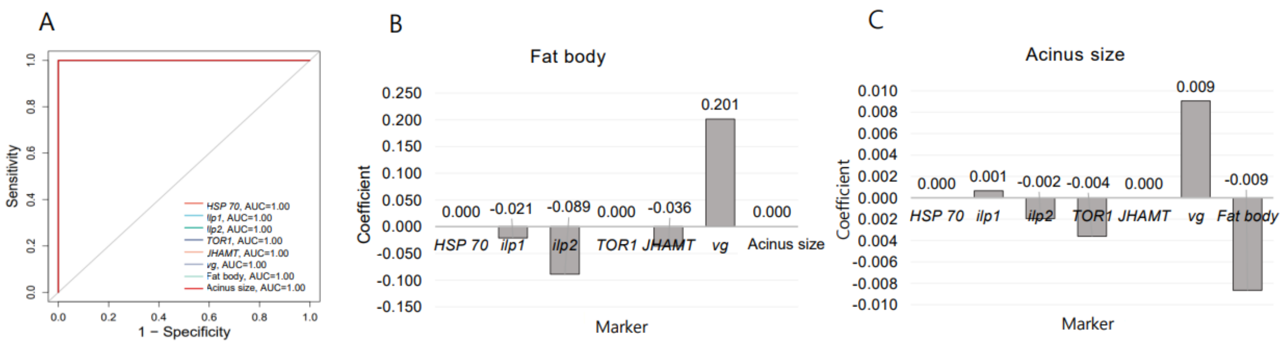

Fig 3. Elastic Net Regression model for the dependent variables Fat body and Acinus size. **(A)** The quality verification plot (specificity *vs* sensitivity) demonstrated a perfectly fitted model linking physiology to molecular markers (gene expression profile). **(B)** The regression model of predictor importance and ranking for Fat body weight. **(C)** The regression model of predictor importance and ranking for Acinus size.

Finally, Elastic Net Regression was used to rank the markers by importance by

modeling the relationships between seven independent variables and one dependent variable **(Fig 3A, B, C).** This analysis was conducted separately for Fat body **(Fig 3B)** and Acinus size **(Fig 3C).** The Fat body model identified significant predictors, with variable *vg* having the highest positive coefficient (0.201) and variable *ilp2* having the highest negative coefficient (-0.089). In the Acinus size model, significant predictors were identified, with the variable *vg* having the highest positive coefficient (0.009) and the variable Fat body having the highest negative coefficient (-0.009).

Comparative analysis of the biological age of overwintered western honey bees through physiology markers

The comparative biological age of the overwintered honey bees was evaluated based on the molecular and physiological markers. The latter, included in this results, consisted of the Acinus size of the hypopharyngeal glands (HGs) and analysis of Fat body weight. The mean and standard deviation of the Fat body weight for the honey bees in the Groups control, 25, and 35 were 4.33 ± 1.247, 4.000 ± 1.633, and 2.000 ± 0.816 mg/bee, respectively, with no significant differences (P = 0.222, $p < 0.05$), even though the Fat body weight of the honey bees in Group 35 was slightly lower **(Fig 4A).**

Similarly, the mean and standard deviation of the size of the honey bee Acinus from the Groups control, 25, and 35 were 0.026 ± 0.001, 0.027 ± 0.003, and 0.018

± 0.001 mm², respectively. Notably, the Acinus size of the honey bee in Group 35 was significantly lower (P = 0.006, $p < 0.05$) than that of the Group control and 25 **(Fig 4B).** Importantly, both the Fat body weight and the Acinus size of the overwintered honey bees in Group 35 were lower than those of the overwintered honey bees in Groups control and 25 **(Fig 4)**, suggesting accelerated physiology aging of honey bees from Group 35.

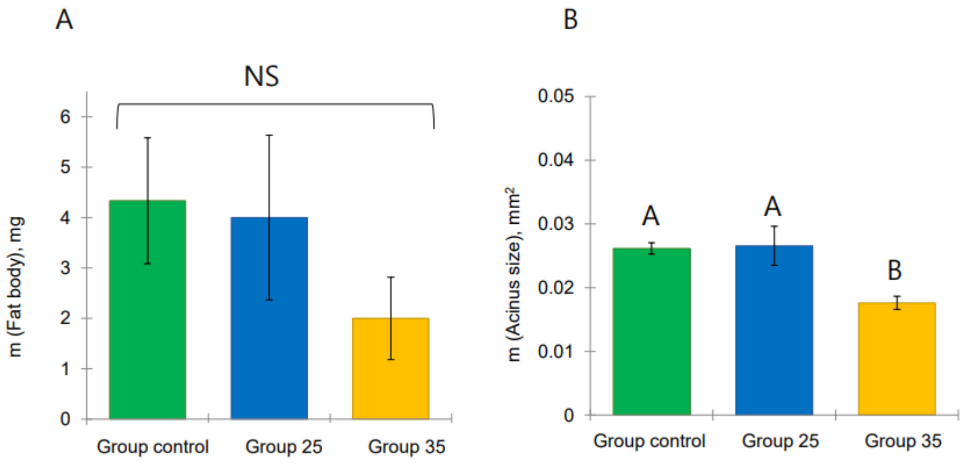

Fig 4. Physiological markers of long-lived honey bees at the end of winter (February) (A) Average Acinus size, n > 200 (mean ± SD). **(B)** Average Fat body weight, n = 10 (mean ± SD). Statistical significance was determined using one-way ANOVA and Duncan post hoc tests ($p < 0.05$, indicated by different letters above the columns). SD - Standard Deviation. NS - Not Significant. Honey bee colonies in Group 25 were maintained in room conditions at 25°C; colonies in Group 35 were kept in room conditions at 35°C, while Group Control colonies were maintained under natural conditions.

Comparative analysis of the biological age of overwintered western honey bees through molecular markers

These are the results of the comparative biological age of the overwintered honey bees based on molecular markers. They include two previously defined task-related markers, *vg* and *JHAMT*, as well as four genes expected to respond to aging: *TOR1*, *ilp1*, *ilp2*, and *HSP70* **(Fig. 5).**

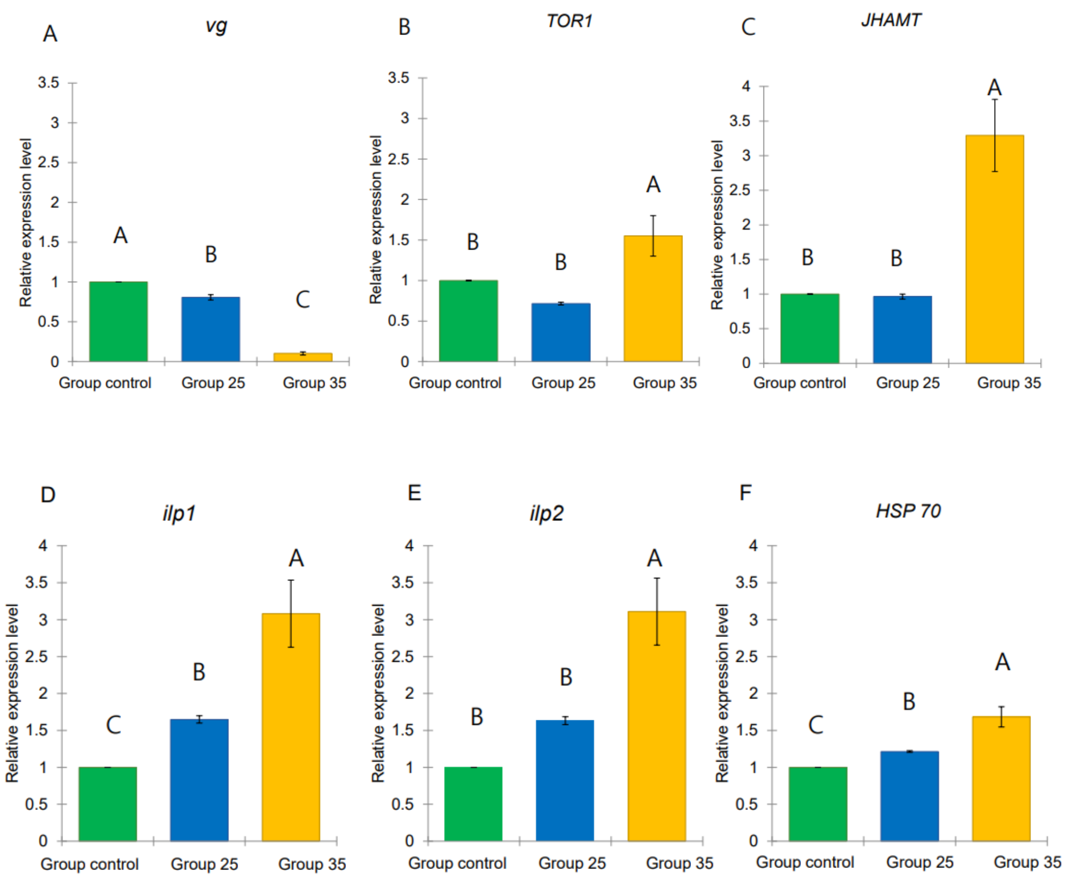

Fig 5. Gene expression analysis The gene expression (ddCt) of *vg** **(A),** *TOR1*** **(B),** *JHAMT*** **(C),** *ilp1*** **(D),** *ilp2*** **(E),** and *HSP70*** **(F)** in long-lived honey bees at the end of overwintering (February) was evaluated. Statistical significance was determined using one-way ANOVA and Duncan post hoc tests ($p < 0.05$, indicated by different letters above the columns). The bars in the graphs represent the mean ± SD. SD – Standard Deviation. *n=9. **n=15. Honey bee colonies in Group 25 were maintained in room conditions at 25°C; colonies in Group 35 were kept in room conditions at 35°C, while Group Control colonies were maintained under natural conditions.

Expression of *vg* gene was significantly upregulated (ANOVA, Duncan post-hoc test, $p < 0.05$) in honey bees from Group control and 25 compared to honey bees from Group 35. In contrast, the expression of the *TOR1*, *JHAMT, ilp1*, *ilp2*, and *HSP70* genes was significantly downregulated (ANOVA, Duncan post-hoc test, $p < 0.05$) in the same honey bees, suggesting aging of honey bees from Group 35 compared to honey bees from Groups control and 25 **(Fig. 5B, C, D, E, and F)**. However, there were no significant differences in the expression of *TOR1*, *JHAMT*, and *ilp2* (ANOVA, Duncan post-hoc test, $p > 0.05$) between honey bees from Group control and Group 25. The expression levels of *ilp1* and *HSP70* were significantly lower (ANOVA, Duncan post-hoc test, $p < 0.05$) in the honey bees Group control than in Group 25.

Discussion

Key triggers influencing the transition of insects from summer to winter physiology include photoperiod, ambient temperature, brood microclimate, availability of pollen, and some yet-to-be-identified factors (Knoll et al., 2020; Rodrigues and Flatt, 2016; Quinlan et al., 2023), which obviously have a particular influence on aging and subsequent winter loss in honey bees. However, due to the complex and unclear interactions among all these triggers, we designed an experiment in which only one trigger, ambient temperature (25, 35°C, and

control), was manipulated during the previous summer, September, and October. Colony development and nest climate regulation were investigated in August and September, during which long-lived honey bees were reared and marked to study the biological age of honey bees alongside their clear chronological age in February. Aging was assessed using two physiological markers (Acinus size and Fat body weight) and six molecular markers (*vg*, *TOR*, *JHAMT*, *ilp1*, *ilp2*, and *HSP70*) at the beginning of February, during the expected diapause continuation.

Interestingly, the typical nest temperature range for honey bee brood rearing fluctuates between 33 and 36°C (Kovac et al., 2009). However, honey bee Group 25 maintained a subnormal brood temperature, lower than 3.403°C compared to Group 35 at the end of August **(Fig 1)**. Additionally, Szentgyörgyi et al. (2018) reported that short-lived honey bees live longer after developing at a low temperature (32°C), which is crucial for overwintering. Due to the successful overwintering of honey bees Groups 25 and 35, we suggest that honey bees initially had to decrease the nest temperature to rear brood with a slower metabolism, larger hypopharyngeal gland (HG) size, and higher Fat body weight for overwintering (Knoll et al., 2020). However, in high ambient temperatures, this adjustment was not feasible. Moreover, we confirmed that high ambient temperatures had an indirect effect on honey bees' transition through changes in physiology and molecular markers value at the end of

winter.

The physiological markers of long-lived (winter) honey bees include hypertrophied HG, which plays a key function in royal jelly production for larval rearing in spring, and an enlarged Fat body, resulting from the accumulation of nutrients (Smedal et al., 2009). A comparative study revealed that Acinus sizes in honey bee Groups 25 and the control were significantly greater ($p < 0.05$) than those in honey bee Group 35. The same trend was observed in Fat body weights, although the differences were not statistically significant. Given that the sampling was conducted on February 5-6, these physiological differences were indicative of the honey bees' diapause condition. The honey bee Group 25 and control exhibited a higher body nutrient stock than honey bee Group 35, suggesting an aging process.

This process is closely associated with molecular markers (Alaux et al., 2018; Kaur and Singh, 2020; Daniels et al., 2023). In long-lived (winter) honey bees, these markers include elevated vg levels and suppressed JH levels (Smedal et al., 2009) in comparison to short-lived (spring-summer) honey bees (Amdam and Omholt, 2002; Döke et al., 2015; Knoll et al., 2020). These molecular levels remain stable during diapause and undergo a switch before the onset of brood rearing (Amdam and Omholt, 2002). Notably, *vg* gene expression was significantly higher in honey bee Group 25 and the control, whereas *JHAMT* gene

expression was significantly lower compared to honey bees from Group 35, which also suggests an aging process in the latter honey bees.

The relationship between *vg* gene expression and the hypopharyngeal gland (HG) and Fat body was validated using Elastic Net Regression models **(Fig 3B, C).** Furthermore, Discriminant Analysis (DA) identified three genes, *JHAMT*, *vg*, and *HSP70*, with high scores, underscoring their significance in discriminating among honey bee groups. Even though the *HSP70* gene is synthesized in response to stress (Zhang et al., 2015), it is associated with honey bee transitions in summer and aging (Sario˘glu-Bozkurt et al., 2022). The highest *HSP70* gene expression was observed in honey bee Group 35, while the lowest was in the control group, supporting the aging hypothesis of temperature-treated honey bees. The subsequent gene, *TOR1*, wasn't selected by Data Mining methods but can be considered aging-related (Barth et al., 2010; Fahrbach et al., 2012). This gene exhibited high expression in honey bee Group 35, explaining the decrease in Fat body weight compared to the Group control and 25, even though it was not significant. The last two genes, *ilp1*, and *ilp2* require careful consideration as they exhibit complex metabolic interactions with relationships that depend not only on nutrition, age, and season (Nilsen et al., 2011; Chowański et al., 2021).

In summary, we suggested a connection between regular ambient

temperature, simulated climate warming, below-average brood temperature, and the delayed aging of long-lived honey bees in February. Conversely, under conditions of high-temperature stress, honey bees were unable to lower brood temperature, resulting in the expedited aging of long-lived individuals throughout the winter season. The acceleration of biological aging in Group 35 honey bees was evidenced by downregulated *vg* gene expression and upregulated *JHAMT, HSP70*, and *TOR1* gene expression. These molecular changes resulted in low internal nutrient stores in the Fat body and reduced Acinus size, suggesting a potential set of molecular markers for aging. We believe that this research can pave the way for additional studies and, ultimately, the development of a marker kit to identify the reasons and processes of aging at an early stage. This can help protect nature and humans from ecological challenges. Climate change poses a threat to organisms worldwide, as does understanding how organisms respond to extreme heat events, associated with climate change can inform conservation actions.

Materials and methods

Experimental bees

Western honey bees (*Apis mellifera ligustica*) were obtained from nine colonies kept at the Incheon National University apiary in the Republic of Korea **(Fig 6A,**

B, C, and D), managed by a professional beekeeper following standard beekeeping practices (Delaplane et al., 2013).

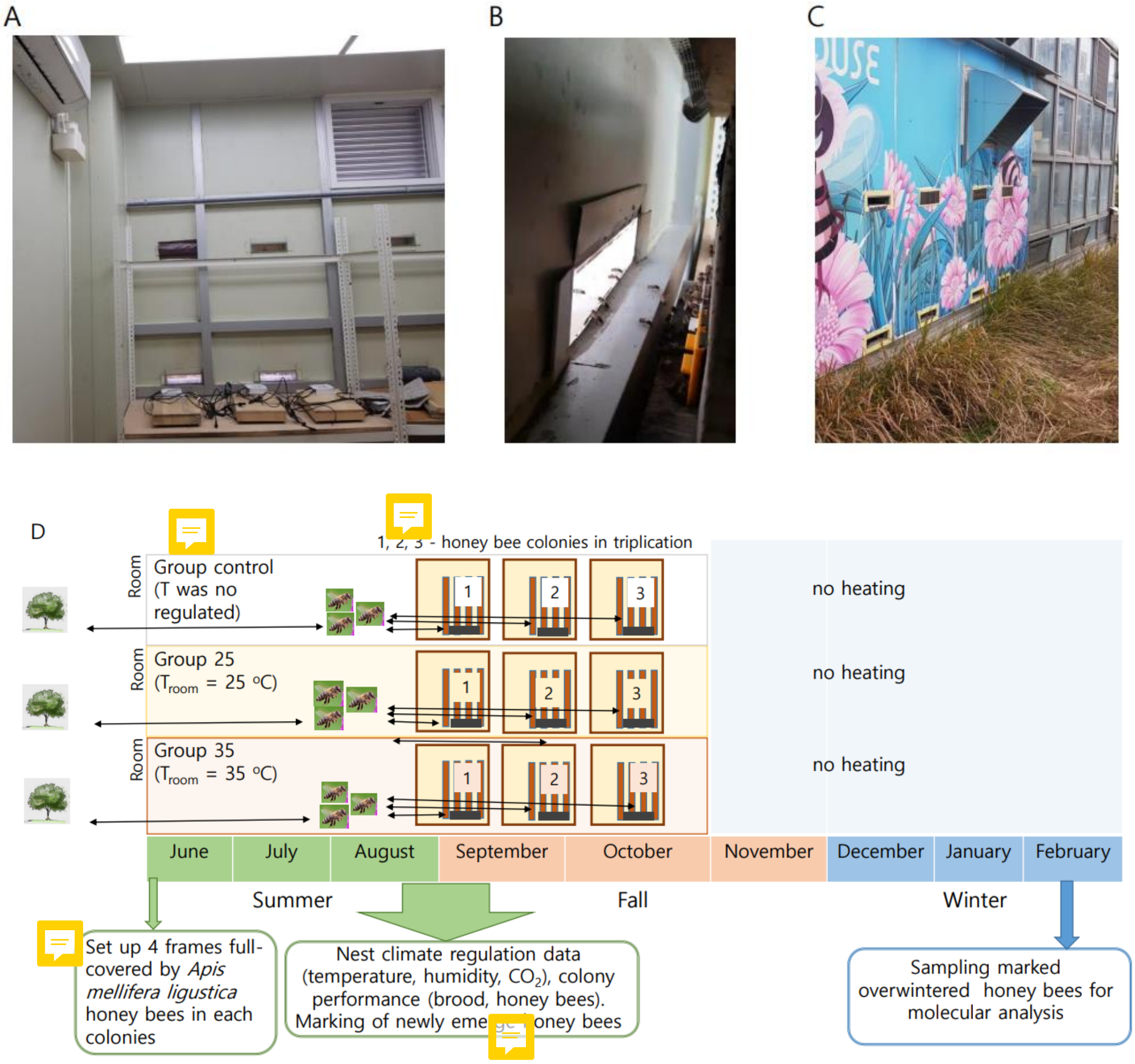

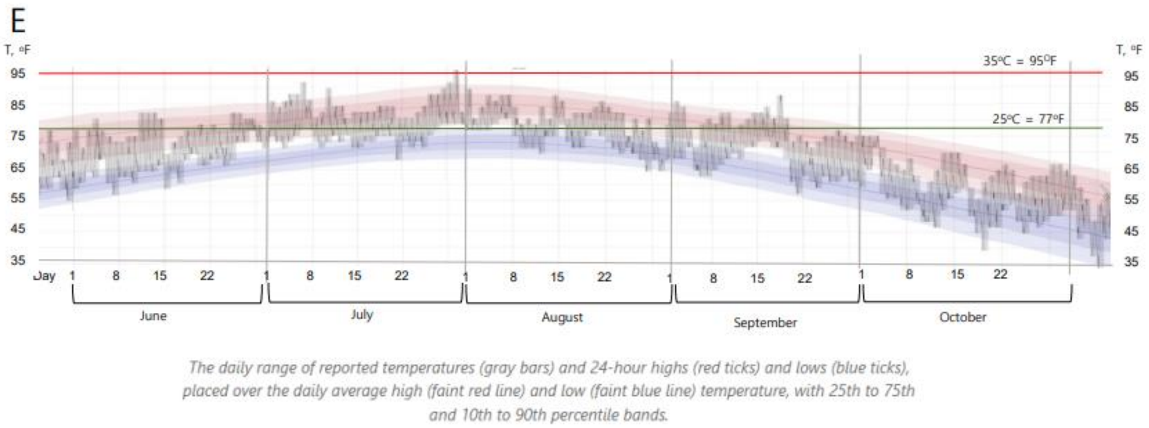

Fig 6. Overview of the experimental design (A) The room environment before the experiment. **(B)** The process of honey bees freely flying outside from the experimental room. **(C)** The view of the room and the entrance for honey bees outside. **(D)** Timeline of the experiment. **(E)** Ambient temperature of the Group control, Group 25 (green line), and Group 35 (red line) under the temperature experiment in 2022, Incheon, Republic of Korea. The data were obtained from https://weatherspark.com/h/m/142040/2022/10/Historical-Weather-in-October-2022-in-Incheon-South-Korea#Figures-Temperature.

The experimental colonies housed first-year young sister queens, naturally mated and beginning egg-laying in May 2022. Prior to the experiment in June, colonies were standardized in terms of the number of frames (total 4), brood population (3 frames), honey amount (1 frame), and the number of honey bees. Colony strength was assessed through visual inspection of adult and brood populations both before and during the experiment (Delaplane et al., 2013).

Two rooms equipped with a climate-control system were established to maintain honey bee colonies at 25°C (Group 25) and 35°C (Group 35), simulating climate warming without day and night temperature differences. These

temperatures were selected to mimic the effects of climate change, with 25°C representing the summer average and 35°C being the highest recorded in Incheon City under typical conditions. However, the highest temperature was not naturally reached during the summer of 2022 **(Fig 6E).** Other environmental conditions were not controlled. Light was used during colony inspections. In the third room, housing honey bee colonies, the control group was provided with free ventilation without additional heating. All honey bees had equal opportunities to collect pollen and nectar around the apiary.

On September 18th and 25th, 2022, two combs with sealed brood were placed in an incubator set at 34°C. Upon hatching, the workers were individually marked with colored paint on their thoraxes and then reintroduced into their original colony. Throughout the previous summer, as well as in September and October of 2022, the honey bee colonies were subjected to varying room ambient temperatures. Afterward, the controlled temperatures in the rooms were turned off, and the honey bee colonies were overwintered in the same rooms under natural temperature conditions.

The overwintered marked honey bees, aged approximately 4.5 months, were sampled on February 5th, 2023, a time when queens had not yet begun laying eggs and the overwintered bees were expected to have successfully survived the winter. Forty honey bees from each colony were sampled and individually placed

in labeled 50 ml tubes, then stored at -80 °C before analysis.

Brood microclimate measurements

The temperature, humidity (AM2320, BeeOnFarm, Korea), and CO_2 concentration (BO-100, BeeOnFarm, Korea) sensor systems were placed on the top and middle of each colony in August 2022. The data logging system was included in the sensor system because it provided a real-time clock. Data consisting of time (seconds), CO_2 (in ppm), temperature (in °C), and relative humidity (as a percentage) were saved, with approximately three measurements in minutes to approximately one file per day. The data from 6 honey bee colonies kept in each room (three at 25°C and three at 35°C) were collected over 15 days from 11 to 25 August. Average values were used for statistical calculations.

Hypopharyngeal gland (HG) measurements

The Acinus size from HGs of overwintered honey bees Groups control, 25 and 35 were measured to determine whether differed depending on the ambient temperature in the previous fall season. Thirty bees were collected from each colony, flash frozen in liquid nitrogen, and maintained at −80 °C until their glands were measured. For each bee, the HGs were dissected into 1× phosphate-buffered saline (PBS) with 0.1% Tween® 20 and visualized at 40× magnification. The area (mm^2) of a minimum of 10 randomly selected Acinus per bee was measured using

Nikon Eclipse E200 (Nikon, Tokyo, Japan) Toupview (Touptek, Hangzhou, China) software. Only Acinus with clear borders were used. Acinus areas were averaged within individuals.

Fat body lipid content

The Fat body lipid content in the abdomen of overwintered honey bees was studied in early February by the standard method (Martin, 1969; Human et al., 2013). Ten honey bees were collected from each colony; 90 honey bees were flash frozen in liquid nitrogen and maintained at −80 °C until their abdomens were dissected and their guts were removed. Pools of 10 abdomens were weighed on a scale (name, company, country) before and after drying to a constant weight in a fume hood (SSFH-1000, Shinsaeng, Korea). The total lipid content was determined by measuring the decrease in weight following the removal of lipids from the dried tissue through extraction in 5 ml portions of diethyl ether (179272-1 L; Sigma–Aldrich, USA).

RNA Extraction and cDNA Synthesis

A total of 90 honey bee (10 winter bees from each colony) *Apis mellifera ligustica* colonies were sampled in labeled tubes and stored at -80°C. Three honey bees from each colony were analyzed. RNA was extracted from the brain for *HSP70*, *JHAMT*, *ilp1*, *ilp2*, and *TOR1* expression analysis and from the abdomen for *vg*

gene expression analysis. Total tissue RNA (brain or abdomen) was extracted from 15 randomly selected bees in each group using a Qiagen RNeasy Mini Kit (#74,104; Qiagen, Valencia, CA, USA). The tissues in each replicate were brain tissue for *TOR1*, *ilp1*, *ilp2*, *JHAMT*, and *HSP70* and abdomen for *vg* gene expression. The total RNA concentration and purity were quantified using OD260/OD280 values between 1.8 and 2.0. Next, reverse transcription was performed using an RNA to cDNA EcoDryTM Premix (Oligo dT) kit (Takara, Japan). The reverse transcription reaction mixture included 50 ng/µl total RNA (with the clear volume calculated for each sample) and RNase-free water for a total volume of 20 µl. Reverse transcription was conducted at 42°C for 60 min, followed by heating at 70°C for 10 min.

Quantitative real-time PCR

The relative expression of six genes related to nutrition (*vg*, *ilp1*, *ilp2*, *TOR1*), development (*JHAMT*), and stress response (*HSP70*) was measured. The housekeeping gene *RpL32* (Ribosomal protein L32) was used as an endogenous control. The PCR primer sequences are shown in **S1 Table.**

The reaction conditions were optimized. The qRT–PCR mixture had a volume of 20 µl which included 2 µl of template cDNA, 10 µl of Green qPCR Master Mix, 1 µl of upstream and downstream primers (5 pM/µl), and 6 µl of nuclease-free water (AM9930, Invitrogen, USA). Quantitative real-time PCR (qRT–PCR)

was conducted using Brilliant III Ultra-Fast SYBR® Green qPCR Master Mix (600882, Agilent Technologies, USA) on an AriaMx Real-Time PCR System (Agilent Technologies LDA, Malaysia) with AriaMx 96 Well Plates, Skirted, LP (401490, Agilent Technologies, USA) covered by MicroAmp™ Optical Adhesive Film (4311971, Thermo Fisher Scientific, USA).

The qRT–PCR amplification procedure was as follows: initial denaturation at 95°C for 10 minutes; 40 cycles of denaturation at 95°C for 30 seconds, annealing at 60°C for 25 seconds, and extension at 72°C for 15 seconds. Each sample was technically replicated three times. Data analysis was performed using Agilent AriaMx version 2.0 analysis software. Relative gene expression data were analyzed using the 2^(-Delta Delta C(T)) method (Livak and Schmittgen, 2001; Bustin et al., 2009).

Confirmation of amplicons in the RT–PCR products was performed by separating them through electrophoresis in a 2% agarose gel at 80 V for 40 minutes and analyzing them using a gel documentation system, the Gerix 1010 transilluminator (Biostep GmbH, USA). A Dyne 50 bp DNA Ladder (Cat. No. A701, DYNEBIO, Korea) was used as a reference.

Statistical analysis

The statistical analysis, designed as illustrated in **Fig 7**, was conducted using Microsoft Excel and XLSTAT software (Addinsoft Pearson Edition 2014,

Addinsoft, Paris, France). The characterization was performed using the following strategies: (a) classification of the marker dataset to find a combination of features that best separates different groups (supervised approach, Discriminant Analysis (DA)); (b) clustering to discover natural groupings in the absence of labeled information (unsupervised approach, Agglomerative Hierarchical Clustering (AHC)); and (c) modeling the relationship between independent variables and a dependent variable and dealing with multicollinearity in the marker dataset to rank the markers by importance for identifying overwintered honey bee task development (supervised approach, Elastic Net Regression).

Apis mellifera ligustica honey bees

Month of sampling		Group control<br>(3 colonies)	Group 25<br>(3 colonies)	Group 35<br>(3 colonies)
Experimental condition<br>(summer, September, and October)			T_{room} = 25 °C	T_{room} = 35 °C
**August<br>September**	Data set 1<br>(brood microclimate and colony development, marking of hatched honey bees)		Nest climate regulation	
	Statistics of data set 1		T-test ($p < 0.05$)	
**February**	Sampling of marked honey bees, n	(for physiology markers): 30+30+30<br>(for molecular markers): 10+10+10	30+30+30<br>10+10+10	30+30+30<br>10+10+10
	Data set 2	Two physiology markers (hypopharengial glands (HG) and fat body)<br>Molecular marker genes related to nutrition (*vg*, *ilp1*, *ilp2*, *TOR1*), development (*JHAMT*), and stress response (*HSP70*).		
	Statistics of data set 2	1. Discriminant analysis (define the variables with high discrimination score);<br>2. Agglomerative Hierarchical Clustering (check the natural groupings within dataset);<br>3. Elastic Net Regression (model the relationship between HG and fat body with molecular markers);<br>4. ANOVA, Duncan post-hoc test (comparison of significance within each variable, $p < 0.05$).		

Fig 7. Experimental conditions, sampling schedule, data sets, and statistical tests used for analysis

Specifically, the honey bees were categorized into groups, and the variables

with the highest discrimination scores were selected using DA because it determines the linear combination of variables and provides maximal separation between groups. To discriminate the group, Fisher distances were applied, which emphasize the linear combinations of variables that maximize the differences between groups.

AHC analysis provided a visual representation of the clustered dataset, revealing the arrangement of individual data points or observations based on their dissimilarity. The evaluation metric for clustering algorithms was the silhouette index. The score is bounded between (− 1) for incorrect clustering and (+ 1) for highly dense clustering.

Elastic Net Regression analysis was used to test two models of the relationship (Jaqaman and Danuser, 2006; Moses, 2017) between physiology and molecular markers. The dependent variables were Acinus size and Fat body weight. The seven independent variables were ranked by coefficient. Receiver operating characteristic (ROC) curves were generated to quantify how accurately regression analysis can discriminate between honey bee groups (Hajian-Tilaki, 2013). The ROC specificity and sensitivity plot demonstrated a perfectly fitted model linking physiology to molecular markers (gene expression profile). The area under the curve (AUC) can range from 0.5 to 1.0, with a preference for higher scores.

The mean, standard deviation, and variance of each gene were calculated using

descriptive statistics and visualized using the heat map module. For the gene expression analysis, ANOVA was used to test overall effects, followed by the Duncan post hoc test ($p < 0.05$) for multiple comparisons and the t-test for gene expression differences between groups of honey bees. The bar plots were generated using the online SRplot Official Website (https://www.bioinformatics.com.cn/en; accessed on 30 October 2023).

Supporting information

S1 Table. PCR primer sequences.

(PDF)

Acknowledgments

The authors thank BeeOnfarm company (Republic of Korea) for providing the equipment to record the in-hive microclimate. Mention of trade names or commercial products in this publication is solely to provide specific information and does not imply recommendation or endorsement by the INU.

Author Contributions

Conceptualization: Yumi Yun, Hyung-Wook Kwon

Data curation: Yumi Yun

Formal analysis: Olga Frunze, Ravil R. Garafutdinov, Hyun Jee Kim

Investigation: Hyung-Wook Kwon, Young-Eun Na

Methodology: Ravil R. Garafutdinov, Olga Frunze, Yumi Yun

Resources: Hyung-Wook Kwon, Young-Eun Na, Hyun Jee Kim

Software: Olga Frunze, Ravil R. Garafutdinov

Supervision:, Hyung-Wook Kwon, Young-Eun Na

Visualization: Olga Frunze

Writing-original draft: Olga Frunze, Yumi Yun

Writing-review&editing: Olga Frunze, Yumi Yun, Hyun Jee Kim, Hyung-Wook Kwon

Reference

1. Abou-Shaara H, Owayss A, Ibrahim Y., Basuny, N. A review of impacts of temperature and relative humidity on various activities of honey bees. Insectes Soc. 2017; 64: 455 – 463.
2. Alaux C, Soubeyrand S, Prado A, Peruzzi M, Maisonnasse A, Vallon J et al. Measuring biological age to assess colony demographics in honeybees. PLoS ONE. 2018; 13(12):e0209192.
3. Ali H, Alqarni AS, Iqbal J, Owayss AA, Raweh HS, Smith BH. Effect of season and behavioral activity on the hypopharyngeal glands of three honey bee *Apis mellifera* L. races under stressful climatic conditions of central Saudi Arabia. J Hymenopt Res. 2019; 68: 85-101.
4. Amdam GV, Aase AL, Seehuus SC, Kim Fondrk M, Norberg K, Hartfelder K. Social reversal of immunosenescence in honey bee workers. Exp Ger Ontol. 2005; 40: 939e947.
5. Amdam GV, Omholt SW. The regulatory anatomy of honeybee lifespan. J Theor Biol. 2002; 216: 209e228.
6. Ament SA, Corona M, Pollock HS, Robinson GE. Insulin signaling is involved

in the regulation of worker division of labor in honey bee colonies. Proc Natl Acad Sci USA. 2008; 105: 4226-4231.

7. Barth JMI, Szabad J, Hafen E, Köhler K. Autophagy in Drosophila ovaries is induced by starvation and is required for oogenesis. Cell Death Differ. 2010; 18: 915.
8. Bencsik M, McVeigh A, Tsakonas C, Kumar T, Chamberlain L, Newton MI. A Monitoring System for Carbon Dioxide in Honeybee Hives: An Indicator of Colony Health Sensors (Basel). 2023; 23(7):3588.
9. Brejcha M, Prušáková D, Sábová M, Peska V, Černý J, Kodrík D, Konopová B, Čapková Frydrychová R. Seasonal changes in ultrastructure and gene expression in the fat body of worker honey bees. J Insect Physiol. 2023; 146:104504.
10. Bresnahan ST, Döke MA, Giray T, Grozinger CM. Tissue-specific transcriptional patterns underlie seasonal phenotypes in honey bees (*Apis mellifera*). Mol Ecol. 2022; 31:174-184.
11. Bustin SA, Benes V, Garson JA, et al. The MIQE guidelines: minimum information for publication of quantitative real-time PCR experiments. Clin Chem. 2009; 55:611-622.
12. Chowański S, Walkowiak-Nowicka K, Winkiel M, Marciniak P, Urbański A, Pacholska-Bogalska J. Insulin-Like Peptides and Cross-Talk With Other Factors in the Regulation of Insect Metabolism. Front Physiol. 2021; 12:701203.
13. Corby-Harris V, Snyder L, Meador C. Fat body lipolysis connects poor nutrition to hypopharyngeal gland degradation in *Apis mellifera*. J Insect Physiol. 2019; 116:1-9.
14. Corby-Harris V, Snyder LA. Measuring Hypopharyngeal Gland Acinus Size in Honey Bee (*Apis mellifera*) Workers. J Vis Exp. 2018; 139:58261.
15. Corona M, Velarde RA, Remolina S, Moran-Lauter A, Wang Y, Hughes KA et

al. Vitellogenin, juvenile hormone, insulin signaling, and queen honey bee longevity. Proc Natl Acad Sci USA. 2007; 104:7128-7133.

16. Coulibaly KA, Majeed MZ, Sayed SM, Yeo K. Simulated Climate Warming Influenced Colony Microclimatic Conditions and Gut Bacterial Abundance of Honeybee Subspecies *Apis mellifera ligustica* and *A. mellifera sinisxinyuan*. J Apic Sci. 2022; 66: 15-27.
17. Crailsheim K. Dependence of protein-metabolism on age and season in the honeybee (*Apis mellifica carnica* Pollm). J. Insect Physiol. 1986; 32:629–34.
18. Daniels BC, Wang Y, Page RE Jr, Amdam GV. Identifying a developmental transition in honey bees using gene expression data. PLoS Comput Biol. 2023; 9(9):e1010704.
19. Delaplane KS, van der Steen J, Guzman-Novoa E. Standard methods for estimating strength parameters of *Apis mellifera* colonies. J Apic Res. 2013; 52: 1-12.
20. Denlinger DL. Insect diapause: from a rich history to an exciting future. J Exp Biol. 2023; 226 (4): jeb245329.
21. Diebel LWM, Rockwood K. Determination of Biological Age: Geriatric Assessment *vs* Biological Biomarkers. Curr Oncol Rep. 2021; 23: 104.
22. Döke MA, Frazier M, Grozinger CM. Overwintering honey bees: biology and management. Curr Opin Insect Sci. 2015; 10:185-193.
23. Egi Y, Sakamoto K. Genome-wide screening of genes involved in programming diapause in the next generation in silkworm, *Bombyx mori* (Lepidoptera: Bombycidae). Eur J Entomol. 2022; 119:405-412.
24. Elekonich MM, Schulz DJ, Bloch G, Robinson GE. Juvenile hormone levels in honey bee (*Apis mellifera* L.) foragers: foraging experience and diurnal variation. J Insect Physiol. 2001; 47:1119e1125.
25. Fahrbach SE, Nambu JR, Schwartz LM. Programmed cell death in insects.

Insect Mol Biol Biochem. 2012; 12:419–449.

26. Fluri P, Lüscher M, Wille H, Gerig L. Changes in weight of the pharyngeal gland and hemolymph titers of juvenile hormone, protein and vitellogenin in worker honey bees. J Insect Physiol. 1982; 28:61-68.
27. Fluri P, Wille H, Gerig L, Luscher M. Juvenile hormone, vitellogenin and haemocyte composition in winter worker honeybees (*Apis mellifera*). Experientia 1977; 33: 1240–1241.
28. Goodman WG, Granger NA. The juvenile hormones. In: Lawrence IG, Kostas I, Sarjeet SG. Comprehensive Molecular Insect Science. Elselvier. 2005; 319-408.
29. Gray A, Adjlane N, Arab A, Ballis A, Brusbardis V, Dougla AB. et al. Honey bee colony loss rates in 37 countries using the COLOSS survey for winter 2019–2020: the combined effects of operation size, migration and queen replacement. J Apic Res. 2023; 62:204-210.
30. Guidugli KR, Nascimento AM, Amdam GV, Barchuk AR, Omholt S, Simoes ZL et al. 2005. Vitellogenin regulates hormonal dynamics in the worker caste of a eusocial insect. FEBS Lett. 2005; 579:4961e4965.
31. Hajian-Tilaki K. Receiver Operating Characteristic (ROC) Curve Analysis for Medical Diagnostic Test Evaluation. Caspian J Intern Med. 2013; 4:627-635.
32. Hartfelder K, Engels W. Social insect polymorphism: hormonal regulation of plasticity in development and reproduction in the honeybee. Curr Top Dev Biol. 1998; 40:45e77.
33. Hristov P, Shumkova R, Palova N, Neov B. Factors Associated with Honey Bee Colony Losses: A Mini-Review. Vet Sci. 2020; 7:166.
34. Human H, R. Brodschneider V, Dietemann G, Dively JD, Ellis E, Forsgren I, et al. Miscellaneous standard methods for *Apis mellifera* research. J Apic Res. 2013; 52:1-53.

35. Insolia L, Molinari R, Rogers SR, Williams GR, Chiaromonte F, Caloviet M. Honey bee colony loss linked to parasites, pesticides and extreme weather across the United States. Sci Rep. 2022: 12:20787.

36. Jaqaman K, Danuser G. Linking data to models: data regression. Nat Rev Mol Cell Biol. 2006; 7:813–819.

37. Johnson SC, Rabinovitch PS, Kaeberlein M. mTOR is a key modulator of aging and age-related disease. Nature. 2013; 493:338-345.

38. Kaur R, Singh D. Molecular markers a valuable tool for species identification of insects: A review. Ann Entomol. 2020; 38:1-20.

39. Knoll S, Pinna W, Varcasia A, Scala A, Cappa MG. The honey bee (*Apis mellifera* L., 1758) and the seasonal adaptation of productions. Highlights on summer to winter transition and back to summer metabolic activity. A review. Livest Sci. 2020; 235: 104011.

40. Kovac H, Stabentheiner A, Brodschneider R. Contribution of honeybee drones of different age to colonial thermoregulation. Apidologie. 2009; 40:82-95.

41. Laplante M, Sabatini DM. mTOR signaling in growth control and disease. Cell. 2012; 149:274-293.

42. Li W, Huang ZY, Liu F, Li Z, Yan L, et al. Molecular Cloning and Characterization of Juvenile Hormone Acid Methyltransferase in the Honey Bee, *Apis mellifera*, and Its Differential Expression during Caste Differentiation. PLoS ONE. 2013; 8:e68544.

43. Livak KJ, Schmittgen TD. Analysis of relative gene expression data using real-time quantitative PCR and the 2(-Delta Delta C(T)) Method. Methods. 2001; 25:402–408.

44. Martin JS. Lipid composition of fat body and its contribution to the maturing oöcytes in *Pyrrhocoris apterus*. J Insect Physiol. 1969; 15:1025-1045.

45. Medrzycki P, Sgolastra F, Bortolotti L, Bogo G, Tosi S, Padovani E, Porrini C.&

Sabatini AG. Influence of brood rearing temperature on honey bee development and susceptibility to poisoning by pesticides. J Apic Res. 2010; 49:52-59.

46. Moses AM. Statistical Modeling and Machine Learning for Molecular Biology. CRC Press Taylor & Francis Group. 2017: 280.
47. Münch D, Kreibich CD, Amdam GV. Aging and its modulation in a long-lived worker caste of the honey bee. J Exp Biol. 2013; 216: 1638–1649.
48. Nilsen KA, Ihle KE, Frederick K, Fondrk MK, Smedal B, Hartfelder K, et al. Insulin-like peptide genes in honey bee fat body respond differently to manipulation of social behavioral physiology. J Exp Biol. 2011; 1:1488-97.
49. Patel A, Fondrk MK, Kaftanoglu O, Emore C, Hunt G Frederick K, et al. The making of a queen: TOR pathway is a key player in diphenic caste development. PLoS ONE. 2007; 2: e509.
50. Quinlan GM, Feuerborn C, Hines HM, Grozinger CM. Beat the heat: thermal respites and access to food associated with increased bumble bee heat tolerance. J Exp Biol. 2023; 226: jeb245924.
51. Rodrigues MA, Flatt T. Endocrine uncoupling of the trade-off between reproduction and somatic maintenance in eusocial insects. Curr Opin Insect Sci. 2016; 16:1e8.
52. Sarioğlu-Bozkurt A, Topal E, Güneş N, Üçeş E, Cornea-Cipcigan M, Coşkun İ, Cuibus L, Mărgăoanet R. Changes in Vitellogenin (Vg) and Stress Protein (*HSP 70*) in Honey Bee (*Apis mellifera anatoliaca*) Groups under Different Diets Linked with Physico-Chemical, Antioxidant and Fatty and Amino Acid Profiles. Insects. 2022; 13:985.
53. Simpson J. Nest Climate Regulation in Honey Bee Colonies. Science. 1961; 133:1327–1333.
54. Smedal B, Brynem M, Kreibich C, Amdam G. Brood pheromone suppresses physiology of extreme longevity in honeybees (*Apis mellifera*). J Exp Biol.

2009; 212: 3795-801.

55. Szentgyörgyi H, Czekońska K., Tofilski A. Honey bees are larger and live longer after developing at low temperature. J Therm Biol. 2018; 78:219-226.
56. Winston ML. The Biology of the Honey Bee. Harvard University Press, Cambridge. 1987; 294.
57. Zhang W, Wang L, Zhao Y, Wang Y, Chen C, Hu Y, et al. Single-cell transcriptomic analysis of honeybee brains identifies vitellogenin as a caste differentiation-related factor. iScience. 2022; 25:104643.
58. Zhang Y, Liu Y, Zhang J, Guo Y, Ma E. Molecular Cloning and mRNA Expression of Heat Shock Protein Genes and Their Response to Cadmium Stress in the Grasshopper Oxya chinensis. PLoS ONE. 2015; 10: e0131244.
59. Zoncu R, Efeyan A, Sabatini DM. mTOR: from growth signal integration to cancer, diabetes and aging. Nat Rev Mol Cell Biol. 2011; 12:21-35.

Funding: This work was carried out with the support of the Cooperative Research Program for Agriculture Science & Technology Development (Project No. RS-2023-00232749) and the Priority Research Centers Program through the National Research Foundation of Korea (NRF) funded by the Ministry of Education (2020R1A6A1A03041954).

Competing interests: The authors have declared that no competing interests exist.

Abbreviations: DA, discriminant analysis; AHC, Agglomerative Hierarchical Clustering; FD, Fisher distance; HG, hypopharyngeal glands; JH, juvenile hormone; *JHAMT, Juvenile Hormone Acid Methyltransferase; vg, vitellogenin; ilp1, insulin-like peptide 1; ilp2, insulin-like peptide 2; HSP70, heat shock protein 70; TOR1, target of rapamycin 1; RpL32, Ribosomal protein L32.*

Click here to access/download
Supporting Information
Supporting information.docx

Dear Editor-in-Chief, Reviewer 1 and Reviewer 2!

Thank you for the time and effort spent reviewing our manuscript and suggesting some important points to consider.

Our replies are marked by **green color** after the symbol **R** (reply) and question numbers 1 (R1) – 4 (R4) for the Academic Editor, 5 (R5) – 20 (R20) for the first reviewer's comments and (21 (R21) – 25 (R25)) for the second reviewer's comments. Also, we copied these modifications in response for convenience when it was available.

Please, find below the reviewer comments (black) and our response (**green**)

Academic Editor Comments

Unfortunately,

1. the experimental design was not adequately explained throughout. For example, no plausible reason is given for the selection of the genes tested. As a result, the reader does not understand the purpose of the experiments. **R1:** Thank you for bringing this to our attention. Lines 80-99 in the Introduction provide explanations for each gene used, with the gray color highlighting the rationale behind their collective use. All of them can be seen in the screenshot below of the ORIGINAL manuscript (before current corrections).

hormone JH (Elekonich et al., 2001; Amdam et al., 2005; Guidugli et al., 2005), other potential markers such as *JHAMT, ilp1, ilp2, TOR1*, and *HSP70* genes were examined in this study. The enzyme involved in JH biosynthesis, *Juvenile Hormone Acid Methyltransferase (JHAMT)*, exhibits changes in expression direction similar to JH titer and can serve as an alternative marker to JH (Goodman and Granger, 2005; Li et al., 2013; Egi and Sakamoto, 2022). A positive correlation with JH was observed in the *insulin-like peptide, ilp1,* whose expression increases in the brains of foragers and decreases during continued foraging (Corona et al., 2007; Ament et al., 2008). The *insulin-like peptide, ilp2* gene is expected to have task-related expression as it is highly expressed in nurse tissue and plays a role in increasing cell size and number in individual organs (Zhang et al., 2022). Additionally, the *TOR1* gene, a nutrient-sensing kinase, along with the *ilp1* and *ilp2* genes, participates in the mobilization of nutritional resources (autophagy) from tissues such as the fat body and glands in *Drosophila* (Barth et al., 2010). Moreover, the *TOR* pathway is implicated in disease and nutrition in insects (Patel et al., 2007; Laplante and Sabatini, 2012; Johnson et al., 2013), and mammals (Zoncu et al., 2011), and it also regulates longevity extension in social insects (Rodrigues and Flatt, 2016). Lastly, the stress marker *heat shock protein 70 (HSP70)* was chosen as it is detected at low levels in nurses but shows increased expression in foragers (Sario˘glu-Bozkurt et al., 2022).

5

While all these markers are expected to be important for identifying aging in honey bees, testing them together requires further investigation.

This research aimed to simulate the influence of climate warming on colony performance in early fall and compare the aging of overwintered *Apis mellifera ligustica* honey bees. The experiment considered ambient temperatures of 25°C (Group 25) and 35°C (Group 35), which simulated the average and high summer temperatures in Incheon (Republic of Korea), respectively, while natural conditions were maintained in the Group control. The temperature experiments lasted throughout the previous summer, September, and October, with the colonies overwintering under the same conditions. Colony performance, including brood microclimate, was investigated in August and September because that month honey bee transformation from summer to winter physiology started in the Republic of Korea. The aging of honey bees at the end of diapause (broodless period) in February was determined by eight markers, including two physiological markers (Acinus size and Fat body weight) and six molecular gene markers related to the task (*vg* and *JHAMT*), nutrition (*ilp1, ilp2*, and *TOR1*), and stress response (*HSP70*). Our findings suggest that temperature decreases around winter worker broods from Group 25 in the fall lead to their slower physiological aging in winter compared to Group 35 workers, supported by physiology and task-related markers. Furthermore, we observed a relation between aging and

6

If it doesn't seem sufficient, we revised the Introduction, Material and Method, and Discussion chapters to ensure greater clarity for readers, regardless of their level of expertise. You can find numerous corrections addressing this in the resubmitted manuscript.

2. Furthermore, it remains unclear why **no monitoring of the bee colonies** was carried out over the experimental period and why the **chronological age** of the overwintering bees was not stated or estimated. **R2:** We appreciate the editor's comment regarding **the monitoring of the bee colonies**. However, while the colony fitness was estimated, it was not included in the manuscript initially. Therefore, this data has been added to Figure 1 (D, E, and G). Additionally, an explanation of this data has been provided in the appropriate chapter adjacent to Figure 1.

climate inside colonies influences the physiology of winter honey bees and the success of overwintering.

User
삭제됨: overwintering

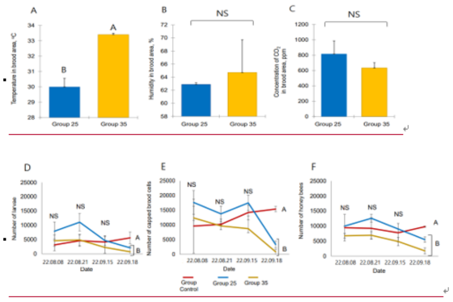

Fig 1. Colony performance in honey bees treated under different ambient temperatures (08 August – 18 September 2022). **(A)** Temperature in brood area (°C). **(B)** Humidity in brood area (%). **(C)** Concentration of CO_2 in brood area (ppm). **(D)** Number of larvae. **(E)** Number of capped brood cells. **(F)** Number of honey bees. Statistical significance was determined using one-way ANOVA and Duncan post hoc tests ($p < 0.05$, indicated by different letters above the columns); bars in the graphs represent the mean ± SD.

SD – Standard Deviation, NS – No Significance. Honey bee colonies in Group 25 were maintained in room conditions at 25°C; colonies in Group 35 were kept in room conditions at 35°C, while Group Control colonies were maintained under natural conditions.

User
[1] 이동함(삽입)

User
[2] 이동함(삽입)

The mean and standard deviation of the nest temperature for honey bee

The chronological age of the overwintering bees was mentioned, but we overlooked providing this explanation in the initial manuscript. Consequently, we have made modifications to the "Material and Methods" chapter under the section "Experimental bees". For your convenience, please find the corrections below in the screenshot.

housing honey bee colonies, the control group was provided with free ventilation without additional heating. All honey bees had equal opportunities to collect pollen and nectar around the apiary.

On September 18th and 25th, 2022, two combs with sealed brood were placed in an incubator set at 34°C. Upon hatching, the workers were individually marked with colored paint on their thoraxes and then reintroduced into their original colony. Throughout the previous summer, as well as in September and October of 2022, the honey bee colonies were subjected to varying room ambient temperatures. Afterward, the controlled temperatures in the rooms were turned off, and the honey bee colonies were overwintered in the same rooms under natural temperature conditions.

The overwintered marked honey bees, aged approximately 4.5 months, were sampled on February 5th, 2023, a time when queens had not yet begun laying eggs and the overwintered bees were expected to have successfully survived the winter. Forty honey bees from each colony were sampled and individually placed in labeled 50 ml tubes, then stored at -80 °C before analysis.

Brood microclimate measurements

3. In many cases, the references given are not appropriate. **R3:** Thank you for bringing this to our attention. We have rechecked each reference and made corrections where necessary. Please find the corrections throughout the entire manuscript.

4. Due to problems with English grammar and syntax, the article is difficult to read and does not correspond to standard English in its current state. **R4:** Thank you for bringing this to our attention. The English was corrected by native speakers before resubmission.

Despite all the criticism, however, some of the results seem very interesting. Therefore, you can understand my decision as an "open reject" decision. This means that if you are able to react adequately to the main points of criticism from the Reviewers, you can resubmit to PLOS One at any time. If resubmitted, the manuscript is considered a new submission. Thank you. Because of your suggestion, we resubmit to PLOS ONE.

Reviewer #1:

General comments.

5. There are many issues with English grammar, syntax, and scientific writing. The paper should be carefully reviewed and improved. **R5:** Thank you for bringing this to our attention. The English was corrected by native speakers before resubmission.

6. Many references are not directly related to the point in question. The authors should check carefully that suitable references are cited in the manuscript. **R6:** Thank you for bringing this to our attention. We have rechecked each reference and made corrections where necessary. Please find the corrections throughout the entire manuscript.

7. The overall experimental design needs to be adequately explained. For example: 1) Explain the rationale of the two temperatures selected. 2) Why did the authors control the temperature until October? I assume the authors want to manipulate the environmental signals before the colony starts overwintering, with the idea that these signals change the physiology of worker/ larvae and induce the subsequent development of winter bees. However, the rationale of the experiment needs to be carefully explained. **R7:** We appreciate the reviewer's comment regarding the experimental design. In response to point 1), we agree that the selection of two temperatures was omitted from the manuscript. We have now included this explanation. For your convenience, please find the corrections in the screenshot below.

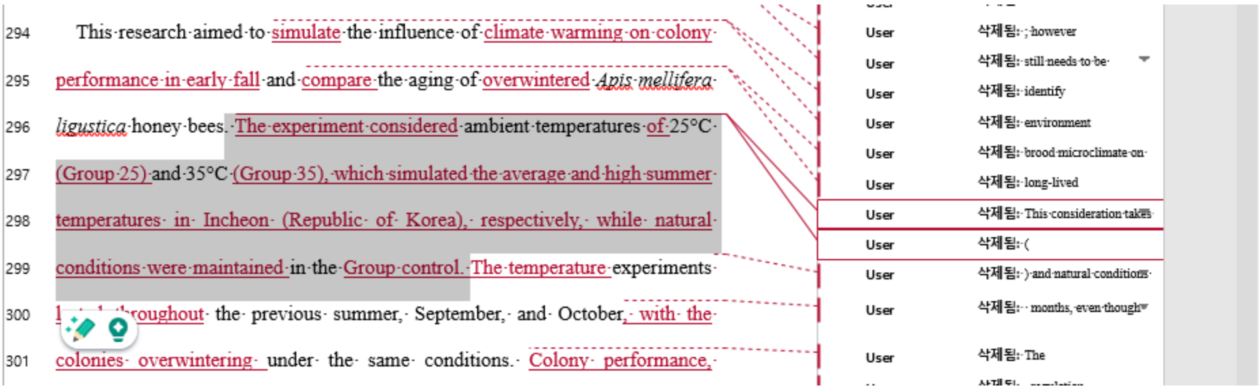
This research aimed to simulate the influence of climate warming on colony performance in early fall and compare the aging of overwintered *Apis mellifera ligustica* honey bees. The experiment considered ambient temperatures of 25°C (Group 25) and 35°C (Group 35), which simulated the average and high summer temperatures in Incheon (Republic of Korea), respectively, while natural conditions were maintained in the Group control. The temperature experiments [illegible]roughout the previous summer, September, and October, with the colonies overwintering under the same conditions. Colony performance,

User 삭제됨: ; however
User 삭제됨: still needs to be
User 삭제됨: identify
User 삭제됨: environment
User 삭제됨: brood microclimate on
User 삭제됨: long-lived
User 삭제됨: This consideration takes
User 삭제됨: (
User 삭제됨:) and natural conditions
User 삭제됨: months, even though
User 삭제됨: The

In response to point 2), the reviewer's suggestion was correct; our intention was indeed to manipulate environmental signals before the colony enters the overwintering phase. We have now included this explanation in the manuscript.

8 One of the main objectives was to identify the expression patterns associated with overwintering bee physiology; the **design should have included comparisons between summer and winter bees**. **R8:** We appreciate the reviewer's comment on the experimental design. Our focus was specifically on the influence of outside temperature during the season-related transition of longevity, rather than comparing summer and winter bees. However, such a comparison could be explored in future research.

9. In addition to estimating the physiological or biological age with molecular markers, the authors should have also estimated the **chronological age** of the overwintering bees. **R9:** Thank you for bringing this to our attention. The chronological age of the overwintering bees was mentioned, but we overlooked providing this explanation in the initial manuscript. Consequently, we have made modifications to the "Material and Methods" chapter under the

section "Experimental bees". For your convenience, please find the corrections below in the screenshot.

housing honey bee colonies, the control group was provided with free ventilation without additional heating. All honey bees had equal opportunities to collect pollen and nectar around the apiary.

On September 18th and 25th, 2022, two combs with sealed brood were placed in an incubator set at 34°C. Upon hatching, the workers were individually marked with colored paint on their thoraxes and then reintroduced into their original colony. Throughout the previous summer, as well as in September and October of 2022, the honey bee colonies were subjected to varying room ambient temperatures. Afterward, the controlled temperatures in the rooms were turned off, and the honey bee colonies were overwintered in the same rooms under natural temperature conditions.

The overwintered marked honey bees, aged approximately 4.5 months, were sampled on February 5th, 2023, a time when queens had not yet begun laying eggs and the overwintered bees were expected to have successfully survived the winter. Forty honey bees from each colony were sampled and individually placed in labeled 50 ml tubes, then stored at -80 °C before analysis.

Brood microclimate measurements

Specific points:

10. .Ln 21.″protecting nature” is a too general statement. Please use a more suitable wording.

R10: Accepted and corrected.

11. Ln 23-26. The sentence is unclear. **R11:** Accepted and corrected.

12. Ln 27-28. Quote: ” aging was evaluated through qPCR” I suggest complete this idea, writing something similar to: “aging was evaluated through the measurement of the expression of biomarkers of age by qPCR”. **R12:** Accepted and corrected.

14. Ln 54. In addition to the reviews, the authors should cite the original studies where winter vg and JH were measured. For example, Fluri et al.., 1977 and 1982. **R14:** Accepted and corrected.

15. Ln 63 Not only in the “end of the winter”, but during the winter. **R15:** Accepted and corrected.

16. Ln-74-76. In this sentence, at least two references are not properly cited: Corona et al 2007, and Amdam and Page 2010. These articles are not related to diapause in winter bees. **R16:** Accepted and corrected.

17. Lines 77-80. I agree that, theoretically, vg should decrease, and JH should increase in the

winter bees by the end of the winter. However, these changes should induce the transformation of the winter bees into spring foragers, not nurses. In any case, please add only the references directly supporting your argument: At least the three first references (Crailsheim, 1986, 1992; Nilsen et al., 2011) do not support it. **R17:** Accepted, rechecked, and corrected (citations). However, in beekeeping science and practice, the terms used to describe the overwintering condition of honey bees were not clearly defined compared to summer bees, where honey bees with different tasks and physiology were categorized into newly emerged, nurses, and foragers. When comparing summer and winter honey bees, it was found that the acinus, fat body, vg, and JH levels of winter bees were similar to those of nurses (Fluri et al., 1977). However, winter bees in diapause neither perform duties similar to those of summer nurses. Can we accurately refer to diapausing winter bees as "nurses" based solely on physiology and molecular analysis? This terminology requires further consideration and discussion. While I mentioned the duties of nurses after overwintering diapauses bees in the initial manuscript, there was no general term for this phenomenon. This issue has been addressed by refining the sentences and omitting the controversial point. The term "diapausing nurse honey bees" was used in the last edition of the manuscript.

18. Line 208-209. First, the authors should refer to relative levels (as indicated in Figure 6) rather than ddCT. Second, qPCR results are usually described, mentioning whether a comparison is significant and providing the p-value (rather than SD). I advise rewriting this section. Please compare as qPCR results are usually described in other studies. **R18:** Accepted, rewrote, and corrected

19. Line 310-322 This section needs a much better description of the colonies and how they were maintained during the experiment. For example, what was the hive size (one or two boxes)? One box What was the colony size (bee population)? Figure 1 (D, E, and H) visualized this data on new plots. Does the colonies were homogenized before the experiment? Yes. What was the age of the queen? 1 year Please also describe the rooms used in this experiment. We corrected Figure 6, and new photos and explanations in the chapter were done. For example: Were the rooms inside or outside the building in greenhouses? Could the bees fly out of the hive, or were they confined inside the hives? Could the bees fly out of the room? What were the light conditions used? **R19:** Thank you for bringing this to our attention. We revised the description of colonies to enhance clarity, and we made corrections to Figure 6 to improve the understanding of the experimental design. For your convenience, please find the corrections

below in the screenshot.

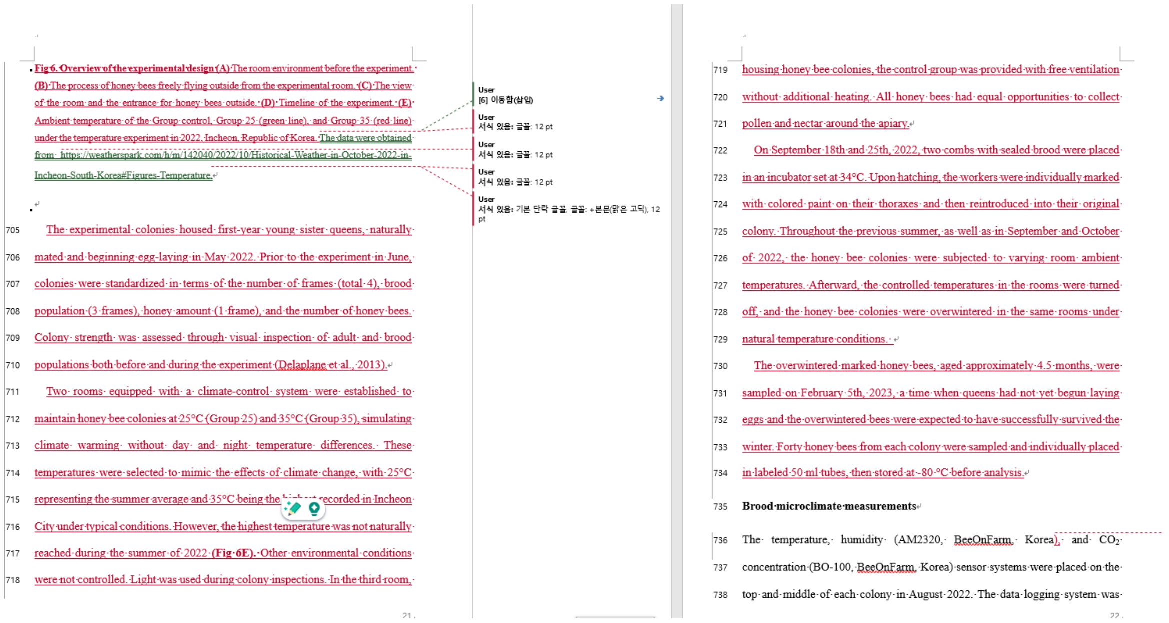

Fig 6. Overview of the experimental design (A) The room environment before the experiment. (B) The process of honey bees freely flying outside from the experimental room. (C) The view of the room and the entrance for honey bees outside. (D) Timeline of the experiment. (E) Ambient temperature of the Group control, Group 25 (green line), and Group 35 (red line) under the temperature experiment in 2022, Incheon, Republic of Korea. The data were obtained from https://weatherspark.com/h/m/142040/2022/10/Historical-Weather-in-October-2022-in-Incheon-South-Korea#Figures-Temperature.

User
[6] 이동함(삽입)
User
서식 있음: 글꼴: 12 pt
User
서식 있음: 글꼴: 12 pt
User
서식 있음: 글꼴: 12 pt
User
서식 있음: 기본 단락 글꼴, 글꼴: +본문(맑은 고딕), 12 pt

The experimental colonies housed first-year young sister queens, naturally mated and beginning egg-laying in May 2022. Prior to the experiment in June, colonies were standardized in terms of the number of frames (total 4), brood population (3 frames), honey amount (1 frame), and the number of honey bees. Colony strength was assessed through visual inspection of adult and brood populations both before and during the experiment (Delaplane et al., 2013).

Two rooms equipped with a climate-control system were established to maintain honey bee colonies at 25°C (Group 25) and 35°C (Group 35), simulating climate warming without day and night temperature differences. These temperatures were selected to mimic the effects of climate change, with 25°C representing the summer average and 35°C being the highest recorded in Incheon City under typical conditions. However, the highest temperature was not naturally reached during the summer of 2022 (Fig 6E). Other environmental conditions were not controlled. Light was used during colony inspections. In the third room, housing honey bee colonies, the control group was provided with free ventilation without additional heating. All honey bees had equal opportunities to collect pollen and nectar around the apiary.

On September 18th and 25th, 2022, two combs with sealed brood were placed in an incubator set at 34°C. Upon hatching, the workers were individually marked with colored paint on their thoraxes and then reintroduced into their original colony. Throughout the previous summer, as well as in September and October of 2022, the honey bee colonies were subjected to varying room ambient temperatures. Afterward, the controlled temperatures in the rooms were turned off, and the honey bee colonies were overwintered in the same rooms under natural temperature conditions.

The overwintered marked honey bees, aged approximately 4.5 months, were sampled on February 5th, 2023, a time when queens had not yet begun laying eggs and the overwintered bees were expected to have successfully survived the winter. Forty honey bees from each colony were sampled and individually placed in labeled 50 ml tubes, then stored at −80 °C before analysis.

Brood microclimate measurements

The temperature, humidity (AM2320, BeeOnFarm, Korea), and CO_2 concentration (BO-100, BeeOnFarm, Korea) sensor systems were placed on the top and middle of each colony in August 2022. The data logging system was

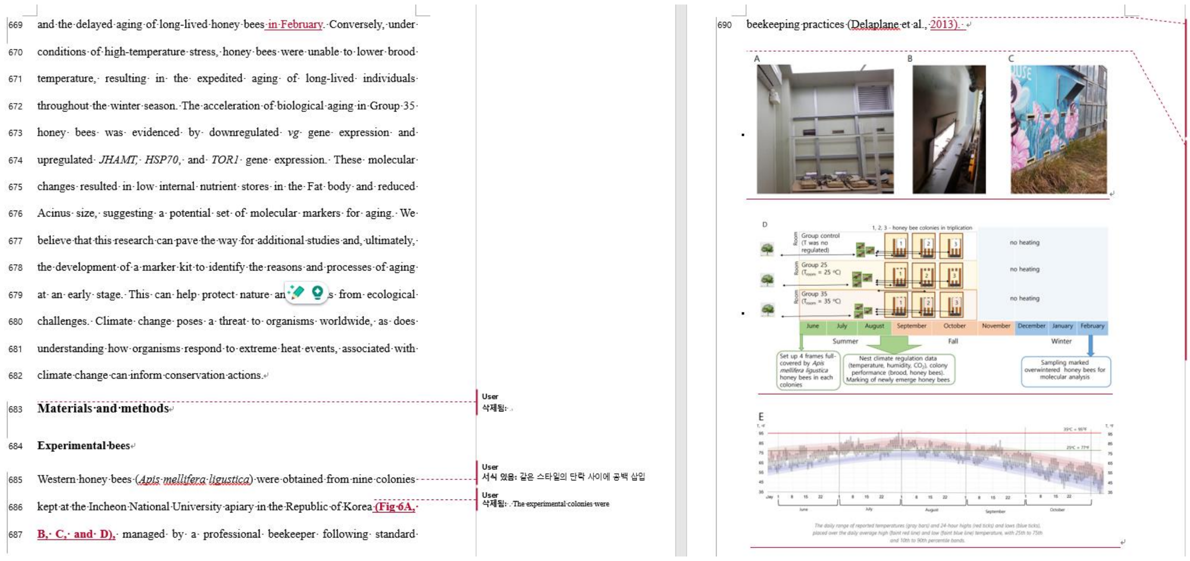

and the delayed aging of long-lived honey bees in February. Conversely, under conditions of high-temperature stress, honey bees were unable to lower brood temperature, resulting in the expedited aging of long-lived individuals throughout the winter season. The acceleration of biological aging in Group 35 honey bees was evidenced by downregulated *vg* gene expression and upregulated *JHAMT*, *HSP70*, and *TOR1* gene expression. These molecular changes resulted in low internal nutrient stores in the Fat body and reduced Acinus size, suggesting a potential set of molecular markers for aging. We believe that this research can pave the way for additional studies and, ultimately, the development of a marker kit to identify the reasons and processes of aging at an early stage. This can help protect nature an[illegible]s from ecological challenges. Climate change poses a threat to organisms worldwide, as does understanding how organisms respond to extreme heat events, associated with climate change can inform conservation actions.

Materials and methods

User
삭제됨: .

Experimental bees

Western honey bees (*Apis mellifera ligustica*) were obtained from nine colonies kept at the Incheon National University apiary in the Republic of Korea (Fig 6A, B, C, and D), managed by a professional beekeeper following standard beekeeping practices (Delaplane et al., 2013).

User
서식 있음: 같은 스타일의 단락 사이에 공백 삽입
User
삭제됨: . The experimental colonies were

20. Figure 6. The legend figure should explain the experimental design much better. For example, what was done differently between summer and fall? We corrected this point on Fig. 6D. What is the meaning of the three boxes depicted in September and October? Triplication of each treatment (three colonies). Are they colonies? What happened in November? Temperature control was off. **R20:** Thank you for bringing this to our attention. Accepted, rechecked, and corrected in the Materials and Methods chapter.

Reviewer #2:

The manuscript aims to investigate the impact of outside temperatures during summer on the

physiological and molecular characteristics of overwintering generation of honeybee workers. The analysis was conducted in February of the subsequent year. The size of the hypopharyngeal glands and the mass of the fat body were selected as physiological markers, while the transcription of the genes *tor, vg, hsp70,* and *jhamt* are chosen as molecular markers. The objective of this study was sort of to establish a relationship between the temperature within the hives and the aging rate of the wintering generation workers, as well as the bees' capacity to endure the winter season.

21. The text lacks an explanation and justification for the selection of tested genes. The authors assert that these markers are employed for the purpose of detecting the process of aging in bees. **R21:** Thank you for bringing this to our attention. This response has already been addressed in the comment 1 to the editor above. Please find the full response there.

22. Regarding *hsp70*, I cannot concur as I am unaware of any published data regarding the function of *hsp70* as an indicator of age in bees. **R22:** We appreciate the reviewer's comment. Some published data regarding the function of *hsp70* as an indicator of age in bees can be found below. Tissue-*Hsp70* basal levels show age-related changes in mice (de Toda et al., 2016). Additionally, evidence suggests that serum concentration of *hsp70* decreases with age in a normal human population (Njemini et al., 2011). Furthermore, *Hsp70* treatment has been shown to extend mean and maximum lifespan, improve learning and memory in old animals, increase curiosity, decrease anxiety, and help maintain synaptic structures that degrade with age in mice (Bobkova et al., 2015).

Therefore, our choice of *hsp70* as the marker to test aging was not random. However, as you correctly pointed out, in honey bee science, this gene is not frequently used for aging tests.

Reference for response:

Njemini, R., Bautmans, I., Onyema, O.O. et al. Circulating Heat Shock Protein 70 in Health, Aging and Disease. BMC Immunol 12, 24 (2011).

Irene Martínez de Toda, Carmen Vida, Eduardo Ortega, Mónica De La Fuente, Hsp70 basal levels, a tissue marker of the rate of aging and longevity in mice, Experimental Gerontology, Volume 84, 2016,Pages 21-28.

Bobkova, N. V., Evgen'ev, M., Garbuz, D. G., Kulikov, A. M., Morozov, A., Samokhin, A., Velmeshev, D., Medvinskaya, N., Nesterova, I., Pollock, A., & Nudler, E. (2015). Exogenous Hsp70 delays senescence and improves cognitive function in aging mice. Proceedings of the National Academy of Sciences of the United States of America, 112(52), 16006–16011.

23. The authors' references on page 5 are completely irrelevant. Additionally, it should be noted that the Brejcha 2023 reference does not pertain to the facts provided. I neglected to consult further sources. **R23:** Thank you for bringing this to our attention. We have rechecked each reference and made corrections where necessary. Please find the corrections throughout the entire manuscript.

24.The transcription of the tested genes in the hive 35 exhibits notable increases or decreases as compared to the other two hives. While the data may appear intriguing first, its interpretation is very simplistic and deceptive, particularly in relation to Tor signaling. The authors make mention of alterations in the quantity of nutrients within the fat body tissue. However, I was unable to locate any data offered in the text. The only section that provides information is the description of lipid extraction in the Material and Methods section. **R24:** We appreciate the reviewer's comment. The fat body weight showed no significant differences, as presented in Figure 4A and mentioned in the chapter "Comparative Analysis of the Biological Age of Overwintered Western Honey Bees through Physiological Markers" in the initial manuscript as well. However, before these changes can become significant, certain metabolic processes must first be initiated, which we discuss in the explanation of the TOR pathway. Although significant differences in fat body weight were not reached during the 20 experimental days, molecular analysis detected some processes that had already begun.

25. Based on published data it is well known that the formation of the overwintering generation is typically influenced by photoperiod and temperature, as well as by a decrease in brooding activity. This leads to changes in the hormonal settings of bees. Furthermore, it is worth noting that winter bees do not only emerge in August, as authors sort of claim. Some sources suggest that they can be observed as early as July and continue throughout the winter, depending on brooding activities. **R25:** We appreciate the reviewer's comment. Our experiments were conducted in the Republic of Korea, and we discussed this matter with scientists who have extensive experience in beekeeping in both Europe and Asia. All of them agreed that July is not a typical month for rearing overwintered honey bees. We believe that further investigation through organizations like Coloss, where this situation can be scientifically studied across different countries, would provide valuable insights, rather than relying solely on private discussions.

Therefore, (1) some author’s claims seem to me too simplified, and also, (2) the samples of bees that were tested may not have been originated from the experimental period of June -

August, when the temperature was altered. Furthermore, the authors should have to assessed the brooding activity rate and colony size throughout the experimental phase, as they are measurable criteria that may be used to determine the significant impact of temperature on the bee. Based on this, the quality of the study is not convincing to me.

We are pleased to resubmit for publication the revised version of the manuscript "**The effect of seasonal temperatures on the aging of the overwintered honey bee**". We would like to thank the reviewers for their careful and thorough reading of this manuscript and for the thoughtful comments and constructive suggestions, which helped to improve the quality of this manuscript.

We look forward to hearing from you.

Sincerely,

Professor Hyung Wook Kwon

Department of Life Science,

Convergence Research Center for Insect Vectors

Incheon National University

119 Academy-ro, Yeonsu-gu, Incheon, 22012, Republic of Korea

+82-32-835-8090 (work);

+82-10-3379-6727 (mobile);

+82-32-835-0764 (fax).

Email: hwkwon@inu.ac.kr
