# Supplementary material for: The effect of seasonal temperatures on the physiology of the overwintered honey bee
Source: PLoS One. 2024 Dec 9;19(12):e0315062. doi: 10.1371/journal.pone.0315062 (PMC11627422; doi:10.1371/journal.pone.0315062)
Supplement: Attachment — Submitted filename: PONE-D-24-13353_R1_reviewer-GWO.pdf [file pone.0315062.s014.pdf]

PLOS ONE

The effect of seasonal temperatures on the aging of the overwintered honey bee

--Manuscript Draft--

**Manuscript Number:**	PONE-D-24-13353R1
**Article Type:**	Research Article
**Full Title:**	The effect of seasonal temperatures on the aging of the overwintered honey bee
**Short Title:**	Aging of honey bees at the end of winter
**Corresponding Author:**	Hyung Wook Kwon<br>Incheon National University<br>Incheon, KOREA, REPUBLIC OF
**Keywords:**	honey bee; aging; vitellogenin; acinus size; molecular markers; climate warming
**Abstract:**	Honey bee physiology follows an annual cycle, with winter bees living ten times longer than summer bees. Their transition can be disrupted by climate change. Several climate factors, mainly temperature, may contribute to global losses of winter bees. We simulated climate warming by maintaining constant temperatures of 25°C (Group 25) and 35°C (Group 35) in rooms around hives from June to October, while a Group control experienced natural conditions. Colony performance was assessed in August and September. In February, workers were examined for physiological traits (acini area and fat body mass) and molecular markers (vg and JHAMT), along with expected markers (ilp1, ilp2, TOR1, and HSP70). Our findings suggest that temperature decreases around winter worker broods from Group 25 in the fall led to their slower physiological aging in winter compared to Group 35 workers, supported by markers. Furthermore, the end of diapause, when aging speeds up, was coupled with upregulation of HSP70, ilp2, and TOR1 genes. This research can predict the winter status of bees in response to fall climate warming and help beekeepers implement strategies to prevent bee losses. Additionally, it could enhance molecular aging identification in insect models, advancing research on climate warming effects.
**Order of Authors:**	Hyung-Wook Kwon
	Olga Frunze
	Yumi Yun
	Hyunjee Kim
	Ravil R. Garafutdinov
	Young-Eun Na
**Response to Reviewers:**	Dear Editor-in-Chief, Reviewer 1 and Reviewer 2!<br><br>Thank you for the time and effort spent reviewing our manuscript number PONE-D-24-13353 (The effect of seasonal temperatures on the aging of the overwintered honey bee) and suggesting some important points to consider.<br><br>We corrected the manuscript according to the recommendations in the Submission Guidelines during the final step of our editing process (the Materials chapter was placed after the Introduction, citations in the text were numbered, and the References were updated). As a result, all figure numbers were changed before resubmission. However, we responded to the reviewer's comments using the previous numbering.<br><br>Our replies are marked by green color after the symbol R (reply) and question numbers for the reviewer’s comments. Also, we copied these modifications in response for convenience when they were available.<br><br>Please, find in the file "Response to Reviewers" the reviewer comments (black) and our response (green)<br><br>We hope you will do the needful by considering the above paper for publication in your esteemed Journal – PLoS ONE.

Powered by Editorial Manager® and ProduXion Manager® from Aries Systems Corporation

<table>
<tr><td></td><td>Sincerely,
Professor Hyung Wook Kwon
College of Life Science and Bioengineering, Division of Life Science
Director of the Convergence Research Center for Insect Vectors
President of the Korean Society for Pestology and Disinfection (KSPD)
Incheon National University, 119 Academy-ro, Yeonsu-gu, Incheon, 22012, Republic of Korea
+82-32-835-0764 (fax); +82-10-3379-6727 (mobile); +82-32-835-8090 (work);
Email: hwkwon@inu.ac.kr</td></tr>
<tr><td>Additional Information:</td><td></td></tr>
<tr><td>Question</td><td>Response</td></tr>
<tr><td>Financial Disclosure

Enter a financial disclosure statement that describes the sources of funding for the work included in this submission. Review the submission guidelines for detailed requirements. View published research articles from PLOS ONE for specific examples.

This statement is required for submission and will appear in the published article if the submission is accepted. Please make sure it is accurate.

Unfunded studies
Enter: The author(s) received no specific funding for this work.

Funded studies
Enter a statement with the following details:
• Initials of the authors who received each award
• Grant numbers awarded to each author
• The full name of each funder
• URL of each funder website
• Did the sponsors or funders play any role in the study design, data collection and analysis, decision to publish, or preparation of the manuscript?
• NO - Include this sentence at the end of your statement: The funders had no role in study design, data collection and analysis, decision to publish, or preparation of the manuscript.
• YES - Specify the role(s) played.

* typeset</td><td>Yes
Hyung Wook Kwon received.
This work was carried out with the support of the Cooperative Research Program for Agriculture Science & Technology Development (Project No. RS-2023-00232749) and the Priority Research Centers Program through the National Research Foundation of Korea (NRF) funded by the Ministry of Education (2020R1A6A1A03041954).</td></tr>
<tr><td>Competing Interests

Use the instructions below to enter a</td><td>The authors have declared that no competing interests exist.</td></tr>
</table>

competing interest statement for this submission. On behalf of all authors, disclose any competing interests that could be perceived to bias this work—acknowledging all financial support and any other relevant financial or non-financial competing interests.<br><br>This statement is required for submission and **will appear in the published article** if the submission is accepted. Please make sure it is accurate and that any funding sources listed in your Funding Information later in the submission form are also declared in your Financial Disclosure statement.<br><br>View published research articles from *PLOS ONE* for specific examples.<br><br>**NO authors have competing interests**<br><br>Enter: *The authors have declared that no competing interests exist.*<br><br>**Authors with competing interests**<br><br>Enter competing interest details beginning with this statement:<br><br>*I have read the journal's policy and the authors of this manuscript have the following competing interests: [insert competing interests here]*<br><br>* typeset	
**Ethics Statement**<br><br>Enter an ethics statement for this submission. This statement is required if the study involved:<br><br>• Human participants<br>• Human specimens or tissue<br>• Vertebrate animals or cephalopods<br>• Vertebrate embryos or tissues<br>• Field research	N/A

Write "N/A" if the submission does not require an ethics statement.<br><br>General guidance is provided below. Consult the submission guidelines for detailed instructions. **Make sure that all information entered here is included in the Methods section of the manuscript.**<br><br>**Format for specific study types**<br><br>**Human Subject Research (involving human participants and/or tissue)**<br>• Give the name of the institutional review board or ethics committee that approved the study<br>• Include the approval number and/or a statement indicating approval of this research<br>• Indicate the form of consent obtained (written/oral) or the reason that consent was not obtained (e.g. the data were analyzed anonymously)<br><br>**Animal Research (involving vertebrate animals, embryos or tissues)**<br>• Provide the name of the Institutional Animal Care and Use Committee (IACUC) or other relevant ethics board that reviewed the study protocol, and indicate whether they approved this research or granted a formal waiver of ethical approval<br>• Include an approval number if one was obtained<br>• If the study involved *non-human primates*, add *additional details* about animal welfare and steps taken to ameliorate suffering<br>• If anesthesia, euthanasia, or any kind of animal sacrifice is part of the study, include briefly which substances and/or methods were applied<br><br>**Field Research**<br><br>Include the following details if this study involves the collection of plant, animal, or other materials from a natural setting:<br>• Field permit number<br>• Name of the institution or relevant body that granted permission	

**Data Availability**<br><br>Authors are required to make all data underlying the findings described fully available, without restriction, and from the time of publication. PLOS allows rare exceptions to address legal and ethical concerns. See the PLOS Data Policy and FAQ for detailed information.<br><br>A Data Availability Statement describing where the data can be found is required at submission. Your answers to this question constitute the Data Availability Statement and **will be published in the article**, if accepted.<br><br>**Important:** Stating 'data available on request from the author' is not sufficient. If your data are only available upon request, select 'No' for the first question and explain your exceptional situation in the text box.<br><br>Do the authors confirm that all data underlying the findings described in their manuscript are fully available without restriction?	Yes - all data are fully available without restriction
**Describe where the data may be found in full sentences. If you are copying our sample text, replace any instances of XXX with the appropriate details.**<br><br>• If the data are **held or will be held in a public repository**, include URLs, accession numbers or DOIs. If this information will only be available after acceptance, indicate this by ticking the box below. For example: *All XXX files are available from the XXX database (accession number(s) XXX, XXX.*).<br>• If the data are all contained **within the manuscript and/or Supporting Information files**, enter the following: *All relevant data are within the manuscript and its Supporting Information files.*<br>• If neither of these applies but you are able to provide **details of access elsewhere**, with or without limitations, please do so. For example:	All relevant data are within the manuscript and its Supporting Information files.

Powered by Editorial Manager® and ProduXion Manager® from Aries Systems Corporation

Data cannot be shared publicly because of [XXX]. Data are available from the XXX Institutional Data Access / Ethics Committee (contact via XXX) for researchers who meet the criteria for access to confidential data.<br><br>*The data underlying the results presented in the study are available from (include the name of the third party and contact information or URL).*<br>• This text is appropriate if the data are owned by a third party and authors do not have permission to share the data.<br><br>* typeset	
Additional data availability information:	

Powered by Editorial Manager® and ProduXion Manager® from Aries Systems Corporation

Click here to access/download;Manuscript;Manuscript.docx

Research article

The effect of seasonal temperatures on the aging of the overwintered honey bee

Olga Frunze[1¶], Yumi Yun[1¶], Hyunjee Kim[1], Ravil R. Garafutdinov[2], Young-Eun Na[3]*, and Hyung-Wook Kwon[1]*

[1]Department of Life Sciences & Convergence Research Center for Insect Vectors (CRCIV), Incheon National University, 119 Academy-ro, Yeonsu-gu, Incheon, Republic of Korea 22012;

[2]Institute of Biochemistry and Genetics, Ufa Federal Research Center, Russian Academy of Sciences, 450054, prosp. Oktyabrya, 71, Ufa, Bashkortostan, Russian Federation

[3]Agro-materials Industry Division, Rural Development Administration (RDA), Wanju 55365, Republic of Korea

[¶]These authors contributed equally to this work

*Correspondence: hwkwon@inu.ac.kr (H.-W.K.) youngman99@korea.kr (Y.-E.N.)

Short title: **Aging of honey bees at the end of winter**

Abstract

Honey bee physiology follows an annual cycle, with winter bees living ten times longer than summer bees. Their transition can be disrupted by climate change. Several climate factors, mainly temperature, may contribute to global losses of winter bees. We simulated climate warming by maintaining constant temperatures of 25°C (Group 25) and 35°C (Group 35) in rooms around hives from June to October, while a Group control experienced natural conditions. Colony performance was assessed in August and September. In February, workers were examined for physiological traits (acini area and fat body mass) and molecular markers (*vg* and *JHAMT*), along with expected markers (*ilp1, ilp2, TOR1*, and *HSP70*). Our findings suggest that temperature decreases around winter worker broods from Group 25 in the fall led to their slower physiological aging in winter compared to Group 35 workers, supported by markers. Furthermore, the end of diapause, when aging speeds up, was coupled with upregulation of *HSP70, ilp2,* and *TOR1* genes. This research can predict the winter status of bees in response to fall climate warming and help beekeepers implement strategies to prevent bee losses. Additionally, it could enhance molecular aging identification in insect models, advancing research on climate warming effects.

Introduction

Honey bees play a crucial role in human life as pollinators on agricultural farms, producers of honey, royal jelly, and other products, which are valued for their health and medicinal benefits, and contributors to ecosystems worldwide. However, the loss of bees has been reported over the last two decades, particularly at the end of the overwintering period [1-3].

Several factors arising during the year can contribute to this [4]. For example, climate warming or cooling [5] impacts the lifespan in flies [6], fish [7], honey bees [8], and rodents [9]. Other factors related to climate change include the quality of forage [10, 11], beekeeper management practices [12, 13], and pesticide exposure from land use [11]. Although we can try to avoid some of these factors, effective methods to mitigate the impacts of climate change and prevent honey bee winter mortality are currently lacking.

To advance in this direction, it is necessary to clarify the general processes occurring in late summer and fall that are involved in the transition from short-lived to long-lived workers [14, 15], as these processes may be disrupted by climate change. This transition has been associated with external (environmental) factors that influence internal (metabolic) processes.

Known external trigger factors include a decrease in pollen availability [16, 17] and cessation of brood rearing [15, 18]. Ambient temperature, as factor of climate change,

can significantly alter brood temperature in summer [19], despite honey bees efforts to maintain a consistent brood microclimate [20-22].

With the onset of fall, external trigger factors initiate the transition of bees through internal processes, leading to the development of winter workers with prolonged longevity, which can last up to 8 months [1]. Briefly, this transition exhibits the classic insect diapause phenotype, characterized by reduced metabolic activity and altered hormonal profiles [14, 23-26]. Unlike other insects, the reduced metabolic activity of honey bees during diapause [17] includes vibrating their wing muscles to generate heat and maintain warmth within the winter cluster. This process depends on a constant energy source and requires a pure carbohydrate diet [27], which is not necessary for other diapausing insects and impacts internal processes.

In winter bees, the physiology of internal processes related to this hormone-regulated phenomenon is well-described. It manifests as hypertrophied secretory vesicles and increased acini diameter in the hypopharyngeal gland (HG) compared to newly emerged and forager summer bees [28]. HG development is known to be positively correlated with vitellogenin (*vg*) throughout a worker's lifespan. However, *vg* is synthesized in the fat body [29], which is more developed in winter bees compared to summer bees [27]. The fat body mass results from the accumulation of nutrients [30], which lipids constituting over >90 % of the mass in the form of triglycerides. These triglycerides are synthesized from dietary carbohydrates and fatty acids and are used not only for *vg* synthesis [30] but also for producing pheromones,

phospholipids, and wax [31].

Significant differences between honey bees and other insects have been observed in the mutually inhibitory interaction between vg (protein) and the juvenile hormone (JH) [32]. An increased level of vg (protein and gene), alongside decreased levels of the JH [1, 15, 33], has been noted, however, the molecular mechanism of this complex relationship remains unclear [34]. It is known that the JH biosynthetic pathway begins in the glands in the brain complex and responds to sensory inputs that underpin the division of labor [34, 35]. Furthermore, at the end of winter, diapause bees gradually transition to nursing brood care and foraging physiology [1]. The last transition is characterized by several physiological and molecular changes, including a decrease in acini area [36] and fat body mass [37], as well as reductions in vg levels and increases in JH levels [15, 38]. These changes contribute to seasonal effects and behavior [39], leading to reduced longevity and increased aging compared to diapause conditions [40, 41].

Alongside with the well-known age-related marker gene *vg* [32, 42-44], this study examined other potential markers, including the genes *JHAMT, ilp1, ilp2, TOR1*, and *HSP70*. The enzyme involved in JH biosynthesis, *Juvenile Hormone Acid Methyltransferase (JHAMT),* exhibits changes in expression similar to JH titer and can serve as an alternative marker to JH [45-47]. A positive correlation with JH was observed in the *insulin-like peptide, ilp1,* whose expression increases in the brains of foragers and decreases with continued foraging [48]. The *insulin-like peptide, ilp2*

gene is expected to have task-related expression as it is highly expressed in nurse tissue and plays a role in increasing cell size and number in individual organs [49]. Additionally, the *TOR1* gene, a nutrient-sensing kinase, along with the *ilp1* and *ilp2* genes, participates in the mobilization of nutritional resources (autophagy) from tissues such as the fat body and glands in *Drosophila* [50]. Moreover, the *TOR* pathway is implicated in disease and nutrition in insects [51-53], and mammals [54], and it also regulates longevity extension in social insects [25]. Lastly, the stress marker *heat shock protein 70 (HSP70)* was chosen because it is detected at low levels in nurses but shows increased expression in foragers [55]. While all these markers are expected to be important for identifying aging in honey bees, testing them together requires further investigation.

This research aimed to simulate the influence of climate warming on colony performance in early fall and to compare the aging of overwintered *Apis mellifera ligustica* honey bees. The experiment involved maintaining colonies at ambient temperatures of 25°C (Group 25) and 35°C (Group 35) in controlled rooms, representing the average and high summer temperatures in Incheon (Republic of Korea), respectively. A Group control was kept under natural conditions. The temperature experiments continued throughout the last summer, September, and October, with the colonies overwintering under the same conditions. Colony performance, including the brood microclimate, was assessed in August and September, during the transition from summer to winter physiology in the Republic of

Korea. In February, we evaluated the aging of the bees at the end of diapause (broodless period) using eight markers: two physiological markers and six molecular gene markers. The physiological markers were acini area and mass of lipid content in the fat body. The molecular markers were for: tasks (*vg* and *JHAMT*), nutrition (*ilp1, ilp2*, and *TOR1*), and stress response (*HSP70*). By linking climate change factors, such as fall temperature, to honey bee responses in winter, this research can inform conservation actions for beekeepers to prevent bee losses. We believe this study can lead to further research and the development of a test kit to identify the causes of aging early.

Methods

Experimental bees

Western honey bees (*Apis mellifera ligustica*) were obtained from nine colonies kept at the Incheon National University apiary in the Republic of Korea **(Fig 1A, B, C, and D),** managed by a professional beekeeper following standard beekeeping practices [56]. The experimental colonies housed first-year young sister queens, naturally mated and beginning egg-laying in May 2022. Prior to the experiment in June, colonies were standardized in terms of the number of frames (total 4), brood population (3 frames), honey amount (1 frame), and the number of honey bees. Colony strength was assessed through visual inspection of adult and brood populations both before and during the experiment [56]. Two rooms equipped with a climate-control system were established

to maintain honey bee colonies at 25°C (Group 25) and 35°C (Group 35), simulating climate warming without day and night temperature differences.

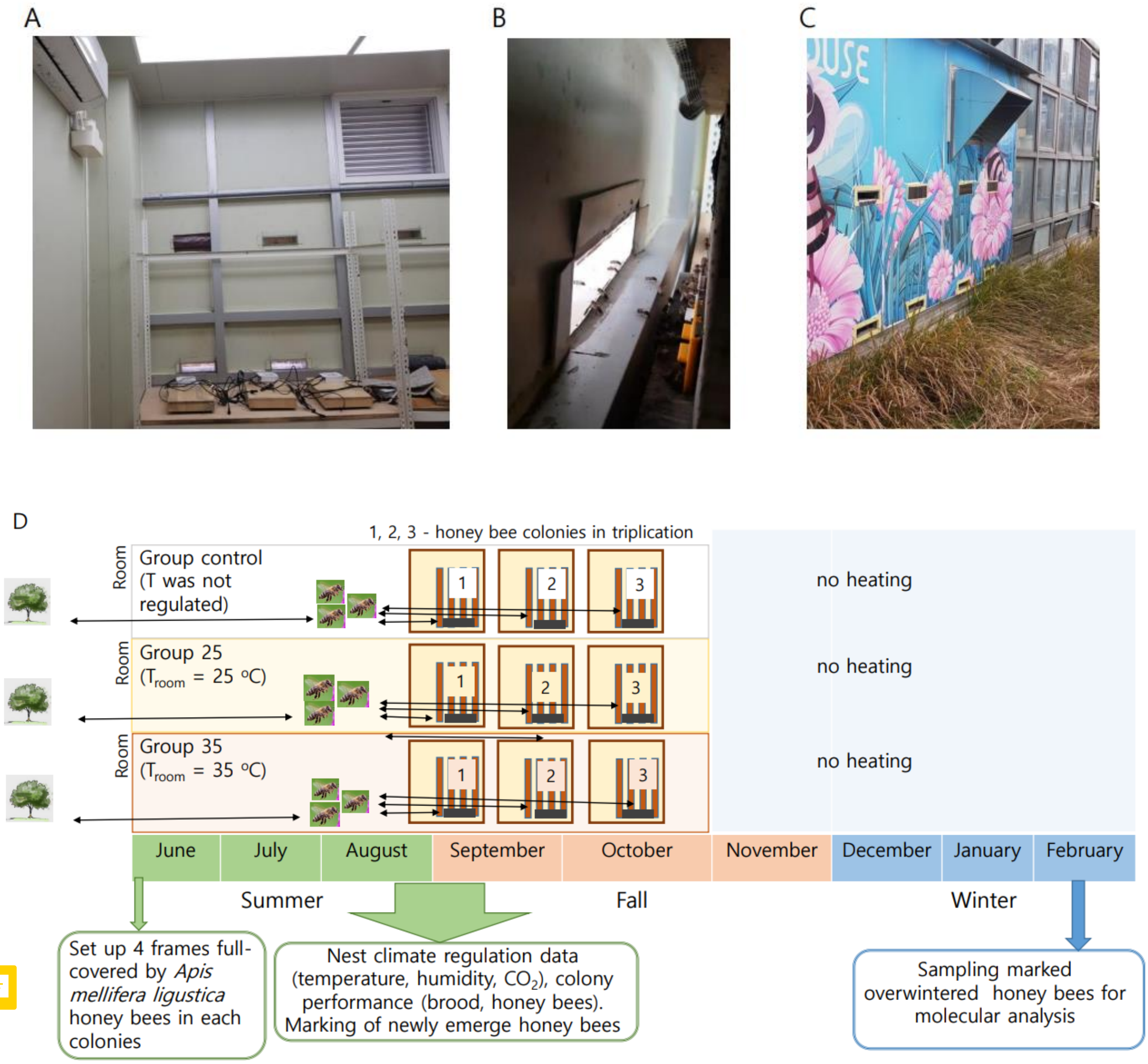

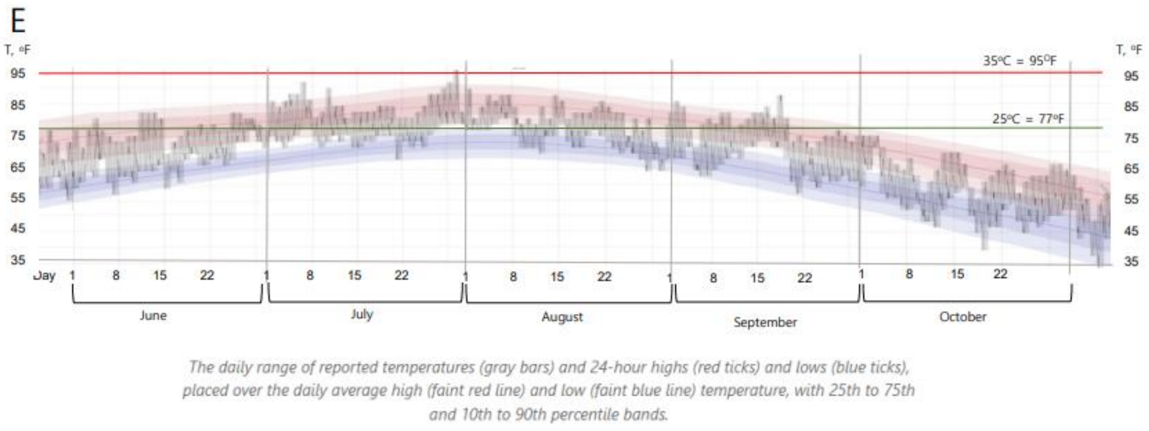

Fig 1. Overview of the experimental design (A) The room environment before the experiment. **(B)** The process of honey bees freely flying outside from the experimental room. **(C)** The view of the room and the entrance for honey bees outside. **(D)** Timeline of the experiment. **(E)** Ambient temperature of the Group control, Group 25 (green line), and Group 35 (red line) under the temperature experiment in 2022, Incheon, Republic of Korea. The data were obtained from https://weatherspark.com/h/m/142040/2022/10/Historical-Weather-in-October-2022-in-Incheon-South-Korea#Figures-Temperature.

These temperatures were selected to mimic the effects of climate warming, with 25°C representing the summer average and 35°C being the highest recorded in Incheon city under typical conditions. However, the highest temperature was not naturally reached during the summer of 2022 **(Fig 1E).** Other environmental conditions were not controlled. The light was used during colony inspections. In the third room, housing honey bee colonies, the control group was provided with free ventilation without additional heating. All honey bees had equal opportunities to collect pollen and nectar around the apiary.

On September 18th and 25th, 2022, two combs with sealed brood were placed in an

incubator set at 34°C. Upon hatching, the workers were individually marked with colored paint on their thoraxes and then reintroduced into their original colony. Throughout the previous summer, as well as in September and October of 2022, the honey bee colonies were subjected to varying room ambient temperatures. Afterward, the controlled temperatures in the rooms were turned off, and the bee colonies were overwintered successfully (**S1 Table**) in the same rooms under natural conditions.

The overwintered marked honey bees, aged approximately 4.5 months, were sampled on February 5th, 2023, a time when queens had not yet begun laying eggs and the overwintered bees were expected to have successfully survived the winter. Forty honey bees from each colony were sampled and individually placed in labeled 50 ml tubes, then stored at -80°C before analysis. The success of colonies overwintering was monitored on February 5th and 27th, when we recorded whether the honey bees survived (+) or did not survive (-) (**S1 Table).**

Brood microclimate measurements

The temperature, humidity (AM2320, BeeOnFarm, Korea), and CO_2 concentration (BO-100, BeeOnFarm, Korea) sensor systems were placed on the top and middle of each colony in August 2022. The data logging system was included in the sensor system because it provided a real-time clock. Data consisting of time (seconds), CO_2 (in ppm), temperature (in °C), and relative humidity (as a percentage) were saved, with approximately three measurements in minutes to approximately one file per day

(S1 File). The data from 6 honey bee colonies kept in each room (three at 25°C and three at 35°C) were collected over 15 days from 11 to 25 August. Average values were used for statistical calculations.

Hypopharyngeal gland (HG) measurements

Areas of ninety acini from the hypopharyngeal glands of overwintered honey bees in each Group (control, 25°C, and 35°C) were measured to determine if there were differences based on the ambient temperature during the previous fall season. Thirty bees were collected from each colony, flash frozen in liquid nitrogen, and maintained at −80°C until their glands were measured **(S2 File)**. For each bee, the HGs were dissected into 1× phosphate-buffered saline (PBS) with 0.1% Tween® 20 and visualized at 40× magnification. The area (mm^2) of a minimum of ten randomly selected acini per bee was measured using Nikon Eclipse E200 (Nikon, Tokyo, Japan) Toupview (Touptek, Hangzhou, China) software. Only acini with clear borders were used. Acini areas were averaged within individuals.

Fat body lipid content

The fat body lipid content in the abdomen of overwintered honey bees was studied in early February by the standard method [57, 58]. Ten honey bees were collected from each colony; ninety honey bees were flash frozen in liquid nitrogen and maintained at −80°C until their abdomens were dissected and their guts were removed. Pools of ten abdomens were weighed on a scale (HS220S, HANSANG Instrument Co., Ltd.,

Republic of Korea) before and after drying to a constant weight in a fume hood (SSFH-1000, Shinsaeng, Republic of Korea). The total lipid content was determined by measuring the decrease in weight following the removal of lipids from the dried tissue through extraction in 5 ml portions of diethyl ether (179272-1 L; Sigma–Aldrich, USA) **(S2 File).**

RNA Extraction and cDNA Synthesis

A total of ninety honey bee (ten winter bees from each colony) *Apis mellifera ligustica* colonies were sampled in labeled tubes and stored at -80°C. Three honey bees from each colony were analyzed. The brain and gut-free abdomen of honey bees were dissected on ice, and RNA was extracted directly. RNA was extracted from the brain for the analysis of *HSP70, JHAMT, ilp1, ilp2*, and *TOR1* expression, and from the abdomen for *vg* gene expression analysis, as this gene is expressed in the fat body located in the abdomen. Genes are measured in the brain to understand their roles in stress responses (*HSP70*), metabolic regulation (*JHAMT* and *TOR*), and neural function (*ilp1* and *ilp2*).

Total tissue RNA (brain or abdomen) was extracted from fifteen randomly selected bees in each group using a Qiagen RNeasy Mini Kit (#74,104; Qiagen, Valencia, CA, USA). The tissues in each replicate were brain tissue for *TOR1, ilp1, ilp2, JHAMT*, and *HSP70* and abdomen for *vg* gene expression. The total RNA concentration and purity were quantified using OD260/OD280 values between 1.8 and 2.0. Next, reverse

transcription was performed using an RNA to cDNA EcoDryTM Premix (Oligo dT) kit (Takara, Japan). The reverse transcription reaction mixture included 50 ng/µl total RNA (with the clear volume calculated for each sample) and RNase-free water for a total volume of 20 µl. Reverse transcription was conducted at 42°C for 60 min, followed by heating at 70°C for 10 min.

Quantitative real-time PCR

The relative expression of six genes related to nutrition (*vg, ilp1, ilp2, TOR1),* development *(JHAMT),* and stress response (*HSP70)* was measured. The housekeeping gene *RpL32* (Ribosomal protein L32*)* was used as an endogenous control. The PCR primer sequences are shown in **S2 Table.**

The reaction conditions were optimized. The qRT–PCR mixture had a volume of 20 µl which included 2 µl of template cDNA, 10 µl of Green qPCR Master Mix, 1 µl of upstream and downstream primers (5 pM/µl), and 6 µl of nuclease-free water (AM9930, Invitrogen, USA). Quantitative real-time PCR (qRT–PCR) was conducted using Brilliant III Ultra-Fast SYBR® Green qPCR Master Mix (600882, Agilent Technologies, USA) on an AriaMx Real-Time PCR System (Agilent Technologies LDA, Malaysia) with AriaMx 96 Well Plates, Skirted, LP (401490, Agilent Technologies, USA) covered by MicroAmp™ Optical Adhesive Film (4311971, Thermo Fisher Scientific, USA).

The qRT–PCR amplification procedure was as follows: initial denaturation at 95°C

for 10 minutes; 40 cycles of denaturation at 95°C for 30 seconds, annealing at 60°C for 25 seconds, and extension at 72°C for 15 seconds. Each sample was technically replicated three times. Data analysis was performed using Agilent AriaMx version 2.0 analysis software. Relative gene expression data were analyzed using the 2^(-Delta Delta C(T)) method [59, 60] (**S3 File**).

Confirmation of amplicons in the RT–PCR products was performed by separating them through electrophoresis in a 2% agarose gel at 80 V for 40 minutes and analyzing them using a gel documentation system, the Gerix 1010 transilluminator (Biostep GmbH, USA) (**S2 Fig**). A Dyne 50 bp DNA Ladder (Cat. No. A701, DYNEBIO, Korea) was used as a reference.

Statistical analysis

The statistical analysis, designed as illustrated in **Fig 2**, was conducted using Microsoft Excel and XLSTAT software (Addinsoft Pearson Edition 2014, Addinsoft, Paris, France). The characterization was performed using the following strategies [61]: (a) classification of the marker dataset to find a combination of features that best separates different groups (supervised approach, Discriminant Analysis (DA)); (b) clustering to discover natural groupings in the absence of labeled information (unsupervised approach, Agglomerative Hierarchical Clustering (AHC)); and (c) modeling the relationship between independent variables and a dependent variable and dealing with multicollinearity in the marker dataset to rank the markers by importance

for identifying overwintered honey bee task development (supervised approach, Elastic Net Regression).

Specifically, the honey bees were categorized into groups, and the variables with the highest discrimination scores were selected using DA because it determines the linear combination of variables and provides maximal separation between groups. To discriminate the groups, Fisher distances were applied, which emphasize the linear combinations of variables that maximize the differences between groups.

AHC analysis provided a visual representation of the clustered dataset, revealing the arrangement of individual data points or observations based on their dissimilarity. The evaluation metric for clustering algorithms was the silhouette index. The score is bounded between (− 1) for incorrect clustering and (+ 1) for highly dense clustering.

			Apis mellifera ligustica honey bees	
Month of sampling		Group control<br>(3 colonies)	Group 25<br>(3 colonies)	Group 35<br>(3 colonies)
Experimental condition<br>(summer, September, and October)			T_{room} = 25 °C	T_{room} = 35 °C
**August**<br>**September**	Data set 1<br>(brood microclimate and colony development)		Nest climate regulation	
	Statistics of data set 1		T-test ($p < 0.05$)	
**February**	Sampling of honey bees, n	(for physiology markers): 30+30+30<br>(for molecular markers): 10+10+10	30+30+30<br>10+10+10	30+30+30<br>10+10+10
	Data set 2	Two physiology markers (hypopharengial glands (HG, acini area) and fat body mass)<br>Molecular marker genes related to nutrition (*vg*, *ilp1*, *ilp2*, *TOR1*), development (*JHAMT*), and stress response (*HSP70*).		
	Statistics of data set 2	1. Discriminant analysis (define the variables with high discrimination score);<br>2. Agglomerative Hierarchical Clustering (check the natural groupings within dataset);<br>3. Elastic Net Regression (model the relationship between HG (acini area) and fat body mass with molecular markers);<br>4. ANOVA, Duncan's post-hoc test (comparison of significance within each variable, $p < 0.05$).		

Fig 2. Experimental conditions, sampling schedule, data sets, and statistical tests used for analysis.

Elastic Net Regression analysis was used to test two models of the relationship [61,

62] between physiology and molecular markers. The dependent variables were acini size and fat body mass. The seven independent variables were ranked by coefficient. Receiver operating characteristic (ROC) curves were generated to quantify how accurately regression analysis can discriminate between honey bee groups [63]. The ROC specificity and sensitivity plot demonstrated a perfectly fitted model linking physiology to molecular markers (gene expression profile) (**S1 Fig**). The area under the curve (AUC) can range from 0.5 to 1.0, with a preference for higher scores.

The mean, standard deviation, and variance of each gene were calculated using descriptive statistics and visualized using the heat map module. For the gene expression analysis, ANOVA was used to test overall effects, followed by the Duncan's post hoc test ($p < 0.05$) for multiple comparisons and the t-test for differences of means between two groups of honey bees (**S4 File**). The bar plots were generated using the online SRplot Official Website (https://www.bioinformatics.com.cn/en; accessed on 30 October 2023).

Results

The experiment was conducted on nine colonies of western honey bees (*Apis mellifera ligustica*). Each Group (control, 25°C, and 35°C) was represented by three colonies. The bees from the Groups 25 and 35 experienced stable, controlled day and night ambient temperatures (modeling climate warming) from June to October, while the room for the Group control was freely ventilated. All bees experienced natural

foraging outside without restrictions. Newly emerged honey bees were marked in September for the overwintering experiment in February.

Dynamics of ambient temperature in summer and fall months

The daily temperature in the first experimental room was set at 25°C for five months, and there were no significant differences compared to the natural daily average temperature in the summer months or September (ANOVA, Duncan's post hoc tests, $p > 0.05$). The difference in experimental conditions for the honey bees from the Group control and the Group 25 was due to day-night temperature fluctuations, which were absent in the latter Group. Although significant temperature differences were not detected, a stable elevated night temperature is one of the factors of climate warming that influences bees. However, it was significantly greater than the average daily temperature in October 2022 (same tests, $p < 0.05$). The daily temperature in the second room was set at 35°C for five months, which was significantly greater than the average daily temperature during the summer months, September, and October (same tests, $p < 0.05$).

Brood microclimate and colony development in August and September

In this study, we evaluated the performance of the colony using temperature, humidity, CO_2, number of larvae, capped brood area, and honey bees' measurements from August 08 to September 18, 2022, when short-lived bees commenced rearing larvae

that would develop into long-lived bees for the overwintering period. Since the expected ambient temperature in August and early September and the comparison of the colony performance of the Groups control and 25 on August 08 (**Fig 3 D, E, F**) showed no significant differences, sensors were not set up in the Group control. We hypothesized that the regulation of the nest climate inside colonies influences the physiology of winter bees and the success of overwintering.

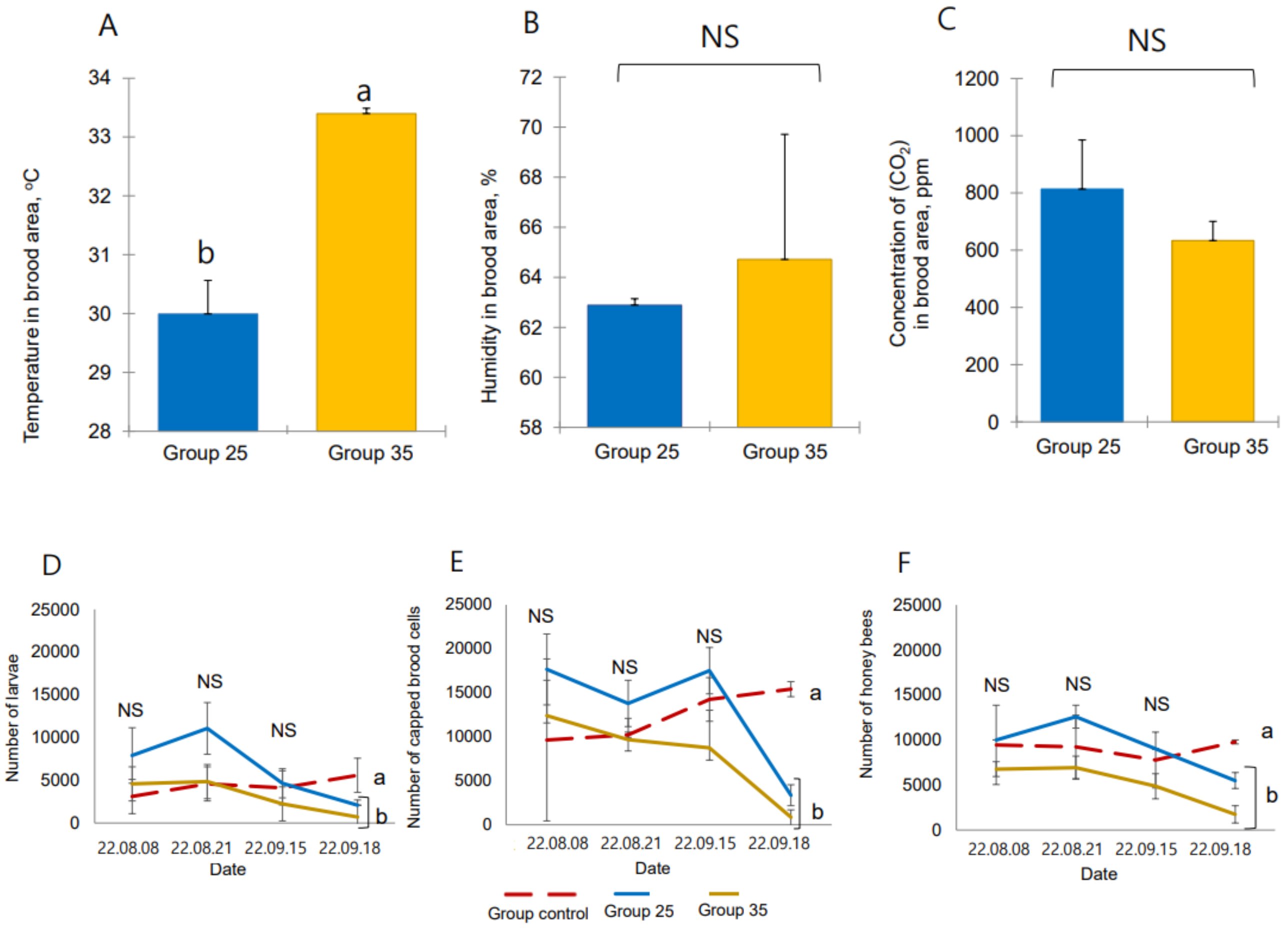

Fig 3. Colony performance in honey bees treated under different ambient temperatures (08 August – 18 September 2022). **(A)** Temperature in the brood area (°C). **(B)** Humidity in the brood area (%). **(C)** Concentration of CO_2 in the brood area (ppm). **(D)** Number of larvae. **(E)** Number of

capped brood cells. **(F)** Number of honey bees. Statistical significance was determined using a t-test in **Fig 3A, B**, and **C** (*p* < 0.05, indicated by different letters above the columns) to compare the means of the two groups, one-way ANOVA, Duncan's post hoc tests (*p* < 0.05, indicated by different letters above the columns) to compare the means of the three groups in **Fig 3D, E**, and **F**; bars in the graphs represent the mean ± SD.

SD – Standard Deviation, NS – No Significance. Honey bee colonies in Group 25 were maintained in room conditions at 25°C; colonies in Group 35 were kept in room conditions at 35°C, while colonies from Group control were maintained under natural conditions.

The mean and standard deviation of the brood temperature for bee colonies kept at 25°C and 35°C were 30.00 ± 0.982 and 33.40 ± 0.154 °C, respectively. Similarly, the nest humidities for these groups were 60.00 ± 0.435 and 64.72 ± 8.650 %, and the nest CO_2 concentrations were 813.55 ± 296.8 and 633.30 ± 117.0 ppm, respectively. Importantly, the nest temperature inside the colonies kept at 35°C was significantly greater than that inside the colonies kept at 25°C (t-test, $p < 0.05$) (**Fig 3A**). However, nest humidity, CO_2 concentration, number of larvae, capped brood cells, and bees were not significantly differing **(Fig 3B, C**: t-test, $p > 0.05$) (**Fig 3D, E, and F:** ANOVA, Duncan's post hoc tests $p > 0.05$). The colonies in the Group control, however, exhibited significantly (ANOVA, Duncan's post hoc tests, $p < 0.05$) larger capped brood area, more larvae, and a higher number of bees on September 18 compared to colonies in Groups 25 and 35.

Analysis of the honey bee groups and the relationship between the

markers

The relationship between molecular markers and the biological age of overwintered honey bees was analyzed using a dataset comprising two established physiological markers (acini area and mass of lipid content in the fat body) and six molecular markers (*vg, TOR1, JHAMT*, *ilp1, ilp2,* and *HSP70*). Data Mining methods, including Discriminant Analysis (DA), Agglomerative Hierarchical Clustering (AHC), and Elastic Net Regression, were employed in the analysis.

DA was performed to plot the Groups control, 25 and 35, and to identify the optimal combination of features that effectively distinguishes them. The Wilcoxon Lambda test within the DA yielded a statistically significant result (two-tailed test, $p < 0.05$), indicating that the model successfully differentiated between groups (**Fig 4A**). The observed Wilks' lambda value of 0.000 signifies a robust separation of the Groups control, 25, and 35, demonstrating a high level of discriminative power. A comparison revealed significant differences between Group 25 and Group 35 ($F(2,8) = 19.37$; two-tailed test, $p < 0.05$; **Fig 4A**).

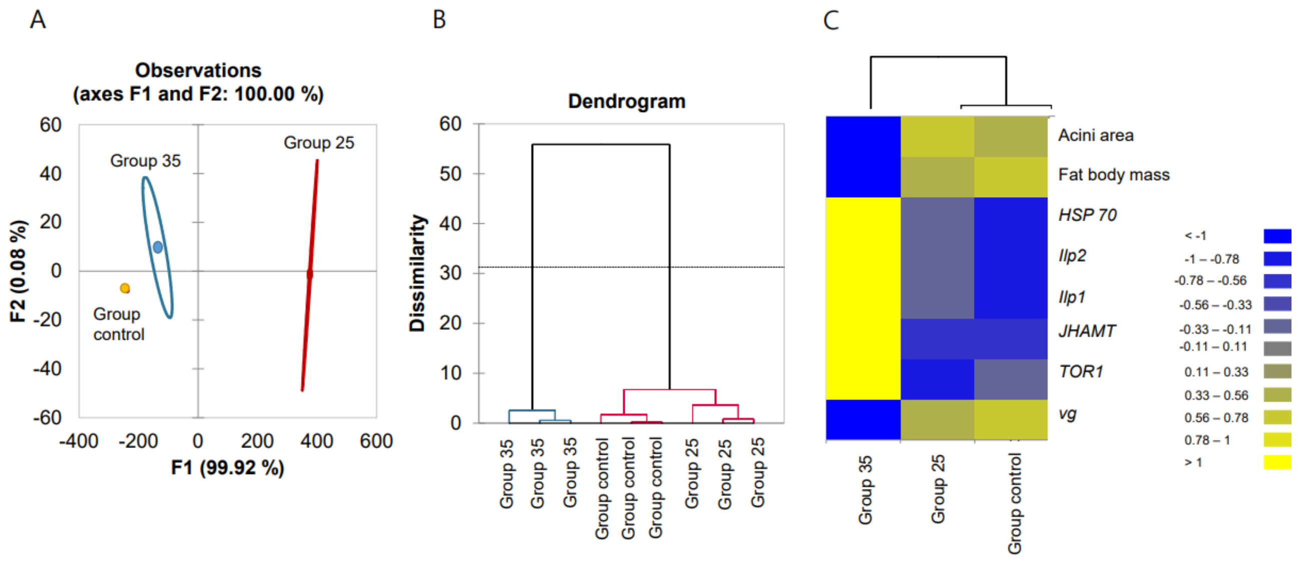

Fig 4. The discrimination of overwintered honey bees was performed using eight variables related to aging: two physiological variables (acini area, n = 30; mass of lipid content in the fat body, n = 10) and six molecular variables (n = 10), including the expression levels of *HSP70, ilp1, ilp2, JHAMT, TOR1,* and *vg* genes. (A) Discriminant Analysis (DA). **(B)** Agglomerative Hierarchical Clustering (AHC). **(C)** Heatmap with cluster analysis based on ddCt values, acini area (mm^2), and the fat body mass (mg).

The Fisher distances (FDs) in the DA between the Groups control, 25, and 35 were significantly different (FD = 27511.10; $p < 0.05$ and FD = 19131.16; $p < 0.05$, respectively). Moreover, the FD density was also significantly different between the Group control and Group 25 (FD = 783.659; $p < 0.05$). The honey bees were separated along the primary discriminant axis F1 in 99.92% accuracy, where discriminant function coefficients for gene expressions *vg, HSP70*, and *JHAMT* were -20.092, -14.960, and 23.576, respectively. These coefficients were the highest, providing significant contributoins to this separation.

Subsequently, AHC was employed to identify natural groupings without labeled information using a dissimilarity measure. The evaluation metric for clustering algorithms, the Silhouette index between clusters 1 and 2, was 0.657, indicating successful clustering, albeit with moderate density **(Fig 4B, C).**

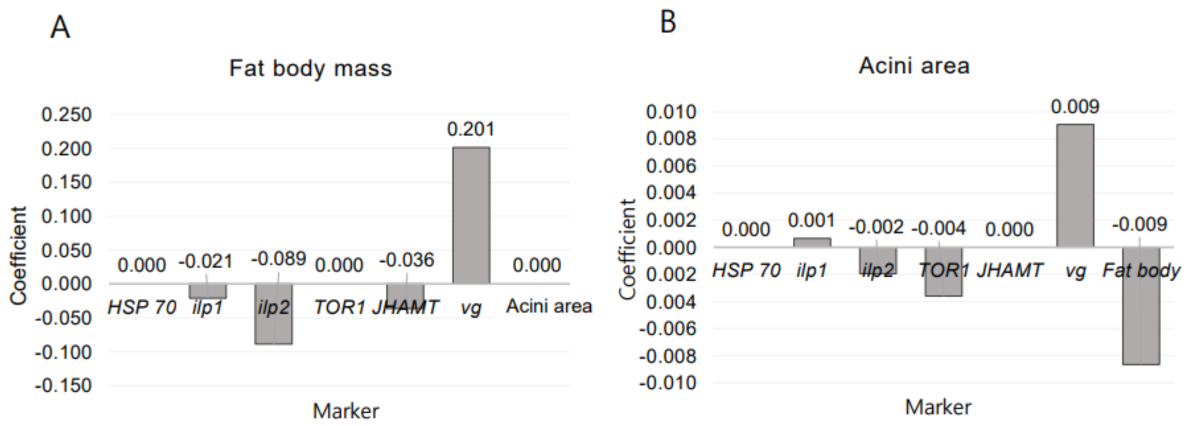

Fig 5. Elastic Net Regression modeling the relationship beween dependent variables fat body mass (mass of lipid content in the fat body, A) and acini area (B) and the independent variables (*HSP70, ilp1, ilp2, TOR1, JHAMT, vg,* and acini area or fat body mass respectively) to rank the variables by importance for predicting dependent variables.

Finally, Elastic Net Regression was used to rank the markers by importance by modeling the relationships between seven independent variables and one dependent variable **(Fig 5A, B).** This analysis was conducted separately for fat body mass **(Fig 5A)** and acini area **(Fig 5B).** The fat body mass model identified significant predictors, with variable *vg* having the highest positive coefficient (0.201) and variable *ilp2* having the highest negative coefficient (-0.089). In the acini area model, significant predictors were identified, with the variable *vg* having the highest positive coefficient (0.009) and the variable fat body mass having the highest negative coefficient (-

0.009). In this both models, only two genes — *vg* and *ilp2* — showed high positive and negative coefficient values, respectively.

Comparative analysis of the biological age of overwintered western honey bees through physiology markers

These are the results based on the two physiological markers, analysis of the mass of lipid content in the fat body (fat body mass) and the acini area from the hypopharyngeal glands (HGs). The mean and standard deviation of the fat body mass for the bees in the Groups control, 25, and 35 were 4.33 ± 1.247, 4.00 ± 1.633, and 2.00 ± 0.816 mg/bee, respectively, with no significant differences (ANOVA, Duncan's post hoc, P = 0.222, $p < 0.05$), even though the fat body mass of the them in Group 35 was slightly lower **(Fig 6A).**

Similarly, the mean and standard deviation of the area of the bee acini from the Groups control, 25, and 35 were 0.026 ± 0.001, 0.027 ± 0.003, and 0.018 ± 0.001 mm^2, respectively. Notably, the acini area in Group 35 was significantly lower (same test, P = 0.006, $p < 0.05$) than that in the Group control and 25 **(Fig 6B).** Importantly, both the fat body mass and the acini area of the overwintered bees in Group 35 were lower than those in Groups control and 25 **(Fig 6)**, suggesting lower nutrition and accelerated physiological aging in bees from Group 35.

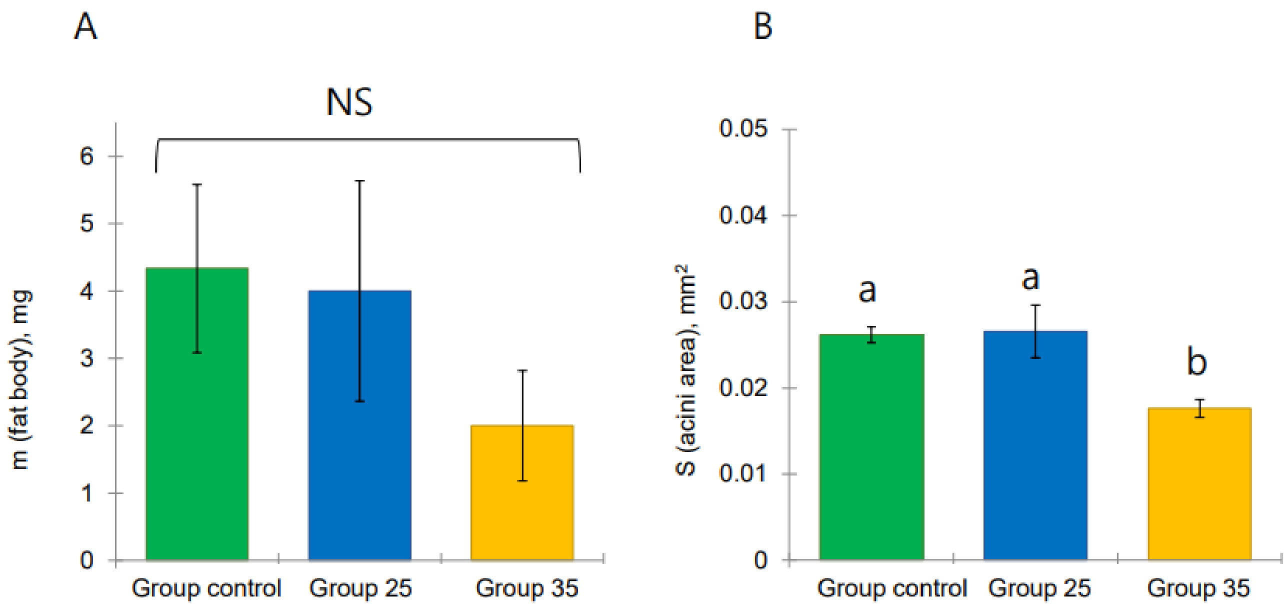

Fig 6. Physiological markers of long-lived honey bees at the end of winter (February) (A) The average of fat body mass (mass of lipid content in the fat body), n = 10 (mean ± SD). **(B)** Average acini area, n = 30 (mean ± SD). Statistical significance was determined using one-way ANOVA, Duncan's post hoc tests ($p < 0.05$, indicated by different letters above the columns).

Comparative analysis of the biological age of overwintered western honey bees through molecular markers

These are the results based on molecular markers. They include two previously defined task-related markers, *vg* and *JHAMT,* as well as four genes expected to respond to aging: *TOR1, ilp1, ilp2*, and *HSP70* **(Fig. 7).**

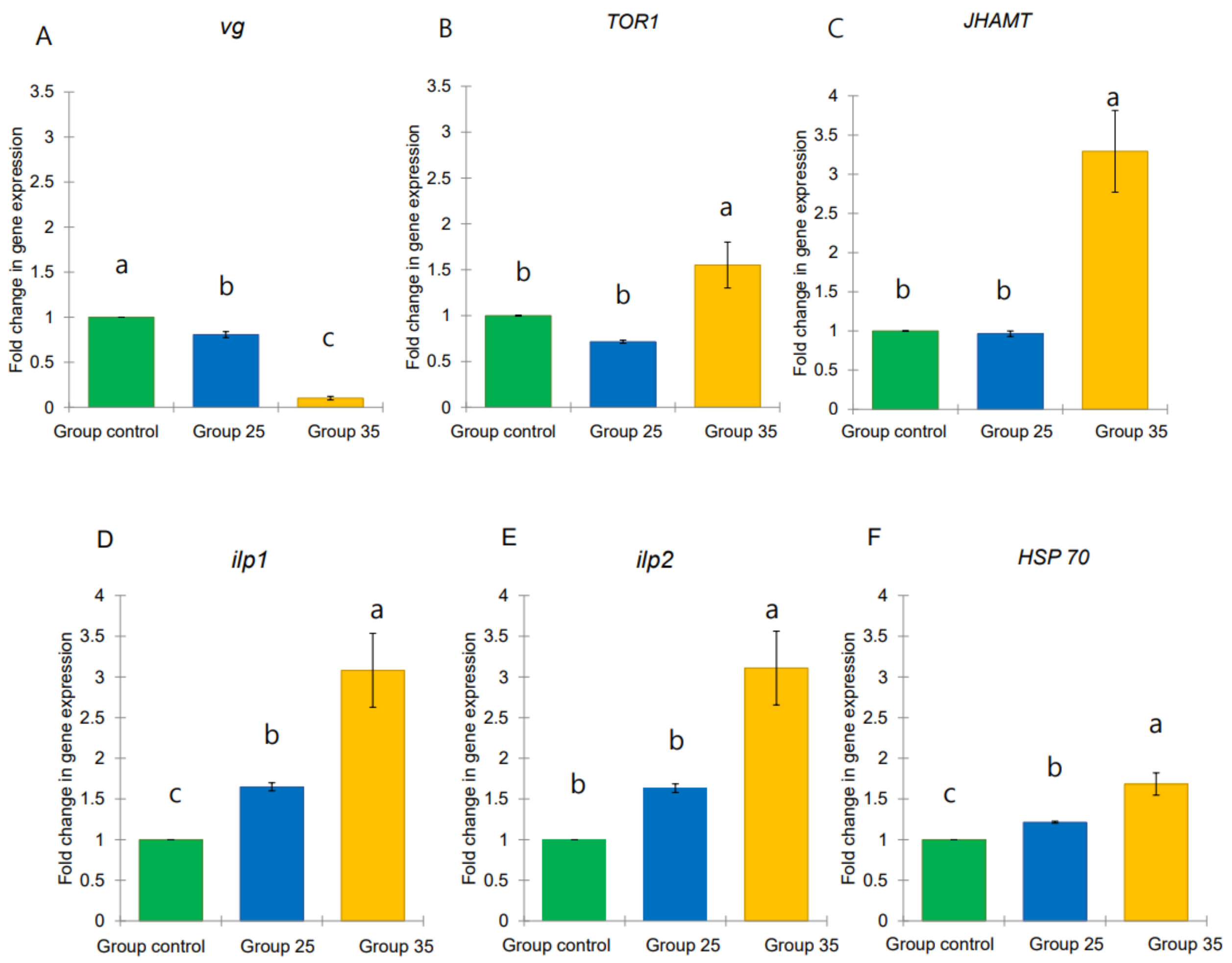

Fig 7. Gene expression analysis. The gene expression (ddCt) of *vg** **(A),** *TOR1*** **(B),** *JHAMT*** **(C),** *ilp1*** **(D),** *ilp2*** **(E),** and *HSP70*** **(F)** in long-lived honey bees at the end of overwintering (February) was evaluated. Statistical significance was determined using one-way ANOVA, Duncan's post hoc tests ($p < 0.05$, indicated by different lowercase letters above the columns). The bars in the graphs represent the mean ± SD. *n=9. **n=15.

Expression of *vg* gene was significantly upregulated (ANOVA, Duncan's post-hoc test, $p < 0.05$) in bees from Group control and 25 compared to those from Group 35. In contrast, the expression of the *TOR1, JHAMT, ilp1, ilp2*, and *HSP70* genes was significantly downregulated (same test, $p < 0.05$) in the same bees, suggesting aging in

bees from Group 35 compared to Groups control and 25 **(Fig. 7B, C, D, E, and F)**. However, there were no significant differences in the expression of *TOR1, JHAMT*, and *ilp2* (same test, $p > 0.05$) between bees from Group control and Group 25. The expression levels of *ilp1* and *HSP70* were significantly lower (same test, $p < 0.05$) in the ones from Group control than in Group 25.

Discussion

Key triggers influencing the transition of insects from summer to winter physiology include photoperiod, ambient temperature, brood microclimate, availability of pollen, and some yet-to-be-identified factors [25, 64, 65], which obviously have a particular influence on aging and subsequent winter loss in honey bees. However, due to the complex and unclear interactions among all these triggers, we designed an experiment in which only one trigger of climate warming, ambient temperature (25, 35°C, and control), was manipulated during the previous summer, September, and October. Colony development and nest climate regulation were investigated in August and September, during which long-lived (winter) bees were reared and marked to study the biological age of honey bees alongside their clear chronological age in February. Aging was assessed using two physiological markers (acini area and mass of lipid content in the fat body) and six molecular markers (*vg, TOR, JHAMT, ilp1, ilp2*, and *HSP70*) at the beginning of February, during the expected diapause continuation.

It was found that prolonged climate warming significantly weakened colony

development in Groups 25 and 35 during the fall season (as of September 18) compared to the control group. This observation supports the importance of day-night and seasonal temperature fluctuations for natural colony development [66], as other colony parameters were the same.

This colony development, as a response to ambient temperature, is closely related to honey bees' regulation of the nest microclimate. A temperature difference of 10°C between Groups 25 and 35 was assessed to study this. However, in August and September, bees adjusted this brood temperature difference to 3.403°C (**Fig. 3**). To achieve this, honey bees in Group 25 heated the brood area to a subnormal temperature of 30.00 ± 0.982 °C, while those in Group 35 cooled it to 33.398 ± 0.154 °C, which is slightly lower than the typical nest temperature of 34.5 ± 1.5 °C [67]. This well demonstrated known microclimate regulation [21, 22]. Although we don't know if the number of bees in colonies from Groups 25 or 35 may not have been sufficient to heat or cool more, nor if the subnormal brood temperature in Groups 25 and 35 may be a mechanism to rear long-lived winter bees, these brood temperatures did not prove fatal for bee survival during overwintering in our experiment (**S1 Table**). Furthermore, since Szentgyörgyi et al. [68] reported that short-lived honey bees live longer after developing at a low temperature (32°C), we preferred the latter explanation and hypothesized that reduction of brood temperature could be related to the development of physiological and molecular markers in winter bees, which are responsible for the processes of aging and longevity [69].

Generally, the physiological markers of winter honey bees include hypertrophied HG compared to newly emerged and forager summer bees [28]. Another physiological marker is an enlarged fat body compared to summer bees [27], resulting from the accumulation of nutrients, particularly lipids [30]. In our research, the acini area in honey bees from Groups 25 and the control was significantly larger (ANOVA, Duncan's post hoc, $p < 0.05$) than in those from Group 35. The same trend was observed in the mass of lipid content in the fat body, although the differences were not statistically significant. Given that the sampling was conducted on February 5, these physiological differences were indicative of the honey bees' diapause condition. The honey bees Groups 25 and control exhibited a higher body nutrient stock compared to the honey bee Group 35.

All of this closely associated with molecular markers [40, 70, 71], especially vg which is synthesized in the fat body [30]. Vg is then transferred to the HG and used as an amino acid donor for the synthesis of royal jelly to feed larvae after diapause ends [72]. Along with elevated vg levels, another molecular marker, suppressed juvenile hormone (JH) levels, is used to quantify the condition of winter honey bees compared to summer) honey bees [1, 33, 38]. These molecular levels remain stable during diapause and undergo a switch before the onset of brood rearing [38]. Notably, *vg* gene expression was significantly higher in honey bees from Group 25 and the control, whereas *JHAMT* gene expression was significantly lower compared to Group 35, suggesting an aging process in the latter bees.

To utilize these well-known interactions, we built Elastic Net Regression models for acini area and fat body mass **(Fig 5A, and B).** In this both models, only two genes — *vg* and *ilp2* — showed high positive and negative coefficient values, respectively. This indicates that during winter diapause, *vg* and *ilp2* gene expressions are expected to be higher and lower, respectively, with larger acini area and fat body mass in honey bees under normal conditions. This supports the well-known relationship between *vg* and physiological markers, and suggests a new relationship between molecular marker *ilp2* and physiology markers. However, in bees from Group 35, this relationship was reversed, which also implied some aging issues.

To identify genes that can significantly discriminate between honey bee groups, Discriminant Analysis (DA) was implemented. Three genes—*JHAMT, vg,* and *HSP70*—were identified with high scores, with the first two being already well-known. Even though the *HSP70* gene is synthesized in response to stress [73], Sarıoğlu-Bozkurt et al. [55] reported that its levels also increase in overwintered honey bees from fall to early spring and respond to different supplemental diets along with *vg*, demonstrating the complex regulation of this gene. We found that the highest *HSP70* gene expression was observed in the winter honey bees from Group 35, while the lowest was found in the Group control. Based on this, we suggest that honey bees from Group 35 responded on some a stress, possibly related to their different nutritional needs, which may later stimulate the ending of diapause and the initiation of earlier colony development compared to honey bees from Groups control and 25.

Also, the increased nutritional needs of honey bees from Group 35 are explained by the significant increase in *TOR1* gene expression, because this evolutionarily conserved gene integrates signals from nutrients (amino acids and energy) and growth factors to regulate cell growth [50, 74, 75]. Additionally, significantly higher *HSP70* and *TOR1* gene expressions in honey bees from Group 35, compared to Group control and Group 25, were associated with a decrease in fat body weight, although this decrease was not yet statistically significant. This supports the aging hypothesis for temperature-treated honey bees. However, *TOR1* gene was not selected by Data Mining methods as marker possibly because it had less fluctuation between Groups of honey bees compared to other genes.

The gene *ilp1* didn't show a clear effect in our experiment and requires careful consideration due to its complex metabolic relationships influenced by nutrition, age, and season [76, 77].

In summary, our study found that exposure to constant day-night temperatures of 25°C and 35°C negatively impacted colony growth in the fall. The 25°C temperature closely resembled natural fall conditions in Incheon, allowing honey bees to regulate the microclimate by lowering brood temperatures. This facilitated the development of winter bees with metabolic markers similar to those of the Group control. In contrast, the 35°C temperature hindered colony development, as honey bees in Group 35 were unable to reduce brood temperatures as effectively as those in Group 25, impairing winter bee development. In February, we observed

increased levels of *JHAMT, TOR1*, and *ilp2*, and decreased levels of *vg*, which were associated with reduced nutrient stores in the fat body mass and a smaller acini area. However, these changes did not result in fatal outcomes for honey bee survival in our experiment. These results can be used to correlate climate change factors, such as fall temperatures, with bee responses in winter. Understanding how organisms respond to extreme heat can inform apiculture practices and help develop guidelines for managing honey bees in winter to prevent losses. We believe this research could lead to further studies and the development of a test kit to identify early causes of aging.

Supporting information

S1 Table. Survival of honey bee colonies at the end of the overwintering (PDF)

S2 Table. PCR primer sequences. (PDF)

S1 Fig. The quality verification plot (specificity vs sensitivity) of Elastic Net Regression model demonstrated a perfectly fitted model linking physiology to molecular markers (gene expression profile). (.tif)

S1 Fig. Gel electrophoresis to verify the amplicons after qPCR. (.tif)

S1 File. Raw data from sensors and XLSTAT 2022.xlsx

S2 File. Raw data of acini area, fat body mass, and colony perfomance.xlsx

S3 File. Raw data qPCR.xlsx

S4 File. Stat analysis in XLSTAT.xlsx

Acknowledgments

The authors thank BeeOnfarm company (Republic of Korea) for providing the equipment to record the in-hive microclimate. Mention of trade names or commercial products in this publication is solely to provide specific information and does not imply recommendation or endorsement by the INU.

Author Contributions

Conceptualization: Yumi Yun, Hyung-Wook Kwon

Data curation: Yumi Yun

Formal analysis: Olga Frunze, Ravil R. Garafutdinov, Hyunjee Kim

Investigation: Hyung-Wook Kwon, Young-Eun Na

Methodology: Ravil R. Garafutdinov, Olga Frunze, Yumi Yun

Resources: Hyung-Wook Kwon, Young-Eun Na, Hyunjee Kim

Software: Olga Frunze, Ravil R. Garafutdinov

Supervision:, Hyung-Wook Kwon, Young-Eun Na

Visualization: Olga Frunze

Writing-original draft: Olga Frunze, Yumi Yun

Writing-review&editing: Olga Frunze, Yumi Yun, Hyunjee Kim, Hyung-Wook Kwon

Reference

1. Döke MA, Frazier M, Grozinger CM. Overwintering honey bees: biology and

management. Curr Opin Insect Sci. 2015; 10:185-93.

2. Hristov P, Shumkova R, Palova N, Neov B. Factors associated with honey bee colony losses: a mini-review. Vet Sci. 2020; 7:166.
3. Gray A, Adjlane N, Arab A, Ballis A, Brusbardis V, Dougla AB. et al. Honey bee colony loss rates in 37 countries using the COLOSS survey for winter 2019–2020: the combined effects of operation size, migration and queen replacement. J Apic Res. 2023; 62:204-10.
4. Calovi M, Grozinger CM, Miller DA, Goslee SC. Summer weather conditions influence winter survival of honey bees (*Apis mellifera*) in the northeastern United States. Sci Rep. 2021; 11: 1553.
5. Dosio A, Mentaschi L, Fischer EM, Wyser K. Extreme heat waves under 1.5°C and 2°C global warming. Environ Res. 2018; 13: 054006.
6. Lamb MJ. Temperature and lifespan in *Drosoph*. Nat. 1968; 220: 808–809.
7. Liu RK, Walford RL. Increased growth and lifespan with lowered ambient temperature in annual fish *Cynolebias adloffi*. Nature. 1966; 212: 1277–78.
8. Mishra DM, Bhunia P, Shubham S, Sen,R, Bhunia R, Gulia J, et. al. The impact of weather changes on honey bee populations and disease. J Adv Zool. 2023; 44: 180–90.
9. Conti NB, Sanchez-Alavez M, Winsky-Sommerer R, Morale MC, Lucero J, Brownell S, et al. Transgenic mice with a reduced core body temperature have an increased life span. Science. 2006; 314, 825-8.

10. Fahrenholz L, Lamprecht I, Schricker B. Thermal investigations of a honey bee colony: thermoregulation of the hive during summer and winter and heat production of members of different bee castes. J Comp Physiol. 1989; 159: 551-60.

11. Switanek M, Crailsheim K, Truhetz H, Brodschneider R. Modelling seasonal effects of temperature and precipitation on honey bee winter mortality in a temperate climate. Sci Total Environ. 2017; 579: 1581–7.

12. van Dooremalen C, Gerritsen L, Cornelissen B, van der Steen JJ, van Langevelde F., Blacquière T. Winter survival of individual honey bees and honey bee colonies depends on level of *Varroa destructor* infestation. PloS ONE. 2012; 7: e36285.

13. Morawetz L, Köglberger H, Griesbacher A, Derakhshifar I, Crailsheim K, Brodschneider R, et al. Health status of honey bee colonies (*Apis mellifera*) and disease-related risk factors for colony losses in Austria. PLoS ONE. 2019; 14: e0219293.

14. Fluri P, Lüscher M, Wille H, Gerig L. Changes in weight of the pharyngeal gland and hemolymph titers of juvenile hormone, protein and vitellogenin in worker honey bees. J Insect Physiol. 1982; 28:61-8.

15. Fluri P, Wille H, Gerig L, Luscher M. Juvenile hormone, vitellogenin and hemocyte composition in winter worker honeybees (*Apis mellifera*). Experientia 1977; 33: 1240–41.

16. Crailsheim K. Dependence of protein-metabolism on age and season in the honeybee (*Apis mellifica carnica* Pollm). J. Insect Physiol. 1986; 32:629–34.

17. Denlinger DL. Insect diapause: from a rich history to an exciting future. J Exp Biol. 2023; 226 (4): 245329.

18. Münch D, Kreibich CD, Amdam GV. Aging and its modulation in a long-lived worker caste of the honey bee. J Exp Biol. 2013; 216: 1638–49.

19. Coulibaly KA, Majeed MZ, Sayed SM, Yeo K. Simulated climate warming influenced colony microclimatic conditions and gut bacterial abundance of honeybee subspecies *Apis mellifera ligustica* and *A. mellifera sinisxinyuan*. J Apic Sci. 2022; 66: 15-27.

20. Simpson J. Nest Climate regulation in honey bee colonies. Science. 1961; 133:1327–1333.

21. Abou-Shaara H, Owayss A, Ibrahim Y, Basuny N. A review of impacts of temperature and relative humidity on various activities of honey bees. Insectes Soc. 2017; 64: 455 – 63.

22. Bencsik M, McVeigh A, Tsakonas C, Kumar T, Chamberlain L, Newton MI. A Monitoring system for carbon dioxide in honeybee hives: An indicator of colony health sensors (Basel). 2023; 23(7):3588.

23. Winston ML. The Biology of the honey bee. Harvard University Press, Cambridge. 1987; 294.

24. Hartfelder K, Engels W. Social insect polymorphism: hormonal regulation of plasticity in development and reproduction in the honeybee. Curr Top Dev Biol. 1998; 40:45-77.

25. Rodrigues MA, Flatt T. Endocrine uncoupling of the trade-off between reproduction and somatic maintenance in eusocial insects. Curr Opin Insect Sci. 2016; 16:1e8.

26. Bresnahan ST, Döke MA, Giray T, Grozinger CM. Tissue-specific transcriptional patterns underlie seasonal phenotypes in honey bees (*Apis mellifera*). Mol Ecol. 2022; 31:174-84.

27. Brejcha M, Prušáková D, Sábová M, Peska V, Černý J, Kodrík D, et al. Seasonal changes in ultrastructure and gene expression in the fat body of worker honey bees. J Insect Physiol. 2023; 146:104504.

28. Deseyn J, Billen J. Age-dependent morphology and ultrastructure of the hypopharyngeal gland of *Apis mellifera* workers (Hymenoptera, Apidae) Apidologie. 2005; 36: 49-57.

29. Dohanik VT, Medeiros-Santana L, Santos CG, Santana, WC, Serrão JE. Expression and function of the vitellogenin receptor in the hypopharyngeal glands of the honey bee *Apis mellifera* (Hymenoptera: Apidae) workers. Arch Insect Biochem Physiol. 2024; 116: e22120.

30. Smedal B, Brynem M, Kreibich C, Amdam G. Brood pheromone suppresses physiology of extreme longevity in honeybees (*Apis mellifera*). J Exp Biol. 2009; 212: 3795-801.

31. Arrese EL, Soulages JL. Insect fat body: energy, metabolism, and regulation. Annu Rev Entomol. 2010; 55: 207-25.

32. Amdam GV, Aase AL, Seehuus SC, Kim Fondrk M, Norberg K, Hartfelder K. Social

reversal of immunosenescence in honey bee workers. Exp Ger Ontol. 2005; 40: 939-47.

33. Knoll S, Pinna W, Varcasia A, Scala A, Cappa MG. The honey bee (*Apis mellifera* L., 1758) and the seasonal adaptation of productions. Highlights on summer to winter transition and back to summer metabolic activity. A review. Livest Sci. 2020; 235: 104011.

34. Schilcher F, Scheine R. New insight into molecular mechanisms underlying division of labor in honeybees. Curr Opin Insect Sci. 2023; 59: 101080.

35. Scheiner R, Reim T, Søvik E, Entler BV, Barron AB, Thamm M. Learning, gustatory responsiveness and tyramine differences across nurse and forager honeybees. J Exp Biol. 2017; 220:1443-50.

36. Corby-Harris V, Snyder LA. Measuring hypopharyngeal gland acinus size in honey bee (*Apis mellifera*) workers. J Vis Exp. 2018; 139:58261.

37. Corby-Harris V, Snyder L, Meador C. Fat body lipolysis connects poor nutrition to hypopharyngeal gland degradation in *Apis mellifera*. J Insect Physiol. 2019; 116:1-9.

38. Amdam GV, Omholt SW. The regulatory anatomy of honeybee lifespan. J Theor Biol. 2002; 216: 209-28.

39. Ali H, Alqarni AS, Iqbal J, Owayss AA, Raweh HS, Smith BH. Effect of season and behavioral activity on the hypopharyngeal glands of three honey bee *Apis mellifera* L. races under stressful climatic conditions of central Saudi Arabia. J Hymenopt Res.

2019; 68: 85-101.

40. Alaux C, Soubeyrand S, Prado A, Peruzzi M, Maisonnasse A, Vallon J et al. Measuring biological age to assess colony demographics in honeybees. PLoS ONE. 2018; 13(12): e0209192.

41. Diebel LWM, Rockwood K. Determination of biological age: geriatric assessment *vs* biological biomarkers. Curr Oncol Rep. 2021; 23: 104.

42. Elekonich MM, Schulz DJ, Bloch G, Robinson GE. Juvenile hormone levels in honey bee (*Apis mellifera* L.) foragers: foraging experience and diurnal variation. J Insect Physiol. 2001; 47:1119-25.

43. Guidugli KR, Nascimento AM, Amdam GV, Barchuk AR, Omholt S, Simoes ZL et al. 2005. Vitellogenin regulates hormonal dynamics in the worker caste of a eusocial insect. FEBS Lett. 2005; 579: 4961-65.

44. Amdam GV, Page RE. The developmental genetics and physiology of honeybee societies. Anim Behav. 2010;79(5): 973-80.

45. Goodman WG, Granger NA. The juvenile hormones. In: Lawrence IG, Kostas I, Sarjeet SG. Comprehensive Molecular Insect Science. Elselvier. 2005; pp. 319-408.

46. Li W, Huang ZY, Liu F, Li Z, Yan L, et al. Molecular cloning and characterization of juvenile hormone acid methyltransferase in the honey bee, *Apis mellifera*, and its differential expression during caste differentiation. PLoS ONE. 2013; 8:e68544.

47. Egi Y, Sakamoto K. Genome-wide screening of genes involved in programming diapause in the next generation in silkworm, *Bombyx mori* (Lepidoptera:

Bombycidae). Eur J Entomol. 2022; 119:405-12.

48. Corona M, Velarde RA, Remolina S, Moran-Lauter A, Wang Y, Hughes KA et al. Vitellogenin, juvenile hormone, insulin signaling, and queen honey bee longevity. Proc Natl Acad Sci USA. 2007; 104: 7128-33.

49. Zhang W, Wang L, Zhao Y, Wang Y, Chen C, Hu Y, et al. Single-cell transcriptomic analysis of honeybee brains identifies vitellogenin as a caste differentiation-related factor. iScience. 2022; 25: 104643.

50. Barth JMI, Szabad J, Hafen E, Köhler K. Autophagy in *Drosophila* ovaries is induced by starvation and is required for oogenesis. Cell Death Differ. 2010; 18: 915.

51. Patel A, Fondrk MK, Kaftanoglu O, Emore C, Hunt G Frederick K, et al. The making of a queen: TOR pathway is a key player in diphenic caste development. PLoS ONE. 2007; 2: e509.

52. Laplante M, Sabatini DM. mTOR signaling in growth control and disease. Cell. 2012; 149:274-293.

53. Johnson SC, Rabinovitch PS, Kaeberlein M. mTOR is a key modulator of aging and age-related disease. Nature. 2013; 493:338-345.

54. Zoncu R, Efeyan A, Sabatini DM. mTOR: from growth signal integration to cancer, diabetes and aging. Nat Rev Mol Cell Biol. 2011; 12:21-35.

55. Sarioğlu-Bozkurt A, Topal E, Güneş N, Üçeş E, Cornea-Cipcigan M, Coşkun İ, et al. Changes in vitellogenin (*Vg*) and stress protein (*HSP 70*) in honey bee (*Apis mellifera anatoliaca*) groups under different diets linked with physico-chemical,

antioxidant and fatty and amino acid profiles. Insects. 2022; 13: 985.

56. Delaplane KS, van der Steen J, Guzman-Novoa E. Standard methods for estimating strength parameters of *Apis mellifera* colonies. J Apic Res. 2013; 52: 1-12.

57. Martin JS. Lipid composition of fat body and its contribution to the maturing oöcytes in *Pyrrhocoris apterus*. J Insect Physiol. 1969; 15:1025-45.

58. Human H, Brodschneider R, Dietemann V, Dively JD, Ellis E, Forsgren I, et al. Miscellaneous standard methods for *Apis mellifera* research. J Apic Res. 2013; 52:1-53.

59. Livak KJ, Schmittgen TD. Analysis of relative gene expression data using real-time quantitative PCR and the 2(-Delta Delta C(T)) Method. Methods. 2001; 25:402–408.

60. Bustin SA, Benes V, Garson JA, et al. The MIQE guidelines: minimum information for publication of quantitative real-time PCR experiments. Clin Chem. 2009; 55:611-22.

61. Moses AM. Statistical modeling and machine learning for molecular biology. CRC Press Taylor & Francis Group. 2017: 280 p.

62. Jaqaman K, Danuser G. Linking data to models: data regression. Nat Rev Mol Cell Biol. 2006; 7:813–819.

63. Hajian-Tilaki K. Receiver operating characteristic (ROC) curve analysis for medical diagnostic test evaluation. Caspian J Intern Med. 2013; 4: 627-35.

64. Medrzycki P, Sgolastra F, Bortolotti L, Bogo G, Tosi S, Padovani E, et al. Influence of brood rearing temperature on honey bee development and susceptibility to

poisoning by pesticides. J Apic Res. 2010; 49:52-59.

65. Quinlan GM, Feuerborn C, Hines HM, Grozinger CM. Beat the heat: thermal respites and access to food associated with increased bumble bee heat tolerance. J Exp Biol. 2023; 226: jeb245924.

66. Stalidzans E, Berzonis A, Temperature changes above the upper hive body reveal the annual development periods of honey bee colonies. Comput Electron Agric. 2013; 90: 1-6.

67. Kovac H, Stabentheiner A, Brodschneider R. Contribution of honeybee drones of different age to colonial thermoregulation. Apidologie. 2009; 40:82-95.

68. Szentgyörgyi H, Czekońska K., Tofilski A. Honey bees are larger and live longer after developing at low temperature. J Therm Biol. 2018; 78:219-226.

69. Kovac H, Crailsheim K. Lifespan of *Apis mellifera carnica* Pollm. infested by *Varroa jacobsoni* Oud. in relation to season and extent of infestation. J Apic Res. 1988; 27: 230-8.

70. Kaur R, Singh D. Molecular markers a valuable tool for species identification of insects: A review. Ann Entomol. 2020; 38:1-20.

71. Daniels BC, Wang Y, Page RE Jr, Amdam GV. Identifying a developmental transition in honey bees using gene expression data. PLoS Comput Biol. 2023; 9(9):e1010704.

72. Ramanathan ANKG, Nair AJ, Sugunan VS. 2018. A review on royal jelly proteins and peptides. J Funct Foods. 2018; 44: 255-64.

73. Zhang Y, Liu Y, Zhang J, Guo Y, Ma E. Molecular cloning and mRNA expression of heat shock protein genes and their response to cadmium stress in the drasshopper *Oxya chinensis*. PLoS ONE. 2015; 10: e0131244.

74. Fingar D, Blenis J. Target of rapamycin (TOR): an integrator of nutrient and growth factor signals and coordinator of cell growth and cell cycle progression. Oncogene. 2004; 23: 3151–71.

75. Fahrbach SE, Nambu JR, Schwartz LM. Programmed cell death in insects. Insect Mol Biol Biochem. 2012; 12:419–449.

76. Nilsen KA, Ihle KE, Frederick K, Fondrk MK, Smedal B, Hartfelder K, et al. Insulin-like peptide genes in honey bee fat body respond differently to manipulation of social behavioral physiology. J Exp Biol. 2011; 1:1488-97.

77. Chowański S, Walkowiak-Nowicka K, Winkiel M, Marciniak P, Urbański A, Pacholska-Bogalska J. Insulin-like peptides and cross-talk with other factors in the regulation of insect metabolism. Front Physiol. 2021; 12:701203.

Data Availability Statement: All relevant data are the manuscript and its Supporting information files.

Funding: This work was carried out with the support of the Cooperative Research Program for Agriculture Science & Technology Development (Project No. RS-2023-00232749) and the Priority Research Centers Program through the National Research Foundation of Korea (NRF) funded by the Ministry of Education

(2020R1A6A1A03041954). The funders had no role in study design, data collection and analysis, decision to publish, or preparation of the manuscript.

Competing interests: The authors have declared that no competing interests exist.

Abbreviations: DA, discriminant analysis; AHC, Agglomerative Hierarchical Clustering; FD, Fisher distance; HG, hypopharyngeal glands; JH, juvenile hormone; *JHAMT, Juvenile Hormone Acid Methyltransferase; vg, vitellogenin; ilp1, insulin-like peptide 1; ilp2, insulin-like peptide 2; HSP70, heat shock protein 70; TOR1, target of rapamycin 1; RpL32, Ribosomal protein L32.*

Click here to access/download;Figure;Fig 1 ABC.TIF

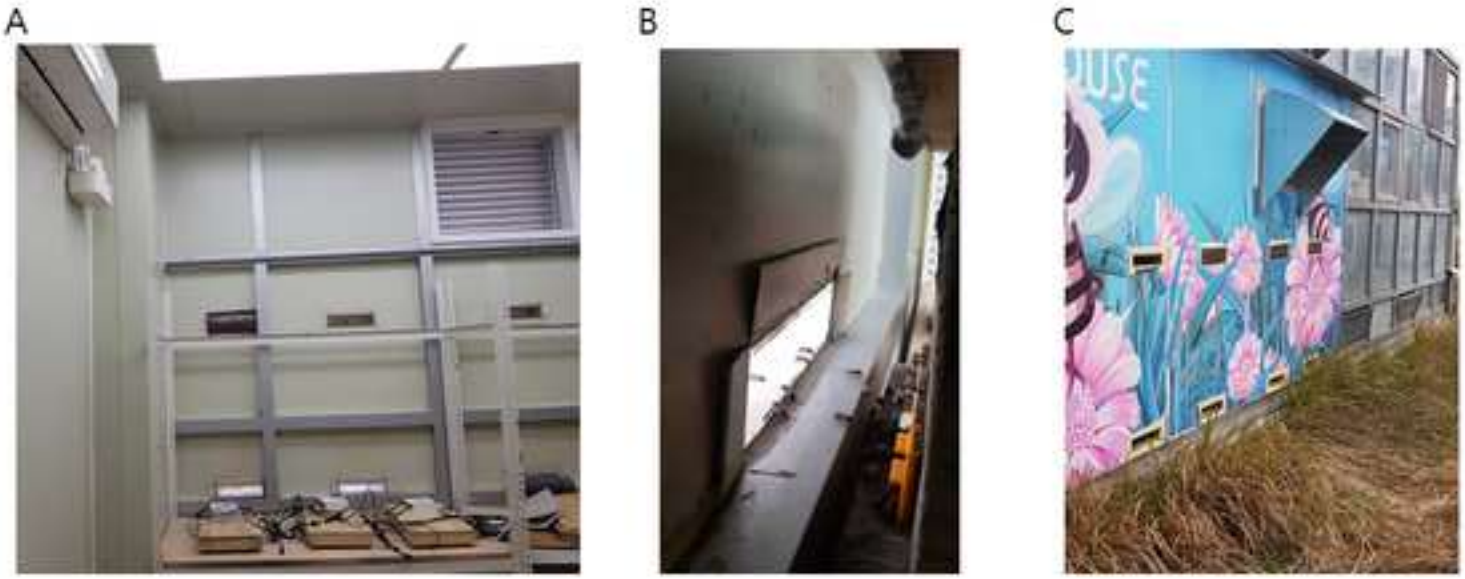

Click here to access/download;Figure;Fig 1 D.TIF

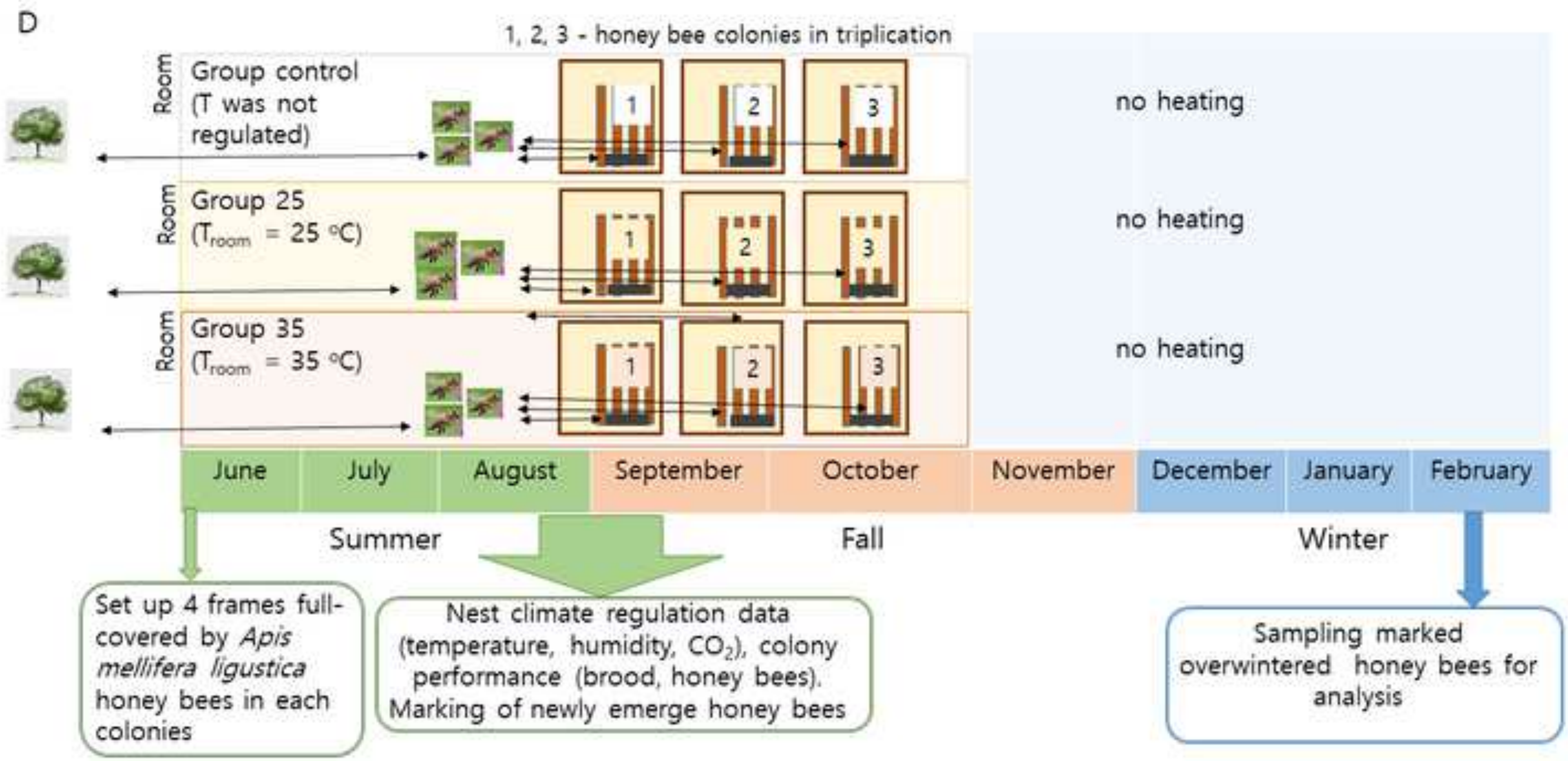

Click here to access/download;Figure;Fig 1 E.TIF

E

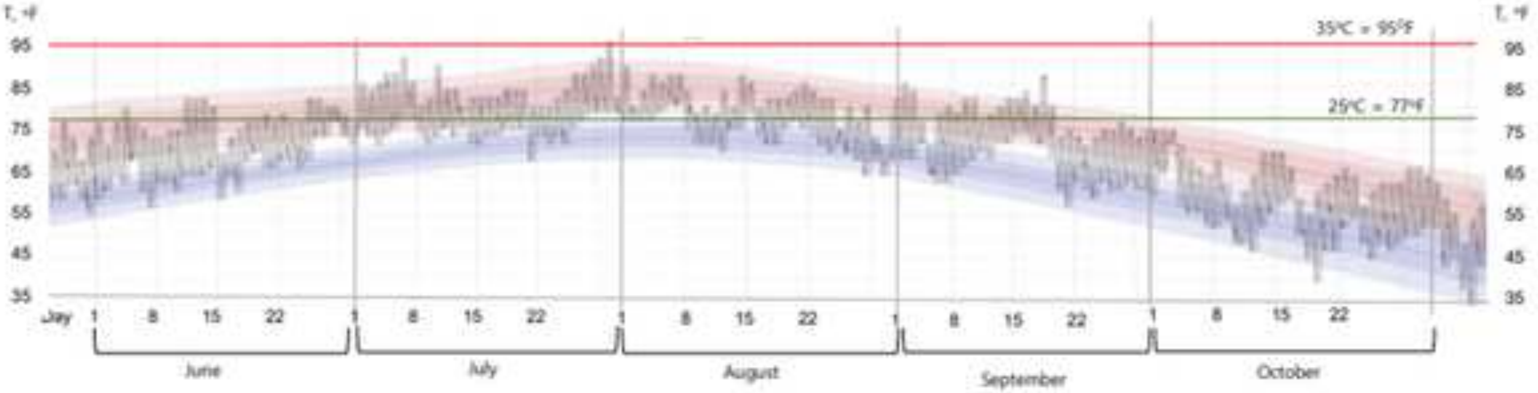

The daily range of reported temperatures (gray bars) and 24-hour highs (red ticks) and lows (blue ticks), placed over the daily average high (faint red line) and low (faint blue line) temperature, with 25th to 75th and 10th to 90th percentile bands.

Click here to access/download;Figure;Fig 2.TIF

Apis mellifera ligustica honey bees

Month of sampling		Group control (3 colonies)	Group 25 (3 colonies)	Group 35 (3 colonies)
Experimental condition (summer, September, and October)			T_{room} = 25 °C	T_{room} = 35 °C
**August September**	Data set 1 (brood microclimate and colony development)		Nest climate regulation	
	Statistics of data set 1		T-test (*p* < 0.05)	
**February**	Sampling of honey bees, n	(for physiology markers): 30+30+30<br>(for molecular markers): 10+10+10	30+30+30<br>10+10+10	30+30+30<br>10+10+10
	Data set 2	Two physiology markers (hypopharengial glands (HG, acini area) and fat body mass)<br>Molecular marker genes related to nutrition (*vg*, *ilp1*, *ilp2*, *TOR1*), development (*JHAMT*), and stress response (*HSP70*).		
	Statistics of data set 2	1. Discriminant analysis (define the variables with high discrimination score);<br>2. Agglomerative Hierarchical Clustering (check the natural groupings within dataset);<br>3. Elastic Net Regression (model the relationship between HG (acini area) and fat body mass with molecular markers);<br>4. ANOVA, Duncan's post-hoc test (comparison of significance within each variable, *p* < 0.05).		

Click here to access/download;Figure;Fig 3 ABC.TIF

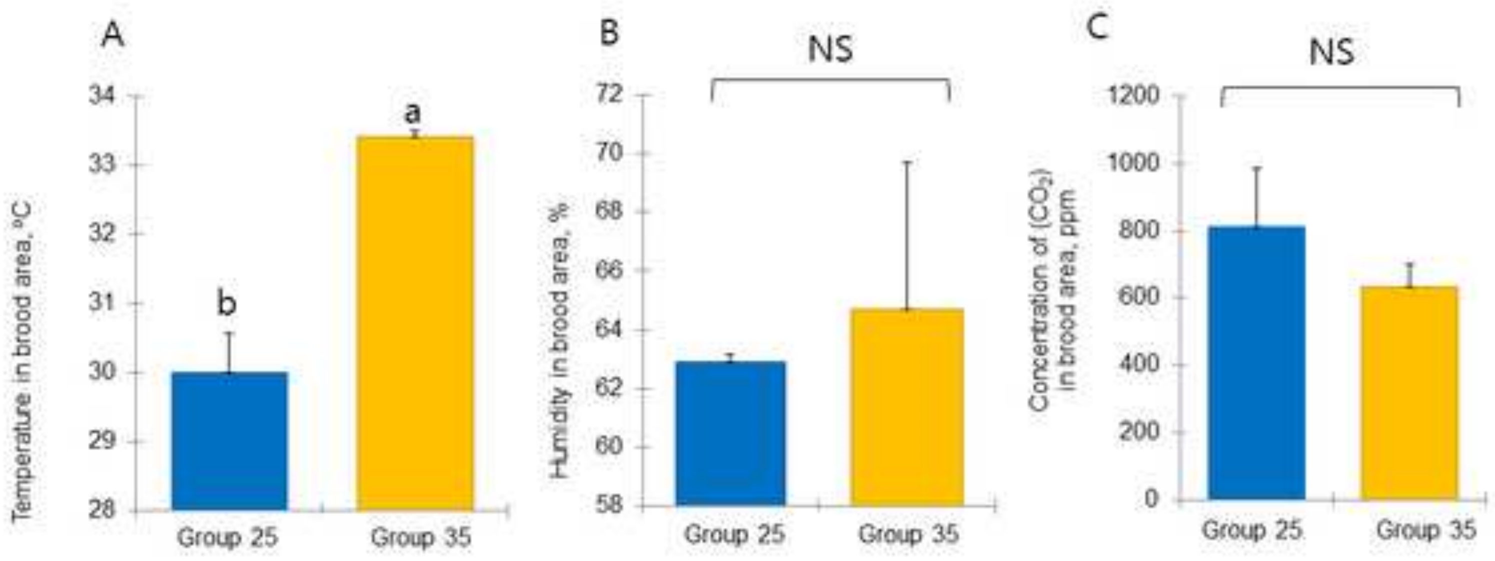

Click here to access/download;Figure;Fig 3 DEF.TIF

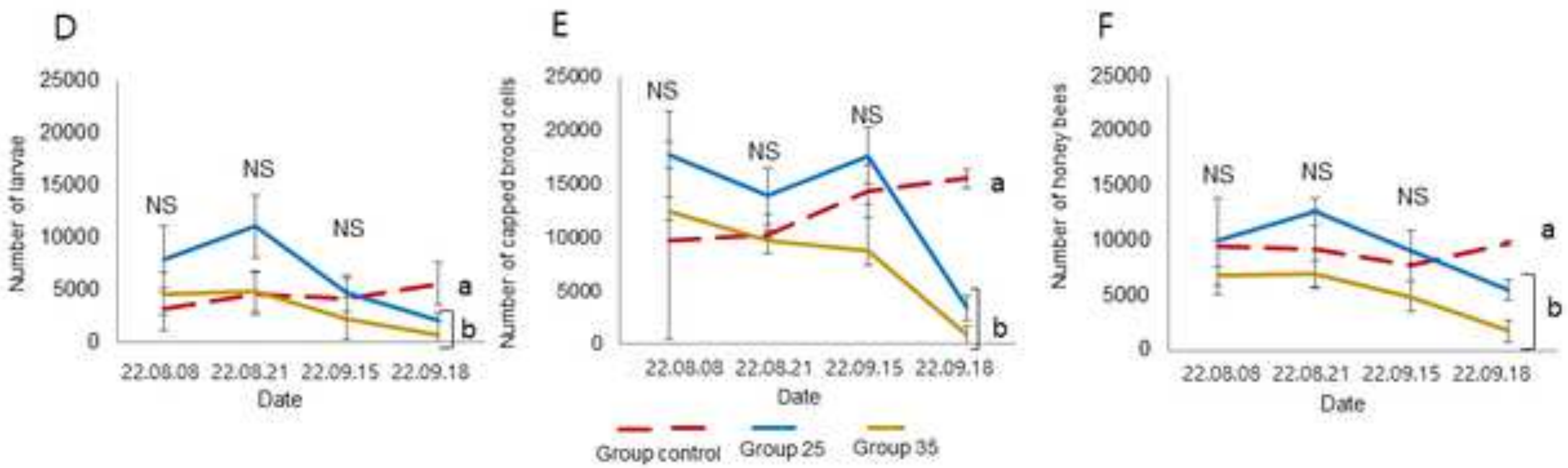

Click here to access/download;Figure;Fig 4.TIF

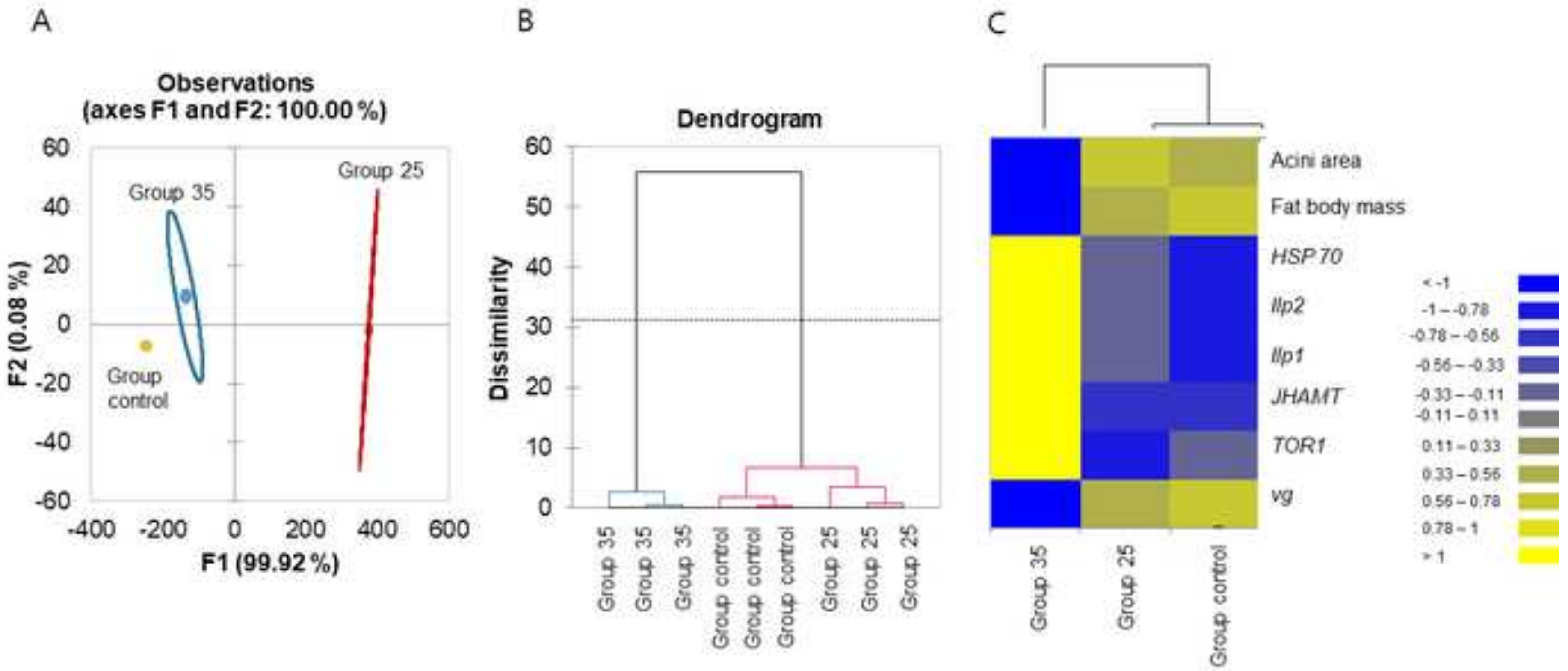

Click here to access/download;Figure;Fig 5.TIF

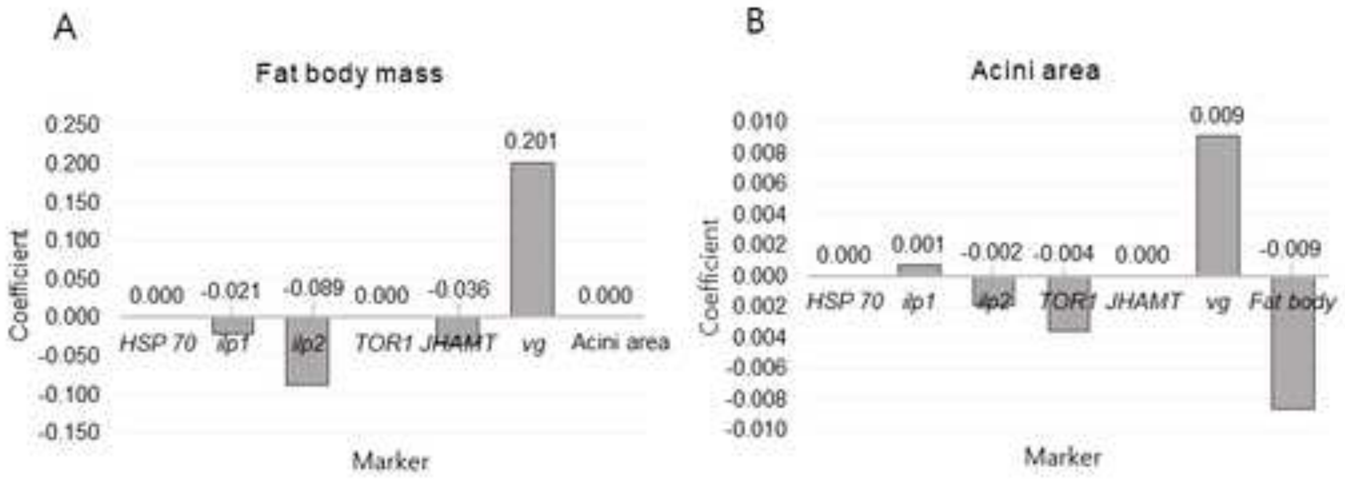

Click here to access/download;Figure;Fig 6.TIF

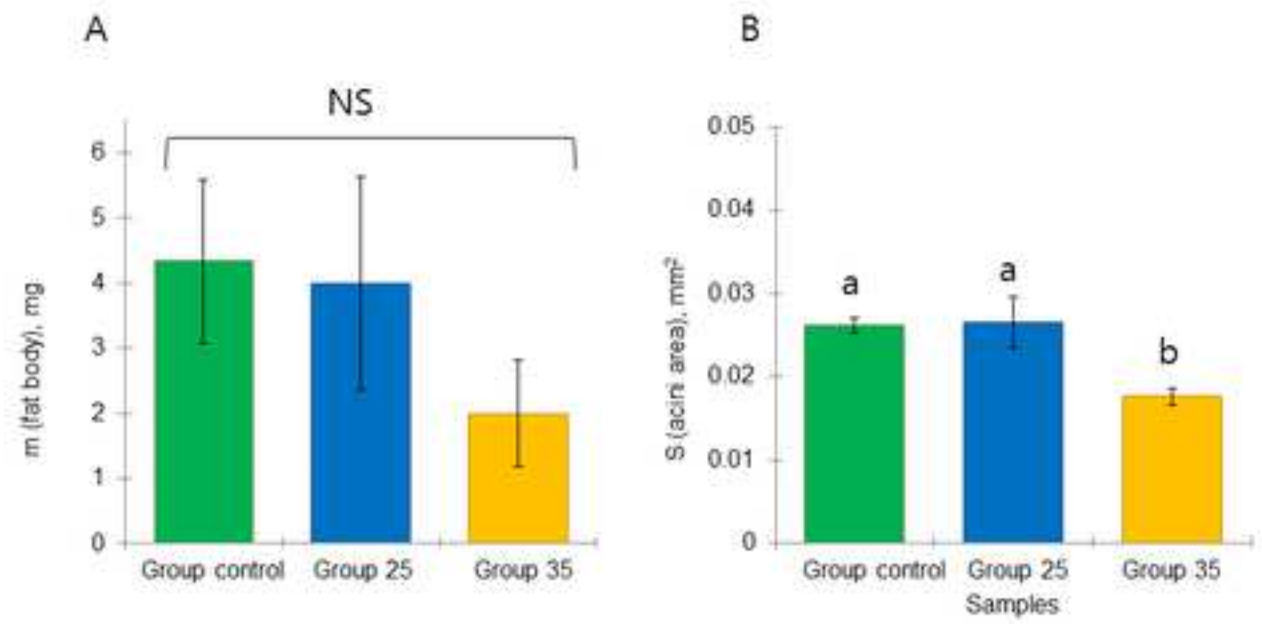

Click here to access/download;Figure;Fig 7 ABC.TIF

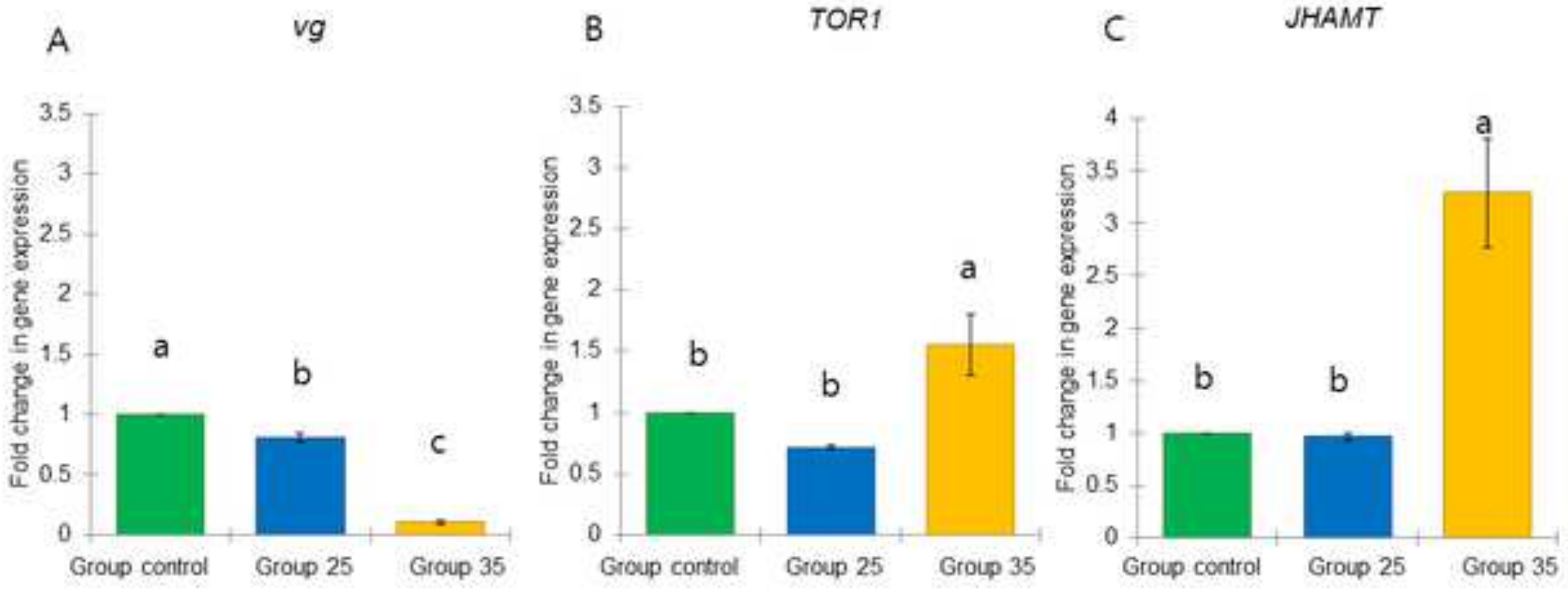

Click here to access/download;Figure;Fig 7 DEF.TIF

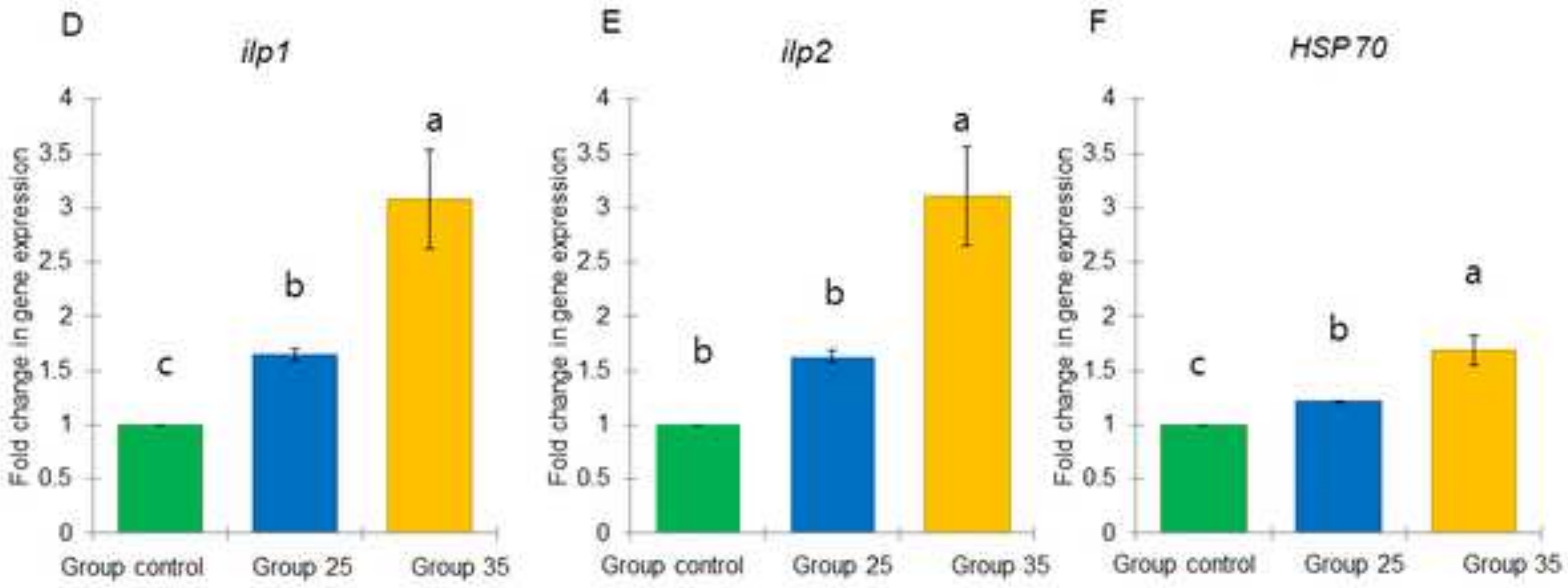

Click here to access/download;Figure;S1 Fig.TIF

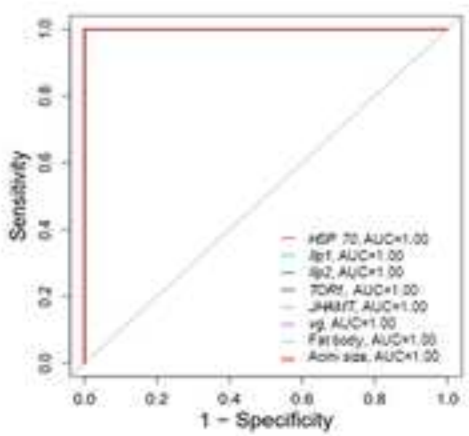

Click here to access/download
Supporting Information - Compressed/ZIP File Archive
Supporting information.zip

Revised Manuscript with Track Changes

Research article

The effect of seasonal temperatures on the aging of the overwintered honey bee

Olga Frunze[&1¶], Yumi Yun[&1¶], Hyunj-Jee Kim[1], Ravil R. Garafutdinov[2], Young-Eun Na[3]*, and Hyung-Wook Kwon[1]*

[1]Department of Life Sciences & Convergence Research Center for Insect Vectors (CRCIV), Incheon National University, 119 Academy-ro, Yeonsu-gu, Incheon, Republic of Korea 22012; ~~frunzeon@gmail (O.F.); yym8804@naver.com (Y.Y.); beamed79@hanmail.net (H.J.K.)~~

[2]Institute of Biochemistry and Genetics, Ufa Federal Research Center, Russian Academy of Sciences, 450054, prosp. Oktyabrya, 71, Ufa, Bashkortostan, Russian Federation ~~garafutdinovr@gmail.com (R.R.G.)~~

[3]Agro-materials Industry Division, Rural Development Administration (RDA), Wanju 55365, Republic of Korea

[¶&]These authors contributed equally to this work

*Correspondence: hwkwon@inu.ac.kr (H.-W.K.) youngman99@korea.kr (Y.-E.N.)

Short title: **Aging of honey bees at the end of winter**

Formatted: Left: 0.75", Right: 0.75", Top: 1"

Formatted: Font: Bold

Formatted: Font: 14 pt, Superscript

Formatted: Font: Bold

Formatted: Font: 14 pt, Superscript

Formatted: Font: Bold

Formatted: Font: 14 pt

Abstract

Formatted: Font: 18 pt

Honey bee physiology follows an annual cycle, with winter bees living ten times longer than summer bees. Their transition can be disrupted by climate change. Several climate factors, mainly temperature, may contribute to global losses of winter bees. We simulated climate warming by maintaining constant temperatures of ~~Honey bee workers' survival hinges on an annual cycle, marked by extended longevity in winter workers (long-lived), approximately 10 times longer than in their summer workers (short-lived). While previous studies indicate temperature's impact on longevity, the underlying molecular mechanism remains elusive. In our study, we maintained constant temperatures in rooms at~~ 25°C (Group 25) and 35°C (Group 35) in rooms around hives from June to October, ~~simulating climate warming,~~ whileith~~ith~~ a Group control ~~group experiencing~~ experienced natural conditions. Colony performance was assessed in August and September.~~, and i~~ In February, workers were examined for physiological traits (a~~A~~cini area~~us size~~ and f~~F~~at body mass~~ weight~~) and ~~expression of known task-related~~ molecular markers (*vg* and *JHAMT*), along with expected markers (*ilp1, i~~p~~lp2, TOR1*, and *HSP70*). Our findings suggest that temperature decreases around winter worker broods from Group 25 in the fall~~autumn~~ le~~a~~d to their slower physiological aging in winter compared to Group 35 workers, supported by

Formatted: Left

~~physiology and task-related~~ markers. Furthermore, the end of diapause, when aging speeds up, was coupled with upregulation~~we observed a relation between aging and~~ of *HSP70*, *ilp2*, and *TOR1* genes~~s~~. This research~~, which~~ can predict~~may serve as additional markers~~the winter status of bees~~,~~ in response to fall climate warming and help beekeepers implement strategies to prevent bee losses. ~~necessitating further research.~~ Additionally, it~~This approach~~ could enhance molecular aging identification in insect models, advancing research on climate warming effects ~~-related research~~.

Formatted: Font: 18 pt

Introduction

~~Honey bees play a crucial role in human life as producers of health and medicinal products, pollinators on agricultural farms, and contributors to ecosystems worldwide.~~ Honey bees play a crucial role in human life as pollinators on agricultural farms, producers of honey, royal jelly, and other products, which are valued for their health and medicinal benefits, and contributors to ecosystems worldwide. However, the loss of~~honey~~ bees has been reported over the last two decades, particularly at the end of the overwintering period [1-3]~~(Döke et al., 2015; Hristov et al., 2020; Insolia et al.,~~

Formatted: Left

Formatted: Highlight

Formatted: Highlight

Formatted: Highlight

2022; Gray et al., 2023).

Several factors arising during the year can contribute to this [4]. For example, climate warming or cooling [5] impacts lifespan in flies [6], fish [7], honey bees [8], and rodents [9]. Other factors related to climate change include the quality of forage [10, 11], beekeeper management practices [12, 13], and pesticide exposure from land use [11]. Although we can try to avoid some of these factors, effective methods to mitigate the impacts of climate change and prevent honey bee winter mortality are currently lacking.

Various factors contribute to honey bee winter mortality, and currently, we do not have effective methods to prevent it.

To advance in this direction, it is necessary to elucidate clarify the general the processes occurring in late summer and fall that are involved in the transition from short-lived workers to long-lived workers [14, 15], onesas these processes may be disrupted by climate change. This transition has been associated withwith external (environmental) factors that leadinfluence to internal (metabolic) processes.

Known external trigger factors includeare a decrease in pollen availability [16, 17](Crailsheim, 1986; Denlinger, 2023), and cessation of brood rearing [15, 18](Fruri et al., 1982; Münch et al., 2013),. and declining ambient temperaturesAmbient temperature, as factor of climate change, can (Simpson, 1961; Medrzycki et al., 2012).

Formatted: Highlight

Formatted: Font: (Default) Times New Roman, 14 pt

Formatted: Left, Indent: First line: 1 ch

Formatted: Left

Formatted: Highlight

Formatted: Highlight

Although seems honey bees maintain a consistent brood microclimate (Abou-Shaara et al., 2017; Bencsik et al., 2023), climate warming has been shown to significantly alterincrease brood temperature in summer in summer [19], (Coulibaly et al., 2022)despite honey bees efforts to maintainmaintain a consistent brood microclimate [20-22](Abou-Shaara et al., 2017; Bencsik et al., 2023).

, and a similar effect can be expected in fall, although this has not been thoroughly studied yet. With the onset of fall, externalnvironmental triggerstrigger factors initiate, the transition of honey bees isthrough initiated internal processes, leading to the development of winter workers with prolonged longevity, which can lastreach up to 8 months [1] (Döke et al., 2015). Briefly, this transition It exhibits the classic insect diapause phenotype, characterized by reduced metabolic activity and altered hormonal profiles [14, 23-26] and reduced metabolic activity (Fluri et al., 1982; Winston, 1987; Hartfelder and Engels, 1998; Rodriges and Flatt, 2016; Bresnahan et al., 2022). Unlike other insects, the reduced metabolic activity of honey bees during diapause [17] includes vibrating their wing muscles to generate heat and maintain warmth within the winter cluster. This process depends on a constant energy source and requires a pure carbohydrate diet [27], which is not necessary for other diapausing insects and impacts internal processes.

–In winterhoney bees, the physiology of internal processes relatedthis to this hormone-regulated phenomenon is well-described., It manifestssing as hypertrophied increased secretory vesicles and increased Aacinius diametersize (in the

Formatted: Highlight

Formatted: Highlight

Formatted: Highlight

Formatted: Font: (Default) Times New Roman, 14 pt

hypopharyngeal gland (, HG) compared to newly emerged and forager summer bees [28],. HG development is known to be positively correlated with vitellogenin (*vg*) throughout a worker’s lifespan. However, *vg* is synthesized in the fat body [29], which is more developed Fat body weightin winter bees compared to summer bees [27],. The fat body mass results from the accumulation of nutrients [30], which lipids constituting over >90 % of the mass in the form of triglycerides. These triglycerides are synthesized from dietary carbohydrates and fatty acids and are used not only for *vg* synthesis [30] but also for producing pheromones, phospholipids, and wax [31].

Significant differences between honey bees and other insects have been observed in the mutually inhibitory interaction between vg (protein) and the juvenile hormone (JH) [32]. An iand vitellogenin (vg)ncreased level of vg (protein and gene)s, alongside decreased levels of the juvenile hormone (JH) [1, 15, 33] levels (Fluri et al., 1977; Döke et al., 2015; Knoll et al., 2020), has been noted, however, . the molecular mechanism of this complex relationship remains unclear [34]. It is known that the JH biosynthetic pathway begins in the glands in the brain complex and responds to sensory inputs that underpin the division of labor [34, 35]. However, unlike other insects during diapause (Denlinger, 2023), honey bees require a pure carbohydrate diet, as their metabolism facilitates the heating of the winter cluster by vibrating the wing muscles (Brejcha et al., 2023). The duration of diapause is crucial for successful honey bee overwintering (Denlinger, 2023). Furthermore, at the end of winter, diapause nursesbees gradually transition to nursing brood care and foraging

Formatted: Font: Italic

Formatted: Font: Italic

Formatted: Font: Not Italic

Formatted: Highlight

physiology [1](Döke et al., 2015). The last transition is characterized by several physiological and molecular changes, including a decrease in aAciniius areasize [36] (Corby-Harris and Snyder, 2018) and fFat body mass [37](Corby-Harris et al., 2019), as well as reductions in vg levels and increases in juvenile hormone (JH) levels [15, 38](Fluri et al., 1977; Amdam and Omholt, 2002). These changes contribute to seasonal effects and behavioral activities [39](Ali et al., 2019), inevitably leading to reduced longevity and increased aging compare to diapause condition [40, 41](Alaux et al., 2018; Diebel and Rockwood, 2021).

Alongside with the well-known age-related marker gene *vgtask-related molecular marker genes vg and hormone JH* [32, 42, 43, 44](Elekonich et al., 2001; Amdam et al., 2005; Guidugli et al., 2005), this study examined other potential markers, including the genes such as *JHAMT, ilp1, ilp2, TOR1*, and *HSP70* genes were examined in this study. The enzyme involved in JH biosynthesis, *Juvenile Hormone Acid Methyltransferase (JHAMT),* exhibits changes in expression direction similar to JH titer and can serve as an alternative marker to JH [45, 46, 47](Goodman and Granger, 2005; Li et al., 2013; Egi and Sakamoto, 2022). A positive correlation with JH was observed in the *insulin-like peptide, ilp1,* whose expression increases in the brains of foragers and decreases with during continued foraging [48](Corona et al., 2007; Ament et al., 2008). The *insulin-like peptide, ilp2* gene is expected to have task-related expression as it is highly expressed in nurse tissue and plays a role in

Formatted: Highlight
Formatted: Highlight
Formatted: Highlight
Formatted: Highlight
Formatted: Highlight
Formatted: Font: Not Bold
Formatted: Highlight
Formatted: Font: Italic
Formatted: Font: Italic
Formatted: Font: Not Bold
Formatted: Highlight
Formatted: Highlight
Formatted: Highlight

increasing cell size and number in individual organs [49] ~~(Zhang et al., 2022)~~. Additionally, the *TOR1* gene, a nutrient-sensing kinase, along with the *ilp1* and *ilp2* genes, participates in the mobilization of nutritional resources (autophagy) from tissues such as the fat body and glands in *Drosophila* [50]~~(Barth et al., 2010)~~. Moreover, the *TOR* pathway is implicated in disease and nutrition in insects [51, 52, 53]~~(Patel et al., 2007; Laplante and Sabatini, 2012; Johnson et al., 2013)~~, and mammals [54]~~(Zoncu et al., 2011)~~, and it also regulates longevity extension in social insects [25]~~(Rodrigues and Flatt, 2016)~~. Lastly, the stress marker *heat shock protein 70 (HSP70)* was chosen because~~as~~ it is detected at low levels in nurses but shows increased expression in foragers [55]~~(Sario˘glu-Bozkurt et al., 2022)~~. While all these markers are expected to be important for identifying aging in honey bees, testing them together requires further investigation.

This research aimed to simulate the influence of climate warming on colony performance in early fall and to compare the aging of overwintered *Apis mellifera ligustica* honey bees. The experiment involved~~considered~~ maintaining colonies at ambient temperatures of 25°C (Group 25) and 35°C (Group 35) in controlled rooms, representing ~~, which simulated~~ the average and high summer temperatures in Incheon (Republic of Korea), respectively. A~~, while~~ Group control was kept under natural conditions ~~were maintained in the Group control~~. The temperature experiments continued~~lasted~~ throughout the last~~previous~~ summer, September, and October, with

Formatted: Highlight

Formatted: Highlight

Formatted: Font: Not Bold

Formatted: Font: Not Bold

Formatted: Highlight

Formatted: Font: Not Bold

Formatted: Highlight

Formatted: Highlight

Formatted: Font: Not Bold

Formatted: Highlight

the , with the colonies overwinteringinging under the same conditions. CColony performance, including the brood microclimate, was assessed in August and September, during the transition , was investigated in August and September because that month honey bee transformation from summer to winter physiology in the Republic of Korea started in the Republic of Korea. In February, we evaluated Tthe aging of the bees of honey bees at the end of of diapause diapause (broodless period) using eight markers: in February was determined by eight markers, including two physiological markers and six molecular gene markers. The physiological markers were (aAcini areaus size and mass of lipid content in the fFat body. The molecular markers were for: weight) and six molecular gene markers related to the tasks (*vg* and *JHAMT*), nutrition (*ilp1, ilp2*, and *TOR1*), and stress response (*HSP70*). By linking climate change factors, such as fall temperature, to honey bee responses in winter, this research can inform conservation actions for beekeepers to prevent bee losses. We believe this study can lead to further research and the development of a test kit to identify the causes of aging early.

Methods

Formatted: Font: 18 pt

Experimental bees

Western honey bees (*Apis mellifera ligustica*) were obtained from nine colonies kept at the Incheon National University apiary in the Republic of Korea **(Fig 1A, B, C, and D),** managed by a professional beekeeper following standard beekeeping practices

Formatted: Indent: First line: 0 ch, Add space between paragraphs of the same style

[56]. The experimental colonies housed first-year young sister queens, naturally mated and beginning egg-laying in May 2022. Prior to the experiment in June, colonies were standardized in terms of the number of frames (total 4), brood population (3 frames), honey amount (1 frame), and the number of honey bees. Colony strength was assessed through visual inspection of adult and brood populations both before and during the experiment [56]. Two rooms equipped with a climate-control system were established to maintain honey bee colonies at 25°C (Group 25) and 35°C (Group 35), simulating climate warming without day and night temperature differences.

Formatted: Font: Not Bold

Formatted: Left

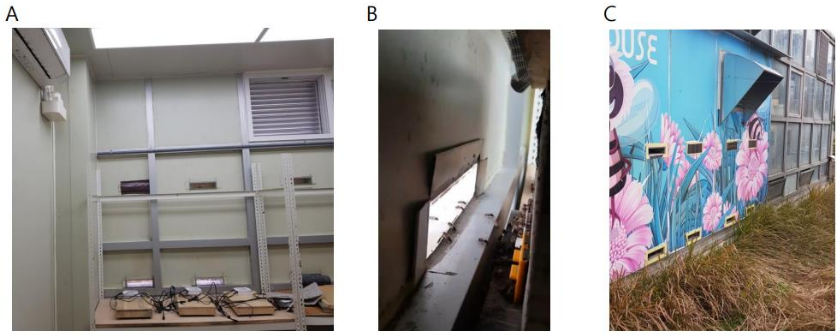

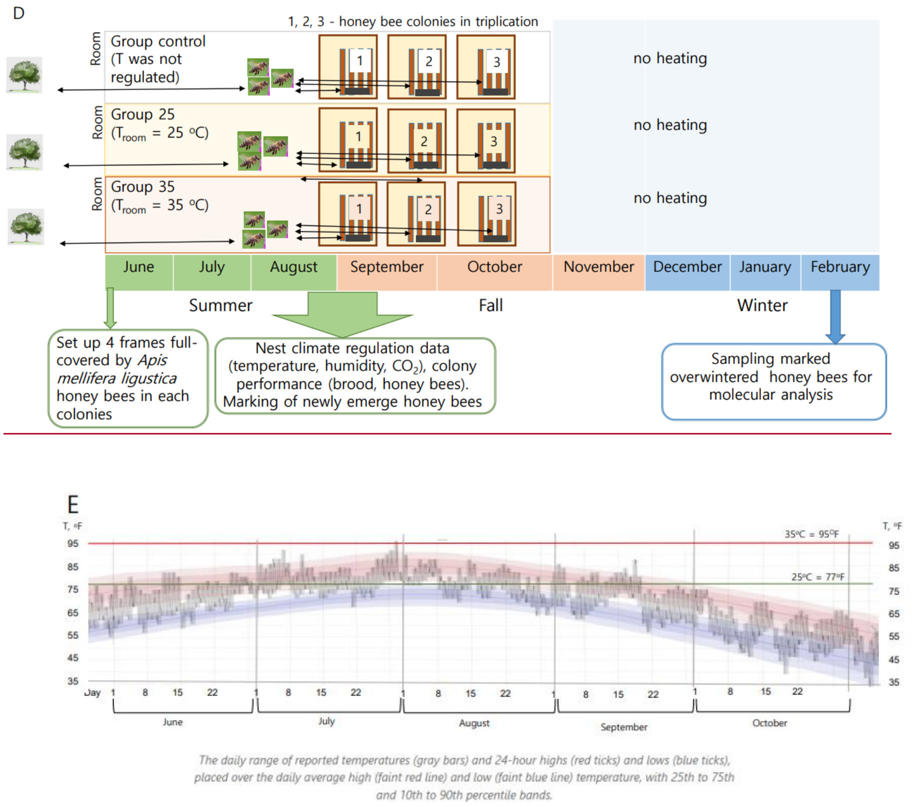

Fig 1. Overview of the experimental design (A) The room environment before the experiment. **(B)** The process of honey bees freely flying outside from the experimental room. **(C)** The view of the room and the entrance for honey bees outside. **(D)** Timeline of the experiment. **(E)** Ambient temperature of the Group control, Group 25 (green line), and Group 35 (red line) under the temperature experiment in 2022, Incheon, Republic of Korea. The data were obtained from https://weatherspark.com/h/m/142040/2022/10/Historical-Weather-in-October-2022-in-Incheon-South-Korea#Figures-Temperature.

These temperatures were selected to mimic the effects of climate warming, with 25°C representing the summer average and 35°C being the highest recorded in

Formatted: Left

Incheon city under typical conditions. However, the highest temperature was not naturally reached during the summer of 2022 **(Fig 1E).** Other environmental conditions were not controlled. Light was used during colony inspections. In the third room, housing honey bee colonies, the control group was provided with free ventilation without additional heating. All honey bees had equal opportunities to collect pollen and nectar around the apiary.

On September 18th and 25th, 2022, two combs with sealed brood were placed in an incubator set at 34°C. Upon hatching, the workers were individually marked with colored paint on their thoraxes and then reintroduced into their original colony. Throughout the previous summer, as well as in September and October of 2022, the honey bee colonies were subjected to varying room ambient temperatures. Afterward, the controlled temperatures in the rooms were turned off, and the bee colonies were overwintered successfully (**S1 Table**) in the same rooms under natural conditions.

Formatted: Font: Bold

The overwintered marked honey bees, aged approximately 4.5 months, were sampled on February 5th, 2023, a time when queens had not yet begun laying eggs and the overwintered bees were expected to have successfully survived the winter. Forty honey bees from each colony were sampled and individually placed in labeled 50 ml tubes, then stored at -80°C before analysis. The success of colonies overwintering was monitored on February 5th and 27th, when we recorded whether the honey bees survived (+) or did not survive (-) (**S1 Table).**

Formatted: Font: Bold

Brood microclimate measurements

The temperature, humidity (AM2320, BeeOnFarm, Korea), and CO_2 concentration (BO-100, BeeOnFarm, Korea) sensor systems were placed on the top and middle of each colony in August 2022. The data logging system was included in the sensor system because it provided a real-time clock. Data consisting of time (seconds), CO_2 (in ppm), temperature (in °C), and relative humidity (as a percentage) were saved, with approximately three measurements in minutes to approximately one file per day **(S1 File)**. The data from 6 honey bee colonies kept in each room (three at 25°C and three at 35°C) were collected over 15 days from 11 to 25 August. Average values were used for statistical calculations.

Formatted: Font: Bold

Hypopharyngeal gland (HG) measurements

Areas of ninety acini from the hypopharyngeal glands of overwintered honey bees in each Group (control, 25°C, and 35°C) were measured to determine if there were differences based on the ambient temperature during the previous fall season. Thirty bees were collected from each colony, flash frozen in liquid nitrogen, and maintained at −80°C until their glands were measured **(S2 File)**. For each bee, the HGs were dissected into 1× phosphate-buffered saline (PBS) with 0.1% Tween® 20 and visualized at 40× magnification. The area (mm^2) of a minimum of ten randomly selected acini per bee was measured using Nikon Eclipse E200 (Nikon, Tokyo, Japan) Toupview (Touptek, Hangzhou, China) software. Only acini with clear borders were

Formatted: Font: Bold

used. Acini areas were averaged within individuals.

Fat body lipid content

The fat body lipid content in the abdomen of overwintered honey bees was studied in early February by the standard method [57, 58]. Ten honey bees were collected from each colony; ninety honey bees were flash frozen in liquid nitrogen and maintained at −80°C until their abdomens were dissected and their guts were removed. Pools of ten abdomens were weighed on a scale (HS220S, HANSANG Instrument Co., Ltd., Republic of Korea) before and after drying to a constant weight in a fume hood (SSFH-1000, Shinsaeng, Republic of Korea). The total lipid content was determined by measuring the decrease in weight following the removal of lipids from the dried tissue through extraction in 5 ml portions of diethyl ether (179272-1 L; Sigma–Aldrich, USA) **(S2 File).**

Formatted: Font: Not Bold

Formatted: Font: Not Bold

Formatted: Font: Bold

RNA Extraction and cDNA Synthesis

A total of ninety honey bee (ten winter bees from each colony) *Apis mellifera ligustica* colonies were sampled in labeled tubes and stored at -80°C. Three honey bees from each colony were analyzed. The brain and gut-free abdomen of honey bees were dissected on ice, and RNA was extracted directly. RNA was extracted from the brain for the analysis of *HSP70, JHAMT, ilp1, ilp2*, and *TOR1* expression, and from the abdomen for *vg* gene expression analysis, as this gene is expressed in the fat body located in the abdomen. Genes are measured in the brain to understand their roles in

stress responses (*HSP70*), metabolic regulation (*JHAMT* and *TOR*), and neural function (*ilp1* and *ilp2*).

Total tissue RNA (brain or abdomen) was extracted from fifteen randomly selected bees in each group using a Qiagen RNeasy Mini Kit (#74,104; Qiagen, Valencia, CA, USA). The tissues in each replicate were brain tissue for *TOR1, ilp1, ilp2, JHAMT*, and *HSP70* and abdomen for *vg* gene expression. The total RNA concentration and purity were quantified using OD260/OD280 values between 1.8 and 2.0. Next, reverse transcription was performed using an RNA to cDNA EcoDryTM Premix (Oligo dT) kit (Takara, Japan). The reverse transcription reaction mixture included 50 ng/µl total RNA (with the clear volume calculated for each sample) and RNase-free water for a total volume of 20 µl. Reverse transcription was conducted at 42°C for 60 min, followed by heating at 70°C for 10 min.

Formatted: Left, Indent: First line: 1 ch

Quantitative real-time PCR

Formatted: Left

The relative expression of six genes related to nutrition (*vg, ilp1, ilp2, TOR1),* development *(JHAMT),* and stress response (*HSP70)* was measured. The housekeeping gene *RpL32* (Ribosomal protein L32*)* was used as an endogenous control. The PCR primer sequences are shown in **S2 Table.**

The reaction conditions were optimized. The qRT–PCR mixture had a volume of 20 µl which included 2 µl of template cDNA, 10 µl of Green qPCR Master Mix, 1 µl of upstream and downstream primers (5 pM/µl), and 6 µl of nuclease-free water

(AM9930, Invitrogen, USA). Quantitative real-time PCR (qRT–PCR) was conducted using Brilliant III Ultra-Fast SYBR® Green qPCR Master Mix (600882, Agilent Technologies, USA) on an AriaMx Real-Time PCR System (Agilent Technologies LDA, Malaysia) with AriaMx 96 Well Plates, Skirted, LP (401490, Agilent Technologies, USA) covered by MicroAmp™ Optical Adhesive Film (4311971, Thermo Fisher Scientific, USA).

The qRT–PCR amplification procedure was as follows: initial denaturation at 95°C for 10 minutes; 40 cycles of denaturation at 95°C for 30 seconds, annealing at 60°C for 25 seconds, and extension at 72°C for 15 seconds. Each sample was technically replicated three times. Data analysis was performed using Agilent AriaMx version 2.0 analysis software. Relative gene expression data were analyzed using the 2^(-Delta Delta C(T)) method [59, 60] (**S3 File**).

Formatted: Left, Indent: First line: 1 ch

Formatted: Font: Not Bold

Formatted: Font: Bold

Confirmation of amplicons in the RT–PCR products was performed by separating them through electrophoresis in a 2% agarose gel at 80 V for 40 minutes and analyzing them using a gel documentation system, the Gerix 1010 transilluminator (Biostep GmbH, USA) (**S2 Fig**). A Dyne 50 bp DNA Ladder (Cat. No. A701, DYNEBIO, Korea) was used as a reference.

Formatted: Left, Indent: First line: 1.5 ch

Formatted: Font: Bold

Statistical analysis

Formatted: Left

The statistical analysis, designed as illustrated in **Fig 2**, was conducted using Microsoft Excel and XLSTAT software (Addinsoft Pearson Edition 2014, Addinsoft,

Paris, France). The characterization was performed using the following strategies [61]: (a) classification of the marker dataset to find a combination of features that best separates different groups (supervised approach, Discriminant Analysis (DA)); (b) clustering to discover natural groupings in the absence of labeled information (unsupervised approach, Agglomerative Hierarchical Clustering (AHC)); and (c) modeling the relationship between independent variables and a dependent variable and dealing with multicollinearity in the marker dataset to rank the markers by importance for identifying overwintered honey bee task development (supervised approach, Elastic Net Regression).

Specifically, the honey bees were categorized into groups, and the variables with the highest discrimination scores were selected using DA because it determines the linear combination of variables and provides maximal separation between groups. To discriminate the groups, Fisher distances were applied, which emphasize the linear combinations of variables that maximize the differences between groups.

AHC analysis provided a visual representation of the clustered dataset, revealing the arrangement of individual data points or observations based on their dissimilarity. The evaluation metric for clustering algorithms was the silhouette index. The score is bounded between (− 1) for incorrect clustering and (+ 1) for highly dense clustering.

Apis mellifera ligustica **honey bees**

Month of sampling		Group control (3 colonies)	Group 25 (3 colonies)	Group 35 (3 colonies)
Experimental condition (summer, September, and October)			T_{room} = 25 °C	T_{room} = 35 °C
**August September**	Data set 1 (brood microclimate and colony development)		Nest climate regulation	
	Statistics of data set 1		T-test ($p < 0.05$)	
**February**	Sampling of honey bees, n	(for physiology markers): 30+30+30<br>(for molecular markers): 10+10+10	30+30+30<br>10+10+10	30+30+30<br>10+10+10
	Data set 2	Two physiology markers (hypopharengial glands (HG, acini area) and fat body mass)<br>Molecular marker genes related to nutrition (*vg*, *ilp1*, *ilp2*, *TOR1*), development *(JHAMT)*, and stress response (*HSP70*).		
	Statistics of data set 2	1. Discriminant analysis (define the variables with high discrimination score);<br>2. Agglomerative Hierarchical Clustering (check the natural groupings within dataset);<br>3. Elastic Net Regression (model the relationship between HG (acini area) and fat body mass with molecular markers);<br>4. ANOVA, Duncan's post-hoc test (comparison of significance within each variable, $p < 0.05$).		

Fig 2. Experimental conditions, sampling schedule, data sets, and statistical tests used for analysis.

Elastic Net Regression analysis was used to test two models of the relationship [61, 62] between physiology and molecular markers. The dependent variables were acini size and fat body mass. The seven independent variables were ranked by coefficient. Receiver operating characteristic (ROC) curves were generated to quantify how accurately regression analysis can discriminate between honey bee groups [63]. The ROC specificity and sensitivity plot demonstrated a perfectly fitted model linking physiology to molecular markers (gene expression profile) (**S1 Fig**). The area under the curve (AUC) can range from 0.5 to 1.0, with a preference for higher scores.

The mean, standard deviation, and variance of each gene were calculated using descriptive statistics and visualized using the heat map module. For the gene expression analysis, ANOVA was used to test overall effects, followed by the Duncan's post hoc test ($p < 0.05$) for multiple comparisons and the t-test for

Formatted: Left

Formatted: Font: Bold

differences of means between two groups of honey bees (**S4 File**). The bar plots were generated using the online SRplot Official Website (https://www.bioinformatics.com.cn/en; accessed on 30 October 2023).

Our findings suggest that temperature decreases around winter worker broods from Group 25 in the fall lead to their slower physiological aging in winter compared to Group 35 workers, supported by physiology and task-related markers. Furthermore, we observed a relation between aging and *HSP70* and *TOR1* genes, which may serve as additional markers, necessitating further research. This approach could enhance the molecular method for identifying aging in insect models, thereby advancing research related to climate warming.

Results

The experiment was conducted on nine colonies of western honey bees (*Apis mellifera ligustica*). Each Group (control, 25°C, and 35°C) was represented by three colonies. The bees from the Groups 25 and 35 experienced stable, controlled day and night ambient temperatures (modeling climate warming) from June to October, while the

Formatted: Font: Bold

Formatted: Font: 18 pt

Formatted: Left, Indent: First line: 1 ch

Formatted: Left

Formatted: Font: 14 pt, Not Bold

Formatted: Font: 14 pt, Not Bold, Italic

Formatted: Font: 14 pt, Not Bold

Formatted: Font: 14 pt, Not Bold

Formatted: Font: 14 pt

Formatted: Font: 14 pt, Not Bold

room for the Group control was freely ventilated. All bees experienced natural foraging outside without restrictions. Newly emerged honey bees were marked in September for the overwintering experiment in February.

Dynamics of ambient temperature in summer and fall months

The daily temperature in the first experimental room was set at 25°C for five months, and there were no significant differences compared to the natural daily average temperature in the summer months or September (ANOVA, Duncan's post hoc tests, $p > 0.05$). The difference in experimental conditions for the honey bees from the Group control and the Group 25 was due to day-night temperature fluctuations, which were absent in the latter Group. Although significant temperature differences were not detected, a stable elevated night temperature is one of the factors of climate warming that influences bees. However, it was significantly greater than the average daily temperature in October 2022 (same tests, $p < 0.05$). The daily temperature in the second room was set at 35°C for five months, which was significantly greater than the average daily temperature during the summer months, September, and October (same tests, $p < 0.05$) ~~(Fig 6E)~~.

Brood microclimate and colony development in August and September

In this study, we evaluated the performance of the colony using temperature, humidity,

Formatted: Font: 14 pt

Formatted: Font: 14 pt, Not Bold

Formatted: Font: 18 pt

Formatted: Font: 16 pt

Formatted: Font: 16 pt

CO_2, number of larvae, capped brood area, and honey bees' measurements from August 08 to September 18, 2022, when short-lived ~~honey~~ bees commenced rearing larvae that would develop into long-lived ~~honey~~ bees for the overwintering period. Since the expected ambient temperature in August and early September and the comparison of the colony performance of the Groups control and 25 on August 08 (**Fig 3 D, E, F**) showed no significant differences, sensors were not set up in the Group control. –We hypothesized that the regulation of the nest climate inside colonies influences the physiology of winter ~~honey~~ bees and the success of overwintering.

Formatted: Font: (Default) Times New Roman, 14 pt

Formatted: Font: (Default) Times New Roman, 14 pt

Formatted: Font: Bold

Formatted: Font: (Default) Times New Roman, 14 pt

Formatted: Font: (Default) Times New Roman, 14 pt

Formatted: Suppress line numbers

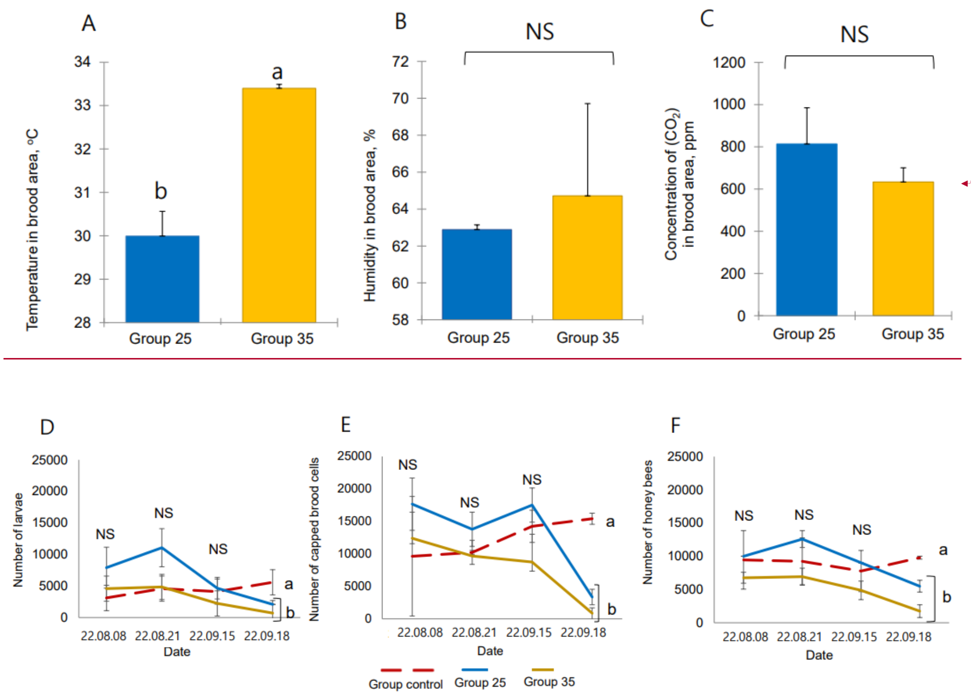

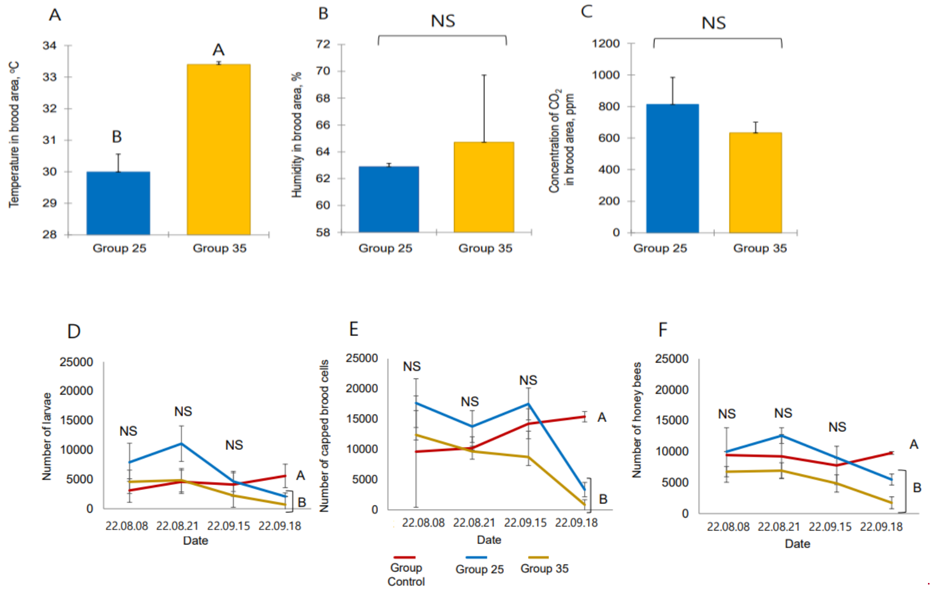

Fig 3~~1~~. Colony performance in honey bees treated under different ambient temperatures (08 August – 18 September 2022). (A) Temperature in the brood area (°C). **(B)** Humidity in the brood area (%). **(C)** Concentration of CO_2 in the brood area (ppm). **(D)** Number of larvae. **(E)** Number of capped brood cells. **(F)** Number of honey bees. Statistical significance was determined using a t-test in **Fig 3A, B**, and **C** ($p < 0.05$, indicated by different letters above the columns) to compare the means of the two groups, one-way ANOVA, ~~and Duncan~~Duncan's post hoc tests ($p < 0.05$, indicated by different letters above the columns) to compare the means of the three groups in **Fig 3D, E**, and **F**; bars in the graphs represent the mean ± SD.

SD – Standard Deviation, NS – No Significance. Honey bee colonies in Group 25 were maintained in room conditions at 25°C; colonies in Group 35 were kept in room conditions at 35°C, while colonies from Group c~~C~~ontrol ~~colonies~~ were maintained under natural conditions.

The mean and standard deviation of the ~~nest~~brood temperature for ~~honey~~ bee

Formatted: Add space between paragraphs of the same style, Line spacing: Double

Formatted: Font: Bold

Formatted: Font: Bold

Formatted: Font: Bold

Formatted: Font: Bold

Formatted: Left

colonies kept at 25°C and 35°C were 30.0029.995 ± 0.982 and 33.40 398 ± 0.154 °C, respectively. Similarly, the nest humidities for these groups were 620.00.889 ± 0.435 and 64.7172 ± 8.6500 %, and the nest CO_2 concentrations were 813.553 ± 296.841 and 633.29830 ± 1167.0959 ppm, respectively. Importantly, the nest temperature inside the honey bee colonies kept at 35°C was significantly greater than that inside the honey bee colonies kept at 25°C (t-test, $p < 0.05$) (**Fig 31A**). However, nest humidity, CO_2 concentration, number of larvae, capped brood cells, and honey bees were not significantly differing (**Fig 3B, C**: t-test, $p > 0.05$) (**Fig 31B, C, D, E, and F**: ANOVA, Duncan's post hoc tests $p > 0.05$). The colonies in the Group control, however, exhibited significantly (ANOVA, Duncan's post hoc tests, $p < 0.05$) larger capped brood area, more larvae, and a higher number of honey bees on September 18 compared to colonies in Groups 25 and 35. This observation suggests the importance of day-night and seasonal temperature fluctuations in the fall for colony development, especially as the colony prepares for overwintering.

Formatted: Font: Bold

Formatted: Font: Bold

Formatted: Font: Not Bold

Analysis of the honey bee groups and the relationship between the markers

Formatted: Font: 16 pt

Molecular marker recognition in overwintered western honey bees

The relationship between –molecular markers andrecognition related to the biological age of long-lived honey bees, which were approximately 4.5 months old, of overwintered honey bees was analyzedverified using through _a dataset comprising

two established physiological markers (a~~A~~cin~~ius~~ area~~size~~ and mass of lipid content in the f~~F~~at body~~ weight~~) and six molecular markers (*vg, TOR1, JHAMT*, *ilp1, ilp2,* and *HSP70*). ~~using~~ Data Mining methods, including Discriminant Analysis (DA), Agglomerative Hierarchical Clustering (AHC), and Elastic Net Regression, were employed in the analysis~~-~~.

DA was performed to plot the ~~honey bee~~ Groups control, 25 and 35, and to identify the optimal combination of features that effectively distinguishes them. The Wilcoxon Lambda test within the DA yielded a statistically significant result (two-tailed test, $p < 0.05$), indicating that the model successfully differentiated between groups (**Fig 4~~2~~A**). The observed Wilks' lambda value of 0.000 signifies a robust separation of the Groups control, 25, and 35, demonstrating a high level of discriminative power. A comparison revealed significant differences between Group 25 and Group 35 ($F(2,8) = 19.37$;~~,~~ two-tailed test, $p < 0.05$; **Fig 4~~2~~A**).

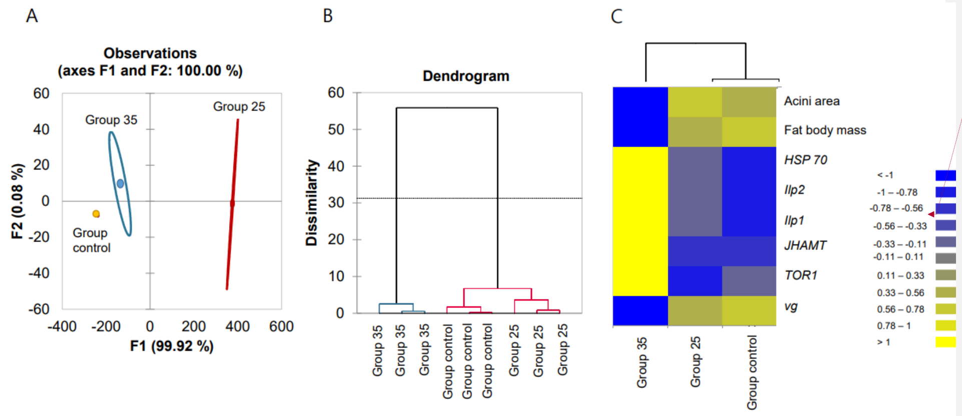

Formatted: Suppress line numbers

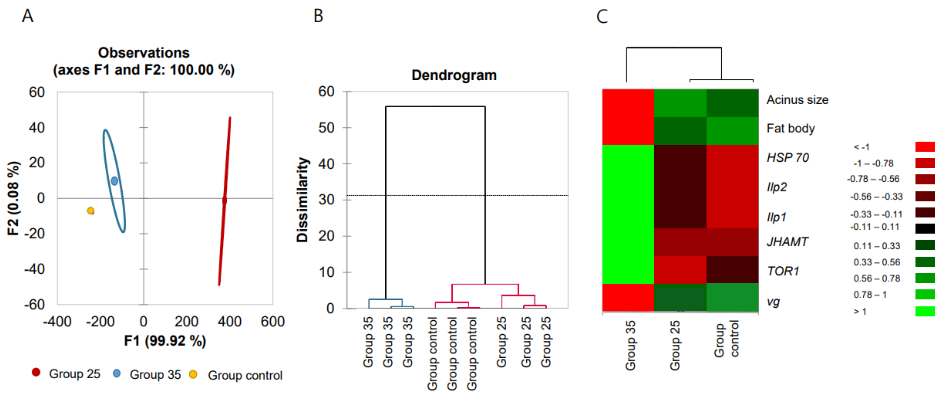

Fig 42. The discrimination of overwintered long-lived honey bees at the end of winter.was performed using eight variables related to aging: two physiological variables (acini area, n = 30; mass of lipid content in the fat body, n = 10) and six molecular variables (n = 10), including the expression levels of *HSP70, ilp1, ilp2, JHAMT, TOR1*, and *vg* genes.

(A) Discriminant Analysis (DA). **(B)** Agglomerative Hierarchical Clustering (AHC). **(C)** Heatmap with cluster analysis based on ddCt values, aAcinusi areasize (mm²), and the Ffat body weightmass (mg). Honey bee colonies in Group 25 were maintained in room conditions at 25°C; colonies in Group 35 were kept in room conditions at 35°C, while Group Control colonies were maintained under natural conditions.

The Fisher distances (FDs) in the DA between the Groups Ccontrol, Group 25, and Group 35 honey bees were significantly different (FD = 27511.10; $p < 0.05$ and FD = 19131.16; $p < 0.05$, respectively). Moreover, the FD density was also significantly different between the honey bees from Group control and Group 25 (FD = 783.659; $p < 0.05$). The honey bees were separated along the primary discriminant axis F1 in

Formatted: Add space between paragraphs of the same style, Line spacing: Double

Formatted: Font: Italic

Formatted: Font: Italic

Formatted: Font: Italic

Formatted: Left

99.928% accuracyof the samples, where discriminant function coefficients for indicating that the gene expressions of *vg, HSP70*, and *JHAMT* were -20.092, -14.960, and 23.576, respectively. These coefficients were the highest, providing significantly contributoinsed to this separation.

Subsequently, AHC was employed to identify natural groupings without labeled information using a dissimilarity measure. The evaluation metric for clustering algorithms, the Silhouette index between clusters 1 and 2, was 0.657, indicating successful clustering, albeit with moderate density **(Fig 24B, C).**

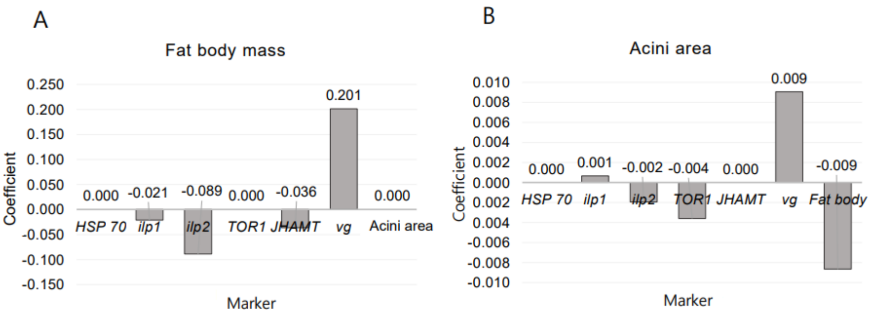

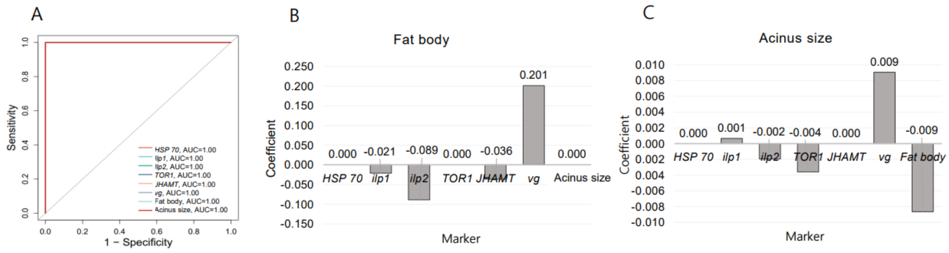

Fig 5~~3~~. Elastic Net Regression modeling the relationship beween ~~for the~~ dependent variables fat body mass (mass– of lipid content in the f~~F~~at body, A) and– a~~A~~cini~~us~~ area ~~size~~ (B) ~~,~~ and the independent variables (*HSP70, ilp1, ilp2, TOR1, JHAMT, vg*, and acini area or fat body mass respectively) to rank the variables by importance for predicting dependent variables. ~~(A) The quality verification plot (specificity *vs* sensitivity) demonstrated a perfectly fitted model linking physiology to molecular markers (gene expression profile). (B) The regression model of predictor importance and ranking for Fat body weight. (C) The regression model of predictor importance and ranking for Acinus size.~~

Finally, Elastic Net Regression was used to rank the markers by importance by modeling the relationships between seven independent variables and one dependent variable **(Fig 5~~3~~A, B~~, C~~).** This analysis was conducted separately for ~~F~~fat body mass **(Fig 5~~3~~A~~B~~)** and a~~A~~cini area~~us size~~ **(Fig 5~~3C~~B).** The f~~F~~at body mass model identified significant predictors, with variable *vg* having the highest positive coefficient (0.201) and variable *ilp2* having the highest negative coefficient (-0.089). In the a~~A~~cini area~~us size~~ model, significant predictors were identified, with the variable *vg* having the highest positive coefficient (0.009) and the variable f~~F~~at body mass having the highest negative coefficient (-0.009). In this both models, only two genes — *vg* and *ilp2* — showed high positive and negative coefficient values, respectively.

Comparative analysis of the biological age of overwintered western honey bees through physiology markers

These are the results ~~comparative biological age of the overwintered honey bees was evaluated~~ based on the ~~molecular and~~ two physiological markers, analysis of the mass of lipid content in the fat body (fat body mass) and the ~~. The latter, included in this~~

Formatted: Font: Bold
Formatted: Font: Bold
Formatted: Font: Bold
Formatted: Font: Bold, Italic
Formatted: Font: Bold
Formatted: Font: Bold
Formatted: Font: Bold
Formatted: Font: Bold
Formatted: Font: Bold
Formatted: Left
Formatted: Font: Italic
Formatted: Font: Italic
Formatted: Font: 16 pt

results, consisted of the aAcinius area size fromof the hypopharyngeal glands (HGs) and analysis of Fat body weight. The mean and standard deviation of the fFat body massweight for the honey bees in the Groups control, 25, and 35 were 4.33 ± 1.247, 4.000 ± 1.633, and 2.000 ± 0.816 mg/bee, respectively, with no significant differences (ANOVA, Duncan's post hoc, P = 0.222, $p < 0.05$), even though the fFat body weightmass of the honey beesthem in Group 35 was slightly lower **(Fig 64A).**

Similarly, the mean and standard deviation of the sizearea of the honey bee Aacinius from the Groups control, 25, and 35 were 0.026 ± 0.001, 0.027 ± 0.003, and 0.018 ± 0.001 mm^2, respectively. Notably, the aAcinius areasize of the honey bee in Group 35 was significantly lower (same test, P = 0.006, $p < 0.05$) than that inof the Group control and 25 **(Fig 46B).** Importantly, both the fFat body weightmass and the aAcinius area size of the overwintered honey bees in Group 35 were lower than those of the overwintered honey bees in Groups control and 25 **(Fig 64)**, suggesting lower nutrition and accelerated physiologicaly aging inof honey bees from Group 35.

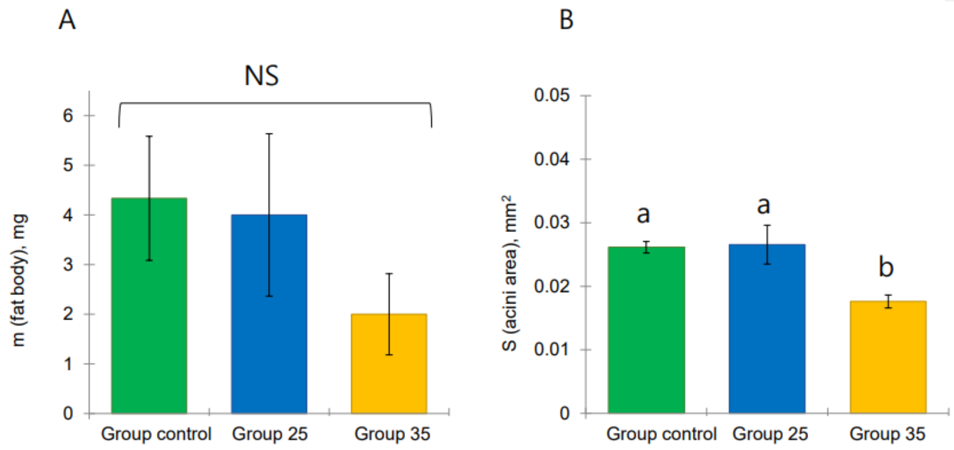

Formatted: Centered

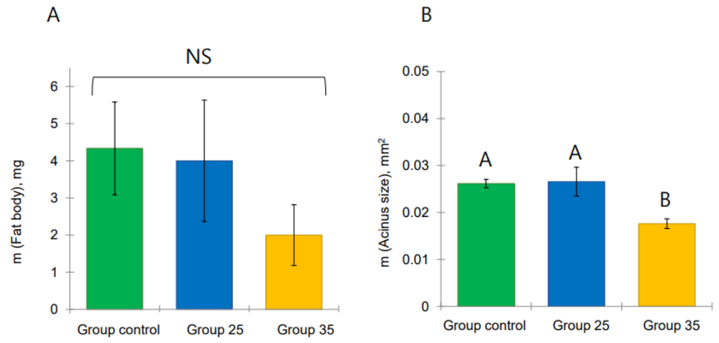

Fig 64. Physiological markers of long-lived honey bees at the end of winter (February) (A) The average of fat body mass (mass of lipid content in the fat body), n = 10 (mean ± SD). Average Acinus size, n > 200 (mean ± SD). **(B)** Average acini area, n = 30 (mean ± SD). Average Fat body weight, n = 10 (mean ± SD). Statistical significance was determined using one-way ANOVA, and DuncanDuncan's post hoc tests (*p* < 0.05, indicated by different letters above the columns).

Formatted: Add space between paragraphs of the same style, Line spacing: Double

SD - Standard Deviation. NS - Not Significant. Honey bee colonies in Group 25 were maintained in room conditions at 25°C; colonies in Group 35 were kept in room conditions at 35°C, while Group

~~Control colonies were maintained under natural conditions.~~

Comparative analysis of the biological age of overwintered western honey bees through molecular markers

These are the results ~~of the comparative biological age of the overwintered honey bees~~ based on molecular markers. They include two previously defined task-related markers, *vg* and *JHAMT,* as well as four genes expected to respond to aging: *TOR1, ilp1, ilp2*, and *HSP70* **(Fig. 7~~5~~).**

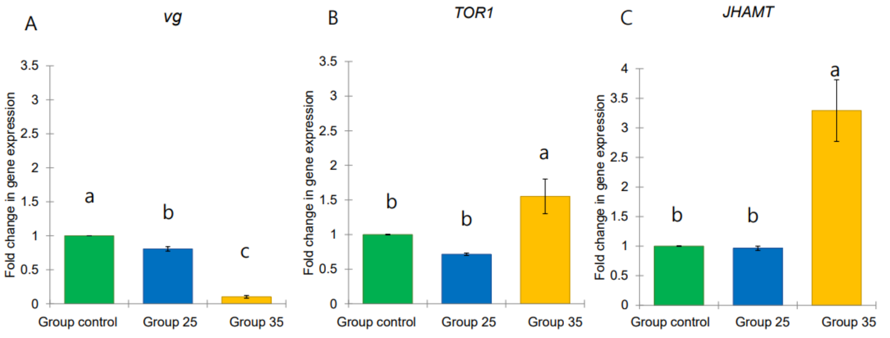

Formatted: Font: 14 pt

Formatted: Left, Add space between paragraphs of the same style, Line spacing: Double

Formatted: Font: 16 pt

Formatted: Left

Formatted: Left, Indent: First line: 1 ch

Formatted: Left

Formatted: Suppress line numbers

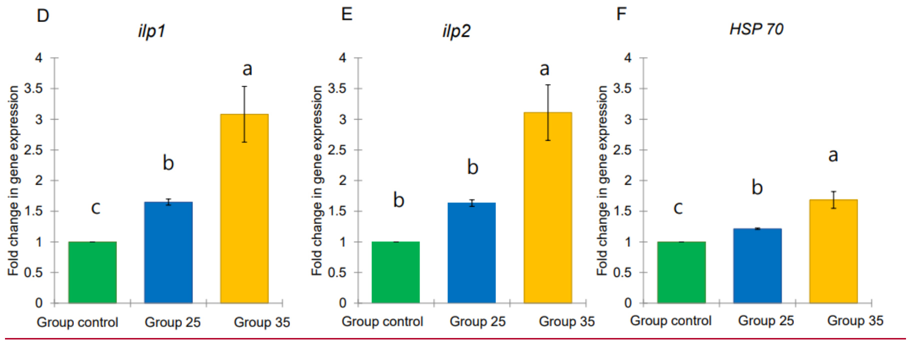

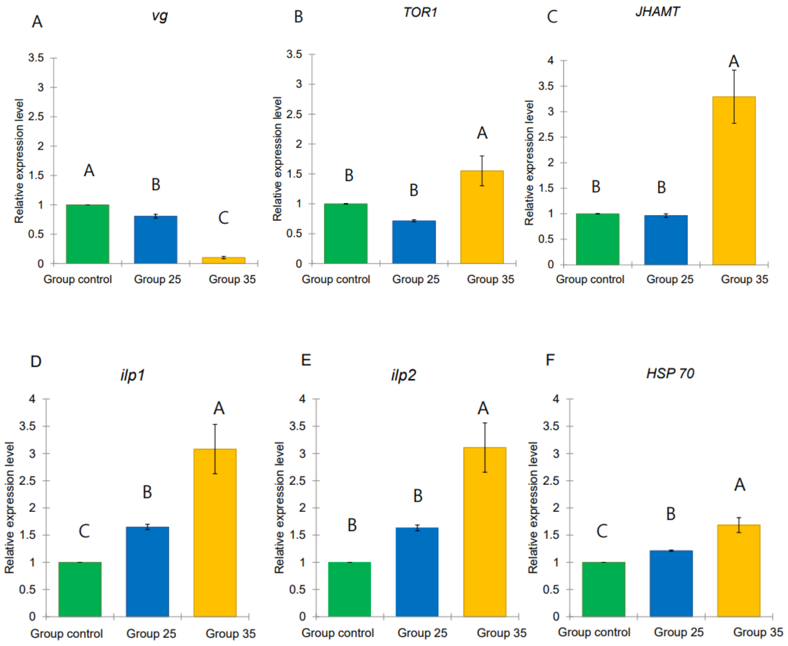

Fig 75. Gene expression analysis. The gene expression (ddCt) of *vg** **(A),** *TOR1*** **(B),** *JHAMT*** **(C),** *ilp1*** **(D),** *ilp2*** **(E),** and *HSP70*** **(F)** in long-lived honey bees at the end of overwintering (February) was evaluated. Statistical significance was determined using one-way ANOVA, and DuncanDuncan's post hoc tests ($p < 0.05$, indicated by different lowercase letters above the columns).

Formatted: Add space between paragraphs of the same style, Line spacing: Double

The bars in the graphs represent the mean ± SD. ~~SD – Standard Deviation.~~ *n=9. **n=15.

~~Honey bee colonies in Group 25 were maintained in room conditions at 25°C; colonies in Group 35 were kept in room conditions at 35°C, while Group Control colonies were maintained under natural conditions.~~

Expression of *vg* gene was significantly upregulated (ANOVA, ~~Duncan~~Duncan's post-hoc test, $p < 0.05$) in ~~honey~~ bees from Group control and 25 compared to those~~honey bees~~ from Group 35. In contrast, the expression of the *TOR1, JHAMT, ilp1, ilp2*, and *HSP70* genes was significantly downregulated (same test~~ANOVA, Duncan post-hoc test~~, $p < 0.05$) in the same ~~honey~~ bees, suggesting aging in bees~~of honey bees~~ from Group 35 compared to ~~honey bees from~~ Groups control and 25 **(Fig. 7~~5~~B, C, D, E, and F)**. However, there were no significant differences in the expression of *TOR1, JHAMT*, and *ilp2* (same test~~ANOVA, Duncan post-hoc test~~, $p > 0.05$) between ~~honey~~ bees from Group control and Group 25. The expression levels of *ilp1* and *HSP70* were significantly lower (same test~~ANOVA, Duncan post-hoc test~~, $p < 0.05$) in the ~~honey bees~~ ones from Group control than in Group 25.

Formatted: Left

Formatted: Font: 18 pt

Discussion

Key triggers influencing the transition of insects from summer to winter physiology include photoperiod, ambient temperature, brood microclimate, availability of pollen,

Formatted: Left

and some yet-to-be-identified factors [25, 64, 65]~~(Knoll et al., 2020; Rodrigues and Flatt, 2016; Quinlan et al., 2023)~~, which obviously have a particular influence on aging and subsequent winter loss in honey bees. However, due to the complex and unclear interactions among all these triggers, we designed an experiment in which only one trigger of climate warming, ambient temperature (25, 35°C, and control), was manipulated during the previous summer, September, and October. Colony development and nest climate regulation were investigated in August and September, during which long-lived (winter) ~~honey~~ bees were reared and marked to study the biological age of honey bees alongside their clear chronological age in February. Aging was assessed using two physiological markers (a~~A~~cini~~us~~ area~~ size~~ and mass of lipid content in the f~~F~~at body~~ weight~~) and six molecular markers (*vg, TOR, JHAMT, ilp1, ilp2*, and *HSP70*) at the beginning of February, during the expected diapause continuation.

It was found that prolonged climate warming significantly weakened colony development in Groups 25 and 35 during the fall season (as of September 18) compared to the control group. This observation supports the importance of day-night and seasonal temperature fluctuations for natural colony development [66], as other colony parameters were the same.

This colony development, as a response to ambient temperature, is closely related to honey bees' regulation of the nest microclimate. A temperature difference of 10°C between Groups 25 and 35 was assessed to study this. However, in August and

Formatted: Font: Not Bold

Formatted: Highlight

Formatted: Font: Not Bold

September, bees adjusted this brood temperature difference to 3.403°C (**Fig. 3**). To achieve this, honey bees in Group 25 heated the brood area to a subnormal temperature of 30.00 ± 0.982 °C, while those in Group 35 cooled it to 33.398 ± 0.154 °C, which is slightly lower than the typical nest temperature of 34.5 ± 1.5 °C [67]. This well demonstrated known microclimate regulation [21, 22]. Although we don't know if the number of bees in colonies from Groups 25 or 35 may not have been sufficient to heat or cool more, nor if the subnormal brood temperature in Groups 25 and 35 may be a mechanism to rear long-lived winter bees, these brood temperatures did not prove fatal for bee survival during overwintering in our experiment (**S1 Table**). Furthermore, since

Interestingly, the typical nest temperature range for honey bee brood rearing fluctuates between 33 and 36°C (Kovac et al., 2009). However, honey bee Group 25 maintained a subnormal brood temperature, lower than 3.403°C compared to Group 35 at the end of August **(Fig 1)**. Additionally, Szentgyörgyi et al. (2018) [68] reported that short-lived honey bees live longer after developing at a low temperature (32°C), we preferred the latter explanation and hypothesized that reduction of brood temperature could be related to the development of physiological and molecular markers in winter bees, which are responsible for the processes of aging and longevity [69].

Generally, which is crucial for overwintering. Due to the successful overwintering of honey bees Groups 25 and 35, we suggest that However, in high

Formatted: Font: Bold

Formatted: Font: Not Bold

Formatted: Font: Bold

Formatted: Font: Not Bold

Formatted: Font: Not Bold

ambient temperatures, this adjustment was not feasible.honey bees initially had to decrease the nest temperature to rear brood with a slower metabolism, larger hypopharyngeal gland (HG) size, and higher Fat body weight for overwintering (Knoll et al., 2020). However, in high ambient temperatures, this adjustment was not feasible. Moreover, we confirmed that high ambient temperatures had an indirect effect on honey bees' transition through changes in physiology and molecular markers value at the end of winter.

Tthe physiological markers of long-lived (winter) honey bees include hypertrophied HG compared to newly emerged and forager summer bees [28]. Another physiological marker is , which plays a key function in royal jelly production for larval rearing in spring, and an enlarged fFat body compared to summer bees [27], resulting from the accumulation of nutrients, particularly lipids [30](Smedal et al., 2009). In our research, the A comparative study revealed that aAcinusi areasizes in honey bees from Groups 25 and the control wasere significantly largergreater (ANOVA, Duncan's post hoc, $p < 0.05$) than in those from in honey bee Group 35. The same trend was observed in the mass of lipid content in the fFat body weights, although the differences were not statistically significant. Given that the sampling was conducted on February 5-6, these physiological differences were indicative of the honey bees' diapause condition. The honey bees Groups 25 and control exhibited a higher body nutrient stock compared tothan the honey bee Group 35.

All of this , suggesting an aging process.

Formatted: Highlight

~~This process is~~ closely associated with molecular markers [40, 70, 71]~~(Alaux et al., 2018; Kaur and Singh, 2020; Daniels et al., 2023~~), especially ~~.~~vg which is synthesized in the fat body [30]. Vg is then transferred to the HG and used as an amino acid donor for the synthesis of royal jelly to feed larvae after diapause ends [72]. Along with elevated vg levels, another molecular marker, suppressed juvenile hormone (JH) levels, is used to quantify the condition of winter honey bees compared ~~In long-lived (winter) honey bees, these markers include elevated vg levels and suppressed JH levels (Smedal et al., 2009) in comparison~~ to ~~short-lived (spring-~~summer) honey bees [1, 38, 33]~~(Amdam and Omholt, 2002; Döke et al., 2015; Knoll et al., 2020~~). These molecular levels remain stable during diapause and undergo a switch before the onset of brood rearing [38]~~(Amdam and Omholt, 2002~~). Notably, *vg* gene expression was significantly higher in honey bees from Group 25 and the control, whereas *JHAMT* gene expression was significantly lower compared to ~~honey bees from~~ Group 35, ~~which also~~ suggestings an aging process in the latter ~~honey~~ bees.

To utilize these well-known interactions, we built Elastic Net Regression models for acini area and fat body mass **(Fig 5A, and B).** In this both models, only two genes — *vg* and *ilp2* — showed high positive and negative coefficient values, respectively. This indicates that during winter diapause, *vg* and *ilp2* gene expressions are expected to be higher and lower, respectively, with larger acini area and fat body mass in honey bees under normal conditions. This supports the well-

Formatted: Font: Not Bold

Formatted: Highlight

Formatted: Font: Not Bold

Formatted: Highlight

Formatted: Highlight

Formatted: Font: Not Bold

Formatted: Font: Not Bold

Formatted: Font: Not Bold

Formatted: Font: Italic

Formatted: Font: Italic

Formatted: Font: Italic

Formatted: Font: Italic

known relationship between *vg* and physiological markers, and suggests a new relationship between molecular marker *ilp2* and physiology markers. However, in bees from Group 35, this relationship was reversed, which also implied some aging issues.

To identify genes that can significantly discriminate between honey bee groups, Discriminant Analysis (DA) was implemented. Three genes—*JHAMT, vg,* and *HSP70*—were identified with high scores, with the first two being already well-known. The relationship between *vg* gene expression and the hypopharyngeal gland (HG) and Fat body was validated using Elastic Net Regression models **(Fig 3B, C).** Furthermore, Discriminant Analysis (DA) identified three genes, *JHAMT, vg*, and *HSP70,* with high scores, underscoring their significance in discriminating among honey bee groups. Even though the *HSP70* gene is synthesized in response to stress [73](Zhang et al., 2015), Sarıoğlu-Bozkurt et al. [55] reported that its levels also increase in overwintered honey bees from fall to early spring and respond to different supplemental diets along with *vg*, demonstrating the complex regulation of this gene. We found that it is associated with honey bee transitions in summer and aging (Sario˘glu-Bozkurt et al., 2022). Tthe highest *HSP70* gene expression was observed was observed in the winter honey bees from –Group 35, while while the lowest was found was in the Group control. Based on this, we suggest that honey bees from Group 35 responded on some a stress group, , possibly related to their different nutritional needs, which may later stimulate the ending of diapause and the initiation of earlier colony development compared to honey bees from Groups control and 25. Also, the

Formatted: Font: Italic

Formatted: Font: Italic

Formatted: Font: Italic

Formatted: Font: Italic

Formatted: Highlight

Formatted: Font: Italic

increased nutritional needs of honey bees from Group 35 are explained by the significant increase in *TOR1* gene expression, because this evolutionarily conserved gene integrates signals from nutrients (amino acids and energy) and growth factors to regulate cell growth [50, 74, 75]~~Barth et al., 2010; Fahrbach et al., 2012~~. Additionally, significantly higher *HSP70* and *TOR1* gene expressions in honey bees from Group 35, compared to Group control and Group 25, were associated with a decrease in fat body weight, although this decrease was not yet statistically significant. This support~~ing~~s the aging hypothesis for ~~of~~ temperature-treated honey bees. However, ~~The subsequent gene,~~ *TOR1*~~,~~ gene was not~~n't~~ selected by Data Mining methods as marker possibly because it had less fluctuation between Groups of honey bees compared to other genes~~.~~. ~~but can be considered aging-related (Barth et al., 2010; Fahrbach et al., 2012). This gene exhibited high expression in honey bee Group 35, explaining the decrease in Fat body weight compared to the Group control and 25, even though it was not significant.~~

The ~~last two~~ gene~~s,~~ *ilp1*~~,~~ didn’t show a clear effect in our experiment and ~~and *ilp2*~~ requires careful consideration due to its ~~as they exhibit~~ complex metabolic ~~interactions with~~ relationships influenced~~that depend not only on~~ by nutrition, age, and season [76, 77] ~~(Nilsen et al., 2011; Chowański et al., 2021~~).

In summary, our study found that exposure to constant day-night temperatures of 25°C and 35°C negatively impacted colony growth in the fall. The 25°C

Formatted: Font: Italic

Formatted: Highlight

Formatted: Font: Italic

Formatted: Font: Italic

Formatted: Font: Not Italic

Formatted: Font: Not Italic

Formatted: Font: Not Italic

Formatted: Font: Not Bold

Formatted: Highlight

temperature closely resembled natural fall conditions in Incheon, allowing honey bees to regulate the microclimate by lowering brood temperatures. This facilitated the development of winter bees with metabolic markers similar to those of the Group control. In contrast, the 35°C temperature hindered colony development, as honey bees in Group 35 were unable to reduce brood temperatures as effectively as those in Group 25, impairing winter bee development. In February, we observed increased levels of *JHAMT, TOR1*, and *ilp2*, and decreased levels of *vg*, which were associated with reduced nutrient stores in the fat body mass and a smaller acini area. However, these changes did not result in fatal outcomes for honey bee survival in our experiment. These results can be used to correlate climate change factors, such as fall temperatures, with bee responses in winter. Understanding how organisms respond to extreme heat can inform apiculture practices and help develop guidelines for managing honey bees in winter to prevent losses. we suggested a connection between regular ambient temperature, simulated climate warming, below-average brood temperature, and the delayed aging of long-lived honey bees in February. Conversely, under conditions of high-temperature stress, honey bees were unable to lower brood temperature, resulting in the expedited aging of long-lived individuals throughout the winter season. The acceleration of biological aging in Group 35 honey bees was evidenced by downregulated *vg* gene expression and upregulated *JHAMT, HSP70*, and *TOR1* gene expression. These molecular changes resulted in low internal nutrient stores in the Fat body and reduced Acinus sizeClimate change poses a threat

Formatted: Font: Italic

Formatted: Font: Italic

Formatted: Font: Italic

to organisms worldwide, as does understanding how organisms respond to extreme heat events, associated with climate change can inform conservation actions., suggesting a potential set of molecular markers for aging. We believe that this research can pave the way for additional studies and, ultimately, the development of a marker kit to identify the reasons and processes of aging at an early stage. could lead to further studies and the development of a test kit to identify early causes of aging.

This can help protect nature and humans from ecological challenges. Climate change poses a threat to organisms worldwide, as does understanding how organisms respond to extreme heat events, associated with climate change can inform conservation actions.

Formatted: Font: 18 pt

Materials and methods

Experimental bees

Western honey bees (*Apis mellifera ligustica*) were obtained from nine colonies kept at the Incheon National University apiary in the

~~Republic of Korea **(Fig 6A, B, C, and D)**, managed by a professional beekeeper following standard beekeeping practices (Delaplane et al., 2013).~~

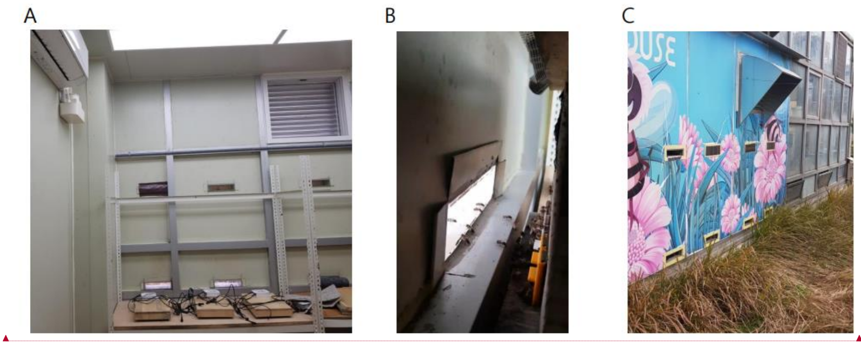

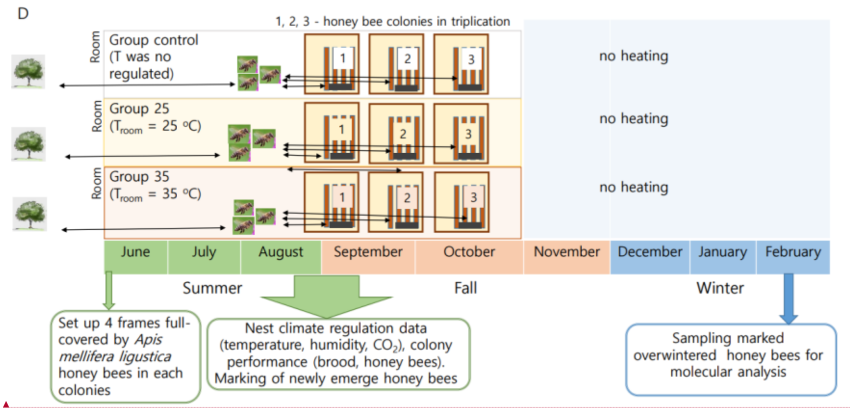

Formatted: Font: 18 pt

Formatted: Font: 18 pt

Formatted: Font: 18 pt

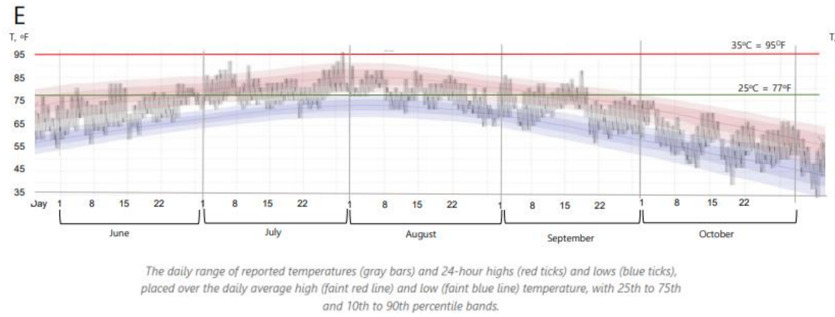

~~**Fig 6. Overview of the experimental design (A)** The room environment before the experiment. **(B)** The process of honey bees freely flying outside from the experimental room. **(C)** The view of the room and the entrance for honey bees outside. **(D)** Timeline of the experiment. **(E)** Ambient temperature of the Group control, Group 25 (green line), and Group 35 (red line) under the temperature experiment in 2022, Incheon, Republic of Korea. The data were obtained from https://weatherspark.com/h/m/142040/2022/10/Historical-Weather-in-October-2022-in-Incheon-South-Korea#Figures-Temperature.~~

~~The experimental colonies housed first-year young sister queens, naturally mated and beginning egg-laying in May 2022. Prior to the experiment in June, colonies were standardized in terms of the~~

Formatted: Font: 18 pt

Formatted: Font: 18 pt

Formatted: Font: 18 pt

number of frames (total 4), brood population (3 frames), honey amount (1 frame), and the number of honey bees. Colony strength was assessed through visual inspection of adult and brood populations both before and during the experiment (Delaplane et al., 2013).

Two rooms equipped with a climate-control system were established to maintain honey bee colonies at 25°C (Group 25) and 35°C (Group 35), simulating climate warming without day and night temperature differences. These temperatures were selected to mimic the effects of climate change, with 25°C representing the summer average and 35°C being the highest recorded in Incheon City under typical conditions. However, the highest temperature was not naturally reached during the summer of 2022 **(Fig 6E).** Other environmental conditions were not controlled. Light was used during colony inspections. In the third room, housing honey bee colonies, the control group was provided with free ventilation

without additional heating. All honey bees had equal opportunities to collect pollen and nectar around the apiary.

On September 18th and 25th, 2022, two combs with sealed brood were placed in an incubator set at 34°C. Upon hatching, the workers were individually marked with colored paint on their thoraxes and then reintroduced into their original colony. Throughout the previous summer, as well as in September and October of 2022, the honey bee colonies were subjected to varying room ambient temperatures. Afterward, the controlled temperatures in the rooms were turned off, and the honey bee colonies were overwintered in the same rooms under natural temperature conditions.

The overwintered marked honey bees, aged approximately 4.5 months, were sampled on February 5th, 2023, a time when queens had not yet begun laying eggs and the overwintered bees were expected to have successfully survived the winter. Forty honey bees from each colony were sampled and individually placed in labeled

~~50 ml tubes, then stored at -80 °C before analysis.~~

~~Brood microclimate measurements~~

~~The temperature, humidity (AM2320, BeeOnFarm, Korea), and CO_2 concentration (BO-100, BeeOnFarm, Korea) sensor systems were placed on the top and middle of each colony in August 2022. The data logging system was included in the sensor system because it provided a real-time clock. Data consisting of time (seconds), CO_2 (in ppm), temperature (in °C), and relative humidity (as a percentage) were saved, with approximately three measurements in minutes to approximately one file per day. The data from 6 honey bee colonies kept in each room (three at 25°C and three at 35°C) were collected over 15 days from 11 to 25 August. Average values were used for statistical calculations.~~

~~Hypopharyngeal gland (HG) measurements~~

~~The Acinus size from HGs of overwintered honey bees Groups~~

control, 25 and 35 were measured to determine whether differed depending on the ambient temperature in the previous fall season. Thirty bees were collected from each colony, flash frozen in liquid nitrogen, and maintained at −80 °C until their glands were measured. For each bee, the HGs were dissected into 1× phosphate-buffered saline (PBS) with 0.1% Tween® 20 and visualized at 40× magnification. The area (mm^2) of a minimum of 10 randomly selected Acinus per bee was measured using Nikon Eclipse E200 (Nikon, Tokyo, Japan) Toupview (Touptek, Hangzhou, China) software. Only Acinus with clear borders were used. Acinus areas were averaged within individuals.

Fat body lipid content

The Fat body lipid content in the abdomen of overwintered honey bees was studied in early February by the standard method (Martin, 1969; Human et al., 2013). Ten honey bees were collected from each colony; 90 honey bees were flash frozen in liquid nitrogen and

maintained at −80 °C until their abdomens were dissected and their guts were removed. Pools of 10 abdomens were weighed on a scale (name, company, country) before and after drying to a constant weight in a fume hood (SSFH-1000, Shinsaeng, Korea). The total lipid content was determined by measuring the decrease in weight following the removal of lipids from the dried tissue through extraction in 5 ml portions of diethyl ether (179272-1 L; Sigma-Aldrich, USA).

RNA Extraction and cDNA Synthesis

A total of 90 honey bee (10 winter bees from each colony) *Apis mellifera ligustica* colonies were sampled in labeled tubes and stored at -80°C. Three honey bees from each colony were analyzed. RNA was extracted from the brain for *HSP70, JHAMT, ilp1, ilp2*, and *TOR1* expression analysis and from the abdomen for *vg* gene expression analysis. Total tissue RNA (brain or abdomen) was extracted from 15 randomly selected bees in each group using a

Qiagen RNeasy Mini Kit (#74,104; Qiagen, Valencia, CA, USA). The tissues in each replicate were brain tissue for *TOR1, ilp1, ilp2, JHAMT*, and *HSP70* and abdomen for *vg* gene expression. The total RNA concentration and purity were quantified using OD260/OD280 values between 1.8 and 2.0. Next, reverse transcription was performed using an RNA to cDNA EcoDryTM Premix (Oligo dT) kit (Takara, Japan). The reverse transcription reaction mixture included 50 ng/µl total RNA (with the clear volume calculated for each sample) and RNase-free water for a total volume of 20 µl. Reverse transcription was conducted at 42°C for 60 min, followed by heating at 70°C for 10 min.

Quantitative real-time PCR

The relative expression of six genes related to nutrition (*vg, ilp1, ilp2, TOR1), development (JHAMT),* and stress response (*HSP70)* was measured. The housekeeping gene *RpL32* (Ribosomal protein L32*)* was used as an endogenous control. The PCR primer sequences are

shown in **S1 Table.**

The reaction conditions were optimized. The qRT PCR mixture had a volume of 20 µl which included 2 µl of template cDNA, 10 µl of Green qPCR Master Mix, 1 µl of upstream and downstream primers (5 pM/µl), and 6 µl of nuclease-free water (AM9930, Invitrogen, USA). Quantitative real-time PCR (qRT PCR) was conducted using Brilliant III Ultra-Fast SYBR® Green qPCR Master Mix (600882, Agilent Technologies, USA) on an AriaMx Real-Time PCR System (Agilent Technologies LDA, Malaysia) with AriaMx 96 Well Plates, Skirted, LP (401490, Agilent Technologies, USA) covered by MicroAmp™ Optical Adhesive Film (4311971, Thermo Fisher Scientific, USA).

The qRT PCR amplification procedure was as follows: initial denaturation at 95°C for 10 minutes; 40 cycles of denaturation at 95°C for 30 seconds, annealing at 60°C for 25 seconds, and extension at 72°C for 15 seconds. Each sample was technically

replicated three times. Data analysis was performed using Agilent AriaMx version 2.0 analysis software. Relative gene expression data were analyzed using the 2^(-Delta Delta C(T)) method (Livak and Schmittgen, 2001; Bustin et al., 2009).

Confirmation of amplicons in the RT–PCR products was performed by separating them through electrophoresis in a 2% agarose gel at 80 V for 40 minutes and analyzing them using a gel documentation system, the Gerix 1010 transilluminator (Biostep GmbH, USA). A Dyne 50 bp DNA Ladder (Cat. No. A701, DYNEBIO, Korea) was used as a reference.

Statistical analysis

The statistical analysis, designed as illustrated in **Fig 7**, was conducted using Microsoft Excel and XLSTAT software (Addinsoft Pearson Edition 2014, Addinsoft, Paris, France). The characterization was performed using the following strategies: (a) classification of the marker dataset to find a combination of features

that best separates different groups (supervised approach, Discriminant Analysis (DA)); (b) clustering to discover natural groupings in the absence of labeled information (unsupervised approach, Agglomerative Hierarchical Clustering (AHC)); and (c) modeling the relationship between independent variables and a dependent variable and dealing with multicollinearity in the marker dataset to rank the markers by importance for identifying overwintered honey bee task development (supervised approach, Elastic Net Regression).

Apis mellifera ligustica honey bees

Month of sampling		Group control (3 colonies)	Group 25 (3 colonies)	Group 35 (3 colonies)
Experimental condition (summer, September, and October)			T_{room} = 25 °C	T_{room} = 35 °C
**August September**	Data set 1 (brood microclimate and colony development, marking of hatched honey bees)		Nest climate regulation	
	Statistics of data set 1		T-test ($p < 0.05$)	
**February**	Sampling of marked honey bees, n	(for physiology markers): 30+30+30<br>(for molecular markers): 10+10+10	30+30+30<br>10+10+10	30+30+30<br>10+10+10
	Data set 2	Two physiology markers (hypopharengial glands (HG) and fat body)<br>Molecular marker genes related to nutrition (*vg*, *ilp1*, *ilp2*, *TOR1*), development (*JHAMT*), and stress response (*HSP70*).		
	Statistics of data set 2	1. Discriminant analysis (define the variables with high discrimination score);<br>2. Agglomerative Hierarchical Clustering (check the natural groupings within dataset);<br>3. Elastic Net Regression (model the relationship between HG and fat body with molecular markers);<br>4. ANOVA, Duncan post-hoc test (comparison of significance within each variable, $p < 0.05$).		

Fig 7. Experimental conditions, sampling schedule, data sets,

Formatted: Font: 18 pt

Formatted: Font: 18 pt

and statistical tests used for analysis

Specifically, the honey bees were categorized into groups, and the variables with the highest discrimination scores were selected using DA because it determines the linear combination of variables and provides maximal separation between groups. To discriminate the group, Fisher distances were applied, which emphasize the linear combinations of variables that maximize the differences between groups.

AHC analysis provided a visual representation of the clustered dataset, revealing the arrangement of individual data points or observations based on their dissimilarity. The evaluation metric for clustering algorithms was the silhouette index. The score is bounded between (− 1) for incorrect clustering and (+ 1) for highly dense clustering.

Elastic Net Regression analysis was used to test two models of the relationship (Jaqaman and Danuser, 2006; Moses, 2017) between

~~physiology and molecular markers. The dependent variables were Acinus size and Fat body weight. The seven independent variables were ranked by coefficient. Receiver operating characteristic (ROC) curves were generated to quantify how accurately regression analysis can discriminate between honey bee groups (Hajian-Tilaki, 2013). The ROC specificity and sensitivity plot demonstrated a perfectly fitted model linking physiology to molecular markers (gene expression profile). The area under the curve (AUC) can range from 0.5 to 1.0, with a preference for higher scores.~~

~~The mean, standard deviation, and variance of each gene were calculated using descriptive statistics and visualized using the heat map module. For the gene expression analysis, ANOVA was used to test overall effects, followed by the Duncan post hoc test ($p < 0.05$) for multiple comparisons and the t-test for gene expression differences between groups of honey bees. The bar plots were generated using the online SRplot Official Website~~

~~(https://www.bioinformatics.com.cn/en; accessed on 30 October 2023).~~

Supporting information

S1 Table. Survival of honey bee colonies at the end of the overwintering (PDF)

S~~1~~2 Table. PCR primer sequences. (PDF)

S 1 Fig. The quality verification plot (specificity vs sensitivity) of Elastic Net Regression model demonstrated a perfectly fitted model linking physiology to molecular markers (gene expression profile) (.tif)

S1 Fig. Gel electrophoresis to verify the amplicons after qPCR. (.tif)

S1 File. Raw data from sensors and XLSTAT 2022.xlsx

S2 File. Raw data of acini area, fat body mass, and colony perfomance.xlsx

S3 File. Raw data qPCR.xlsx

S4 File. Stat analysis in XLSTAT.xlsx

~~(PDF)~~

Formatted: Font: Not Bold
Formatted: Font: 14 pt, Not Bold
Formatted: Font: Not Bold
Formatted: Line spacing: Double
Formatted: Font: 14 pt, Not Bold
Formatted: Font: Not Bold
Formatted: Font: 14 pt, Not Bold
Formatted: Font: 14 pt
Formatted: Font: 14 pt
Formatted: Font: 14 pt, Not Bold
Formatted: Font: 14 pt, Not Bold
Formatted: Font: 14 pt
Formatted: Line spacing: Double
Formatted: Font: 14 pt, Not Bold
Formatted: Font: 14 pt
Formatted: Font: 14 pt, Not Bold
Formatted: Font: 14 pt
Formatted: Font: 14 pt, Not Bold
Formatted: Font: 14 pt
Formatted: Font: 14 pt, Not Bold
Formatted: Font: 14 pt
Formatted: Font: 14 pt, Not Bold
Formatted: Font: 14 pt
Formatted: Font: 14 pt, Not Bold
Formatted: Font: 14 pt
Formatted: Font: 14 pt, Not Bold
Formatted: Font: 14 pt
Formatted: Font: 14 pt, Not Bold
Formatted: Font: 14 pt
Formatted: Font: 14 pt, Not Bold
Formatted: Font: 14 pt
Formatted: Font: 16 pt

Acknowledgments

The authors thank BeeOnfarm company (Republic of Korea) for providing the equipment to record the in-hive microclimate. Mention of trade names or commercial products in this publication is solely to provide specific information and does not imply recommendation or endorsement by the INU.

Author Contributions

Conceptualization: Yumi Yun, Hyung-Wook Kwon

Data curation: Yumi Yun

Formal analysis: Olga Frunze, Ravil R. Garafutdinov, Hyun-~~J~~jee Kim

Investigation: Hyung-Wook Kwon, Young-Eun Na

Methodology: Ravil R. Garafutdinov, Olga Frunze, Yumi Yun

Resources: Hyung-Wook Kwon, Young-Eun Na, Hyun-j~~J~~ee Kim

Software: Olga Frunze, Ravil R. Garafutdinov

Supervision:, Hyung-Wook Kwon, Young-Eun Na

Visualization: Olga Frunze

Writing-original draft: Olga Frunze, Yumi Yun

Writing-review&editing: Olga Frunze, Yumi Yun, Hyunj~~-J~~ee Kim, Hyung-Wook Kwon

Reference

Formatted: Line spacing: Double

1. Döke MA, Frazier M, Grozinger CM. Overwintering honey bees: biology and management. Curr Opin Insect Sci. 2015; 10:185-93.
2. Hristov P, Shumkova R, Palova N, Neov B. Factors associated with honey bee

colony losses: a mini-review. Vet Sci. 2020; 7:166.

3. Gray A, Adjlane N, Arab A, Ballis A, Brusbardis V, Dougla AB. et al. Honey bee colony loss rates in 37 countries using the COLOSS survey for winter 2019–2020: the combined effects of operation size, migration and queen replacement. J Apic Res. 2023; 62:204-10.
4. Calovi M, Grozinger CM, Miller DA, Goslee SC. Summer weather conditions influence winter survival of honey bees (*Apis mellifera*) in the northeastern United States. Sci Rep. 2021; 11: 1553.
5. Dosio A, Mentaschi L, Fischer EM, Wyser K. Extreme heat waves under 1.5°C and 2°C global warming. Environ Res. 2018; 13: 054006.
6. Lamb MJ. Temperature and lifespan in *Drosoph*. Nat. 1968; 220: 808–809.
7. Liu RK, Walford RL. Increased growth and lifespan with lowered ambient temperature in annual fish *Cynolebias adloffi*. Nature. 1966; 212: 1277–78.
8. Mishra DM, Bhunia P, Shubham S, Sen,R, Bhunia R, Gulia J, et. al. The impact of weather changes on honey bee populations and disease. J Adv Zool. 2023; 44: 180–90.
9. Conti NB, Sanchez-Alavez M, Winsky-Sommerer R, Morale MC, Lucero J, Brownell S, et al. Transgenic mice with a reduced core body temperature have an increased life span. Science. 2006; 314, 825-8.
10. Fahrenholz L, Lamprecht I, Schricker B. Thermal investigations of a honey bee colony: thermoregulation of the hive during summer and winter and heat production

Formatted: Indent: Left 0 ch, Line spacing: Double

Formatted: Font: Italic

of members of different bee castes. J Comp Physiol. 1989; 159: 551-60.

11. Switanek M, Crailsheim K, Truhetz H, Brodschneider R. Modelling seasonal effects of temperature and precipitation on honey bee winter mortality in a temperate climate. Sci Total Environ. 2017; 579: 1581–7.

12. van Dooremalen C, Gerritsen L, Cornelissen B, van der Steen JJ, van Langevelde F., Blacquière T. Winter survival of individual honey bees and honey bee colonies depends on level of *Varroa destructor* infestation. PloS ONE. 2012; 7: e36285.

13. Morawetz L, Köglberger H, Griesbacher A, Derakhshifar I, Crailsheim K, Brodschneider R, et al. Health status of honey bee colonies (*Apis mellifera*) and disease-related risk factors for colony losses in Austria. PLoS ONE. 2019; 14: e0219293.

14. Fluri P, Lüscher M, Wille H, Gerig L. Changes in weight of the pharyngeal gland and hemolymph titers of juvenile hormone, protein and vitellogenin in worker honey bees. J Insect Physiol. 1982; 28:61-8.

15. Fluri P, Wille H, Gerig L, Luscher M. Juvenile hormone, vitellogenin and hemocyte composition in winter worker honeybees (*Apis mellifera*). Experientia 1977; 33: 1240–41.

16. Crailsheim K. Dependence of protein-metabolism on age and season in the honeybee (*Apis mellifica carnica* Pollm). J. Insect Physiol. 1986; 32:629–34.

17. Denlinger DL. Insect diapause: from a rich history to an exciting future. J Exp Biol. 2023; 226 (4): 245329.

18. Münch D, Kreibich CD, Amdam GV. Aging and its modulation in a long-lived worker caste of the honey bee. J Exp Biol. 2013; 216: 1638–49.

19. Coulibaly KA, Majeed MZ, Sayed SM, Yeo K. Simulated climate warming influenced colony microclimatic conditions and gut bacterial abundance of honeybee subspecies *Apis mellifera ligustica* and *A. mellifera sinisxinyuan*. J Apic Sci. 2022; 66: 15-27.

20. Simpson J. Nest Climate regulation in honey bee colonies. Science. 1961; 133:1327–1333.

21. Abou-Shaara H, Owayss A, Ibrahim Y, Basuny N. A review of impacts of temperature and relative humidity on various activities of honey bees. Insectes Soc. 2017; 64: 455 – 63.

22. Bencsik M, McVeigh A, Tsakonas C, Kumar T, Chamberlain L, Newton MI. A Monitoring system for carbon dioxide in honeybee hives: An indicator of colony health sensors (Basel). 2023; 23(7):3588.

23. Winston ML. The Biology of the honey bee. Harvard University Press, Cambridge. 1987; 294.

24. Hartfelder K, Engels W. Social insect polymorphism: hormonal regulation of plasticity in development and reproduction in the honeybee. Curr Top Dev Biol. 1998; 40:45-77.

25. Rodrigues MA, Flatt T. Endocrine uncoupling of the trade-off between reproduction and somatic maintenance in eusocial insects. Curr Opin Insect Sci. 2016; 16:1e8.

26. Bresnahan ST, Döke MA, Giray T, Grozinger CM. Tissue-specific transcriptional patterns underlie seasonal phenotypes in honey bees (*Apis mellifera*). Mol Ecol. 2022; 31:174-84.

27. Brejcha M, Prušáková D, Sábová M, Peska V, Černý J, Kodrík D, et al. Seasonal changes in ultrastructure and gene expression in the fat body of worker honey bees. J Insect Physiol. 2023; 146:104504.

28. Deseyn J, Billen J. Age-dependent morphology and ultrastructure of the hypopharyngeal gland of *Apis mellifera* workers (Hymenoptera, Apidae) Apidologie. 2005; 36: 49-57.

29. Dohanik VT, Medeiros-Santana L, Santos CG, Santana, WC, Serrão JE. Expression and function of the vitellogenin receptor in the hypopharyngeal glands of the honey bee *Apis mellifera* (Hymenoptera: Apidae) workers. Arch Insect Biochem Physiol. 2024; 116: e22120.

30. Smedal B, Brynem M, Kreibich C, Amdam G. Brood pheromone suppresses physiology of extreme longevity in honeybees (*Apis mellifera*). J Exp Biol. 2009; 212: 3795-801.

31. Arrese EL, Soulages JL. Insect fat body: energy, metabolism, and regulation. Annu Rev Entomol. 2010; 55: 207-25.

32. Amdam GV, Aase AL, Seehuus SC, Kim Fondrk M, Norberg K, Hartfelder K. Social reversal of immunosenescence in honey bee workers. Exp Ger Ontol. 2005; 40: 939-47.

33. Knoll S, Pinna W, Varcasia A, Scala A, Cappa MG. The honey bee (*Apis mellifera* L., 1758) and the seasonal adaptation of productions. Highlights on summer to winter transition and back to summer metabolic activity. A review. Livest Sci. 2020; 235: 104011.

34. Schilcher F, Scheine R. New insight into molecular mechanisms underlying division of labor in honeybees. Curr Opin Insect Sci. 2023; 59: 101080.

35. Scheiner R, Reim T, Søvik E, Entler BV, Barron AB, Thamm M. Learning, gustatory responsiveness and tyramine differences across nurse and forager honeybees. J Exp Biol. 2017; 220:1443-50.

36. Corby-Harris V, Snyder LA. Measuring hypopharyngeal gland acinus size in honey bee (*Apis mellifera*) workers. J Vis Exp. 2018; 139:58261.

37. Corby-Harris V, Snyder L, Meador C. Fat body lipolysis connects poor nutrition to hypopharyngeal gland degradation in *Apis mellifera*. J Insect Physiol. 2019; 116:1-9.

38. Amdam GV, Omholt SW. The regulatory anatomy of honeybee lifespan. J Theor Biol. 2002; 216: 209-28.

39. Ali H, Alqarni AS, Iqbal J, Owayss AA, Raweh HS, Smith BH. Effect of season and behavioral activity on the hypopharyngeal glands of three honey bee *Apis mellifera* L. races under stressful climatic conditions of central Saudi Arabia. J Hymenopt Res. 2019; 68: 85-101.

40. Alaux C, Soubeyrand S, Prado A, Peruzzi M, Maisonnasse A, Vallon J et al.

Measuring biological age to assess colony demographics in honeybees. PLoS ONE. 2018; 13(12): e0209192.

41. Diebel LWM, Rockwood K. Determination of biological age: geriatric assessment *vs* biological biomarkers. Curr Oncol Rep. 2021; 23: 104.

42. Elekonich MM, Schulz DJ, Bloch G, Robinson GE. Juvenile hormone levels in honey bee (*Apis mellifera* L.) foragers: foraging experience and diurnal variation. J Insect Physiol. 2001; 47:1119-25.

43. Guidugli KR, Nascimento AM, Amdam GV, Barchuk AR, Omholt S, Simoes ZL et al. 2005. Vitellogenin regulates hormonal dynamics in the worker caste of a eusocial insect. FEBS Lett. 2005; 579: 4961-65.

44. Amdam GV, Page RE. The developmental genetics and physiology of honeybee societies. Anim Behav. 2010;79(5): 973-80.

45. Goodman WG, Granger NA. The juvenile hormones. In: Lawrence IG, Kostas I, Sarjeet SG. Comprehensive Molecular Insect Science. Elselvier. 2005; pp. 319-408.

46. Li W, Huang ZY, Liu F, Li Z, Yan L, et al. Molecular cloning and characterization of juvenile hormone acid methyltransferase in the honey bee, *Apis mellifera*, and its differential expression during caste differentiation. PLoS ONE. 2013; 8:e68544.

47. Egi Y, Sakamoto K. Genome-wide screening of genes involved in programming diapause in the next generation in silkworm, *Bombyx mori* (Lepidoptera: Bombycidae). Eur J Entomol. 2022; 119:405-12.

48. Corona M, Velarde RA, Remolina S, Moran-Lauter A, Wang Y, Hughes KA et al.

Formatted: Indent: Left 0 ch, Line spacing: Double

Vitellogenin, juvenile hormone, insulin signaling, and queen honey bee longevity. Proc Natl Acad Sci USA. 2007; 104: 7128-33.

49. Zhang W, Wang L, Zhao Y, Wang Y, Chen C, Hu Y, et al. Single-cell transcriptomic analysis of honeybee brains identifies vitellogenin as a caste differentiation-related factor. iScience. 2022; 25: 104643.

50. Barth JMI, Szabad J, Hafen E, Köhler K. Autophagy in *Drosophila* ovaries is induced by starvation and is required for oogenesis. Cell Death Differ. 2010; 18: 915.

51. Patel A, Fondrk MK, Kaftanoglu O, Emore C, Hunt G Frederick K, et al. The making of a queen: TOR pathway is a key player in diphenic caste development. PLoS ONE. 2007; 2: e509.

52. Laplante M, Sabatini DM. mTOR signaling in growth control and disease. Cell. 2012; 149:274-293.

53. Johnson SC, Rabinovitch PS, Kaeberlein M. mTOR is a key modulator of aging and age-related disease. Nature. 2013; 493:338-345.

54. Zoncu R, Efeyan A, Sabatini DM. mTOR: from growth signal integration to cancer, diabetes and aging. Nat Rev Mol Cell Biol. 2011; 12:21-35.

55. Sarioğlu-Bozkurt A, Topal E, Güneş N, Üçeş E, Cornea-Cipcigan M, Coşkun İ, et al. Changes in vitellogenin (*Vg*) and stress protein (*HSP 70*) in honey bee (*Apis mellifera anatoliaca*) groups under different diets linked with physico-chemical, antioxidant and fatty and amino acid profiles. Insects. 2022; 13: 985.

56. Delaplane KS, van der Steen J, Guzman-Novoa E. Standard methods for estimating

Formatted: Font: Italic

strength parameters of *Apis mellifera* colonies. J Apic Res. 2013; 52: 1-12.

57. Martin JS. Lipid composition of fat body and its contribution to the maturing oöcytes in *Pyrrhocoris apterus*. J Insect Physiol. 1969; 15:1025-45.

58. Human H, Brodschneider R, Dietemann V, Dively JD, Ellis E, Forsgren I, et al. Miscellaneous standard methods for *Apis mellifera* research. J Apic Res. 2013; 52:1-53.

59. Livak KJ, Schmittgen TD. Analysis of relative gene expression data using real-time quantitative PCR and the 2(-Delta Delta C(T)) Method. Methods. 2001; 25:402–408.

60. Bustin SA, Benes V, Garson JA, et al. The MIQE guidelines: minimum information for publication of quantitative real-time PCR experiments. Clin Chem. 2009; 55:611-22.

61. Moses AM. Statistical modeling and machine learning for molecular biology. CRC Press Taylor & Francis Group. 2017: 280 p.

62. Jaqaman K, Danuser G. Linking data to models: data regression. Nat Rev Mol Cell Biol. 2006; 7:813–819.

63. Hajian-Tilaki K. Receiver operating characteristic (ROC) curve analysis for medical diagnostic test evaluation. Caspian J Intern Med. 2013; 4: 627-35.

64. Medrzycki P, Sgolastra F, Bortolotti L, Bogo G, Tosi S, Padovani E, et al. Influence of brood rearing temperature on honey bee development and susceptibility to poisoning by pesticides. J Apic Res. 2010; 49:52-59.

65. Quinlan GM, Feuerborn C, Hines HM, Grozinger CM. Beat the heat: thermal

respites and access to food associated with increased bumble bee heat tolerance. J Exp Biol. 2023; 226: jeb245924.

66. Stalidzans E, Berzonis A, Temperature changes above the upper hive body reveal the annual development periods of honey bee colonies. Comput Electron Agric. 2013; 90: 1-6.

67. Kovac H, Stabentheiner A, Brodschneider R. Contribution of honeybee drones of different age to colonial thermoregulation. Apidologie. 2009; 40:82-95.

68. Szentgyörgyi H, Czekońska K., Tofilski A. Honey bees are larger and live longer after developing at low temperature. J Therm Biol. 2018; 78:219-226.

69. Kovac H, Crailsheim K. Lifespan of *Apis mellifera carnica* Pollm. infested by *Varroa jacobsoni* Oud. in relation to season and extent of infestation. J Apic Res. 1988; 27: 230-8.

70. Kaur R, Singh D. Molecular markers a valuable tool for species identification of insects: A review. Ann Entomol. 2020; 38:1-20.

71. Daniels BC, Wang Y, Page RE Jr, Amdam GV. Identifying a developmental transition in honey bees using gene expression data. PLoS Comput Biol. 2023; 9(9):e1010704.

72. Ramanathan ANKG, Nair AJ, Sugunan VS. 2018. A review on royal jelly proteins and peptides. J Funct Foods. 2018; 44: 255-64.

73. Zhang Y, Liu Y, Zhang J, Guo Y, Ma E. Molecular cloning and mRNA expression of heat shock protein genes and their response to cadmium stress in the drasshopper

Oxya chinensis. PLoS ONE. 2015; 10: e0131244.

74. Fingar D, Blenis J. Target of rapamycin (TOR): an integrator of nutrient and growth factor signals and coordinator of cell growth and cell cycle progression. Oncogene. 2004; 23: 3151–71.

75. Fahrbach SE, Nambu JR, Schwartz LM. Programmed cell death in insects. Insect Mol Biol Biochem. 2012; 12:419–449.

76. Nilsen KA, Ihle KE, Frederick K, Fondrk MK, Smedal B, Hartfelder K, et al. Insulin-like peptide genes in honey bee fat body respond differently to manipulation of social behavioral physiology. J Exp Biol. 2011; 1:1488-97.

77. Chowański S, Walkowiak-Nowicka K, Winkiel M, Marciniak P, Urbański A, Pacholska-Bogalska J. Insulin-like peptides and cross-talk with other factors in the regulation of insect metabolism. Front Physiol. 2021; 12:701203.

Data Availability Statement: All relevant data are the manuscript and its Supporting information files.

~~1. Abou-Shaara H, Owayss A, Ibrahim Y., Basuny, N. A review of impacts of temperature and relative humidity on various activities of honey bees. Insectes Soc. 2017; 64: 455–463.~~

~~2. Alaux C, Soubeyrand S, Prado A, Peruzzi M, Maisonnasse A, Vallon J et al. Measuring biological age to assess colony demographics in honeybees. PLoS ONE. 2018; 13(12):e0209192.~~

Formatted: Font: Bold

Formatted: Normal, Indent: Left 1 ch, Line spacing: Double, No bullets or numbering

Formatted: Font: (Default) Times New Roman, 14 pt

Formatted: Line spacing: Double

3. Ali H, Alqarni AS, Iqbal J, Owayss AA, Raweh HS, Smith BH. Effect of season and behavioral activity on the hypopharyngeal glands of three honey bee *Apis mellifera* L. races under stressful climatic conditions of central Saudi Arabia. J Hymenopt Res. 2019; 68: 85-101.

4. Amdam GV, Aase AL, Seehuus SC, Kim Fondrk M, Norberg K, Hartfelder K. Social reversal of immunosenescence in honey bee workers. Exp Ger Ontol. 2005; 40: 939e947.

5. Amdam GV, Omholt SW. The regulatory anatomy of honeybee lifespan. J Theor Biol. 2002; 216: 209e228.

6. Ament SA, Corona M, Pollock HS, Robinson GE. Insulin signaling is involved in the regulation of worker division of labor in honey bee colonies. Proc Natl Acad Sci USA. 2008; 105: 4226-4231.

7. Barth JMI, Szabad J, Hafen E, Köhler K. Autophagy in Drosophila ovaries is induced by starvation and is required for oogenesis. Cell Death Differ. 2010; 18: 915.

8. Bencsik M, McVeigh A, Tsakonas C, Kumar T, Chamberlain L, Newton MI. A Monitoring System for Carbon Dioxide in Honeybee Hives: An Indicator of Colony Health Sensors (Basel). 2023; 23(7):3588.

9. Brejcha M, Prušáková D, Sábová M, Peska V, Černý J, Kodrík D, Konopová B, Čapková Frydrychová R. Seasonal changes in ultrastructure and gene expression in the fat body of worker honey bees. J Insect Physiol. 2023; 146:104504.

10. Bresnahan ST, Döke MA, Giray T, Grozinger CM. Tissue-specific transcriptional

patterns underlie seasonal phenotypes in honey bees (*Apis mellifera*). Mol Ecol. 2022; 31:174-184.

11. Bustin SA, Benes V, Garson JA, et al. The MIQE guidelines: minimum information for publication of quantitative real-time PCR experiments. Clin Chem. 2009; 55:611-622.

12. Chowański S, Walkowiak-Nowicka K, Winkiel M, Marciniak P, Urbański A, Pacholska-Bogalska J. Insulin-Like Peptides and Cross-Talk With Other Factors in the Regulation of Insect Metabolism. Front Physiol. 2021; 12:701203.

13. Corby-Harris V, Snyder L, Meador C. Fat body lipolysis connects poor nutrition to hypopharyngeal gland degradation in *Apis mellifera*. J Insect Physiol. 2019; 116:1-9.

14. Corby-Harris V, Snyder LA. Measuring Hypopharyngeal Gland Acinus Size in Honey Bee (*Apis mellifera*) Workers. J Vis Exp. 2018; 139:58261.

15. Corona M, Velarde RA, Remolina S, Moran-Lauter A, Wang Y, Hughes KA et al. Vitellogenin, juvenile hormone, insulin signaling, and queen honey bee longevity. Proc Natl Acad Sci USA. 2007; 104:7128-7133.

16. Coulibaly KA, Majeed MZ, Sayed SM, Yeo K. Simulated Climate Warming Influenced Colony Microclimatic Conditions and Gut Bacterial Abundance of Honeybee Subspecies *Apis mellifera ligustica* and *A. mellifera sinisxinyuan*. J Apic Sci. 2022; 66: 15-27.

17. Crailsheim K. Dependence of protein-metabolism on age and season in the honeybee (*Apis mellifica carnica* Pollm). J. Insect Physiol. 1986; 32:629–34.

18. Daniels BC, Wang Y, Page RE Jr, Amdam GV. Identifying a developmental transition in honey bees using gene expression data. PLoS Comput Biol. 2023; 9(9):e1010704.

19. Delaplane KS, van der Steen J, Guzman-Novoa E. Standard methods for estimating strength parameters of *Apis mellifera* colonies. J Apic Res. 2013; 52: 1-12.

20. Denlinger DL. Insect diapause: from a rich history to an exciting future. J Exp Biol. 2023; 226 (4): jeb245329.

21. Diebel LWM, Rockwood K. Determination of Biological Age: Geriatric Assessment *vs* Biological Biomarkers. Curr Oncol Rep. 2021; 23: 104.

22. Döke MA, Frazier M, Grozinger CM. Overwintering honey bees: biology and management. Curr Opin Insect Sci. 2015; 10:185-193.

23. Egi Y, Sakamoto K. Genome-wide screening of genes involved in programming diapause in the next generation in silkworm, *Bombyx mori* (Lepidoptera: Bombycidae). Eur J Entomol. 2022; 119:405-412.

24. Elekonich MM, Schulz DJ, Bloch G, Robinson GE. Juvenile hormone levels in honey bee (*Apis mellifera* L.) foragers: foraging experience and diurnal variation. J Insect Physiol. 2001; 47:1119e1125.

25. Fahrbach SE, Nambu JR, Schwartz LM. Programmed cell death in insects. Insect Mol Biol Biochem. 2012; 12:419–449.

26. Fluri P, Lüscher M, Wille H, Gerig L. Changes in weight of the pharyngeal gland and hemolymph titers of juvenile hormone, protein and vitellogenin in worker honey bees. J Insect Physiol. 1982; 28:61-68.

27. Fluri P, Wille H, Gerig L, Luscher M. Juvenile hormone, vitellogenin and haemocyte composition in winter worker honeybees (*Apis mellifera*). Experientia 1977; 33: 1240–1241.

28. Goodman WG, Granger NA. The juvenile hormones. In: Lawrence IG, Kostas I, Sarjeet SG. Comprehensive Molecular Insect Science. Elselvier. 2005; 319-408.

29. Gray A, Adjlane N, Arab A, Ballis A, Brusbardis V, Dougla AB. et al. Honey bee colony loss rates in 37 countries using the COLOSS survey for winter 2019–2020: the combined effects of operation size, migration and queen replacement. J Apic Res. 2023; 62:204-210.

30. Guidugli KR, Nascimento AM, Amdam GV, Barchuk AR, Omholt S, Simoes ZL et al. 2005. Vitellogenin regulates hormonal dynamics in the worker caste of a eusocial insect. FEBS Lett. 2005; 579:4961e4965.

31. Hajian-Tilaki K. Receiver Operating Characteristic (ROC) Curve Analysis for Medical Diagnostic Test Evaluation. Caspian J Intern Med. 2013; 4:627-635.

32. Hartfelder K, Engels W. Social insect polymorphism: hormonal regulation of plasticity in development and reproduction in the honeybee. Curr Top Dev Biol. 1998; 40:45e77.

33. Hristov P, Shumkova R, Palova N, Neov B. Factors Associated with Honey Bee Colony Losses: A Mini-Review. Vet Sci. 2020; 7:166.

34. Human H, R. Brodschneider V, Dietemann G, Dively JD, Ellis E, Forsgren I, et al. Miscellaneous standard methods for *Apis mellifera* research. J Apic Res. 2013; 52:1-

53.

35. Insolia L, Molinari R, Rogers SR, Williams GR, Chiaromonte F, Caloviet M. Honey bee colony loss linked to parasites, pesticides and extreme weather across the United States. Sci Rep. 2022: 12:20787.

36. Jaqaman K, Danuser G. Linking data to models: data regression. Nat Rev Mol Cell Biol. 2006; 7:813–819.

37. Johnson SC, Rabinovitch PS, Kaeberlein M. mTOR is a key modulator of aging and age-related disease. Nature. 2013; 493:338-345.

38. Kaur R, Singh D. Molecular markers a valuable tool for species identification of insects: A review. Ann Entomol. 2020; 38:1-20.

39. Knoll S, Pinna W, Varcasia A, Scala A, Cappa MG. The honey bee (*Apis mellifera* L., 1758) and the seasonal adaptation of productions. Highlights on summer to winter transition and back to summer metabolic activity. A review. Livest Sci. 2020; 235: 104011.

40. Kovac H, Stabentheiner A, Brodschneider R. Contribution of honeybee drones of different age to colonial thermoregulation. Apidologie. 2009; 40:82-95.

41. Laplante M, Sabatini DM. mTOR signaling in growth control and disease. Cell. 2012; 149:274-293.

42. Li W, Huang ZY, Liu F, Li Z, Yan L, et al. Molecular Cloning and Characterization of Juvenile Hormone Acid Methyltransferase in the Honey Bee, *Apis mellifera*, and Its Differential Expression during Caste Differentiation. PLoS ONE. 2013; 8:e68544.

43. Livak KJ, Schmittgen TD. Analysis of relative gene expression data using real-time quantitative PCR and the 2(-Delta Delta C(T)) Method. Methods. 2001; 25:402–408.

44. Martin JS. Lipid composition of fat body and its contribution to the maturing oöcytes in *Pyrrhocoris apterus*. J Insect Physiol. 1969; 15:1025-1045.

45. Medrzycki P, Sgolastra F, Bortolotti L, Bogo G, Tosi S, Padovani E, Porrini C.& Sabatini AG. Influence of brood rearing temperature on honey bee development and susceptibility to poisoning by pesticides. J Apic Res. 2010; 49:52-59.

46. Moses AM. Statistical Modeling and Machine Learning for Molecular Biology. CRC Press Taylor & Francis Group. 2017: 280.

47. Münch D, Kreibich CD, Amdam GV. Aging and its modulation in a long-lived worker caste of the honey bee. J Exp Biol. 2013; 216: 1638–1649.

48. Nilsen KA, Ihle KE, Frederick K, Fondrk MK, Smedal B, Hartfelder K, et al. Insulin-like peptide genes in honey bee fat body respond differently to manipulation of social behavioral physiology. J Exp Biol. 2011; 1:1488-97.

49. Patel A, Fondrk MK, Kaftanoglu O, Emore C, Hunt G Frederick K, et al. The making of a queen: TOR pathway is a key player in diphenic caste development. PLoS ONE. 2007; 2: e509.

50. Quinlan GM, Feuerborn C, Hines HM, Grozinger CM. Beat the heat: thermal respites and access to food associated with increased bumble bee heat tolerance. J Exp Biol. 2023; 226: jeb245924.

51. Rodrigues MA, Flatt T. Endocrine uncoupling of the trade-off between reproduction

and somatic maintenance in eusocial insects. Curr Opin Insect Sci. 2016; 16:1e8.

52. Sarioğlu-Bozkurt A, Topal E, Güneş N, Üçeş E, Cornea-Cipcigan M, Coşkun İ, Cuibus L, Mărgăoanet R. Changes in Vitellogenin (Vg) and Stress Protein (*HSP 70*) in Honey Bee (*Apis mellifera anatoliaca*) Groups under Different Diets Linked with Physico-Chemical, Antioxidant and Fatty and Amino Acid Profiles. Insects. 2022; 13:985.

53. Simpson J. Nest Climate Regulation in Honey Bee Colonies. Science. 1961; 133:1327–1333.

54. Smedal B, Brynem M, Kreibich C, Amdam G. Brood pheromone suppresses physiology of extreme longevity in honeybees (*Apis mellifera*). J Exp Biol. 2009; 212: 3795-801.

55. Szentgyörgyi H, Czekońska K., Tofilski A. Honey bees are larger and live longer after developing at low temperature. J Therm Biol. 2018; 78:219-226.

56. Winston ML. The Biology of the Honey Bee. Harvard University Press, Cambridge. 1987; 294.

57. Zhang W, Wang L, Zhao Y, Wang Y, Chen C, Hu Y, et al. Single-cell transcriptomic analysis of honeybee brains identifies vitellogenin as a caste differentiation-related factor. iScience. 2022; 25:104643.

58. Zhang Y, Liu Y, Zhang J, Guo Y, Ma E. Molecular Cloning and mRNA Expression of Heat Shock Protein Genes and Their Response to Cadmium Stress in the Grasshopper Oxya chinensis. PLoS ONE. 2015; 10: e0131244.

59. Zoncu R, Efeyan A, Sabatini DM. mTOR: from growth signal integration to cancer,

Formatted: Indent: Left: -0.14", Line spacing: Double

diabetes and aging. Nat Rev Mol Cell Biol. 2011; 12:21-35.

Formatted: Line spacing: Double

Funding: This work was carried out with the support of the Cooperative Research Program for Agriculture Science & Technology Development (Project No. RS-2023-00232749) and the Priority Research Centers Program through the National Research Foundation of Korea (NRF) funded by the Ministry of Education (2020R1A6A1A03041954). The funders had no role in study design, data collection and analysis, decision to publish, or preparation of the manuscript.

Competing interests: The authors have declared that no competing interests exist.

Abbreviations: DA, discriminant analysis; AHC, Agglomerative Hierarchical Clustering; FD, Fisher distance; HG, hypopharyngeal glands; JH, juvenile hormone; *JHAMT, Juvenile Hormone Acid Methyltransferase; vg, vitellogenin; ilp1, insulin-like peptide 1; ilp2, insulin-like peptide 2; HSP70, heat shock protein 70; TOR1, target of rapamycin 1; RpL32, Ribosomal protein L32.*

Dear Editor-in-Chief, Reviewer 1 and Reviewer 2!

Thank you for the time and effort spent reviewing our manuscript number PONE-D-24-13353 (**The effect of seasonal temperatures on the aging of the overwintered honey bee**) and suggesting some important points to consider.

We corrected the manuscript according to the recommendations in the Submission Guidelines during the final step of our editing process (the Materials chapter was placed after the Introduction, citations in the text were numbered, and the References were updated). As a result, all figure numbers were changed before resubmission. However, we responded to the reviewer's comments using the previous numbering.

Our replies are marked by **green color** after the symbol **R** (reply) and **question numbers** for the reviewer's comments. Also, we copied these modifications in response for convenience when they were available.

Please, find below the reviewer comments **(black**) and our response (**green**)

Reviewer #1: Comments on PLOS ONE ms. "Winter honey bees"

This manuscript describes an experiment designed to determine how elevated brood nest temperature affects the physiology and gene expression of wintering honey bees. I think the design of the experiment and statistical analyses were appropriate. However, I have substantial concerns about some of the wording and failure to address some of the implications of the results. Below I highlight some of my most important concerns. I have placed MANY comments and suggested changes to wording on the manuscript itself.
Despite my many comments, I enjoyed the study. I feel that the authors need to fix some of the glaring issues (i.e. HP glands are relatively undeveloped in wintering bees, not hypertrophied/well developed, as was mentioned at least 3 times), improve the wording, and better discuss the implications of their results with respect to our warming planet.

1. I anticipated that this study would lead to a discussion of how climate change, and specifically warming temperatures, may affect wintering bees. You mention this briefly in the

last sentence of the Introduction. Therefore, I thought this would be the main point brought out in the Discussion. Instead, the Introduction and Discussion largely ignored the possible effects of climate warming on individual wintering bees. Why? **R1.** Thank you for pointing this out. We corrected some parts of the Introduction and Discussion.

Climate change is a complex set of factors that influences honey bee survival through temperature and the amount and quality of melliferous plants, pest and pathogen development, etc. Notably, the season of appearance and the duration of cooling or warming will have different impacts on honey bees and other plants and animals. This topic deserves several dedicated articles in the future.

Our field experiment focused on only one aspect of climate change: warming, characterized by stable, long-term high temperatures during the reported months, including summer. The effect on the overwintering of honey bees was estimated, as overwinter mortality continuously decreases the honey bee population worldwide. This experimental challenge was conducted solely on honey bee colonies to study their response, excluding the effects on plants and other environmental factors outside the rooms. For your convenience, you can review the corrections in the screenshots below.

Screenshot 1 (Introduction)

These factors arising throughout the year include climate warming or cooling (Dosio et al., 2018; Mishra et al., 2023), which imp[illegible]fespan by shortening or extending it respectively in flies (Lamb, 1968), fish (Liu and Walford, 1966), and rodents (Conti et al., 2006). Other factors include the quality of forage (Switanek

Screenshort 2 (Introduction)

2012). Another newly added external process for honey bee development was climate warming which has been shown to significantly increase brood t[illegible]erature in summer (Coulibaly et al., 2022) despite honey bees maintain a consistent brood microclimate (Abou-Shaara et al., 2017; Bencsik et al., 2023).

Screenshot 3 (Results)

25°C and 35°C groups experienced stable, controlled day and night ambient temperatures (modeling climate warming) from June to October, while the room for the Control group was freely ventilated. All honey bees experienced natural foraging outside without restrictions. Newly emerged honey bees were marked in September for the overwintering experiment in February.

Screenshot 4 (Discussion)

particular influence on aging and subsequent winter loss in honey bees. However, due to the complex and unclear interactions among all these triggers, we designed an experiment in which only one trigger, ambient temperature (25, 35°C, and control), was manipulated during the previous summer, September, and October to model the influence of one factor of climate warming: temperature. Colony

Screenshot 5 (Material and Method)

Two rooms equipped with a climate-control system were established to maintain honey bee colonies at 25°C (Group 25) and 35°C (Group 35), simulating climate warming without day and night temperature differences. These temperatures were selected to mimic the effects of climate change, with 25°C representing the summer average and 35°C being the highest recorded in Incheon City under typical conditions. However, the highest temperature was not naturally reached during the summer of 2022 **(Fig 6E).** Other environmental conditions

2. Lines 126-132 verbally describe the ambient temperatures for the three experimental groups. **A)** The Control and 25-degree colonies experienced similar temperatures, but experienced significantly lower temperatures than the colonies in the room kept at 35 degrees. **B)** The authors refer to Fig. 6E for these results—but there is no Fig. 6E. Moreover, Fig. 6E is referred to before Fig 1 in the text—figures must be referred to sequentially (1, 2, 3, …). **C**) Related to this, the next paragraph describes how the brood nest temperature was significantly elevated in the 35 degree group compared to the 25 degree group. However, lines 141-149 of the Results fails to mention the control group! Figs. 1A 1B, 1C should include the control group. **D)** Figure 1E, that compares the numbers of capped brood cells over time, is therefore an enigma—as the 25 and 35 degree groups have similar numbers, but different from the Control, but the Control

and 25 degree groups experienced similar temperatures in their experimental settings (except in the month of October). This odd result is never mentioned or discussed. **R2.** Thank you for this questions. We divided the points for the response into A) ~ D) for convenience. **A)** The difference in temperatures for the honey bees from the Control group and the 25°C group was due to day-night fluctuations, which were absent in the latter group. On average, significant temperature differences were not detected. However, a stable elevated night temperature is one of the factors of climate warming that was assessed in this research. We added this explanation to the paragraph "Dynamics of ambient temperature in summer and fall months". **B)** This concerns the experimental design (Fig. 6E), which is detailed in the Materials and Methods section, following the Results section according to PLOS ONE guidelines. Due to the complexity of this numbering, we have removed this reference. **C)** Because of ambient temperature and colony performance (Fig 1 D, E, F) of honey bees from Group control and 25 at the beginning of the measurement (22.08.08) had no significant differences, sensors were not set up in honey bee colonies from Group control. Corrected in manuscript (see Screenshot). **D)** Honey bees, as social insects, have mechanisms for warming or cooling their nest to regulate the temperature around the brood, reducing the effect of ambient temperature on brood rearing. Despite the different ambient temperatures, similar numbers of capped brood areas in honey bee Groups control, 25 and 35 were observed in August and September 8. This indicates successful brood temperature regulation for colony development by the bees until September 18, when hidden problems from the summer were revealed through comparison with the control group. The only factor of climate warming considered here is temperature. Continuously stable ambient temperatures of 25°C and 35°C over 3 months suggest stress due to the lack of day-night temperature fluctuations and wind-cooling effects. This could weaken the colony in the fall, but it might not be a significant problem in the summer if food is sufficient. We added clarification to the Results.

Brood microclimate and colony development in August and September

In this study, we evaluated the performance of the colony using temperature, humidity, CO_2, number of larvae, capped brood area, and honey bees' measurements from August 08 to September 18, 2022, when short-lived honey bees commenced rearing larvae that would develop into long-lived honey bees for the overwintering period. Because the expected ambient temperature in August and early September and the colony performance (Fig 1 D, E, F) of honey bees from the Groups control and 25°C at the beginning of the measurement (22.08.08) showed no significant differences, sensors were not set up in the honey bees from the Control group. We hypothesized that the regulation of the nest climate inside colonies influences the physiology of winter honey bees and the success of overwintering.

SNBL_GY 몇 초 전
서식 있음: 들여쓰기: 왼쪽 4 글자

SNBL_GY
서식 있음: 글꼴: (영어) Times New Roman, 14 pt

SNBL_GY
서식 있음: 글꼴: (영어) Times New Roman, 14 pt

SNBL_GY 몇 초 전
삭제됨:

3. Line 48-49: You state: "it is necessary to elucidate the processes involved in the transition from short-lived workers to long-lived ones." This is a valid point, but it is not the focus of your research! Therefore, it creates an expectation in the reader of what you investigated, but later you explain that you are really interested in factors operating in fall that affect the longevity of workers during winter. **R3.** Thank you for this question. Corrected.

To advance in this direction, it is necessary to clarify the known processes involved in the transition from short-lived workers to long-lived ones in the late summer and fall seasons. This transition has been associated with external (environmental) factors that lead to internal (metabolic) processes.

삭제됨: elucidate

SNBL_GY
삭제됨: the

4. It is not clear why you chose to capitalize "Acinus" and "Fat body" throughout the manuscript. I see no reason to do so. **R4.** Thank you for this question. Corrected.

5. The use of the term "honey bee" throughout the paper. It is clear from the Introduction (lines 103-104) of the paper that you studied honey bees, Apis mellifera. You may want to state that again at the beginning of the Methods and Results, but in many many locations you can replace "honey bee" with "bee" or "bees", or even in some places to reduce repetition with "it" or "them" (singular/plural). **R5.** Thank you for this question. Corrected.

6. "Acinus" is singular. In honey bees, an acinus is a single globular structure of the HP gland. The hypopharyngeal gland is composed of many grape-line "acini"- plural. You need to check through the paper to determine which mentions of "Acinus" should remain "acinus" (singular),

and which mentions refer to multiple structures and should be referred to as “acini” (plural). **R6**. Thank you for this question. Corrected.

7. Line 63-64: The authors have written that the winter bee phenotype is characterized by “increased Acinus size (hypopharyngeal gland, HP)”. This statement is false. Refer to Fig. 2 in Doke et al., a paper you cited. Similar incorrect statements about larger HP glands in winter bees appears in Line 249 and Line 256.
You may also want to cite:
Wang K., Liu Z.-G., Pang Q., Zhang W.-W., Chen X.-M., Fan R.-L., et al. (2018). Investigating the regulation of hypopharyngeal gland activity in honeybees (Apis mellifera carnica) under overwintering conditions via morphologic analysis combined with iTRAQ-Based comparative proteomics. Ann. Entomol. Soc. America 111 (3), 127–135. 10.1093/aesa/say012 **R7.** Thank you for this question. We fixed this inaccuracy and changed "increased" to "hypertrophied" (as in Reference 1) (see in Screenshot 1, 2 and 3), because it used in literature (Knoll et al., 2020). The measurement of the hypopharyngeal gland (HG) includes different parameters, such as the morphology of the HG (area, diameter, width, and length of acini) and the ultrastructure (development of secretory vesicles inside acini). In the HG of winter bees (sampled in November), secretory vesicles were visible, similar to those in 3-day-old bees, which were absent in newly emerged summer bees (Deseyn and Billen, 2005, Reference 2). They also found that the diameter of the acini in winter bees falls within the range of that in 3-day-old bees and was higher than in forager bees. Therefore, the term "hypertrophied" is more appropriate than "increased". As recommended in the article you suggested, Wang et al. (2018) reported the transition of HG development at the end of winter through ultrastructure research (development of secretory granules) by comparing honey bees sampled on February 15th and January 8th, without including data on summer honey bees. Consequently, this gland remains hypertrophied, as described by Cruz-Landim and Hadek (1969). However, the diameter of the acini in winter bees is not as large as in 6-12-day-old nurse summer bees (Deseyn and Billen, 2005).

Reference1

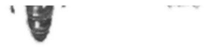

**Family:**	*Apidae*
**Genus:**	*Apis*
**Species:**	*Mellifera*

Sterile female	Fertile female	Male
Short oval abdomen	Long oval abdomen	Short spheroidal abdomen
Long wings	Short wings	Long wings
	"Spider-like" legs	

Fig. 1. Illustration of the different casts of *Apis mellifera*. The green dot on the back of the thorax is used to identify the queen of 2019 within the colony, according to the international queen marking color.

lifespan of 25–40 days, winter bees have been shown to be able to live for over 250 days (Mattila et al., 2001; Amdam and Omholt, 2002; Döke et al., 2015; Kunc et al., 2019; Yamada et al., 2019). Tasks of these long-lived winter bees include regulating in-hive temperatures during winter and the initiation of brood rearing in winter and early spring (Mattila et al., 2001; Mattila and Otis, 2007; Döke et al., 2015; Eyer et al., 2017; Kunc et al., 2019).

Besides the evident difference in lifespan, winter bees show pronounced physiological variations compared to summer bees. Overall, winter bees have substantially lower juvenile hormone (JH) titers (Huang and Robinson, 1995; Mattila and Otis, 2007; Döke et al., 2015; Kunc et al., 2019). JH is mostly associated with foraging activities of summer workers and is inversely related to in-hive activities (Huang and Robinson, 1995; Döke et al., 2015). Second, winter bees are known to hold hypertrophied hypopharyngeal glands (HPGs), glands which play a key function in food production (royal jelly) for brood rearing. Next, long-lived winter bees have enlarged fat bodies due to the heavy accumulation of nutrients (lipids and protein) (Mattila et al., 2001; Amdam and Omholt, 2002; Mattila and Otis, 2007; Döke et al.

rearing once more (Nürenberger et al., 2018; Ricigliano et al., 2018).

The high increase in colony size, following the spring rise, typically leads to swarming, where the majority of workers leave the colony together with the queen. The swarm will venture out in search of a new home, leaving behind a new queen with a limited number of workers who will rebuild the old colony (Döke et al., 2015).

From the onset of spring until late fall, bee colonies deploy a large workforce dedicated to the collection of food supplies. As long as the ambient conditions allow it, forager bees will venture out of the hive in search of pollen and nectar. Pollen, the main protein and lipid source of honey bees (Mattila and Otis, 2007; Di Pasquale et al., 2013; Döke et al., 2015), is collected and immediately stored in designated cells for later consumption by young bees (Toth et al., 2005). In turn, nurse bees will convert this protein rich meal into jelly, secreted through their HPGs as feed for the queen (for this reason called royal), the present larvae and other worker bees (Crailsheim, 1991; Di Pasquale et al., 2013). Nectar, used as a general energy source by bees (Döke et al., 2015), is first collected by foragers before being passed on to in-hive bees through trophallaxis (the exchange of liquids

Knoll S, Pinna W, Varcasia A, Scala A, Cappa MG. The honey bee (*Apis mellifera* L., 1758) and the seasonal adaptation of productions. Highlights on summer to winter transition and back to summer metabolic activity. A review. *Livest Sci*. 2020; 235: 104011.

Reference 2

Ultrastructure of the hypopharyngeal gland 53

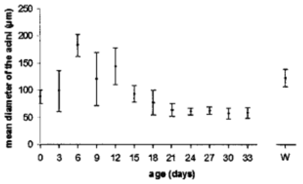

Figure 3. Mean diameter of the acini in function of the age of workers (days). W: winter bees (N = 3 for each category).

They accumulate and are most abundant at an age of 24 days (Fig. 5B). Other organelles disintegrate and the surrounding cytoplasm is less dense. Probably, these structures break down by active substances of the lysosomes.

From an age of 27 days, the RER is electron dense and completely reticular (Figs. 5C, D). Distinction between different structures and organelles becomes more difficult.

In winter bees, 2 types of vesicles with different electron densities are visible (Figs. 5E, F). The more electron dense vesicles are smaller in size, randomly distributed within the cytoplasm and have a diameter between 1–3 µm. The clear masses around the end apparatus correspond with the secretion in summer bees and probably are royal jelly. There is still production of secretory vesicles, but no active discharge. We assume that both types of secretion are stored until spring. As a consequence, this gland stays hypertrophied as described by Cruz-Landim and Hadek (1969). The diameter of the acini in winter bees is not as big as in summer bees.

This species exhibits a similar secretory cycle to the one we describe here for the honeybee.

The structure of the hypopharyngeal gland we report is in agreement with earlier descriptions (Cruz-Landim and Hadek, 1969), however, we also noticed pronounced age-dependent differences, which were revealed through anatomical measurements and light- and electron-microscopy.

In summer bees, the size of the acini changes with age. The peak size is found around 6 days of age, when workers are known to feed the larvae with royal jelly (Hrassnigg and Crailsheim, 1998). From the age of 15 days, we observed a decrease in size of the acini. The size of the glands in foragers corresponds with that of the still undeveloped gland of newly emerged bees. This was also observed by Simpson et al. (1968). Therefore, size of the gland is positively correlated with gland activity.

The amount of secretion in the secretory cells is also positively correlated with size of the acini. When the worker becomes a forager, the gland no longer has a food-producing function. This partially explains the decrease of size as illustrated by the metric results, but also the cytoplasmic structures start to disintegrate.

Organelles modify with age, such as the RER. The endoplasmic reticulum is almost vesicular and becomes more reticular. This situation is also seen in a *Melipona* species (Cruz-Landim et al., 1987).

The amount of secretion clearly changes. We observed a secretory cycle, reflected by the occurrence and amount of secretion around the end apparatus, which reaches a peak value around the age of 6 days. These secretory products probably correspond with the royal jelly, which young bees at this age produce in vast amounts for nursing the larvae (Snodgrass, 1956). Halberstadt (1980) described 2 phases

Deseyn J., Billen J. 2005. Age-dependent morphology and ultrastructure of the hypopharyngeal gland of *Apis mellifera* workers (Hymenoptera, Apidae). *Apidologie* 36: 49-57.

Screenshot 1: correction done

requires a pure carbohydrate diet (Brejcha et al., 2023). In winter honey bees, the physiology of this hormone-regulated phenomenon is well-described, manifesting as hypertrophied secretory vesicles and acini diameter in the hypopharyngeal gland (HG) compared to newly emerged and forager summer bees (Deseyn, Billen, 2005). It also includes increased fat body mass and

Screenshot 2 (line 249 in initial manuscript): the comparison is relevant to our research, where one area is larger than the other.

2010). In our research, acinus area in honey bee Groups 25 and the control were significantly larger ($p < 0.05$) than those in honey bee Group 35. The

Screenshot 3 (line 256 in initial manuscript): we explained this point of view more deeply because it is a citation.

The physiological markers of long-lived (winter) honey bees include hypertrophied HG because the acini include secretory vesicles with a diameter of the acini in winter bees falls within the range of that in 3-day-old bees and is higher than in forager bees which plays a key role in royal jelly production for larval rearing in spring (Deseyn and Billen, 2005). Another physiological

8. Lines 116-121: I feel that these sentences provide too much of the results. In the Introduction it is OK to briefly mention what the results are, in general terms, but this level of explanation of results seems excessive. **R8.** Thank you for this question. We reduced the explanation of the results there.

9. Statistical reporting: I have always referred to “Duncan’s post hoc test”. Not “Duncan”. I did an internet search and several sites also refer use the possessive noun: “Duncan’s”.
It is good to explain that SD refer to Standard Deviation the first time you use it in the text and in a figure legend. I do not think you need to write out “SD - Standard Deviation” repeatedly in the text and figure legends. **R9.** Thank you for this question. Corrected.

10. Number of significant digits to report: There are rules for how many significant digits one should report. It depends on the accuracy of the measurements as well as the number of possible values for the variable. Reporting mean temperature as 29.995 ± 0.982 °C is not correct. This should be either 30.0 °C or 30.00 °C. Please seek advice on significant digits and then check and correct all values reported in the manuscript. **R10.** Thank you for this

question. We checked the SAMPL guidelines for general guidance on statistical reporting in PLOS ONE (screenshot attached). We corrected the issue. We used the 30.00 °C format in this chapter, with a total of 4 digits.

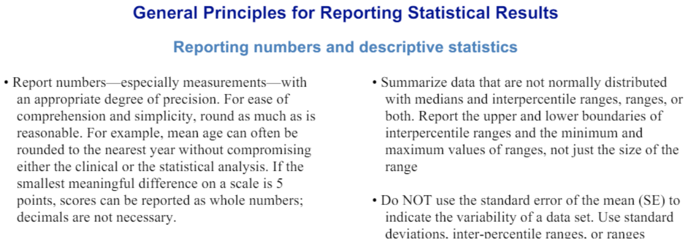
General Principles for Reporting Statistical Results

Reporting numbers and descriptive statistics

- Report numbers—especially measurements—with an appropriate degree of precision. For ease of comprehension and simplicity, round as much as is reasonable. For example, mean age can often be rounded to the nearest year without compromising either the clinical or the statistical analysis. If the smallest meaningful difference on a scale is 5 points, scores can be reported as whole numbers; decimals are not necessary.
- Summarize data that are not normally distributed with medians and interpercentile ranges, ranges, or both. Report the upper and lower boundaries of interpercentile ranges and the minimum and maximum values of ranges, not just the size of the range
- Do NOT use the standard error of the mean (SE) to indicate the variability of a data set. Use standard deviations, inter-percentile ranges, or ranges

11. Check the definitions of “weight” vs. “mass”. I believe you will find that you should refer to “fat body mass”**. R11.** Thank you for this question. Corrected.

12. Lines 152-154: Interpretations of data belong in the Discussion—not in the Results. **R12.** Thank you for this question. Corrected.

13. Line 155: Heading: “Molecular marker recognition in overwintered western honey bees”. I believe you determined “gene expression of molecular markers” And acinus size and fat body mass are not “molecular markers”. You need to reword this heading. **R13.** Thank you for this question. Corrected. For convenience, you can review the corrections on the screenshot below

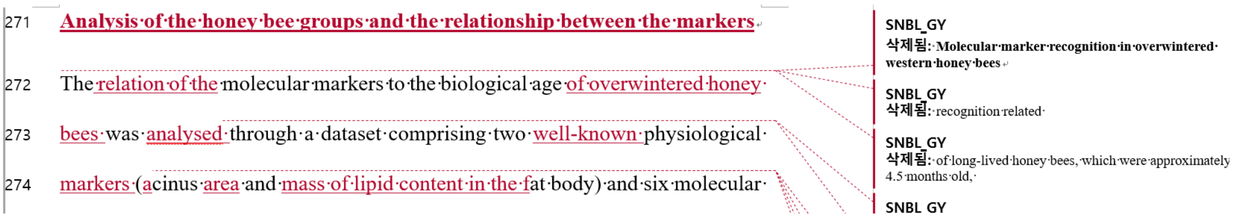
Analysis of the honey bee groups and the relationship between the markers

The relation of the molecular markers to the biological age of overwintered honey bees was analysed through a dataset comprising two well-known physiological markers (acinus area and mass of lipid content in the fat body) and six molecular

SNBL_GY
삭제됨: **Molecular marker recognition in overwintered western honey bees**

SNBL_GY
삭제됨: recognition related

SNBL_GY
삭제됨: of long-lived honey bees, which were approximately 4.5 months old,

SNBL GY

14. Figure 2: Because red-green color blindness is very common, particularly in men of European descent, one should never use red and green together in figures. This red and green graph will NOT be comprehensible by a number of readers. A different color scheme is needed. **R14.** Thank you for your question. Since this disability can affect the perception of red, green, and blue, we used yellow and blue scales to redraw the figure. We also corrected Figure 1 by changing the red line to a dashed line. For convenience, you can review the corrections in the screenshot below.

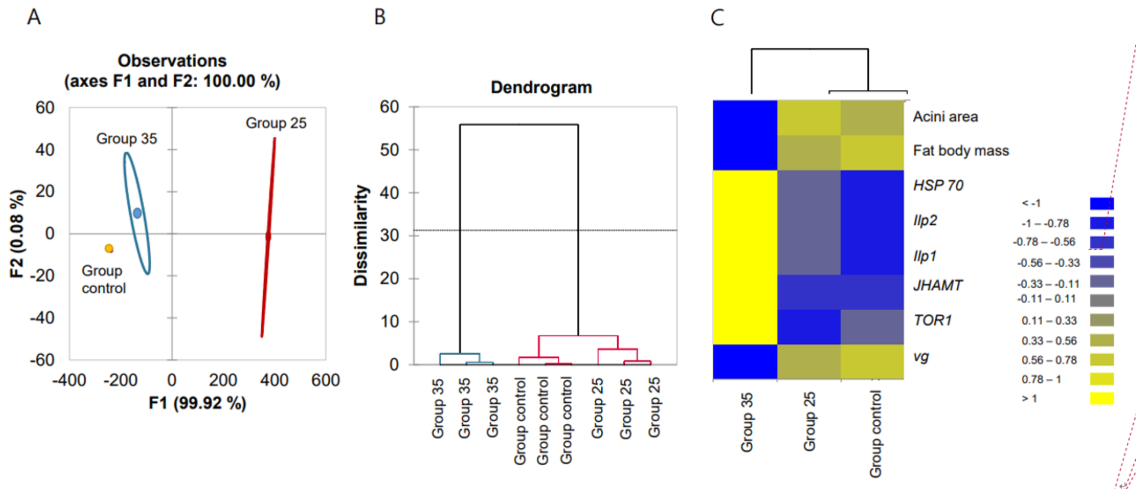

Fig 2. The discrimination of overwintered honey bees was performed using eight variables

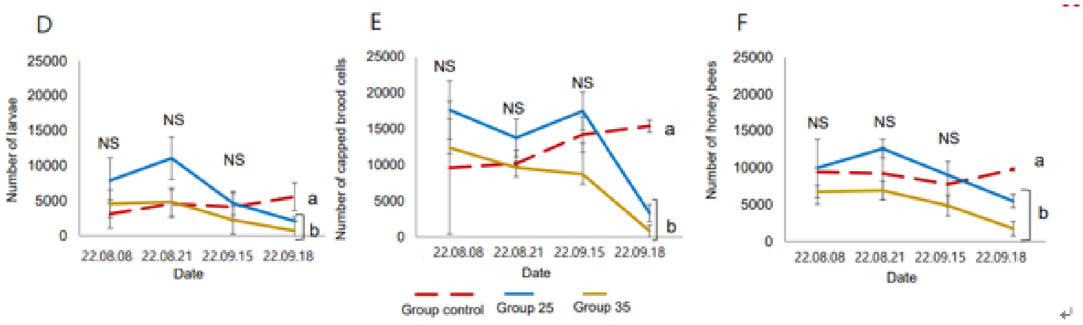

Fig 1. Colony performance in honey bees treated under different ambient temperatures (08

15. Line 247: You state that the bees in Groups 25 and 35 successfully overwintered. I have seen no data in the manuscript that supports that statement. There had to be some bee mortality over the 3.5 months. What percentage of marked bees survived to the date when they were sampled? Did you collect that data? (You may be referring here to colony survival, but that is not as relevant to your study as individual bee survival.) **R15.** Thank you for this question. Survival of honey bee colonies estimated as overwintering loss (YES\NO) in this case at the end of February and their developmental problem in spring (YES\NO). We added this table to the Supplementary material (S1 Table). All colonies got the "NO" that we reported in the manuscript because our target problem overwintering loss. Counting the percentage of marked bees in the middle and end of February under field conditions is not possible due to the low ambient temperatures in Korea, which can cause colonies to overcool and die. For convenience, you can review the corrections on the screenshot below

S1 Table. Survival of honey bee colonies at the end of the overwintering.

Group	control			25			35		
Replication	1	2	3	1	2	3	1	2	3
February 5th	+	+	+	+	+	+	+	+	+
February 27	+	+	+	+	+	+	+	+	+

+ Survival

16. Lines 247-248: I have placed two important comments on the manuscript related to this sentence. **R16.** Thank you for this question. We corrected this part of discussion.

Reviewer #2: Review "The effect of seasonal temperatures on the aging of the overwintered honey bee"

Frunze et al. investigate the effect of winter temperature experienced by larvae on aging markers in workers. To do so, hives are kept at two different temperatures plus a control treatment (natural conditions) over summer and fall. In winter workers of the hives of the different treatments are collected, fatbody size, acinus size and the expression of several aging associated candidate genes are investigated to make inferences on the putative effect of climate warming on aging in winter bees. The aim and topic of this experiment are highly relevant and interesting. At the moment, there are several parts and aspects that need clarification and correction. The authors should also discuss the interplay and the meaning of the various factors, from treatment to measured variables, more clearly.

Major comments

17. -I understand that you collected several candidate genes that have been shown to be associated with aging and you test these in the fatbody. It does not become clear in the discussion whether all these candidates change their expression in the expected direction. Do they differ and if yes how, and what is your interpretation of that? **R17.** Thank you for this question. We added more discussion about this.

18. -Why did you choose to investigate Vg gene expression in the fatbody, and the expression of all other candidates in the head? **R18.** Thank you for this question. We corrected this part of the Introduction. Please, check lines 122-124, 743-745 on the screenshots below.

relationship is not yet clear (Schilcher and Scheiner, 2023). It is known that vg is synthesized in the fat body of workers and is positively correlated with HG development throughout their lifespan (Dohanik et al., 2024). The JH biosynthetic pathway starts in the corpora allata glands in the brain complex and responds to sensory inputs that form the basis for the division of labor (Scheiner

were dissected on ice, and RNA was extracted directly. RNA was extracted from the brain for the analysis of *HSP70, JHAMT, ilp1, ilp2*, and *TOR1* expression, and from the abdomen for *vg* gene expression analysis, as this gene is expressed in the fat body located in the abdomen. Genes are measured in the brain to understand their roles in stress responses (*HSP70*), metabolic regulation (*JHAMT* and *TOR*), and neural function (*ilp1* and *ilp2*).

19. -Why did you measure fatbody and acinus size?

=> the latter three points could be made clear in the introduction, but latest in the results **R19.** Thank you for this question. We corrected this part of the Introduction. Please, check lines 114-116 on the screenshots below.

Screenshot

requires a pure carbohydrate diet (Brejcha et al., 2023). In winter honey bees, the physiology of this hormone-regulated phenomenon is well-described, manifesting as hypertrophied secretory vesicles and acini diameter in the hypopharyngeal gland (HG) compared to newly emerged and forager summer bees (Deseyn, Billen, 2005). It also includes increased fat body mass and vitellogenin (protein vg, gene *vg*) levels, alongside decreased levels of the systemic endocrine factor juvenile hormone (JH) compared to summer bees (Fluri et al., 1977; Döke et al., 2015; Knoll et al., 2020). This mutually inhibitory interaction between vg and the JH hormone distinguishes honey bees from other insects (Amdam et al., 2012), and the molecular mechanism of this complex relationship is not yet clear (Schilcher and Scheiner, 2023). It is known that vg is synthesized in the fat body of workers and is positively correlated with HG

SNBL_GY 삭제됨: us
SNBL_GY 삭제됨: size
SNBL_GY 삭제됨: (
SNBL_GY 삭제됨: ,
SNBL_GY 삭제됨: ,
SNBL_GY 삭제됨: F
SNBL_GY 삭제됨: weight
SNBL_GY 삭제됨: ,
SNBL_GY 서식 있음: 글꼴: 기울
SNBL_GY 삭제됨: levels

20. -What was the mean temperature of the control treatment in the hive, and how does it relate to the two treatment temperatures? **R20**. Thank you for this question. I believe you are referring to ambient temperature. The plot showing the dynamics of ambient temperatures in control and

experimental colonies is presented in Figure 6E. Below on screenshot, we explained why these temperatures were chosen.

These temperatures were selected to mimic the effects of climate warming, with 25°C representing the summer average and 35°C being the highest recorded in Incheon city under typical conditions. However, the highest temperature was

21. -The treatment temperatures differ by 10C, however, the in-hive difference is only 3C. This should be mentioned and discussed somewhere, also in respect to the control. This is especially relevant to be able to interpret why gene expression was only different to the 35C treatment. **R21.** Thank you for this question. Corrected.

the end of August **(Fig 1)**. The total difference in ambient temperatures between the controlled honey bee groups, initially 10°C, was reduced to 3.403°C due to the heating of the brood area by honey bees in Group 25 and cooling by honey bees in Group 35 demonstrating known microclimate regulation (Abou-Shaara et al., 2017; Bencsik et al., 2023). Because of the

22. -I find it highly interesting that the variables measured for Fig1D-F differ between the control and both treatments but later gene expression differs between 35C and the other two. Add this finding to the discussion and add your explanation for this pattern. **R22.** Thank you for this question. We added this points in Discussion.

23.-Results of the statistical test need to be better depicted and also mentioned in full in the text. **R23.** Thank you for this question. Corrected.

See more details below.

Minor comments

Abstract

24. rephrase the first sentence. **R24.** We appreciate the reviewer's valuable comments and have rephrased the first sentence of the Abstract. For your convenience, you can review the corrected lines (21–23) in the screenshot below in the next response, R25.

25. Line 23: Be more concise. Temperature is not the only factor reported to affect winter bee longevity, it would thus be good to at least write that it is ONE factor affecting longevity and thereby that other factors may also play a role. Or also mention other factors putatively affecting longevity in bee workers. Be more specific in the abstract on what was in the treatment and who was tested later. **R25.** Thank you for bringing this to our attention. We fixed this inaccuracy in the sentence (lines 23 – 25). For convenience, you can review the corrections on the screenshot below

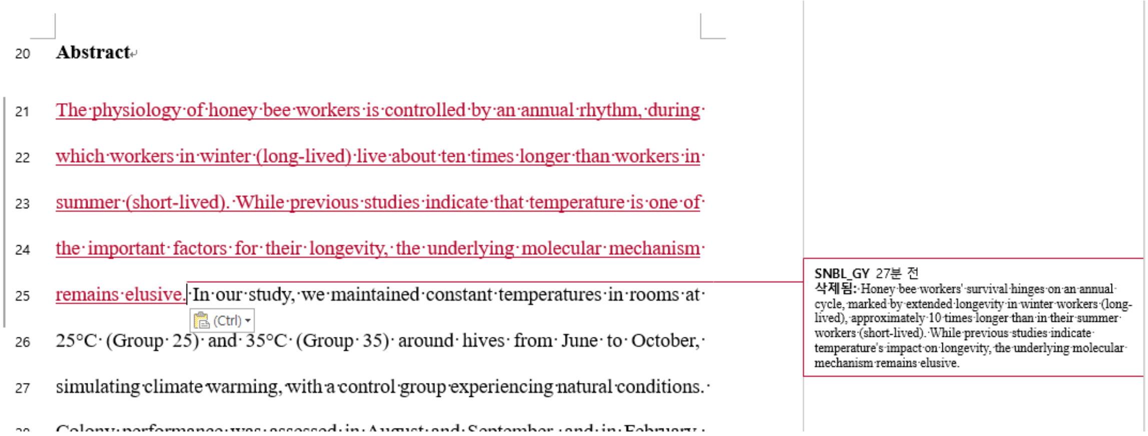
Abstract

The physiology of honey bee workers is controlled by an annual rhythm, during which workers in winter (long-lived) live about ten times longer than workers in summer (short-lived). While previous studies indicate that temperature is one of the important factors for their longevity, the underlying molecular mechanism remains elusive. In our study, we maintained constant temperatures in rooms at 25°C (Group 25) and 35°C (Group 35) around hives from June to October, simulating climate warming, with a control group experiencing natural conditions.

(Ctrl)

SNBL_GY 27분 전
삭제됨: Honey bee workers' survival hinges on an annual cycle, marked by extended longevity in winter workers (long-lived), approximately 10 times longer than in their summer workers (short-lived). While previous studies indicate temperature's impact on longevity, the underlying molecular mechanism remains elusive.

Introduction

26. Line 41: maybe put emphasis first on their role as pollinators and honey production and then production of health and medical products. **R26.** We appreciate the reviewer's valuable comments and have rephrased the first sentence of the Introduction. For your convenience, you can review the corrected lines (47–49) in the screenshot below.

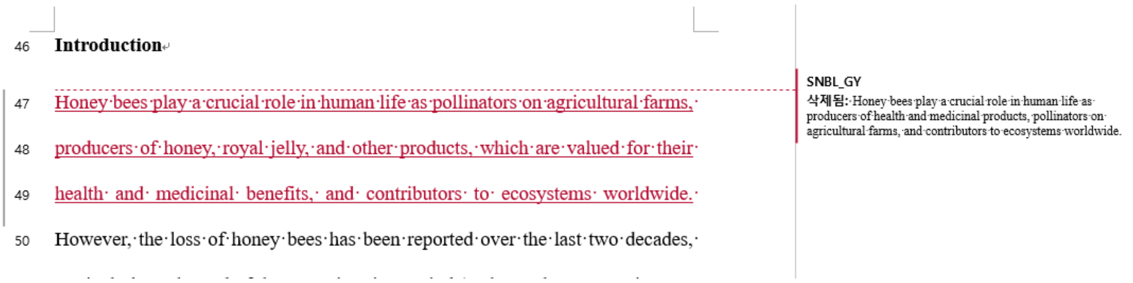
Introduction

Honey bees play a crucial role in human life as pollinators on agricultural farms, producers of honey, royal jelly, and other products, which are valued for their health and medicinal benefits, and contributors to ecosystems worldwide. However, the loss of honey bees has been reported over the last two decades,

SNBL_GY
삭제됨: Honey bees play a crucial role in human life as producers of health and medicinal products, pollinators on agricultural farms, and contributors to ecosystems worldwide.

27. Line 45: be more specific and add information on the "various factors" that may contribute to winter mortality. Plus maybe indicate that there are factors that contribute to strong or abnormal winter mortality. Because even under normal conditions winter bees will die and be

replaced by spring workers...**R27.** Thank you for bringing this to our attention. Included to Introduction

et al., 2023).

Several factors arising during the year can contribute to this (Calovi et al., 2021). For example, climate warming or cooling (Dosio et al., 2018) impacts lifespan in flies (Lamb, 1968), fish (Liu and Walford, 1966), honey bees (Mishra et al., 2023), and rodents (Conti et al., 2006). Other factors related to climate change include the quality of forage (Fahrenholz et al., 1989; Switanek et al., 2017), beekeeper management practices (van Dooremalen et al., 2012; Morawetz et al., 2019), and pesticide exposure from land use (Switanek et al., 2017). Although we can try to avoid some of these factors, effective methods to mitigate the impacts of climate change and prevent honey bee winter mortality are currently lacking.

To advance in this direction, it is necessary to clarify the general processes occurring in late summer and fall that are involved in the transition from short-lived to

SNBL_GY
서식 있음: 글꼴

SNBL_GY
서식 있음: 왼쪽

SNBL_GY
삭제됨: Variou
mortality, and cu
prevent it.

SNBL_GY
서식 있음: 왼쪽

SNBL_GY
삭제됨: elucidat

SNBL_GY
삭제됨: the

SNBL_GY
삭제됨: workers

SNBL_GY
삭제됨: ones

SNBL_GY
삭제됨: with

28. Line 53: delete "seems" **R28.** Thank you, we deleted this word.

29. Line 66/67: what do you mean with "unlike"? Do other insects need diet at all or other dietary composition? **R29.** Thank you for pointing this out. Winter activity is a distinguishing feature of diapause honey bees compared to wasps, bumblebees (eusocial insects where only the queen overwinters), and other non-social insects that overwinter in the adult stage. Usually, diapause means metabolic suppression and insects (excluding honey bees) do not need to move or eat until spring (Denlinger, 2023). This is a special topic for discussion, and we do not have space to deeply explore diapause in insects generally. We have added some corrections to sentences for readers to easily follow the research. For your convenience, you can review the additions in the screenshot below.

Unlike other insects, the reduced metabolic activity of honey bees in diapause (Denlinger, 2023) includes vibrating their wing muscles to generate heat and keep warm inside the winter cluster. This depends on a constant energy source and requires a pure carbohydrate diet (Brejcha et al., 2023). In honey bees, physiology of this hormone-regulated phenomenon is well-described, manifesting as increased Acinus size (hypopharyngeal gland, HG), Fat body weight, and

SNBL_GY 삭제됨: ·

Denlinger DL. Insect diapause: from a rich history to an exciting future. J Exp Biol. 2023; 226 (4): jeb245329.

30. Line 73/74: "Acinus" and "Fat" should be written in lower letters. **R30**. Thank you for this question. Corrected.

31. Line 77: what do you mean by "behavioral activities"? Why "inevitably" leading to reduced longevity and increased aging? **R31**. Thank you for this question. We believe "behavior" is a better term than "behavior activity," and "inevitably" was a superfluous word that was deleted during the correction. We added explanation briefly in six lines before this summarized sentence. Additionally, we avoided repetition and didn't extend the explanation to maintain the focus on the idea of the research, as this information is well-described for honey bees (Prado et al., 2020).

Reference for response:

Prado A, Requier F, Crauser D, Le Conte Y, Bretagnolle V, Alaux C. 2020 Honeybee lifespan: the critical role of pre-foraging stage. R. Soc. Open Sci. 7: 200998.

32. Please also add information on the roles and functions of JH and Vg as you do for the other candidate genes. **R32.** Thank you for this question. Added. For your convenience, you can review the additions in the screenshot below.

Introduction

other diapausing insects and impacts internal processes.

In winter bees, the physiology of internal processes related to this hormone-regulated phenomenon is well-described. It manifests as hypertrophied secretory vesicles and increased acini diameter in the hypopharyngeal gland (HG) compared to

4

SNBL_GY
삭제됨: …n winterhoney…bees, the physiology o… processes relatedthis…to this hormone-regulated is well-described.,…It manifestsing…as hypertroph… increased …ecretory vesicles and increased A…cinius…diametersize…(…n the hypopharynge…

newly emerged and forager summer bees (Deseyn and Billen, 2005). HG development is known to be positively correlated with vitellogenin (*vg*) throughout a worker's lifespan. However, *vg* is synthesized in the fat body (Dohanik et al., 2024), which is more developed in winter bees compared to summer bees (Brejcha et al., 2023). The fat body mass results from the accumulation of nutrients (Smedal et al., 2009), which lipids constituting over >90 % of the mass in the form of triglycerides. These triglycerides are synthesized from dietary carbohydrates and fatty acids and are used not only for *vg* synthesis (Smedal et al., 2009) but also for producing pheromones, phospholipids, and wax (Arrese and Soulages, 2010).

SNBL_GY
삭제됨: ,
SNBL_GY
서식 있음: 글꼴: 기울임꼴
SNBL_GY 2024년 July 29일
삭제됨: Fat body weight
SNBL_GY
삭제됨: ,
SNBL_GY
서식 있음: 글꼴: 기울임꼴
SNBL_GY
서식 있음: 글꼴: 기울임꼴
SNBL_GY
삭제됨: and vitellogenin (vg)
SNBL_GY
서식 있음: 글꼴: 기울임꼴
SNBL_GY

Discussion

All of this closely associated with molecular markers (Alaux et al., 2018; Kaur and Singh, 2020; Daniels et al., 2023), especially *vg* which is synthesized in the fat body (Smedal et al., 2009; Amdam et al., 2012). Vg is then transferred to the HG and used as an amino acid donor for the synthesis of royal jelly to feed larvae after diapause ends (Ramanathan et al., 2018). Along with elevated *vg* levels, another molecular marker, suppressed juvenile hormone (JH) levels, is used to quantify the condition of winter honey bees compared to summer) honey bees (Amdam and Omholt, 2002; Döke et al., 2015; Knoll et al., 2020). These molecular levels remain stable during diapause and undergo a switch before the onset of brood rearing (Amdam and Omholt, 2002). Notably, *vg* gene expression was significantly higher in honey bees from Group 25 and the control, whereas *JHAMT* gene expression was significantly lower compared to Group 35, suggesting an aging process in the latter bees.

SNBL_GY
삭제됨: , suggesting This process is
SNBL_GY
삭제됨: .
SNBL_GY
서식 있음: 글꼴: 기
SNBL_GY
서식 있음: 글꼴: 기
SNBL_GY
삭제됨: In long-live… include elevated vg l… et al., 2009) in compa…
SNBL_GY
삭제됨: short-lived (
SNBL_GY
삭제됨: honey bees f…
SNBL_GY
삭제됨: which also
SNBL_GY
삭제됨: s
SNBL_GY
삭제됨: honey
SNBL_GY
서식 있음: 글꼴: 굵
SNBL_GY

33. Line 96: "longevity" please be careful with using aging and longevity interchangeably. Longevity is basically the end result of the aging process; they are highly related but not the same. **R33.** Thank you for this question. We rechecked the manuscript for any mistakes in meaning, as we intended the same like you meaning for these words.

34. Line 102: you might want to add some information on why and how temperature may affect lifespan. **R34.** Thank you for this question. We have added this sentence to lines 57–60 and included the cited articles in the References. For your convenience, you can review the additions in the screenshot below.

Several arising during the year factors can interfere with bees' ability to overwinter, regulate their body temperature during winter, and maintain the lifespan of winter bees, and gather and store sufficient food during summer and fall, all of which contributing to honey bee winter mortality (Calovi et al., 2021). These factors arising throughout the year include climate warming or cooling (Dosio et al., 2018; Mishra et al., 2023), which impact lifespan by shortening or extending it respectively in flies (Lamb, 1968), fish (Liu and Walford, 1966), and rodents (Conti et al., 2006). Other factors include the quality of forage (Switanek

SNBL_GY 4시간 전
서식 있음: 들여쓰기: 첫 줄: 1 글자

35.Line 107: What exactly does "maintain" mean? Was the control group located outdoors or were outdoor conditions "simulated". **R35.** Thank you for this question. This sentence has been rephrased, and the word “maintain” has been deleted. For your convenience, you can review the correction in the screenshot below.

temperatures in Incheon (Republic of Korea), respectively, while Group control was kept under natural conditions. The temperature experiments lasted

SNBL_GY
삭제됨: were maintained in the Group control

36.Line 117: "winter worker broods" unclear whether you investigated workers or brood. So far I understood that the aim was to investigate workers but here it sounds like it was brood? But then for Group35 you only talk about workers... Please phrase more clearly. => after reading the manuscript I now understand what you mean and it makes sense. Maybe consider writing "brood resulting in winter workers" or something like that? **R36.** Thank you for this question. These words (winter worker brood) have been replaced (brood resulting in winter workers) for clear meaning. For your convenience, you can review the correction in the screenshot below.

markers related to the task (*vg* and *JHAMT*), nutrition (*ilp1*, *ilp2*, and *TOR1*), and stress response (*HSP70*). Our findings suggest that temperature decreases around brood resulting in winter workers from Group 25 in the fall lead to their slower physiological aging in winter compared to Group 35 workers, supported by

SNBL_GY
삭제됨: winter worker broods

37. Line 120: HSP70 and TOR1 may serve as additional markers for what? Please clarify. And what kind of research is additionally needed? **R37.** Thank you for this question. We have added clarification to the sentence about this. For your convenience, you can review the correction in the screenshot below.

physiology and task-related markers. Furthermore, we observed a relation between aging and *HSP70* and *TOR1* genes, which may serve as additional markers of aging and necessitate further research using other animal models. This approach could enhance the molecular method for identifying aging in insect models, thereby advancing research related to climate warming.

SNBL_GY
삭제됨: .

SNBL_GY
삭제됨: ,

SNBL_GY
삭제됨: ing

38. Line 122: I assume you hint towards faster aging under higher temperatures, but this is nowhere clearly stated. Please add this information and make the relationship easier understandable for the readers. **R38.** Thank you for this question. Added in Discussion.

Results

39. since M&M comes at the end of the manuscript it would be helpful to get some additional information in the results already. E.g. at which stage you put the frames of which staged brood in the experimental conditions, you marked workers and those were tested later, **R39**. Thank you for your question. We apologize for the oversight. According to PLOS ONE guidelines, the Materials and Methods chapter should precede the Results. We have corrected this, and the logical flow is now restored.

40. Line 128 and all following statistical results: please add test statistics in addition to the p-value. Currently unclear which test you conducted and its effect size. **R40.** Thank you for this question. We have addressed this omission and added the name of the statistical method to the text of the manuscript where it was used. For your convenience, you can review the corrections in the screenshot below.

Dynamics of ambient temperature in summer and fall months

SNBL_GY 삭제됨:

The daily temperature in the first experimental room was set at 25°C for five months, and there were no significant differences compared to the natural daily average temperature in the summer months or September (ANOVA, Duncan post hoc tests, $p > 0.05$). The difference in experimental conditions for the honey bees from the Group control and the Group 25 was due to day-night temperature fluctuations, which were absent in the latter Group. Although significant temperature differences were not detected, a stable elevated night temperature is one of the factors of climate warming that influences honey bees. However, it was significantly greater than the average daily temperature in October 2022 (ANOVA, Duncan post-hoc test, $p < 0.05$). The daily temperature in the second

41. Line 132 and 312: Why do you start with Figure 6? This should be Figure 1. **R41.** Thank you for this question. This concerns the experimental design (Fig. 6E), which is detailed in the Materials and Methods section, following the Results section according to PLOS ONE guidelines. Due to the complexity of this numbering, we have removed this reference.

42. A) Line 142: 29.995 ± 0.982 and 33.398 => the difference for the brood between the groups was only 3C instead of the 10C of the chambers. This should be mentioned much earlier. It basically means that the bees heat up the brood area to ~30C. Does 33C mean that the bees cool down the nest in the 35C treatment or are discrepancies in the ways nest and room temperatures are measured? B) What were the according measures in the control group? **R42.** A) Thank you for this question. It is well-known that honey bees maintain a consistent brood microclimate (Abou-Shaara et al., 2017; Bencsik et al., 2023) through cooling or heating, so a small difference in brood temperature of 3°C was expected even though the difference in ambient temperature was 10°C. The normal temperature range within a honey bee colony is from 33 to 36°C (Kovac et al., 2009) during spring and summer, with a more precise range of 34.5 ± 1.5°C (Jones et al., 2005), as mentioned in the Discussion. Additionally, Szentgyörgyi et al. (2018) reported that workers and drones developing at 32°C live longer than those developing at 35°C in the spring generation of bees. We observed lower brood temperatures in August and September, which is suggested to be related to the longer lifespan of honey bees reared for overwintering. B**)** We added this important point to the manuscript. Please, check the screenshot for convenience.

Brood microclimate and colony development in August and September

In this study, we evaluated the performance of the colony using temperature, humidity, CO_2, number of larvae, capped brood area, and honey bees' measurements from August 08 to September 18, 2022, when short-lived bees commenced rearing larvae that would develop into long-lived bees for the overwintering period. Since the colony performance (Fig 1 D, E, F) of bees and the expected ambient temperature in August and early September showed no significant differences between the Groups control and the 25 on August 08, sensors were not set up in the Group control. We hypothesized that the regulation of the nest climate inside colonies influences the physiology of winter bees

SNBL_GY 삭제됨: hone
SNBL_GY 삭제됨: hone
SNBL_GY 서식 있음: [illegible]
SNBL_GY 서식 있음: [illegible]
SNBL_GY 서식 있음: [illegible]
SNBL_GY 서식 있음: [illegible]
SNBL_GY 서식 있음: [illegible]
SNBL_GY 서식 있음: [illegible]
SNBL_GY

43. Line 156++: "The molecular markers recognition related to the biological age of long-lived honey bees, which were approximately 4.5 months old, was verified through a dataset comprising": sorry but I don't understand what you want to say here... E.g. what do the molecular markers "recognize"? And what was verified? **R43.** Thank you for this question. We have concisely rephrased the subtitle of this paragraph and the first sentence. In summary, comparing well-known and not-yet-studied markers allows us to analyze their relationship with aging. The word "verify" did not express idea the of "analyze the relationship" and has been replaced. For your convenience, you can review the corrections in the screenshot below.

The analysis of the relationship between molecular markers and aging

The relation of the molecular markers to the biological age of overwintered honey bees was analysed through a dataset comprising two well-known physiological markers (Acinus size and Fat body weight) and six molecular markers (*vg*, *TOR1*, *JHAMT*, *ilp1*, *ilp2*, and *HSP70*) using Data Mining methods, including Discriminant Analysis (DA), Agglomerative Hierarchical Clustering (AHC), and Elastic Net Regression.

SNBL_GY 삭제됨: in overwintered western honey bees
SNBL_GY 삭제됨: recognition related
SNBL_GY 삭제됨: of long-lived honey bees, which were approximately 4.5 months old,
SNBL_GY 삭제됨: verified
SNBL_GY 삭제됨:

44. Line 168:"A comparison revealed significant differences between Group 25 and Group 35" Please be more specific. What kind of comparison, which statistics are you talking about here?"**R44.** Thank you for this question. This paragraph describes the results of the Discriminant Analysis using Wilks' Lambda, which measures how well the discriminant functions differentiate between the groups. You can see this data in Screenshot 1 of the Excel sheet. Additionally, we have added the name of the statistic before the p-value. For your convenience, you can review the corrections in Screenshot 2 below.

Screenshot 1

Discriminant Analysis:

Means by class:

Class \ Variable	HSP 70	ILP-1	ILP-2	TOR	Jhamt	Vg	Fat body	Acini size
25	1.214	1.648	1.633	0.716	0.964	0.807	0.833	1.000
35	1.684	3.082	3.109	1.551	3.292	0.103	0.208	0.994
Ctrl	1.000	1.000	1.000	1.000	1.000	1.000	1.000	1.000

Unidimensional test of equality of the means of the classes:

	Lambda	F	DF1	DF2	p-value
HSP 70	0.072	25.907	2	4	0.005
ILP-1			2	4	
ILP-2			2	4	
TOR			2	4	
Jhamt	0.071	26.044	2	4	0.005
Vg	0.003	611.655	2	4	<0.0001
Fat body			2	4	
Acini size	0.244	6.191	2	4	0.060

Wilks' Lambda test (Rao's approximation):

Lambda	0.000	
F (Observed value)	873.322	
F (Critical value)	19.371	
DF1	8	
DF2	2	
p-value (Two-tailed)	**0.001**	**
alpha	0.05	

*Signification codes: 0 < "***" < 0.001 < "**" < 0.01 < "*" < 0.05 < "." < 0.1 < " " < 1*

Test interpretation:
H0: The means vectors of the 3 classes are equal.
Ha: At least one of the means vector is different from another.
As the computed p-value is lower than the significance level alpha=0.05, one should reject the null hypothesis H0, and accept the alternative hypothesis Ha.

Screenshot 2

identify the optimal combination of features that effectively distinguishes them. The Wilcoxon Lambda test within the DA yielded a statistically significant result (Two-tailed test, $p < 0.05$), indicating that the model successfully differentiated between groups (**Fig 2A**). The observed Wilks' lambda value of 0.000 signifies a robust separation of the Groups control, 25, and 35, demonstrating a high level of discriminative power. A comparison revealed significant differences between Group 25 and Group 35 ($F(2,8) = 19.37$; Two-tailed test, $p < 0.05$; **Fig 2A**).

45. Line 169: How many individuals were tested per colony. Based on Fig 2B I assume that 3 colonies per treatment were tested? One individual per colony or multiple? Please also add this information somewhere in the results, to give an idea of sample size. It would also be more telling to plot all the data points that went in the PCA (Fig 2A), which would also give an impression on the variation between samples. **R45.** Thank you for this question. We modified the title of Figure 2 by adding an explanation about used variables and sample size (Screenshot 1 below). Also, we created Figure 7, which includes the sample size for each data set (Screenshot 2 below).

Screenshot 1

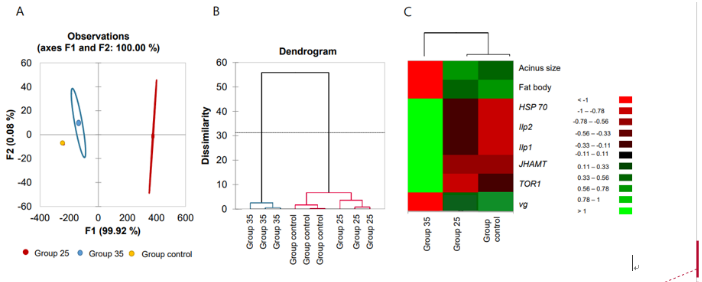

Fig 2. The discrimination of overwintered honey bees was performed using eight variables related to aging: two physiological variables (Acinus size, n = 30; Fat body weight, n = 10) and six molecul[illegible]ables (n = 10), including the expression levels of *HSP70, ilp1, ilp2, JHAMT, TOR1,* and *vg* genes.

(A) Discriminant Analysis. **(B)** Agglomerative Hierarchical Clustering (AHC). **(C)** Heatmap with cluster analysis based on ddCt values, Acinus size (mm²), and Fat body weight (mg). Honey bee colonies in Group 25 were maintained in room conditions at 25°C; colonies in Group 35 were kept in room conditions at 35°C, while Group Control colonies were maintained

SNBL_GY
삭제됨: long-lived

SNBL_GY
삭제됨: at the end of winter.

SNBL_GY
서식 있음: 글꼴: 기울임꼴

SNBL_GY
서식 있음: 글꼴: 기울임꼴

SNBL_GY
서식 있음: 글꼴: 기울임꼴

Screenshot 2

Apis mellifera ligustica **honey bees**

Month of sampling		Group control (3 colonies)	Group 25 (3 colonies)	Group 35 (3 colonies)
Experimental condition (summer, September, and October)			T_{room} = 25 °C	T_{room} = 35 °C
**August September**	Data set 1 (brood microclimate and colony development, marking of hatched honey bees)		Nest climate regulation	
	Statistics of data set 1		T-test (p < 0.05)	
**February**	Sampling of marked honey bees, n	(for physiology markers): 30+30+30 (for molecular markers): 10+10+10	30+30+30 10+10+10	30+30+30 10+10+10
	Data set 2	Two physiology markers (hypopharengial glands (HG) and fat body) Molecular marker genes related to nutrition (*vg, ilp1, ilp2, TOR1*), development (*JHAMT*), and stress response (*HSP70*).		
	Statistics of data set 2	1. Discriminant analysis (define the variables with high discrimination score); 2. Agglomerative Hierarchical Clustering (check the natural groupings within dataset); 3. Elastic Net Regression (model the relationship between HG and fat body with molecular markers); 4. ANOVA, Duncan post-hoc test (comparison of significance within each variable, p < 0.05).		

Fig 7. Experimental conditions, sampling schedule, data sets, and statistical tests used for analysis

46. Line 174+175: Please correct the interpretation of the DA results here: 99.98% is the percent variation explained along this axis. You could check based on the factor loadings which of the factors is the most telling here. How does this figure indicate that gene expression of ... contribute to this separation? Please clarify. **R46.** Thank you for this question. We sorry and fixed this inaccuracy in the text of manuscript and changed 99.98% to 99.92% as pointed in Figure 2A (Screenshot 1). When a higher Fisher's distance indicates better separation between the groups, the discriminant function coefficients contribute the value of each variable to this separation in XLSTAT soft**.** You can see these coefficients in the table in the Screenshot 2 below. Discriminant function coefficient in DA has the same meaning as factor loadings in Factor Analysis.

Screenshot 1

(FD = 783.659; $p < 0.05$). The honey bees were separated along the primary discriminant axis F1 in 99.92% accuracy, where discriminant function coefficients for gene expressions *vg*, *HSP70*, and *JHAMT* were -20.092, -14.960, and 23.576, respectively. These coefficients were the highest, providing significant contributoins to this separation.

Screenshot 2

Standardized canonical discriminant function coefficients:

	F1	F2
HSP 70	-14.960	7.939
ILP-1	0.000	0.000
ILP-2	0.000	0.000
TOR	0.000	0.000
Jhamt	23.576	-7.889
Vg	-20.092	-0.120
Fat body	0.000	0.000
Acini size	15.134	1.315

47. Fig3: maybe move panel A to supplement. Panel B, add "fatbody size" to header. Currently it is not clear what exactly you are testing. I.e. instead of "dependent variable" specify that you are testing which of the gene expression levels correlates with fatbody and acinus size. Also add that you are testing. **R47**. Thank you for this question. We accepted the correction and removed Figure 3A, renaming Figure 3B to 3A and 3C to 3B. Additionally, we corrected

"Fat body" to "Fat body weight." We also rewrote the footnotes of Figure 3 to include more details. For your convenience, you can review the corrections in the screenshot below.

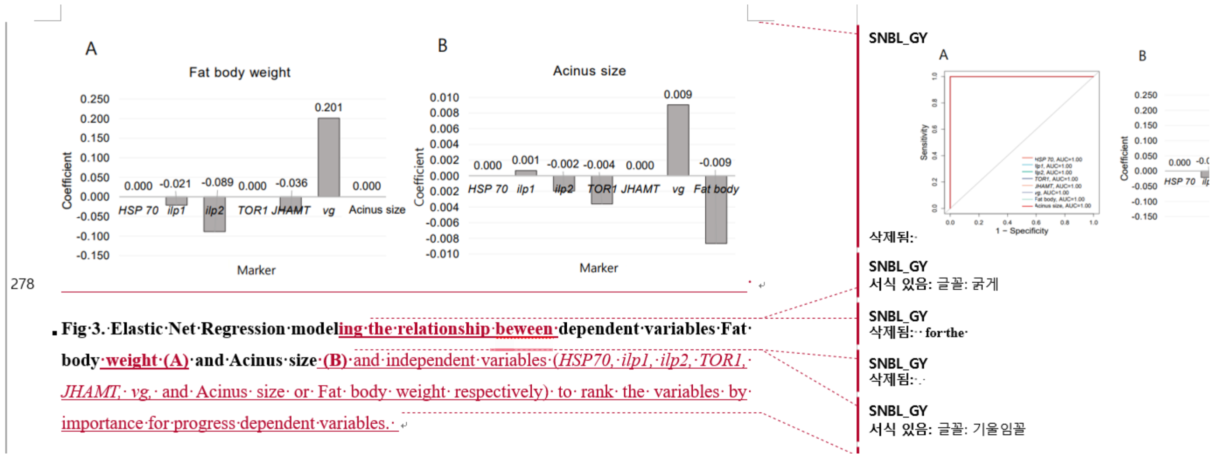

48. I am actually wondering why you are conducting this analysis at all? If I understand correctly you are testing which of the variables, i.e. the expression of the different genes affects fatbody or acinus size, and whether fatbody size affects acinus size and vise versa. A) do you really expect that there is a causal relationship? B) I thought that the main aim was to test whether there are gene expression differences of these candidate genes between the different treatment groups, i.e. the results given in Fig 5. **R48.** Thank you for this question. Elastic Net regression, Discriminant Analysis, Agglomerative Hierarchical Clustering, and Correlation Analysis are part of Data mining. They are used to analyze large datasets to uncover patterns, relationships, and insights that cannot be found using only comparative analysis of variables one by one.

The strategy for choosing these methods is described in the Materials and Methods chapter. Briefly: (a) **Discriminant Analysis** (DA) classifies the marker dataset to find a combination of features that best separates groups. (b) To discover natural groupings in the absence of labeled information, **Agglomerative Hierarchical Clustering** (AHC) was used. (c) **Elastic Net Regression** models the relationship between the target variable and all other variables to rank them by importance for the target. (d) Moreover, **Correlation Analysis** is a fundamental tool in Data mining used to identify relationships between variables.

Addressing specific points: A) **Causal Relationships:** While these methods do not establish causality, they help identify potential relationships and patterns within the data. The goal is not

necessarily to demonstrate a causal link but to explore the potential connections and variables that warrant further investigation. B) **Main Aim:** The primary aim is indeed to test gene expression differences between treatment groups, as illustrated in Figure 5. The additional analyses are intended to explore the relationships between gene expression, fat body size, and acinus size in greater depth. These analyses help to understand whether and how these variables might interact, which can provide a more comprehensive view of the data and guide future research directions.

In summary, while these methods may predict possible relationships, they are tools to broaden our understanding and to build hypotheses for future experiments on aging.

49. Fig 4: again it would be better to depict the data as boxplot with single data points visible. Line 207: change to "physiological aging" **R49.** Thank you for this question. We corrected the mistyping in line 207. We aim to use a consistent plot style throughout the manuscript whenever possible, and we prefer to keep the same plot. The sample size, which you suggested be indicated with individual data points on another plot, is noted in the footnotes of the plots in Figure 4.

50. Line 209: I understand what you mean with "physiological age" above, and it makes sense. Here I am lost what you mean by "biological age"? Is this also supposed to mean "physiological age", because based on M&M all the tested workers should be 4.5 months old and age matched, right? **R50.** Thank you for this question. The terms "physiological age" and "biological age" are used interchangeably, as both express the of metabolic processes which speed and quality can be different at the person with same chronological age.

51. Fig5: which measure for "relative expression level" did you use, deltaCT? Add information to Y-axes. Add measures to the Y-axes. Again depict results as boxplots with data points. **R51.** Thank you for this missing clarification. Yes, the above-mentioned "relative expression" originates from the Ct data, which is used to calculate the fold differences between a target gene and a reference gene (delta Ct), and later, between a certain condition and control ($2^{-\Delta\Delta CT}$) (Livak and Schmittgen, 2001) (Screenshot 1). In the original article, the Y-axis is labeled as "Fold change in gene expression" (please see Fig. 3 in Screenshot 2), and we suggest using this instead of "Relative expression". For your convenience, you can review the corrections in the Screenshot 3 below.

Screenshot 1

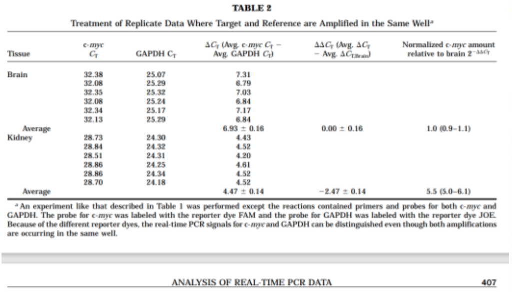

TABLE 2

Treatment of Replicate Data Where Target and Reference are Amplified in the Same Well[a]

Tissue	c-*myc* C_T	GAPDH C_T	ΔC_T (Avg. c-*myc* C_T − Avg. GAPDH C_T)	$\Delta\Delta C_T$ (Avg. ΔC_T − Avg. $\Delta C_{T,Brain}$)	Normalized c-*myc* amount relative to brain $2^{-\Delta\Delta C_T}$
Brain	32.38	25.07	7.31		
	32.08	25.29	6.79		
	32.35	25.32	7.03		
	32.08	25.24	6.84		
	32.34	25.17	7.17		
	32.13	25.29	6.84		
Average			6.93 ± 0.16	0.00 ± 0.16	1.0 (0.9–1.1)
Kidney	28.73	24.30	4.43		
	28.84	24.32	4.52		
	28.51	24.31	4.20		
	28.86	24.25	4.61		
	28.86	24.34	4.52		
	28.70	24.18	4.52		
Average			4.47 ± 0.14	−2.47 ± 0.14	5.5 (5.0–6.1)

[a] An experiment like that described in Table 1 was performed except the reactions contained primers and probes for both c-*myc* and GAPDH. The probe for c-*myc* was labeled with the reporter dye FAM and the probe for GAPDH was labeled with the reporter dye JOE. Because of the different reporter dyes, the real-time PCR signals for c-*myc* and GAPDH can be distinguished even though both amplifications are occurring in the same well.

ANALYSIS OF REAL-TIME PCR DATA 407

Screenshot 2

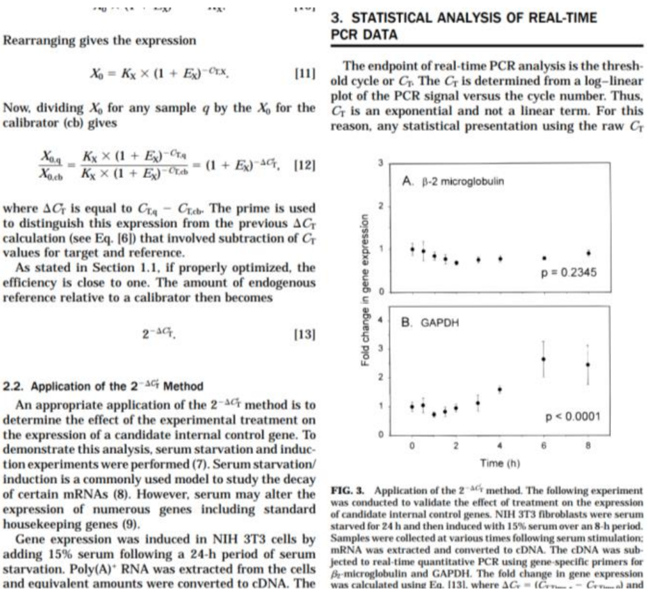

Rearranging gives the expression

$$X_0 = K_X \times (1 + E_X)^{-C_{T,X}}. \quad [11]$$

Now, dividing X_0 for any sample q by the X_0 for the calibrator (cb) gives

$$\frac{X_{0,q}}{X_{0,cb}} = \frac{K_X \times (1 + E_X)^{-C_{T,q}}}{K_X \times (1 + E_X)^{-C_{T,cb}}} = (1 + E_X)^{-\Delta C'_T}, \quad [12]$$

where $\Delta C'_T$ is equal to $C_{T,q} - C_{T,cb}$. The prime is used to distinguish this expression from the previous ΔC_T calculation (see Eq. [6]) that involved subtraction of C_T values for target and reference.

As stated in Section 1.1, if properly optimized, the efficiency is close to one. The amount of endogenous reference relative to a calibrator then becomes

$$2^{-\Delta C'_T}. \quad [13]$$

2.2. Application of the $2^{-\Delta C'_T}$ Method

An appropriate application of the $2^{-\Delta C'_T}$ method is to determine the effect of the experimental treatment on the expression of a candidate internal control gene. To demonstrate this analysis, serum starvation and induction experiments were performed (7). Serum starvation/induction is a commonly used model to study the decay of certain mRNAs (8). However, serum may alter the expression of numerous genes including standard housekeeping genes (9).

Gene expression was induced in NIH 3T3 cells by adding 15% serum following a 24-h period of serum starvation. Poly(A)+ RNA was extracted from the cells and equivalent amounts were converted to cDNA. The

3. STATISTICAL ANALYSIS OF REAL-TIME PCR DATA

The endpoint of real-time PCR analysis is the threshold cycle or C_T. The C_T is determined from a log–linear plot of the PCR signal versus the cycle number. Thus, C_T is an exponential and not a linear term. For this reason, any statistical presentation using the raw C_T

FIG. 3. Application of the $2^{-\Delta C'_T}$ method. The following experiment was conducted to validate the effect of treatment on the expression of candidate internal control genes. NIH 3T3 fibroblasts were serum starved for 24 h and then induced with 15% serum over an 8-h period. Samples were collected at various times following serum stimulation; mRNA was extracted and converted to cDNA. The cDNA was subjected to real-time quantitative PCR using gene-specific primers for β_2-microglobulin and GAPDH. The fold change in gene expression was calculated using Eq. [13], where [illegible] and

Screenshot 3

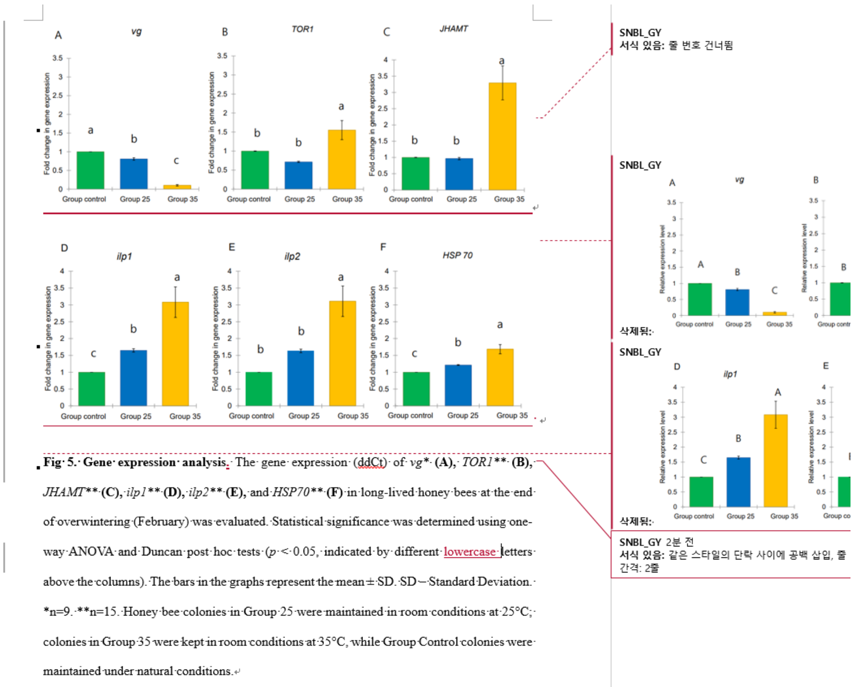

Fig 5. Gene expression analysis. The gene expression (ddCt) of *vg** **(A)**, *TOR1*** **(B)**, *JHAMT*** **(C)**, *ilp1*** **(D)**, *ilp2*** **(E)**, and *HSP70*** **(F)** in long-lived honey bees at the end of overwintering (February) was evaluated. Statistical significance was determined using one-way ANOVA and Duncan post hoc tests ($p < 0.05$, indicated by different lowercase letters above the columns). The bars in the graphs represent the mean ± SD. SD—Standard Deviation. *n=9. **n=15. Honey bee colonies in Group 25 were maintained in room conditions at 25°C; colonies in Group 35 were kept in room conditions at 35°C, while Group Control colonies were maintained under natural conditions.

52. General comment in respect to results. P-values are mostly > or <0.05. "<0.05 means between 0.01-0.05, is this always the case, are p-values always in this range? **R52.** Thank you for this question. Our reporting follows the standard scientific practice of providing more precise p-values when necessary to convey the statistical significance more accurately. Therefore, while some p-values might be reported as "<0.05", others are reported with more precision if they fall below commonly used thresholds (e.g., 0.01, 0.001). In summary, "<0.05"

means the *p*-value is less than 0.05, but not necessarily restricted to the range of 0.01 to 0.05. We ensure to report *p*-values with appropriate precision to reflect the significance of the results accurately.

Discussion

53. Line 234: "during the previous summer" does this mean "during larval development"? Maybe specify this information for better understanding. **R53.** Thank you for this question. Honey bee colony development in spring, summer, and fall includes stages of open brood (eggs and larvae) and capped brood (pre-pupae and pupae), which are cared for by younger nurse-stage adults. Food carrying is provided by older adults in the forager stage, with the amount and numbers adjusted according to weather and colony conditions. When I mention "during the previous summer", I am referring to the influence of ambient temperature on the entire colony, including the larval stage, and how this necessitates a colony response to survive under experimental conditions and prepare for overwintering in the late summer and fall. Since 'during the previous summer' encompasses the meaning of 'during larval development,' we prefer to use the former expression.

54. Line 243: you need to give the actual mean temperatures here as well, not just the difference. Otherwise readers might not know and/or remember that they had 30 C. **R54.** Thank you for this question. We fixed it. For your convenience, you can review the corrections in the screenshot below.

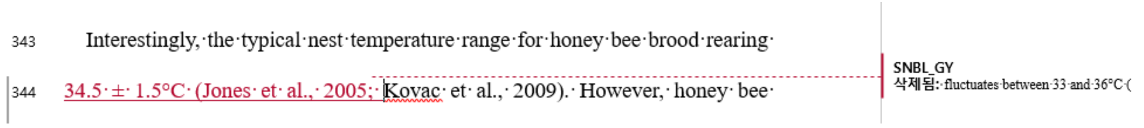
343 Interestingly, the typical nest temperature range for honey bee brood rearing
344 34.5 ± 1.5°C (Jones et al., 2005; Kovac et al., 2009). However, honey bee
SNBL_GY
삭제됨: fluctuates between 33 and 36°C (

55. Line 248: what do you mean by "initially had to decrease the nest temperature"? It sounds like both groups had to...? But the 25 C group actually needs to increase temperature to reach 30C. **R55.** Thank you for this question. We need to address some misunderstandings. We believed that "in nature" more clearly emphasized the natural condition as opposed to "initially" (i.e., without experiments) and made this change. We mean that the need to "decrease the nest temperature" is not yet a well-known adaptation for rearing long-lived honey bees, especially compared to the well-documented nest microclimate temperature in spring/ summer found in

other research and in late summer/ early fall in our experiment with Group 35. It requires additional field experiments to deepen our understanding of the transformation related to longevity of honey bees.

56. Please clarify that Vg and JH expression are connected. Which way and in which bees/castes should become clearer and to what extent does it fit to your results...**R56.** Thank you for this question. Added to Introduction and Discussion.

57. Line 276: "relationship between vg gene expression and the hypopharyngeal gland (HG) and Fat body" I neither see anything about the hypopharyngeal gland in Fig3, nor anywhere in the results, nor any relationship of this gland and Vg and the fatbody. **R57.** Thank you for this question. We added this clarification. You can review the corrections in the screenshot below for your convenience.

35, which also suggests an aging process in the latter honey bees.

It is known that Vg is synthesized in the fat body (Amdam et al., 2012), from where it transfers to the HG and is used as an amino acid donor for the synthesis of royal jelly in nurse honey bees (Ramanathan et al., 2018). This relationship between *vg* gene expression and the hypopharyngeal gland (HG) and Fat body was validated by the high coefficient values using Elastic Net Regression models **(Fig 3B, C).** Furthermore, Discriminant Analysis (DA) identified three

SNBL_GY
삭제됨: e

58. Line 282: " it is associated with honey bee transitions in summer and aging" not sure what you mean here? Please be more specific and clarify. **R58.** Thank you for this question. We rewrite this sentence and related following discussion. For your convenience, you can review the corrections in the screenshot below.

significance in discriminating among honey bee groups. Even though the *HSP70* gene is synthesized in response to stress (Zhang et al., 2015), Sarıoğlu-Bozkurt et al. (2022) reported that its levels also increase in overwintered honey bees from fall to early spring and respond to different supplemental diets along with vg, demonstrating the complex regulation of this gene. We found that the highest *HSP70* gene expression was observed in the overwintered honey bees from Group 35, while the lowest was found in the Group control. Based on this, we suggest that honey bees from Group 35 experienced a stress response, possibly related to their different nutritional needs, which may later stimulate the ending of diapause and the initiation of earlier colony development than honey bees from Groups control and 25. This supports the aging hypothesis temperature-treated honey bees.

SNBL_GY
삭제됨: it is associated with honey bee transitions i and aging (Sario˘glu-Bozkurt et al., 2022). T

SNBL_GY
삭제됨: was observed

SNBL_GY
삭제됨:

SNBL_GY
삭제됨: while

SNBL_GY
삭제됨: was

SNBL_GY
삭제됨: group,

SNBL_GY
삭제됨: ing

SNBL_GY
삭제됨: of

59. Line 286: "The subsequent gene, TOR1, wasn't selected by Data Mining methods but can be considered aging-related (Barth et al., 2010; Fahrbach et al., 2012). " This statement needs to be reformulated. You do not find an association of TOR1 expression with physiological aging in your study. There are other studies which do. What could be the difference why they find it you don't? **R59.** Thank you for this question. We rewrite this discussion part. For your convenience, you can review the corrections in the screenshot below.

development compared to honey bees from Groups control and 25. Also, the increased nutritional needs of honey bees from Group 35 are explained by the

21

significant increase in *TOR1* gene expression, because this evolutionarily conserved gene integrates signals from nutrients (amino acids and energy) and growth factors to regulate cell growth (Fingar, Blenis, 2004; Barth et al., 2010; Fahrbach et al., 2012). Additionally, significantly higher *HSP70* and *TOR1* gene expressions in honey bees from Group 35, compared to Group Control and Group 25, were associated with a decrease in fat body weight, although this decrease was not yet statistically significant. This supports the aging hypothesis for temperature-treated honey bees. However, *TOR1* gene was not selected by Data Mining methods as marker possibly because it had less fluctuation between Groups of honey bees compared to other genes.

SNBL_GY
서식 있음: 글꼴: 기울임꼴

SNBL_GY
[2] 이동함(삽입)

SNBL_GY
서식 있음: 글꼴: 기울임꼴

SNBL_GY
서식 있음: 글꼴: 기울임꼴

SNBL_GY 2시간 전
삭제됨: ing

SNBL_GY
삭제됨: of

SNBL_GY
삭제됨: The subsequent gene,

SNBL_GY
삭제됨: .

SNBL_GY
서식 있음: 글꼴: 기울임꼴 없음

SNBL_GY
삭제됨: n't

60. Line 288: "explaining the decrease in Fat body " this is not correct. You find a correlation of both not a causal relationship, please reformulate. **R60.** Thank you for this question. We rewrite this discussion part. For your convenience, you can review the corrections in the screenshot below.

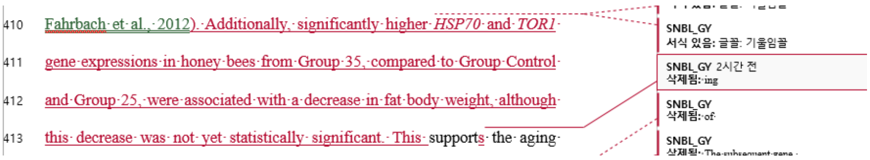
Fahrbach et al., 2012). Additionally, significantly higher *HSP70* and *TOR1* gene expressions in honey bees from Group 35, compared to Group Control and Group 25, were associated with a decrease in fat body weight, although this decrease was not yet statistically significant. This supports the aging

61. Line 293: this summary sentence is rather confusing. **R61.** Thank you for this point. We rewrote this summary. For your convenience, you can review this in the screenshot below.

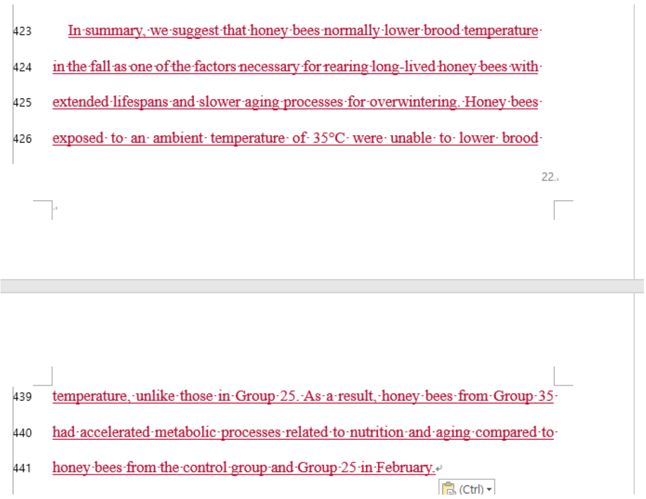
In summary, we suggest that honey bees normally lower brood temperature in the fall as one of the factors necessary for rearing long-lived honey bees with extended lifespans and slower aging processes for overwintering. Honey bees exposed to an ambient temperature of 35°C were unable to lower brood

22

temperature, unlike those in Group 25. As a result, honey bees from Group 35 had accelerated metabolic processes related to nutrition and aging compared to honey bees from the control group and Group 25 in February.

62 Line 295: why "conversely"? **R62.** Thank you for this point. We rewrote this and the "conversely" word was deleted. For your convenience, you can review this in the screenshot above in R61.

63. Did you continue observing those colonies? Did the differences in the 35C group lead to lower survival of the colonies, smaller colony sizes,... any fitness differences? **R63.** Thank you for this question. After detecting the overwintering success in mid-February, these colonies were given nutritional stimulation and involved in other experiments, making continued observation of the colonies impossible.

64. Figure 6D: change "no regulated" to "not regulated" **R64.** Thank you for this question. We corrected. Please, check Figure 6D.

65. Line 357: change "whether differed" to "whether it differed" **R65.** Thank you for this

question. We corrected.

66. Line 372: "name, company, country" add according information
Are the results of the lipid content given in the results, or somewhere in the manuscript? => either add results or remove information from M&M **R66.** Thank you for your question. We apologize for the typographical error. We have added “name, company, country”. In this research, a method was used to analyze lipids in the fat body, as it is the most prominent component. This clarification has been added to the abstract and elsewhere in the manuscript. For your convenience, you can review this in the screenshots below.

Abstract:

Colony performance was assessed in August and September. In February, workers were examined for physiological traits (Acinus size and weight of lipid content in the Fat body) and expression of known task-related molecular markers (*vg* and

Introduction:

Republic of Korea. The aging of honey bees at the end of diapause (broodless period) in February was determined by eight markers, including two physiological markers (Acinus size and weight of lipid content in the Fat body) and six molecular gene markers related to the task (*vg* and *JHAMT*), nutrition (*ilp1, ilp2*, and *TOR1*), and stress response (*HSP70*). Our findings suggest that

Discussion:

main fat body component, and >90 % of the lipids are in the form of triglycerides, which are synthesized from dietary carbohydrates, fatty acids and used for the synthesis of pheromones, phospholipids, and wax (Arrese and Soulages, 2010). In our research, Acinus sizes in honey bee Groups 25 and the control were significantly greater ($p < 0.05$) than those in honey bee Group 35. The same trend was observed in weight of lipid content in the Fat body, although the differences were not

SNBL_GY
삭제됨: A comparative study revealed that

SNBL_GY
삭제됨: weights

Also, corrections were made in the Results chapter as well.

67. Line 377: add information on how and when the tissue(s) were dissected. Before freezing at -80C? Were they dissected and then directly isolated? Were samples dissected on ice? **R67.** Thank you for bringing up this point. We have clarified this step of the experiment. For your convenience, a screenshot is provided below for your review.

colonies were sampled in labeled tubes and stored at -80°C. Three honey bees from each colony were analyzed. The brain and gut-free abdomen of honey bees were dissected on ice, and RNA was extracted directly. RNA was extracted from

SNBL_GY 서식 있음:

SNBL_GY 서식 있음:

68. Line 435: change "group" to "groups"

Please indicate which tests were conducted using excel. **R68.** Thank you for bringing up this point. We have fixed the missing letter (Screenshot 1). This paragraph describes the results of the Discriminant Analysis (DA), which was fully spelled out in the paragraph above Figure 7 (Screenshot 2). For your convenience, a screenshots are provided below for your review.

Screenshot 1

with the highest discrimination scores were selected using DA because it determines the linear combination of variables and provides maximal separation between groups. To discriminate the groups, Fisher distances were applied, which emphasize the linear combinations of variables that maximize the differences

Screenshot 2

of features that best separates different groups (supervised approach, Discriminant Analysis (DA)); (b) clustering to discover natural groupings in the absence of labeled information (unsupervised approach, Agglomerative

69. Line 460: there should be additional supplementary tables with the raw data of all measures/data reported on. **R69**. Corrected.

PONE-D-24-13353
PLOS ONE

We included when submitting your revised manuscript:
• A rebuttal letter that responds to each point raised by the academic editor and reviewer(s). You should upload this letter as a separate file labeled **'Response to Reviewers'**.
• A marked-up copy of your manuscript that highlights changes made to the original version. You should upload this as a separate file labeled **'Revised Manuscript with Track Changes'**.
• An unmarked version of your revised paper without tracked changes. You should upload this as a separate file labeled '**Manuscript**'.

If you would like to make changes to your financial disclosure, please include your updated statement in your cover letter. Guidelines for resubmitting your figure files are available below the reviewer comments at the end of this letter.

When submitting your revision, we need you to address these additional requirements.
1. Please ensure that your manuscript meets PLOS ONE's style requirements, including those **for file naming**. The PLOS ONE style templates can be found at https://journals.plos.org/plosone/s/file?id=wjVg/PLOSOne_formatting_sample_main_body.pdf and https://journals.plos.org/plosone/s/file?id=ba62/PLOSOne_formatting_sample_title_authors_affiliations.pdf **1R: Corrected**
2. We note that **the grant information** you provided in the 'Funding Information' and 'Financial Disclosure' sections do **not match**.
When you resubmit, please ensure that you provide the correct grant numbers for the awards you received for your study in the 'Funding Information' section. **2R: during resubmission will do**
3. Thank you for **stating the following financial disclosure**:
"Yes
Hyung Wook Kwon received.
This work was carried out with the support of the Cooperative Research Program for Agriculture Science & Technology Development (Project No. RS-2023-00232749) and the Priority Research Centers Program through the National Research Foundation of Korea (NRF) funded by the Ministry of Education (2020R1A6A1A03041954)."
Please state what role the funders took in the study. If the funders had no role, please state: "The funders had no role in study design, data collection and analysis, decision to publish, or preparation of the manuscript."
If this statement is not correct you must amend it as needed.
Please include this amended Role of Funder statement in your cover letter; we will change the online submission form on your behalf. **3R: Sorry, "no" is correct. We added the sentence in Manuscript "The funders had no role in study design, data collection and analysis, decision to publish, or preparation of the manuscript."**

We are pleased to resubmit for publication the manuscript "The effect of seasonal temperatures on the aging of the overwintered honey bee". We would like to thank the reviewers for their careful and thorough reading of this manuscript and for the thoughtful comments and

constructive suggestions, which helped to improve the quality of this manuscript.

We look forward to hearing from you.

Sincerely,

Professor Hyung Wook Kwon

Department of Life Science,

Convergence Research Center for Insect Vectors

Incheon National University

119 Academy-ro, Yeonsu-gu, Incheon, 22012, Republic of Korea

+82-32-835-8090 (work);

+82-10-3379-6727 (mobile);

+82-32-835-0764 (fax).

Email: hwkwon@inu.ac.kr
