# Supplementary material for: The effect of seasonal temperatures on the physiology of the overwintered honey bee
Source: PLoS One. 2024 Dec 9;19(12):e0315062. doi: 10.1371/journal.pone.0315062 (PMC11627422; doi:10.1371/journal.pone.0315062)
Supplement: Attachment — Submitted filename: PONE-D-24-13353_R2-1 Nov.pdf [file pone.0315062.s016.pdf]

PLOS ONE

The effect of seasonal temperatures on the physiology of the overwintered honey bee

--Manuscript Draft--

**Manuscript Number:**	PONE-D-24-13353R2
**Article Type:**	Research Article
**Full Title:**	The effect of seasonal temperatures on the physiology of the overwintered honey bee
**Short Title:**	Physiology of honey bees at the end of winter
**Corresponding Author:**	Hyung Wook Kwon<br>Incheon National University<br>Incheon, KOREA, REPUBLIC OF
**Keywords:**	honey bee; Aging; vitellogenin; acinus size; molecular markers; climate warming
**Abstract:**	Honey bee physiology follows an annual cycle, with winter bees living ten times longer than summer bees. Their transition can be disrupted by climate change. Several climate factors, mainly temperature, may contribute to the global losses of winter bees. We simulated global warming by maintaining constant temperatures of 25°C (Group 25) and 35°C (Group 35) in rooms around hives from June to October, while a Group control experienced natural conditions. Colony performance was assessed in August and September. In February, workers were examined for physiological traits (acinus size and lipid content in the fat body) and molecular markers (vg and JHAMT), along with potential markers (ilp1,ilp2, TOR1, and HSP70). Our findings suggest that temperature decreases around winter worker broods from Group 25 in the fall led to their different physiological states related to aging in winter compared to Group 35 workers. Changes in bees from Group 35 the end of diapause were detected with an upregulation of HSP70, ilp2, and TOR1 genes. These signs of winter bees in response to summer global warming could lead to the development of strategies to prevent bee losses and improve the identification of physiological states in insect models.
**Order of Authors:**	Hyung-Wook Kwon
	Olga Frunze
	Yumi Yun
	Hyunjee Kim
	Ravil R. Garafutdinov
	Young-Eun Na
**Response to Reviewers:**	Dear Editor-in-Chief, Reviewer 1 and Reviewer 2!<br><br>Thank you for the time and effort spent reviewing our manuscript number PONE-D-24-13353R1 (The effect of seasonal temperatures on the physiology of the overwintered honey bee) and suggesting some important points to consider. We corrected the manuscript according to the reviewers' comments. Our replies are marked by blue after the symbol R (reply) and question numbers for the reviewer's comments. Also, we copied these modifications in response for convenience when they were available. Please, find below in attached file "Response to Reviewers" the reviewer comments (black) and our response (blue).<br><br>We would like to thank the reviewers for their careful and thorough reading of this manuscript and for the thoughtful comments and constructive suggestions, which helped to improve the quality of this manuscript.<br>We look forward to hearing from you.<br><br>Sincerely,<br>Professor Hyung Wook Kwon<br>Department of Life Science,<br>Convergence Research Center for Insect Vectors<br>Incheon National University, R&D Complex,<br>265 Harmony-ro, Yeonsu-gu, Incheon, 22014, Republic of Korea;

Powered by Editorial Manager® and ProduXion Manager® from Aries Systems Corporation

+82-32-835-8090 (work);
+82-10-3379-6727 (mobile);
+82-32-835-0764 (fax).
Email: hwkwon@inu.ac.kr

Additional Information:	
**Question**	**Response**
**Financial Disclosure**<br><br>Enter a financial disclosure statement that describes the sources of funding for the work included in this submission. Review the submission guidelines for detailed requirements. View published research articles from *PLOS ONE* for specific examples.<br><br>This statement is required for submission and **will appear in the published article** if the submission is accepted. Please make sure it is accurate.<br><br>**Unfunded studies**<br>Enter: *The author(s) received no specific funding for this work.*<br><br>**Funded studies**<br>Enter a statement with the following details:<br>• Initials of the authors who received each award<br>• Grant numbers awarded to each author<br>• The full name of each funder<br>• URL of each funder website<br>• Did the sponsors or funders play any role in the study design, data collection and analysis, decision to publish, or preparation of the manuscript?<br>• **NO** - Include this sentence at the end of your statement: *The funders had no role in study design, data collection and analysis, decision to publish, or preparation of the manuscript.*<br>• **YES** - Specify the role(s) played.<br><br>* typeset	Yes<br>Hyung Wook Kwon received.<br>This work was carried out with the support of the Cooperative Research Program for Agriculture Science & Technology Development (Project No. RS-2023-00232749) and the Priority Research Centers Program through the National Research Foundation of Korea (NRF) funded by the Ministry of Education (2020R1A6A1A03041954).<br><br>The funders had no role in study design, data collection and analysis, decision to publish, or preparation of the manuscript.
**Competing Interests**<br><br>Use the instructions below to enter a competing interest statement for this submission. On behalf of all authors, disclose any competing interests that could be perceived to bias this work—acknowledging all financial support	The authors have declared that no competing interests exist.

and any other relevant financial or non-financial competing interests.<br><br>This statement is required for submission and **will appear in the published article** if the submission is accepted. Please make sure it is accurate and that any funding sources listed in your Funding Information later in the submission form are also declared in your Financial Disclosure statement.<br><br>View published research articles from *PLOS ONE* for specific examples.<br><br>**NO authors have competing interests**<br><br>Enter: *The authors have declared that no competing interests exist.*<br><br>**Authors with competing interests**<br><br>Enter competing interest details beginning with this statement:<br><br>*I have read the journal's policy and the authors of this manuscript have the following competing interests: [insert competing interests here]*<br><br>* typeset	
**Ethics Statement**<br><br>Enter an ethics statement for this submission. This statement is required if the study involved:<br><br>• Human participants<br>• Human specimens or tissue<br>• Vertebrate animals or cephalopods<br>• Vertebrate embryos or tissues<br>• Field research<br><br>Write "N/A" if the submission does not require an ethics statement.<br><br>General guidance is provided below.	N/A

Powered by Editorial Manager® and ProduXion Manager® from Aries Systems Corporation

Consult the submission guidelines for detailed instructions. **Make sure that all information entered here is included in the Methods section of the manuscript.**<br><br>**Format for specific study types**<br><br>**Human Subject Research (involving human participants and/or tissue)**<br>• Give the name of the institutional review board or ethics committee that approved the study<br>• Include the approval number and/or a statement indicating approval of this research<br>• Indicate the form of consent obtained (written/oral) or the reason that consent was not obtained (e.g. the data were analyzed anonymously)<br><br>**Animal Research (involving vertebrate animals, embryos or tissues)**<br>• Provide the name of the Institutional Animal Care and Use Committee (IACUC) or other relevant ethics board that reviewed the study protocol, and indicate whether they approved this research or granted a formal waiver of ethical approval<br>• Include an approval number if one was obtained<br>• If the study involved *non-human primates*, add *additional details* about animal welfare and steps taken to ameliorate suffering<br>• If anesthesia, euthanasia, or any kind of animal sacrifice is part of the study, include briefly which substances and/or methods were applied<br><br>**Field Research**<br><br>Include the following details if this study involves the collection of plant, animal, or other materials from a natural setting:<br>• Field permit number<br>• Name of the institution or relevant body that granted permission	
**Data Availability**<br><br>Authors are required to make all data underlying the findings described fully available, without restriction, and from the	Yes - all data are fully available without restriction

time of publication. PLOS allows rare exceptions to address legal and ethical concerns. See the PLOS Data Policy and FAQ for detailed information.<br><br>A Data Availability Statement describing where the data can be found is required at submission. Your answers to this question constitute the Data Availability Statement and **will be published in the article**, if accepted.<br><br>**Important:** Stating 'data available on request from the author' is not sufficient. If your data are only available upon request, select 'No' for the first question and explain your exceptional situation in the text box.<br><br>Do the authors confirm that all data underlying the findings described in their manuscript are fully available without restriction?	
**Describe where the data may be found in full sentences. If you are copying our sample text, replace any instances of XXX with the appropriate details.**<br><br>• If the data are **held or will be held in a public repository**, include URLs, accession numbers or DOIs. If this information will only be available after acceptance, indicate this by ticking the box below. For example: *All XXX files are available from the XXX database (accession number(s) XXX, XXX.*).<br>• If the data are all contained **within the manuscript and/or Supporting Information files**, enter the following: *All relevant data are within the manuscript and its Supporting Information files.*<br>• If neither of these applies but you are able to provide **details of access elsewhere**, with or without limitations, please do so. For example:<br><br>*Data cannot be shared publicly because of [XXX]. Data are available from the XXX Institutional Data Access / Ethics Committee (contact via XXX) for*	All relevant data are within the manuscript and its Supporting Information files.

researchers who meet the criteria for access to confidential data.<br><br>*The data underlying the results presented in the study are available from (include the name of the third party and contact information or URL).*<br>• This text is appropriate if the data are owned by a third party and authors do not have permission to share the data.<br><br>* typeset	
Additional data availability information:	

Manuscript Click here to access/download;Manuscript;Manuscript2.docx

Research article

The effect of seasonal temperatures on the physiology of the overwintered honey bee

Olga Frunze[1¶], Yumi Yun[1¶], Hyunjee Kim[1], Ravil R. Garafutdinov[2], Young-Eun Na[3]*, and Hyung-Wook Kwon[1]*

[1]Department of Life Sciences & Convergence Research Center for Insect Vectors (CRCIV), Incheon National University R&D Complex, 265 Harmony-ro, Yeonsu-gu, Incheon, Republic of Korea 22014;

[2]Institute of Biochemistry and Genetics, Ufa Federal Research Center, Russian Academy of Sciences, 450054, prosp. Oktyabrya, 71, Ufa, Bashkortostan, Russian Federation

[3]Agro-materials Industry Division, Rural Development Administration (RDA), Wanju 55365, Republic of Korea

[¶]These authors contributed equally to this work

*Correspondence: hwkwon@inu.ac.kr (H.-W.K.) youngman99@korea.kr (Y.-E.N.)

Short title: **Physiology of honey bees at the end of winter**

Abstract

Honey bee physiology follows an annual cycle, with winter bees living ten times longer than summer bees. Their transition can be disrupted by climate change. Several climate factors, mainly temperature, may contribute to the global losses of winter bees. We simulated global warming by maintaining constant temperatures of 25°C (Group 25) and 35°C (Group 35) in rooms around hives from June to October, while a Group control experienced natural conditions. Colony performance was assessed in August and September. In February, workers were examined for physiological traits (acinus size and lipid content in the fat body) and molecular markers (*vg* and *JHAMT*), along with potential markers (*ilp1, ilp2, TOR1*, and *HSP70*). Our findings suggest that temperature decreases around winter worker broods from Group 25 in the fall led to their different physiological states related to aging in winter compared to Group 35 workers. Changes in bees from Group 35 the end of diapause were detected with an upregulation of *HSP70, ilp2,* and *TOR1* genes. These signs of winter bees in response to summer global warming could lead to the development of strategies to prevent bee losses and improve the identification of physiological states in insect models.

Introduction

Honey bees (below bees) play a crucial role in human life as pollinators on agricultural farms, producers of honey, royal jelly, and other products, which are valued for their health and medicinal benefits, and contributors to ecosystems worldwide. However, the loss of bees has been reported over the last two decades, particularly at the end of the overwintering period [1-3].

Several factors arising during the year can contribute to this [4]. For example, climate warming or cooling [5] impacts the lifespan in flies [6], fish [7], bees [8], and rodents [9]. Other factors related to climate change include the quality of forage [10, 11], beekeeper management practices [12, 13], and pesticide exposure from land use [11]. Although we can try to avoid some of these factors, no effective methods to mitigate the impacts of climate change and prevent bee winter mortality have been reported.

To advance in this direction, it is necessary to clarify the general processes occurring in late summer and fall that are involved in the transition from short-lived to long-lived workers [14, 15], as these processes may be disrupted by climate change. This transition has been associated with external (environmental) factors that influence internal (metabolic) processes.

Known external trigger factors include a decrease in pollen availability [16, 17] and cessation of brood rearing [15, 18]. Ambient temperature, as factor of climate change,

can significantly alter brood temperature in summer [19], despite bees` efforts to maintain a consistent brood microclimate [20-22].

With the onset of fall, external trigger factors initiate the transition of bees through internal processes, leading to the development of winter workers with prolonged longevity, which can last up to 8 months [1]. Briefly, this transition exhibits the classic insect diapause phenotype, characterized by reduced metabolic activity and altered hormonal profiles [14, 23-26]. Unlike other insects, the reduced metabolic activity of bees during diapause [17] includes vibrating their wing muscles to generate heat and maintain warmth within the winter cluster. This process depends on a constant energy source and requires a pure carbohydrate diet [27], which is not necessary for other diapausing insects.

In winter bees, the physiology of internal processes related to this hormone-regulated phenomenon is well-described. It manifests as hypertrophied secretory vesicles and increased acini diameter in the hypopharyngeal gland (HG) compared to newly emerged and forager summer bees [28]. HG development is known to be positively correlated with vitellogenin (*vg*) throughout a worker's lifespan. However, *vg* is synthesized in the fat body [29], which is more developed in winter bees compared to summer bees [27]. The fat body mass results from the accumulation of nutrients [30], with lipids constituting over >90 % of the mass in the form of triglycerides. These triglycerides are synthesized from dietary carbohydrates and fatty acids and are used not only for *vg* synthesis [30] but also for producing pheromones,

phospholipids, and wax [31].

Significant differences between bees and other insects have been observed in the mutually inhibitory interaction between vg (protein) and the juvenile hormone (JH) [32]. An increased level of vg (protein and gene), alongside decreased levels of the JH [1, 15, 33], has been noted, however, the molecular mechanism of this complex relationship remains unclear [34]. It is known that the JH biosynthetic pathway begins in the glands in the brain complex and responds to sensory inputs that underpin the division of labor [34, 35]. Furthermore, at the end of winter, diapause bees gradually transition to nursing brood care and foraging physiology [1]. The last transition is characterized by several physiological and molecular changes, including a decrease in acinus size [36] and fat body mass [37], as well as reductions in vg levels and increases in JH levels [15, 38]. These changes contribute to seasonal effects and behavior [39], leading to reduced longevity and increased aging compared to diapause conditions [40, 41].

Alongside with the well-known age-related marker gene *vg* [32, 42-44], this study examined other potential markers, including the genes *JHAMT, ilp1, ilp2, TOR1*, and *HSP70*. The enzyme involved in JH biosynthesis, *Juvenile Hormone Acid Methyltransferase (JHAMT),* exhibits changes in expression similar to JH titer and can serve as an alternative marker to JH [45-47]. A positive correlation with JH was observed in the *insulin-like peptide, ilp1,* whose expression increases in the brains of foragers and decreases with continued foraging [48]. The *insulin-like peptide, ilp2,*

gene may have task-related expression as it is highly expressed in nurse tissue and plays a role in increasing cell size and number in individual organs [49]. Additionally, the *TOR1* gene, a nutrient-sensing kinase, along with the *ilp1* and *ilp2* genes, participates in the mobilization of nutritional resources (autophagy) from tissues such as the fat body and glands in *Drosophila* [50]. Moreover, the *TOR* pathway is implicated in disease and nutrition in insects [51-53], and mammals [54], and it also regulates longevity extension in social insects [25]. Lastly, the stress marker *heat shock protein 70 (HSP70)* was chosen because it is detected at low levels in nurses but shows increased expression in foragers [55]. While all these markers are expected to be important for identifying the physiological state in overwintered honey bees, testing them together in the same experiment requires evaluating their responses to the same triggers.

This research aimed to simulate the influence of global warming on colony performance in previouse seasons and to compare the physiological state of overwintered *Apis mellifera ligustica* bees. The experiment involved maintaining colonies at ambient constant temperatures of 25°C (Group 25) and 35°C (Group 35) in controlled rooms, representing the average and high summer temperatures in Incheon (Republic of Korea), respectively. A Group control was kept under natural conditions. The temperature experiments continued throughout the summer, September, and October, with the colonies overwintering under the same conditions. Colony performance, including the brood microclimate, was assessed in August and

September, during the transition from summer to winter physiology in the Republic of Korea. In February, we compared the long-term effects of ambient temperature on the physiological and molecular state of the winter bees at the end of diapause (broodless period) using eight markers. The two physiological markers were acinus size and lipid content in the fat body. The six molecular markers were for: tasks (*vg* and *JHAMT*), nutrition (*ilp1, ilp2*, and *TOR1*), and stress response (*HSP70*). We believe this study can lead to further research to identify the environmental causes and prevent honey bees losses early.

Methods

Experimental bees

Western bees (*Apis mellifera ligustica*) were obtained from nine colonies kept at the Incheon National University apiary in the Republic of Korea **(Fig 1A, B, C, and D),** managed by a professional beekeeper following standard beekeeping practices [56]. The experimental colonies contained first-year young sister queens, naturally mated which started to lay eggs in May 2022. Prior to the experiment in June, colonies were standardized in terms of the number of frames (total 4), brood population (3 frames), honey amount (1 frame), and the number of bees (around 3000~3500 bees). Colony strength was assessed through visual inspection of adult population and brood area both before and during the experiment [56]. Two rooms equipped with a climate-control system were established to maintain bee colonies at 25°C (Group 25) and 35°C

(Group 35), simulating global warming without day and night temperature differences.

Fig 1. Overview of the experimental design (A) The room environment before the experiment. **(B)** The process of honey bees freely flying outside from the experimental room. **(C)** The view of the room and the entrance for bees outside. **(D)** Timeline of the experiment. **(E)** Ambient temperature of the Group control, Group 25 (green line), and Group 35 (red line) under the temperature experiment in 2022, Incheon, Republic of Korea. The data were obtained from https://weatherspark.com/h/m/142040/2022/10/Historical-Weather-in-October-2022-in-Incheon-South-Korea#Figures-Temperature.

These temperatures were selected to mimic the effects of global warming, with 25°C representing the summer average and 35°C being the highest recorded in Incheon city under typical conditions. However, the highest temperature was not naturally reached during the summer of 2022 **(Fig 1E).** Other environmental conditions were not controlled. The ceiling light was used during colony inspections. In the third room housing bee colonies, the control group was provided with free ventilation without additional heating. All bees had equal opportunities to collect pollen and nectar around the apiary.

On September 18th and 25th, 2022, two combs with sealed brood were placed in an incubator set at 34°C. Upon hatching, around 200 workers were individually marked with colored paint on their thoraxes and then reintroduced into their original colony. Throughout summer, as well as in September and October of 2022, the honey bee colonies were subjected to the according ambient temperatures. Starting from

November 2022, all colonies were kept under natural conditions and successfully overwintered. The success of colonies overwintering was monitored on February 5th and 27th, when we recorded whether the bees survived (+) or did not survive (-) (**S1 Table**).

The overwintered marked bees, aged approximately 4.5 months, were sampled on February 5th, 2023, a time when queens had not yet begun laying eggs. Forty bees from each colony were sampled and individually placed in labeled 50 ml tubes, then stored at -80°C before analysis.

Brood microclimate measurements

Temperature, humidity (AM2320, BeeOnFarm, Korea), and CO_2 concentration (BO-100, BeeOnFarm, Korea) sensor systems were placed on the top and middle of each colony in August 2022. The data logging system was included in the sensor system because it provided a real-time clock. Data consisting of time (seconds), CO_2 (in ppm), temperature (in °C), and relative humidity (as a percentage) were saved **(S1 File)**. The data from 6 colonies kept in each room (three at 25°C and three at 35°C) were collected over 15 days from 11 to 25 August. Average values for the day were used for statistical calculations.

Hypopharyngeal gland (HG) measurements

Acinus size from the hypopharyngeal glands of ninety overwintered bees in each

Group (control, 25°C, and 35°C) were measured to determine if there were differences based on the ambient temperature during the previous seasons. Thirty bees were collected from each colony, flash frozen in liquid nitrogen, and maintained at −80°C until their glands were measured **(S2 File)**. For each bee, the HGs were dissected into 1× phosphate-buffered saline (PBS) with 0.1% Tween® 20 and visualized at 40× magnification. The size (mm^2) of at least ten randomly selected acini per bee was measured using a Nikon Eclipse E200 microscope (Nikon, Tokyo, Japan) equipped with ToupView software (Touptek, Hangzhou, China) installed on a PC. Only acini with clearly defined borders were included. Acinus size was averaged within each individual.

Fat body lipid content

The fat body lipid content in the abdomen of overwintered honey bees was studied in early February by the standard method [57, 58]. Ten bees per colony were collected; the ninety bees were flash frozen in liquid nitrogen and maintained at −80°C until their abdomens were dissected and their guts were removed. Pools of ten abdomens per colony were weighed on a scale (HS220S, HANSANG Instrument Co., Ltd., Republic of Korea) before and after drying to a constant weight in a fume hood (SSFH-1000, Shinsaeng, Republic of Korea). The total lipid content was determined by measuring the decrease in weight following the removal of lipids from the dried tissue through extraction in 5 ml portions of diethyl ether (179272-1 L; Sigma–Aldrich, USA) **(S2**

File).

RNA Extraction and cDNA Synthesis

A total of ninety bees (ten winter bees from each colony) were sampled in labeled tubes and stored at -80°C. The brain and gut-free abdomen of five sampled bees from each colony (fifteen bees for treatment) were dissected on ice, and RNA was extracted directly. RNA was extracted from the brain for the analysis of *HSP70, JHAMT, ilp1, ilp2*, and *TOR1* expression, and from the abdomen for *vg* gene expression analysis, as this gene is expressed in the fat body located in the abdomen. Genes are measured in the brain to understand their roles in stress responses (*HSP70*), metabolic regulation (*JHAMT* and *TOR*), and neural function (*ilp1* and *ilp2*). Total tissue RNA (brain or abdomen) was extracted using a Qiagen RNeasy Mini Kit (#74,104; Qiagen, Valencia, CA, USA). The total RNA concentration and purity were quantified using OD260/OD280 values between 1.8 and 2.0. Next, reverse transcription was performed using an RNA to cDNA EcoDryTM Premix (Oligo dT) kit (Takara, Japan). The reverse transcription reaction mixture included 50 ng/µl total RNA (with the clear volume calculated for each sample) and RNase-free water for a total volume of 20 µl. Reverse transcription was conducted at 42°C for 60 min, followed by heating at 70°C for 10 min.

Quantitative real-time PCR

The relative expression of six genes (*vg, ilp1, ilp2, TOR1, JHAMT,* and *HSP70)* was measured. The housekeeping gene *RpL32* (Ribosomal protein L32*)* was used as an endogenous control. The PCR primer sequences are shown in **S2 Table.**

The reaction conditions were optimized. The qRT–PCR mixture had a volume of 20 µl which included 2 µl of template cDNA, 10 µl of Green qPCR Master Mix, 1 µl of upstream and downstream primers (5 pM/µl), and 6 µl of nuclease-free water (AM9930, Invitrogen, USA). Quantitative real-time PCR (qRT–PCR) was conducted using Brilliant III Ultra-Fast SYBR® Green qPCR Master Mix (600882, Agilent Technologies, USA) on an AriaMx Real-Time PCR System (Agilent Technologies LDA, Malaysia) with AriaMx 96 Well Plates, Skirted, LP (401490, Agilent Technologies, USA) covered by MicroAmp™ Optical Adhesive Film (4311971, Thermo Fisher Scientific, USA).

The qRT–PCR amplification procedure was as follows: initial denaturation at 95°C for 10 minutes; 40 cycles of denaturation at 95°C for 30 seconds, annealing at 60°C for 25 seconds, and extension at 72°C for 15 seconds. Each sample was replicated three times. Data analysis was performed using Agilent AriaMx version 2.0 analysis software. Relative gene expression data were analyzed using the 2^(-Delta Delta C(T)) method [59, 60] (**S3 File**).

Confirmation of amplicons in the RT–PCR products was performed by separating them through electrophoresis in a 2% agarose gel at 80 V for 40 minutes and analyzing them using a gel documentation system, the Gerix 1010 transilluminator

(Biostep GmbH, USA) (**S2 Fig**). A Dyne 50 bp DNA Ladder (Cat. No. A701, DYNEBIO, Korea) was used as a reference.

Statistical analysis

The statistical analysis, designed as illustrated in **Fig 2**, were conducted using Microsoft Excel and XLSTAT software (Addinsoft Pearson Edition 2014, Addinsoft, Paris, France). The characterization was performed using the following strategies [61]: (a) classification of the marker dataset to find a combination of features that best separates different groups (supervised approach, Discriminant Analysis (DA)); (b) clustering to discover natural groupings in the absence of labeled information (unsupervised approach, Agglomerative Hierarchical Clustering (AHC)); and (c) modeling the relationship between independent variables and a dependent variable and dealing with multicollinearity in the marker dataset to rank the markers by importance for identifying overwintered bee task development (supervised approach, Elastic Net Regression).

Specifically, the bees were categorized into groups, and the variables with the highest discrimination scores were selected using DA because it determines the linear combination of variables and provides maximal separation between groups. To discriminate the groups, Fisher distances were applied, which emphasize the linear combinations of variables that maximize the differences between groups.

AHC analysis provided a visual representation of the clustered dataset, revealing the

arrangement of individual data points or observations based on their dissimilarity. The evaluation metric for clustering algorithms was the silhouette index. The score is bound between (− 1) for incorrect clustering and (+ 1) for highly dense clustering.

Fig 2. Experimental conditions, sampling schedule, data sets, and statistical tests used for analysis.

Elastic Net Regression analysis was used to test three models of the relationship [61, 62] between temperatures, physiology and molecular markers. The dependent variables were ambient temperatures, acinus size and lipid content in the fat body. The seven independent variables were ranked by coefficient. Receiver operating characteristic (ROC) curves were generated to quantify how accurately regression analysis can discriminate between honey bee groups [63]. The ROC specificity and sensitivity plot demonstrated a perfectly fitted model linking physiology to molecular markers (gene expression profile) (**S1 Fig**). The area under the curve (AUC) can range from 0.5 to 1.0, with a preference for higher scores, meaning that the model effectively distinguishes between the two classes.

The mean, standard deviation, and variance of each gene were calculated using descriptive statistics and visualized using the heat map module. For the gene expression analysis, ANOVA was used to test overall effects, followed by the Duncan's post hoc test ($p < 0.05$) for multiple comparisons and the t-test for differences of means between two groups of bees (**S4 File**).

Results

The experiment was conducted on nine colonies of western honey bees (*Apis mellifera ligustica*). Each Group (control, 25°C, and 35°C) was represented by three colonies. The bees in Groups 25 and 35 experienced constant diurnal ambient temperatures (modeling global warming) from June to October, while the room for the Group control was freely ventilated to allow for natural ambient temperatures. All bees experienced natural foraging outside without restrictions. Newly emerged bees from each Group were marked in September for the overwintering experiment in February.

Dynamics of ambient temperature in summer and fall months

The daily temperature in the first experimental room was set at 25°C for five months, and there were no significant differences compared to the natural daily average temperature in the summer months or September (t-test, $p > 0.05$). The difference in experimental conditions between the Group control and the Group 25 was due to diurnal temperature fluctuations, which were eliminated in Group 25, where warmer days and cooler nights did not affect the bees. In our experiment, this stable elevated night temperature in Group 25 simulated a global warming scenario. However, the temperature in room Group 25 was significantly higher than the average daily temperature in October 2022 (t-test, $p < 0.0001$). The daily temperature in the second room was set at 35°C for five months, which was significantly greater than the average diurnal temperatures during the summer months, September, and October (t-test, $p <$

0.0001).

Brood microclimate and colony development in August and September

In this study, we hypothesized that the regulation of the nest climate by colony performance influences the physiology of winter bees and the success of overwintering. The temperature, humidity, CO_2, number of larvae, capped brood area, and bees' measurements from August 08 to September 18, 2022 were evaluated, when short-lived bees commenced rearing larvae that would develop into long-lived bees for the overwintering period. Since the expected ambient temperature in August and early September and the comparison of the colony performance of Group control and Group 25 on August 08 (**Fig 3 D, E, F**) showed no significant differences, sensors were not set up in the Group control.

Fig 3. Colony performance in honey bees treated under different ambient temperatures (08 August – 18 September 2022). (A) Temperature in the brood area (°C). **(B)** Humidity in the brood area (%). **(C)** Concentration of CO_2 in the brood area (ppm). **(D)** Number of larvae. **(E)** Number of capped brood cells. **(F)** Number of bees. Statistical significance was determined using a t-test in **Fig 3A, B**, and **C** ($p < 0.01$ for 3A, indicated by different letters above the columns) to compare the means of the two groups, one-way ANOVA, Duncan's post hoc tests ($p < 0.05$ for 3D and 3F, $p < 0.01$ for 3E, indicated by different letters above the columns) to compare the means of the three groups in **Fig 3D, E**, and **F**; bars in the graphs represent the mean ± SD.

SD – Standard Deviation, NS – No Significance. Honey bee colonies in Group 25 were maintained in room conditions at 25°C; colonies in Group 35 were kept in room conditions at 35°C; colonies from Group control were maintained under natural conditions.

The mean and standard deviation of the brood temperature for bee colonies kept at 25°C and 35°C were 30.00 ± 0.98 and 33.40 ± 0.15 °C, respectively. Similarly, the brood humidities for these groups were 60.00 ± 0.43 and 64.72 ± 8.65 %, and the brood CO_2 concentrations were 813.55 ± 296.80 and 633.30 ± 117.00 ppm, respectively. Importantly, the brood temperature inside the colonies kept at 35°C was significantly greater than that inside the colonies kept at 25°C (t-test, $p < 0.01$) (**Fig 3A**). However, brood humidity, CO_2 concentration, number of larvae, capped brood cells, and bees were not significantly differing **(Fig 3B, C**: t-test, $p > 0.05$) (**Fig 3D, E,** and **F:** ANOVA, Duncan's post hoc tests $p > 0.05$). The control colonies, however, exhibited significantly (ANOVA, $p < 0.01$) larger capped brood area, more larvae, and a higher number of bees (ANOVA, $p < 0.05$) on September 18 compared to colonies in Groups 25 and 35.

Distinguishing honey bee groups through marker ranking

The overwintered bees were distinguished by their physiological state using a dataset comprising two established physiological markers (acinus size area and lipid content in the fat body) and six molecular markers (*vg, TOR1, JHAMT*, *ilp1, ilp2,* and *HSP70*). Data Mining methods, including Discriminant Analysis (DA), Agglomerative Hierarchical Clustering (AHC), and Elastic Net Regression, were employed in the

analysis.

DA was performed to plot the Groups control, 25 and 35, and to identify the optimal combination of features that effectively distinguishes them. The Wilcoxon Lambda test within the DA yielded a statistically significant result (two-tailed test, $p < 0.05$), indicating that the model successfully differentiated between groups (**Fig 4A**). The observed Wilks' lambda value of 0.0 signifies a robust separation of the Groups control, 25, and 35, demonstrating a high level of discriminative power. A comparison revealed significant differences between Group 25 and Group 35 ($F(2,8) = 19.37$; two-tailed test, $p < 0.05$; **Fig 4A**).

Fig 4. The discrimination of overwintered honey bees was performed using eight variables related to aging: two physiological variables (acinus size, n = 30; lipid content in the fat body, n = 10) and six molecular variables (n = 10), including the expression levels of *HSP70, ilp1, ilp2, JHAMT, TOR1,* and *vg* genes. (A) Discriminant Analysis (DA). **(B)** Agglomerative Hierarchical Clustering (AHC). **(C)** Heatmap with cluster analysis based on ddCt values, acinus size (mm^2), and the lipid content in the fat body (mg).

The Fisher distances (FDs) in the DA between the Groups control, 25, and 35 were significantly different (FD = 27511; $p < 0.05$ and FD = 19131; $p < 0.05$, respectively). Moreover, the FD density was also significantly different between the Group control and Group 25 (FD = 784; $p < 0.05$). The bees were separated along the primary discriminant axis F1 in 99.92% accuracy, where discriminant function coefficients for gene expressions *vg, HSP70*, and *JHAMT* were -20.09, -14.96, and 23.58, respectively.

These coefficients were the highest, providing significant role to this separation.

Subsequently, AHC was employed to identify natural groupings without labeled information using a dissimilarity measure. The evaluation metric for clustering algorithms, the Silhouette index between clusters 1 and 2, was 0.657, indicating successful clustering, albeit with moderate density **(Fig 4B, C).**

Finally, Elastic Net Regression was used to rank the markers by importance by modeling the relationships between eight **(Fig 5A)** or seven **(Fig 5 B, C)** independent variables and one dependent variable**.** This analysis was conducted separately for ambient temperature **(Fig 5A)**, fat body mass **(Fig 5B)** and acinus size **(Fig 5C).** The ambient temperature model identified significant predictors, with variable acinus size having the highest positive coefficient (305.773) and variable *TOR1* and *vg* having the highest negative coefficients (-29.969) and (-14.627) respectively. In the acinus size model, significant predictors were identified, with the variable *vg* having the highest positive coefficient (0.009) and the variable fat body mass having the highest negative coefficient (-0.009). In this all models *vg* showed high coefficient values.

Fig 5. Elastic Net Regression modeling the relationship between dependent variables ambient temperatures (settings in summer and fall) (A), lipid content in the fat body (in February) (B), and acinus size (in February) (C) and the independent variables (*HSP70, ilp1, ilp2, TOR1, JHAMT, vg,* and acinus size or lipid content of fat body where appropriate, in February) to rank the variables by importance for predicting dependent variables.

Physiological markers of honey bees

These results are based on the two physiological markers, analysis of lipid content in the fat body and the acinus size of the hypopharyngeal glands (HGs) in overwintered bees. The mean and standard deviation of the lipid content for the bees in the Groups control, 25, and 35 were 4.33 ± 1.25, 4.00 ± 1.63, and 2.00 ± 0.82 mg/bee, respectively, with no significant differences (ANOVA, Duncan's post hoc, P = 0.22, $p > 0.05$), even though the lipid content in Group 35 was slightly lower **(Fig 6A).**

Fig 6. Physiological markers of long-lived honey bees at the end of winter (February) (A) The average of lipid content in the fat body, n = 10 (mean ± SD). **(B)** Average acinus size, n = 30 (mean ± SD). Statistical significance was determined using one-way ANOVA, Duncan's post hoc tests ($p < 0.05$, indicated by different letters above the columns).

Similarly, the mean and standard deviation of the size of the bee acinus from the Groups control, 25, and 35 were 0.026 ± 0.001, 0.027 ± 0.003, and 0.018 ± 0.001 mm^2, respectively. Notably, the acini in bees from Group 35 were significantly smaller (ANOVA, P = 0.006, $p < 0.01$) than in bees of the Group control and 25 **(Fig 6B).** Importantly, both the lipid content and the acinus size of the overwintered bees in Group 35 were lower than those in Groups control and 25 **(Fig 6)**, suggesting lower nutrition and different physiological state in bees from Group 35.

Molecular markers of honey bees

These results include two previously defined task-related markers, *vg* and *JHAMT,* as well as four genes predicted to respond to physiology: *TOR1, ilp1, ilp2*, and *HSP70* **(Fig. 7).**

Fig 7. Gene expression analysis. The gene expression (ddCt) of *vg** **(A),** *TOR1*** **(B),** *JHAMT*** **(C),** *ilp1*** **(D),** *ilp2*** **(E),** and *HSP70*** **(F)** in long-lived honey bees at the end of overwintering (February) was evaluated. Statistical significance was determined using one-way ANOVA, Duncan's post hoc tests ($p < 0.05$, indicated by different lowercase letters above the columns). The bars in the graphs represent the mean ± SD. *n=9. **n=15.

Expression of *vg* gene was significantly upregulated (ANOVA, Duncan's post-hoc test, $p < 0.0001$) in bees from Group control and 25 compared to those from Group 35. In contrast, the expression of the *TOR1, JHAMT, ilp1, ilp2*, and *HSP70* genes was significantly downregulated (ANOVA, $p < 0.001$ for *TOR1*; $p < 0.0001$ for other listed genes) in the same bees, suggesting aging in bees from Group 35 compared to Groups control and 25 **(Fig. 7B, C, D, E,** and **F)**. However, there were no significant differences in the expression of *TOR1, JHAMT*, and *ilp2* (ANOVA, $p > 0.05$) between bees from Group control and Group 25. The expression levels of *ilp1* and *HSP70* were significantly lower (ANOVA, $p < 0.05$) in the ones from Group control than in Group 25.

Discussion

Key triggers influencing the transition of insects from summer to winter physiology

include photoperiod, ambient temperature, brood microclimate, availability of pollen, and some yet-to-be-identified factors [25, 64, 65], which obviously have a particular influence on overwintering physiological state, and also linked to aging and subsequent winter loss in bees.

However, due to the complex and unclear interactions among all these triggers, we designed an experiment in which only one factor associated with global warming - ambient temperature (25, 35°C, and a control), was manipulated during the late summer, September, and October, and kept constant in experimental rooms. Colony development and brood climate regulation were investigated in August and September, during which long-lived (winter) bees were reared and marked to study the physiological state of bees alongside their clear chronological age in February. Physiology was assessed using two physiological markers (acinus size and lipid content in the fat body) and six molecular markers (*vg, TOR, JHAMT, ilp1, ilp2*, and *HSP70*) at the beginning of February, during the expected diapause continuation.

Since other colony parameters (e.g., food availability, hive size, and environmental conditions) excluding ambient temperature were consistent during the experiment (in summer and fall), the observed colony development in the Groups is likely due to the altered thermal environments, which can be the effects of temperature values and the constant diurnal temperature regime. It is crucial during ambient temperature extremes and heat waves since honey bee colonies rely on precise temperature regulation to maintain the brood microclimate [64] and

support metabolic activity [4].

It was found that constant diurnal and seasonal temperatures (25°C and 35°C) significantly impaired colony development in Groups 25 and 35 in the fall season (from September 18 onward), resulting in reduced brood and worker numbers compared to the Group control. Also, brood microclimate was effectively regulated by the bees. Despite a 10°C difference in the experimental ambient temperatures, their efforts reduced this difference to approximately 3°C (**Fig. 3**). Bees in Group 25 raised the brood temperature to about 30°C, while those in Group 35 lowered the brood temperature to around 33°C, slightly below the typical brood temperature of around 34.5°C [67], reflecting well-documented microclimate regulation behaviors [21, 22].

Reduced brood and worker numbers, combined with challenges in brood temperature regulation, suggest that constant temperatures affect colony performance more than ambient temperature alone, as the average ambient temperatures for the Control and 25°C groups in summer showed no significant difference. However, the brood temperature responses in Groups 25 and 35 suggest different survival strategies for the colonies. Although it is unclear whether the number of bees in colonies from Groups 25 or 35 was insufficient to heat or cool the hive, or if the subnormal brood temperature in these Groups were a strategy for rearing long-lived winter bees, these temperatures did not prove fatal for colony survival during overwintering in our experiment (**S1 Table**). Furthermore, since Szentgyörgyi et al. [68] reported that short-lived bees tend to live longer after developing at a lower temperature (32°C), we favor

this explanation. We hypothesize that reducing brood temperature may trigger physiological changes associated with extended longevity (long-live) in winter bees compared to summer bees [69].

All these factors were expected to influence the physiological state of overwintered honey bees in diapause in February, which hatched under experimental temperatures. The known physiological markers of winter bees physiology include hypertrophied hypopharyngeal glands (HG) compared to newly emerged and forager summer bees [28]. This is important because the enlarged HGs of overwintering honey bees secrete more royal jelly to adequately feed the first larvae after diapause [39]. Thus, the nutrition of winter bees includes both the bee's own nutritional needs and the nutritional needs of larvae cared for by nurse bees, which seem to stimulate each other. Another marker is an enlarged fat body compared to summer bees [27], resulting from the accumulation of nutrients, particularly lipids [30]. These lipids support the production of hormones and molecular markers [40, 70, 71], as well as the synthesis of vg protein, which is later transported to the HG as a source of amino acids after diapause ends [72]. Based on this, larger acini in the HG and higher lipid content in the fat body of bees of the same chronological age may indicate a comparatively better physiological state. On February 5, winter bees from Group 25 and Group control, still in diapause, had the best physiological state compared to bees from Group 35, as the acinus size in honey bees from Groups 25 and the Group control was significantly larger than in

those from Group 35. A similar trend was observed in the fat body's lipid content, although the differences were not statistically significant.

The previously mentioned vg (gene and protein) is the most studied molecular marker in the dual suppression model in bees. Its elevation suppresses the levels of juvenile hormone (JH) and genes related to the JH pathway (*JHAMT*) [38]. This relationship has been used to compare the physiological states of both winter and summer bees [1, 33]. These molecular levels remain stable during diapause and undergo a switch before the onset of brood rearing [38]. Notably, *vg* gene expression was significantly higher in bees from Group 25 and the control, while *JHAMT* gene expression was significantly lower compared to Group 35. This indicates a different physiological state and suggests some changes in the aging process in the latter group of bees.

To utilize these well-known interactions, we built Elastic Net Regression models for ambient temperature, acinus size and lipid content in the fat body **(Fig 5A, B** and **C).** In this models *vg* showed high negative (ambient temperature model) and positive (lipid content of fat body and acinus size models) coefficient values. This indicates new knowledge that high ambient temperature is related to *vg* levels in reared honey bees **(Fig 5A)**. It was also shown that during winter diapause, *vg* gene expression highly related to acinus size **(Fig 5C)** and lipid content **(Fig 5B)** at bees under normal conditions. This supports the well-known relationship between *vg,* acinus size, and lipid content of fat body [14, 15, 27, 28, 37]. However, when each characteristic (*vg*,

acinus size and lipid content of fat body) were analyzed separately, we found that in bees from Group control and Group 25, compared to Group 35, this relationship was reversed, suggesting potential physiological issues in bees from Group 35.

To identify genes that can significantly discriminate between the three treatment groups in our study, Discriminant Analysis (DA) was implemented. Three genes were identified with high scores: *JHAMT* and *vg*, which are well-known genes related to physiological states and are also suggested to be associated with aging, and the *HSP70* gene.

Because of *HSP70* gene expression responded on stress [73] and diets along with *vg* in bees during winter [55], we suggest that highest *HSP70* gene expression in bees from Group 35, while the lowest was found in the Group control, responded on a stress, possibly related to their diet. We can suggest that this can be increasing nutritional needs at the end of diapause in bees from Group 35, because the significant increase in the *TOR1* gene expression was observed there. This evolutionarily conserved gene integrates signals from nutrients (amino acids and energy) and growth factors to regulate cell growth [50, 74, 75]. The nutritional needs of bees and the diet selection they consume from colony stock (bee bread and honey) are linked to their physiology. Winter bees in diapause require carbohydrate-rich food (honey) without a protein source, such as bee bread, to produce royal jelly related to *vg* metabolism, as there is no brood during diapause [17, 27]. This protein (vg) will be used after diapause ends, when the bees' HG increase in size to secrete

royal jelly for feeding the larvae [1]. Additionally, significantly higher *HSP70* and *TOR1* gene expressions in bees from Group 35, compared to Group control and Group 25, were associated with a low in fat body lipid content, although it was not yet statistically significant. This supports the hypothesis that honey bees from Group 35 experienced long-term effects of ambient temperature, which are reflected in physiological changes indicated by two new molecular markers (high expression of the *HSP70* and *TOR1* genes), suggesting possible differences in diets and increased nutritional needs.

The gene *ilp1*and *ilp2* did not show a clear effect in our experiment to ambient temperature, which may be due to several factors. First, the interaction of this genes with nutrition, age, and season may involve more subtle or context-specific metabolic pathways that were not fully captured under our experimental conditions [76]. Additionally, *ilp1*and *ilp2* could be influenced by other unmeasured variables that obscure its direct effects [77]. It is also possible that the gene's influence on these factors is non-linear or dependent on specific thresholds, meaning a wider range of experimental conditions may be required to detect its effect. Further investigation is needed to disentangle these complex relationships and better understand how *ilp1* and *ilp2* functions under varying biological contexts.

In conclusion, our results demonstrate that constant diurnal temperatures negatively impact colony development in summer and fall, reflecting broader issues related to global warming, such as reduced brood and worker numbers, increased brood

temperature regulation demands. We hypothesize that reduced brood temperatures trigger physiological changes in bees that may be linked to extended longevity in winter. Notably, bees raised at the higher temperature (Group 35) exhibited distinct physiological states in bees at the end of diapause in February compared to those from Group 25 and the Group control. Comparative analysis revealed that overwintered bees from Group 35 had smaller acini, lower *vg*, and higher *JHAMT* gene expressions. Additionally, we identified two new molecular markers (high expression of *HSP70* and *TOR1* genes), which had increased expression in overwintered bees from Group 35 related to constant diurnal temperature in summer and fall.

While we cannot definitively conclude that bees from Group 35 faced suboptimal conditions, as the physiological changes did not result in fatal outcomes, further research is warranted. These findings enhance our understanding of how organisms respond to increased diurnal temperatures in summer and fall, providing valuable insights for apiculture practices and guidelines for winter management to prevent losses. Global warming during these seasons alters bee physiology for wintering, potentially threatening their longevity. Unlike bees experiencing typical fall conditions in Korea, which can adapt their colony development, those affected by global warming require assistance safeguarding their colonies at the end of winter and early spring.

Supporting information

S1 Table. Survival of honey bee colonies at the end of the overwintering (PDF)

S2 Table. PCR primer sequences. (PDF)

S1 Fig. The quality verification plot (specificity vs sensitivity) of Elastic Net Regression model demonstrated a perfectly fitted model linking physiology to molecular markers (gene expression profile). (.tif)

S1 Fig. Gel electrophoresis to verify the amplicons after qPCR. (.tif)

S1 File. Raw data from sensors and XLSTAT 2022.xlsx

S2 File. Raw data of acinus size, lipid content of the fat body, and colony perfomance.xlsx

S3 File. Raw data qPCR.xlsx

S4 File. Stat analysis in XLSTAT.xlsx

S5 File. Raw data weather and statistics.xlsx

S6 File. Raw data and stat colony development.xlsx

Acknowledgments

The authors thank BeeOnfarm company (Republic of Korea) for providing the equipment to record the in-hive microclimate. Mention of trade names or commercial products in this publication is solely to provide specific information and does not imply recommendation or endorsement by the INU.

Author Contributions

Conceptualization: Yumi Yun, Hyung-Wook Kwon

Data curation: Yumi Yun

Formal analysis: Olga Frunze, Ravil R. Garafutdinov, Hyunjee Kim

Investigation: Hyung-Wook Kwon, Young-Eun Na

Methodology: Ravil R. Garafutdinov, Olga Frunze, Yumi Yun

Resources: Hyung-Wook Kwon, Young-Eun Na, Hyunjee Kim

Software: Olga Frunze, Ravil R. Garafutdinov

Supervision:, Hyung-Wook Kwon, Young-Eun Na

Visualization: Olga Frunze

Writing-original draft: Olga Frunze, Yumi Yun

Writing-review&editing: Olga Frunze, Yumi Yun, Hyunjee Kim, Hyung-Wook Kwon

Reference

1. Döke MA, Frazier M, Grozinger CM. Overwintering honey bees: biology and management. Curr Opin Insect Sci. 2015; 10:185-93.
2. Hristov P, Shumkova R, Palova N, Neov B. Factors associated with honey bee colony losses: a mini-review. Vet Sci. 2020; 7:166.
3. Gray A, Adjlane N, Arab A, Ballis A, Brusbardis V, Dougla AB. et al. Honey bee colony loss rates in 37 countries using the COLOSS survey for winter 2019–2020: the combined effects of operation size, migration and queen replacement. J Apic Res. 2023; 62:204-10.

4. Calovi M, Grozinger CM, Miller DA, Goslee SC. Summer weather conditions influence winter survival of honey bees (*Apis mellifera*) in the northeastern United States. Sci Rep. 2021; 11: 1553.
5. Dosio A, Mentaschi L, Fischer EM, Wyser K. Extreme heat waves under 1.5°C and 2°C global warming. Environ Res. 2018; 13: 054006.
6. Lamb MJ. Temperature and lifespan in *Drosoph.* Nat. 1968; 220: 808–809.
7. Liu RK, Walford RL. Increased growth and lifespan with lowered ambient temperature in annual fish *Cynolebias adloffi*. Nature. 1966; 212: 1277–78.
8. Mishra DM, Bhunia P, Shubham S, Sen,R, Bhunia R, Gulia J, et. al. The impact of weather changes on honey bee populations and disease. J Adv Zool. 2023; 44: 180–90.
9. Conti NB, Sanchez-Alavez M, Winsky-Sommerer R, Morale MC, Lucero J, Brownell S, et al. Transgenic mice with a reduced core body temperature have an increased life span. Science. 2006; 314, 825-8.
10. Fahrenholz L, Lamprecht I, Schricker B. Thermal investigations of a honey bee colony: thermoregulation of the hive during summer and winter and heat production of members of different bee castes. J Comp Physiol. 1989; 159: 551-60.
11. Switanek M, Crailsheim K, Truhetz H, Brodschneider R. Modelling seasonal effects of temperature and precipitation on honey bee winter mortality in a temperate climate. Sci Total Environ. 2017; 579: 1581–7.
12. van Dooremalen C, Gerritsen L, Cornelissen B, van der Steen JJ, van Langevelde F.,

Blacquière T. Winter survival of individual honey bees and honey bee colonies depends on level of *Varroa destructor* infestation. PloS ONE. 2012; 7: e36285.

13. Morawetz L, Köglberger H, Griesbacher A, Derakhshifar I, Crailsheim K, Brodschneider R, et al. Health status of honey bee colonies (*Apis mellifera*) and disease-related risk factors for colony losses in Austria. PLoS ONE. 2019; 14: e0219293.
14. Fluri P, Lüscher M, Wille H, Gerig L. Changes in weight of the pharyngeal gland and hemolymph titers of juvenile hormone, protein and vitellogenin in worker honey bees. J Insect Physiol. 1982; 28:61-8.
15. Fluri P, Wille H, Gerig L, Luscher M. Juvenile hormone, vitellogenin and hemocyte composition in winter worker honeybees (*Apis mellifera*). Experientia 1977; 33: 1240–41.
16. Crailsheim K. Dependence of protein-metabolism on age and season in the honeybee (*Apis mellifica carnica* Pollm). J. Insect Physiol. 1986; 32:629–34.
17. Denlinger DL. Insect diapause: from a rich history to an exciting future. J Exp Biol. 2023; 226 (4): 245329.
18. Münch D, Kreibich CD, Amdam GV. Aging and its modulation in a long-lived worker caste of the honey bee. J Exp Biol. 2013; 216: 1638–49.
19. Coulibaly KA, Majeed MZ, Sayed SM, Yeo K. Simulated climate warming influenced colony microclimatic conditions and gut bacterial abundance of honeybee subspecies *Apis mellifera ligustica* and *A. mellifera sinisxinyuan*. J Apic

Sci. 2022; 66: 15-27.

20. Simpson J. Nest Climate regulation in honey bee colonies. Science. 1961; 133:1327–1333.

21. Abou-Shaara H, Owayss A, Ibrahim Y, Basuny N. A review of impacts of temperature and relative humidity on various activities of honey bees. Insectes Soc. 2017; 64: 455 – 63.

22. Bencsik M, McVeigh A, Tsakonas C, Kumar T, Chamberlain L, Newton MI. A Monitoring system for carbon dioxide in honeybee hives: An indicator of colony health sensors (Basel). 2023; 23(7):3588.

23. Winston ML. The Biology of the honey bee. Harvard University Press, Cambridge. 1987; 294.

24. Hartfelder K, Engels W. Social insect polymorphism: hormonal regulation of plasticity in development and reproduction in the honeybee. Curr Top Dev Biol. 1998; 40:45-77.

25. Rodrigues MA, Flatt T. Endocrine uncoupling of the trade-off between reproduction and somatic maintenance in eusocial insects. Curr Opin Insect Sci. 2016; 16:1e8.

26. Bresnahan ST, Döke MA, Giray T, Grozinger CM. Tissue-specific transcriptional patterns underlie seasonal phenotypes in honey bees (*Apis mellifera*). Mol Ecol. 2022; 31:174-84.

27. Brejcha M, Prušáková D, Sábová M, Peska V, Černý J, Kodrík D, et al. Seasonal changes in ultrastructure and gene expression in the fat body of worker honey bees.

J Insect Physiol. 2023; 146:104504.

28. Deseyn J, Billen J. Age-dependent morphology and ultrastructure of the hypopharyngeal gland of *Apis mellifera* workers (Hymenoptera, Apidae) Apidologie. 2005; 36: 49-57.

29. Dohanik VT, Medeiros-Santana L, Santos CG, Santana, WC, Serrão JE. Expression and function of the vitellogenin receptor in the hypopharyngeal glands of the honey bee *Apis mellifera* (Hymenoptera: Apidae) workers. Arch Insect Biochem Physiol. 2024; 116: e22120.

30. Smedal B, Brynem M, Kreibich C, Amdam G. Brood pheromone suppresses physiology of extreme longevity in honeybees (*Apis mellifera*). J Exp Biol. 2009; 212: 3795-801.

31. Arrese EL, Soulages JL. Insect fat body: energy, metabolism, and regulation. Annu Rev Entomol. 2010; 55: 207-25.

32. Amdam GV, Aase AL, Seehuus SC, Kim Fondrk M, Norberg K, Hartfelder K. Social reversal of immunosenescence in honey bee workers. Exp Ger Ontol. 2005; 40: 939-47.

33. Knoll S, Pinna W, Varcasia A, Scala A, Cappa MG. The honey bee (*Apis mellifera* L., 1758) and the seasonal adaptation of productions. Highlights on summer to winter transition and back to summer metabolic activity. A review. Livest Sci. 2020; 235: 104011.

34. Schilcher F, Scheine R. New insight into molecular mechanisms underlying division

of labor in honeybees. Curr Opin Insect Sci. 2023; 59: 101080.

35. Scheiner R, Reim T, Søvik E, Entler BV, Barron AB, Thamm M. Learning, gustatory responsiveness and tyramine differences across nurse and forager honeybees. J Exp Biol. 2017; 220:1443-50.

36. Corby-Harris V, Snyder LA. Measuring hypopharyngeal gland acinus size in honey bee (*Apis mellifera*) workers. J Vis Exp. 2018; 139:58261.

37. Corby-Harris V, Snyder L, Meador C. Fat body lipolysis connects poor nutrition to hypopharyngeal gland degradation in *Apis mellifera*. J Insect Physiol. 2019; 116:1-9.

38. Amdam GV, Omholt SW. The regulatory anatomy of honeybee lifespan. J Theor Biol. 2002; 216: 209-28.

39. Ali H, Alqarni AS, Iqbal J, Owayss AA, Raweh HS, Smith BH. Effect of season and behavioral activity on the hypopharyngeal glands of three honey bee *Apis mellifera* L. races under stressful climatic conditions of central Saudi Arabia. J Hymenopt Res. 2019; 68: 85-101.

40. Alaux C, Soubeyrand S, Prado A, Peruzzi M, Maisonnasse A, Vallon J et al. Measuring biological age to assess colony demographics in honeybees. PLoS ONE. 2018; 13(12): e0209192.

41. Diebel LWM, Rockwood K. Determination of biological age: geriatric assessment *vs* biological biomarkers. Curr Oncol Rep. 2021; 23: 104.

42. Elekonich MM, Schulz DJ, Bloch G, Robinson GE. Juvenile hormone levels in

honey bee (*Apis mellifera* L.) foragers: foraging experience and diurnal variation. J Insect Physiol. 2001; 47:1119-25.

43. Guidugli KR, Nascimento AM, Amdam GV, Barchuk AR, Omholt S, Simoes ZL et al. 2005. Vitellogenin regulates hormonal dynamics in the worker caste of a eusocial insect. FEBS Lett. 2005; 579: 4961-65.
44. Amdam GV, Page RE. The developmental genetics and physiology of honeybee societies. Anim Behav. 2010;79(5): 973-80.
45. Goodman WG, Granger NA. The juvenile hormones. In: Lawrence IG, Kostas I, Sarjeet SG. Comprehensive Molecular Insect Science. Elselvier. 2005; pp. 319-408.
46. Li W, Huang ZY, Liu F, Li Z, Yan L, et al. Molecular cloning and characterization of juvenile hormone acid methyltransferase in the honey bee, *Apis mellifera*, and its differential expression during caste differentiation. PLoS ONE. 2013; 8:e68544.
47. Egi Y, Sakamoto K. Genome-wide screening of genes involved in programming diapause in the next generation in silkworm, *Bombyx mori* (Lepidoptera: Bombycidae). Eur J Entomol. 2022; 119:405-12.
48. Corona M, Velarde RA, Remolina S, Moran-Lauter A, Wang Y, Hughes KA et al. Vitellogenin, juvenile hormone, insulin signaling, and queen honey bee longevity. Proc Natl Acad Sci USA. 2007; 104: 7128-33.
49. Zhang W, Wang L, Zhao Y, Wang Y, Chen C, Hu Y, et al. Single-cell transcriptomic analysis of honeybee brains identifies vitellogenin as a caste differentiation-related factor. iScience. 2022; 25: 104643.

50. Barth JMI, Szabad J, Hafen E, Köhler K. Autophagy in *Drosophila* ovaries is induced by starvation and is required for oogenesis. Cell Death Differ. 2010; 18: 915.

51. Patel A, Fondrk MK, Kaftanoglu O, Emore C, Hunt G Frederick K, et al. The making of a queen: TOR pathway is a key player in diphenic caste development. PLoS ONE. 2007; 2: e509.

52. Laplante M, Sabatini DM. mTOR signaling in growth control and disease. Cell. 2012; 149:274-293.

53. Johnson SC, Rabinovitch PS, Kaeberlein M. mTOR is a key modulator of aging and age-related disease. Nature. 2013; 493:338-345.

54. Zoncu R, Efeyan A, Sabatini DM. mTOR: from growth signal integration to cancer, diabetes and aging. Nat Rev Mol Cell Biol. 2011; 12:21-35.

55. Sarioğlu-Bozkurt A, Topal E, Güneş N, Üçeş E, Cornea-Cipcigan M, Coşkun İ, et al. Changes in vitellogenin (*Vg*) and stress protein (*HSP 70*) in honey bee (*Apis mellifera anatoliaca*) groups under different diets linked with physico-chemical, antioxidant and fatty and amino acid profiles. Insects. 2022; 13: 985.

56. Delaplane KS, van der Steen J, Guzman-Novoa E. Standard methods for estimating strength parameters of *Apis mellifera* colonies. J Apic Res. 2013; 52: 1-12.

57. Martin JS. Lipid composition of fat body and its contribution to the maturing oöcytes in *Pyrrhocoris apterus*. J Insect Physiol. 1969; 15:1025-45.

58. Human H, Brodschneider R, Dietemann V, Dively JD, Ellis E, Forsgren I, et al. Miscellaneous standard methods for *Apis mellifera* research. J Apic Res. 2013; 52:1-

53.

59. Livak KJ, Schmittgen TD. Analysis of relative gene expression data using real-time quantitative PCR and the 2(-Delta Delta C(T)) Method. Methods. 2001; 25:402–408.

60. Bustin SA, Benes V, Garson JA, et al. The MIQE guidelines: minimum information for publication of quantitative real-time PCR experiments. Clin Chem. 2009; 55:611-22.

61. Moses AM. Statistical modeling and machine learning for molecular biology. CRC Press Taylor & Francis Group. 2017: 280 p.

62. Jaqaman K, Danuser G. Linking data to models: data regression. Nat Rev Mol Cell Biol. 2006; 7:813–819.

63. Hajian-Tilaki K. Receiver operating characteristic (ROC) curve analysis for medical diagnostic test evaluation. Caspian J Intern Med. 2013; 4: 627-35.

64. Medrzycki P, Sgolastra F, Bortolotti L, Bogo G, Tosi S, Padovani E, et al. Influence of brood rearing temperature on honey bee development and susceptibility to poisoning by pesticides. J Apic Res. 2010; 49:52-59.

65. Quinlan GM, Feuerborn C, Hines HM, Grozinger CM. Beat the heat: thermal respites and access to food associated with increased bumble bee heat tolerance. J Exp Biol. 2023; 226: jeb245924.

66. Stalidzans E, Berzonis A, Temperature changes above the upper hive body reveal the annual development periods of honey bee colonies. Comput Electron Agric. 2013; 90: 1-6.

67. Kovac H, Stabentheiner A, Brodschneider R. Contribution of honeybee drones of different age to colonial thermoregulation. Apidologie. 2009; 40:82-95.
68. Szentgyörgyi H, Czekońska K., Tofilski A. Honey bees are larger and live longer after developing at low temperature. J Therm Biol. 2018; 78:219-226.
69. Kovac H, Crailsheim K. Lifespan of *Apis mellifera carnica* Pollm. infested by *Varroa jacobsoni* Oud. in relation to season and extent of infestation. J Apic Res. 1988; 27: 230-8.
70. Kaur R, Singh D. Molecular markers a valuable tool for species identification of insects: A review. Ann Entomol. 2020; 38:1-20.
71. Daniels BC, Wang Y, Page RE Jr, Amdam GV. Identifying a developmental transition in honey bees using gene expression data. PLoS Comput Biol. 2023; 9(9):e1010704.
72. Ramanathan ANKG, Nair AJ, Sugunan VS. 2018. A review on royal jelly proteins and peptides. J Funct Foods. 2018; 44: 255-64.
73. Zhang Y, Liu Y, Zhang J, Guo Y, Ma E. Molecular cloning and mRNA expression of heat shock protein genes and their response to cadmium stress in the drasshopper *Oxya chinensis*. PLoS ONE. 2015; 10: e0131244.
74. Fingar D, Blenis J. Target of rapamycin (TOR): an integrator of nutrient and growth factor signals and coordinator of cell growth and cell cycle progression. Oncogene. 2004; 23: 3151–71.
75. Fahrbach SE, Nambu JR, Schwartz LM. Programmed cell death in insects. Insect

Mol Biol Biochem. 2012; 12:419–449.

76. Nilsen KA, Ihle KE, Frederick K, Fondrk MK, Smedal B, Hartfelder K, et al. Insulin-like peptide genes in honey bee fat body respond differently to manipulation of social behavioral physiology. J Exp Biol. 2011; 1:1488-97.
77. Chowański S, Walkowiak-Nowicka K, Winkiel M, Marciniak P, Urbański A, Pacholska-Bogalska J. Insulin-like peptides and cross-talk with other factors in the regulation of insect metabolism. Front Physiol. 2021; 12:701203.

Data Availability Statement: All relevant data are the manuscript and its Supporting information files.

Funding: This work was carried out with the support of the Cooperative Research Program for Agriculture Science & Technology Development (Project No. RS-2023-00232749) and the Priority Research Centers Program through the National Research Foundation of Korea (NRF) funded by the Ministry of Education (2020R1A6A1A03041954). The funders had no role in study design, data collection and analysis, decision to publish, or preparation of the manuscript.

Competing interests: The authors have declared that no competing interests exist.

Abbreviations: DA, discriminant analysis; AHC, Agglomerative Hierarchical Clustering; FD, Fisher distance; HG, hypopharyngeal glands; JH, juvenile hormone; *JHAMT, Juvenile Hormone Acid Methyltransferase; vg, vitellogenin; ilp1, insulin-like peptide 1; ilp2, insulin-like peptide 2; HSP70, heat shock protein 70; TOR1, target of*

rapamycin 1; RpL32, Ribosomal protein L32.

Click here to access/download;Figure;Fig 1 ABC.TIF

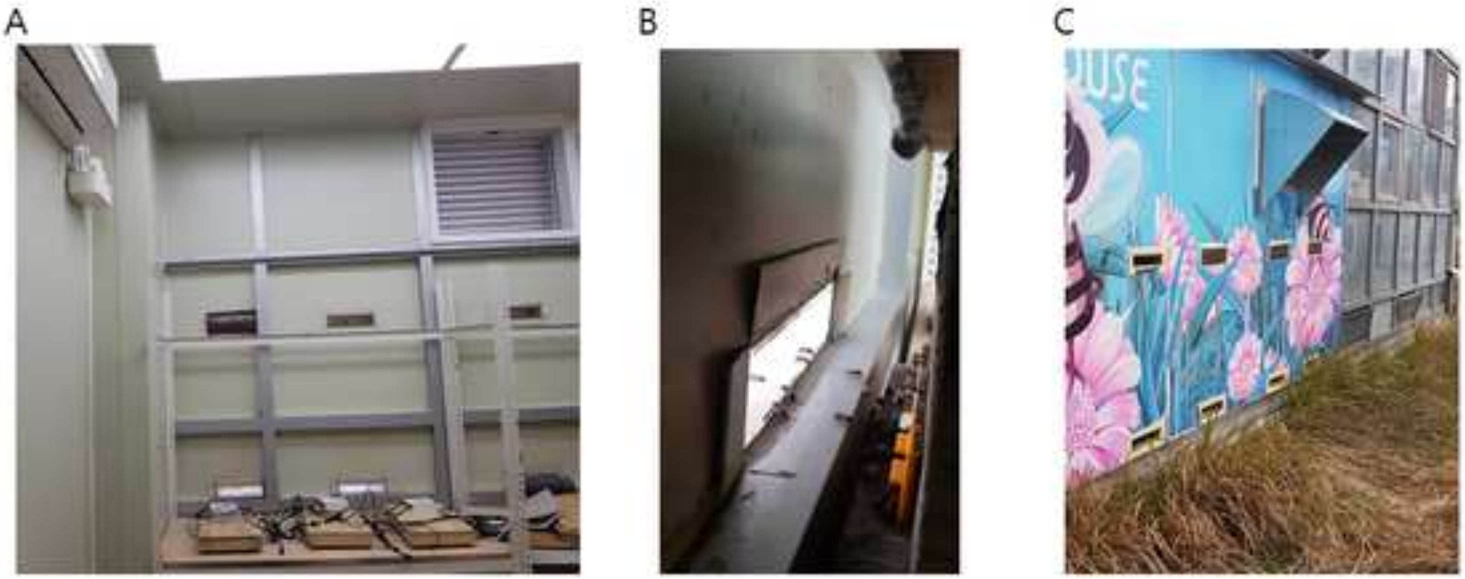

Click here to access/download;Figure;Fig 1 D.TIF

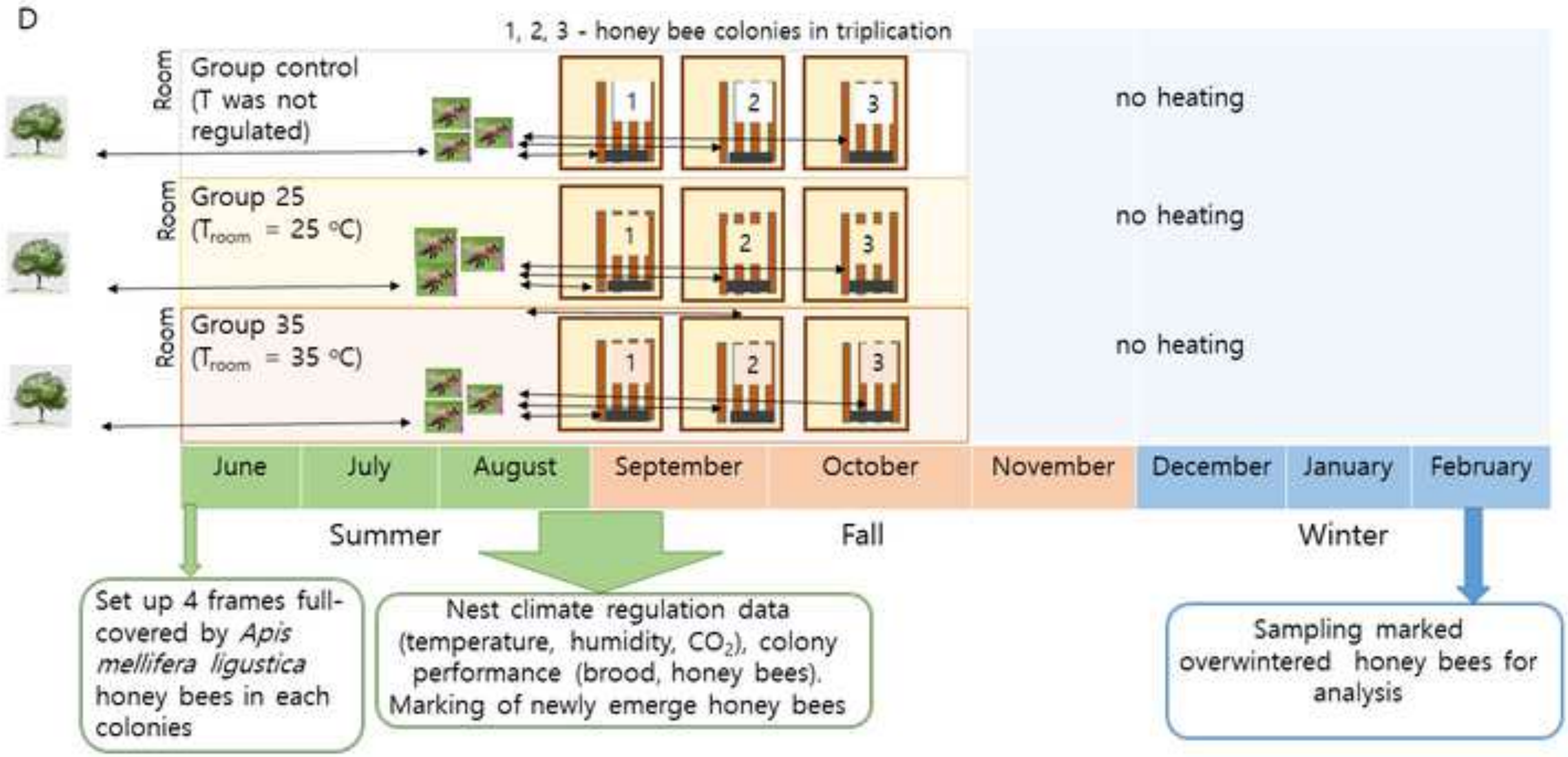

Click here to access/download;Figure;Fig 3 DEF.TIF

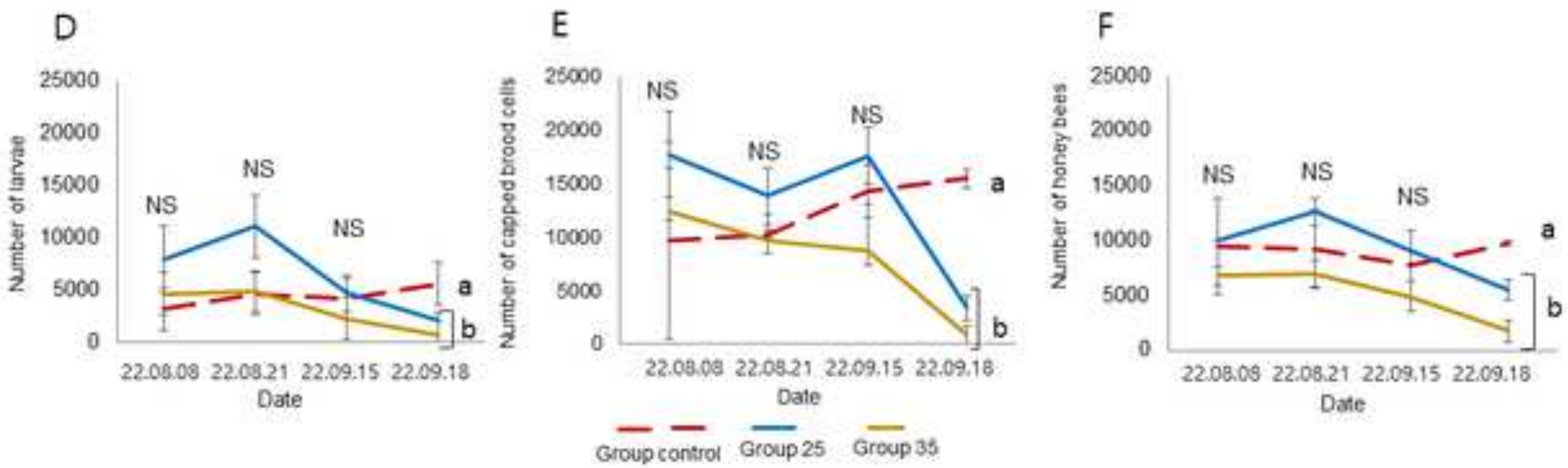

Click here to access/download;Figure;Fig 4.TIF

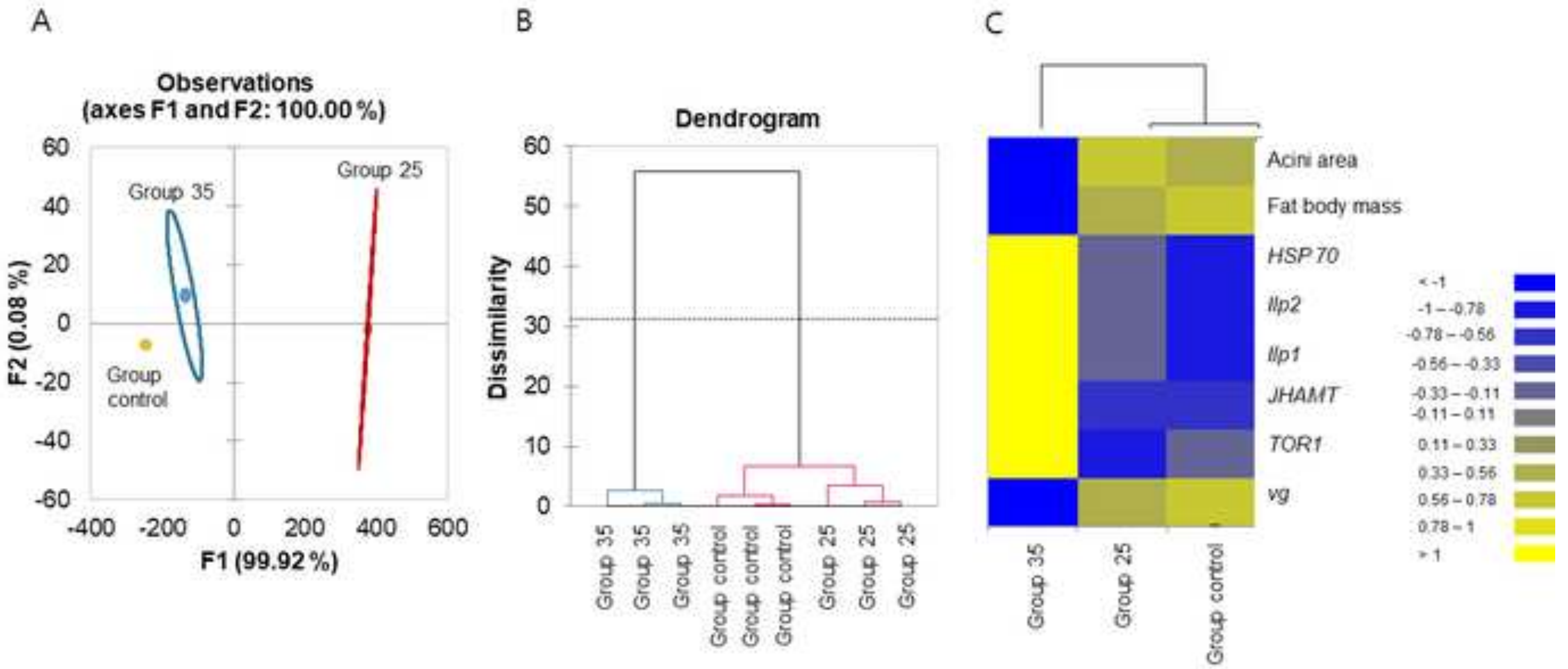

Click here to access/download;Figure;Fig 7 ABC.TIF

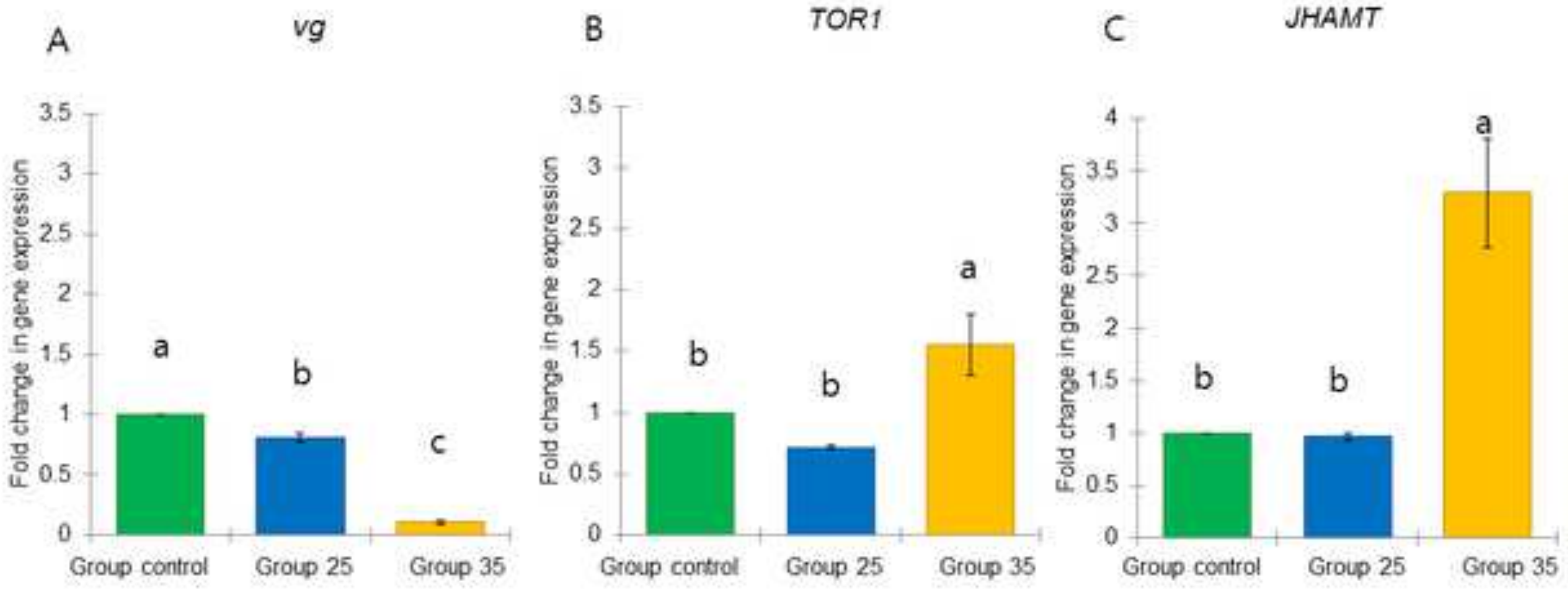

Click here to access/download;Figure;Fig 7 DEF.TIF

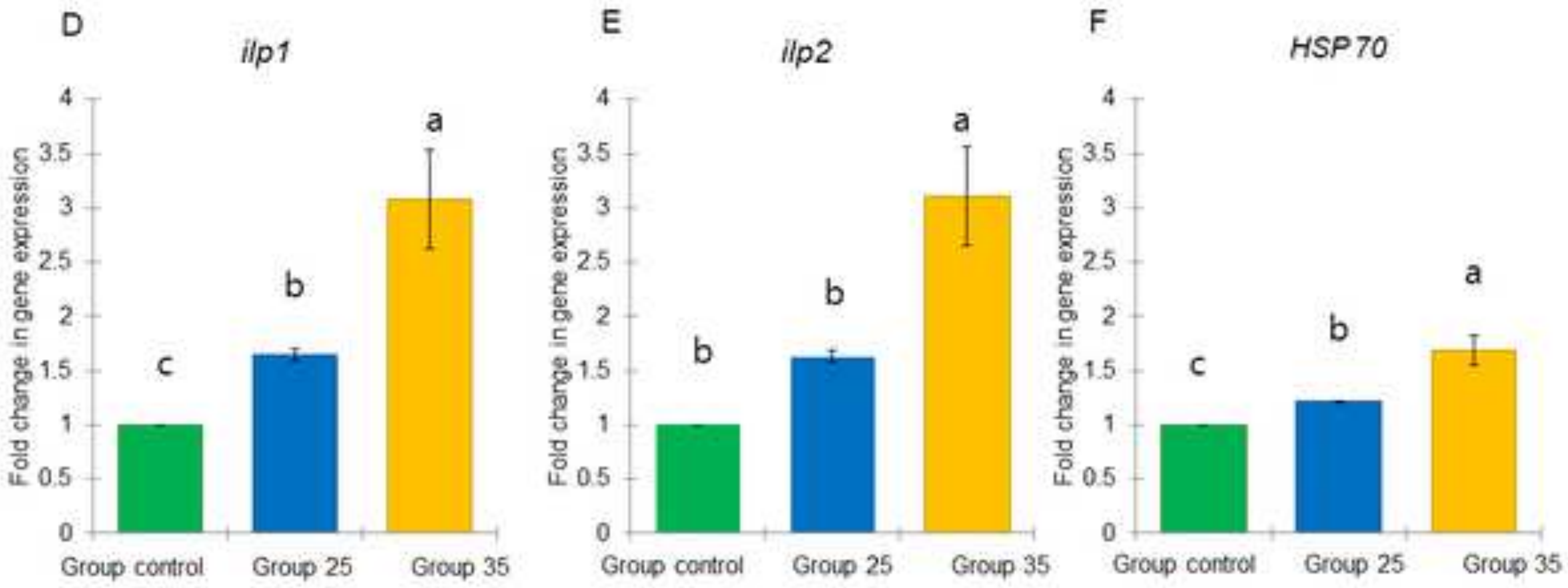

Click here to access/download;Figure;S1 Fig.TIF

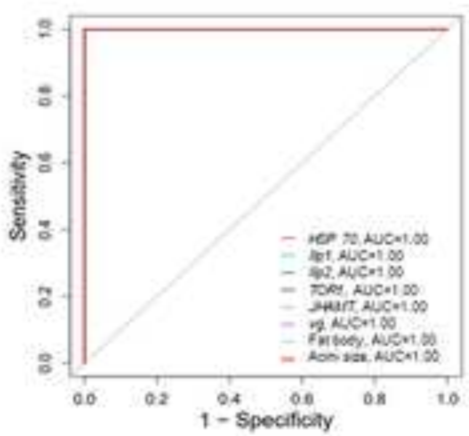

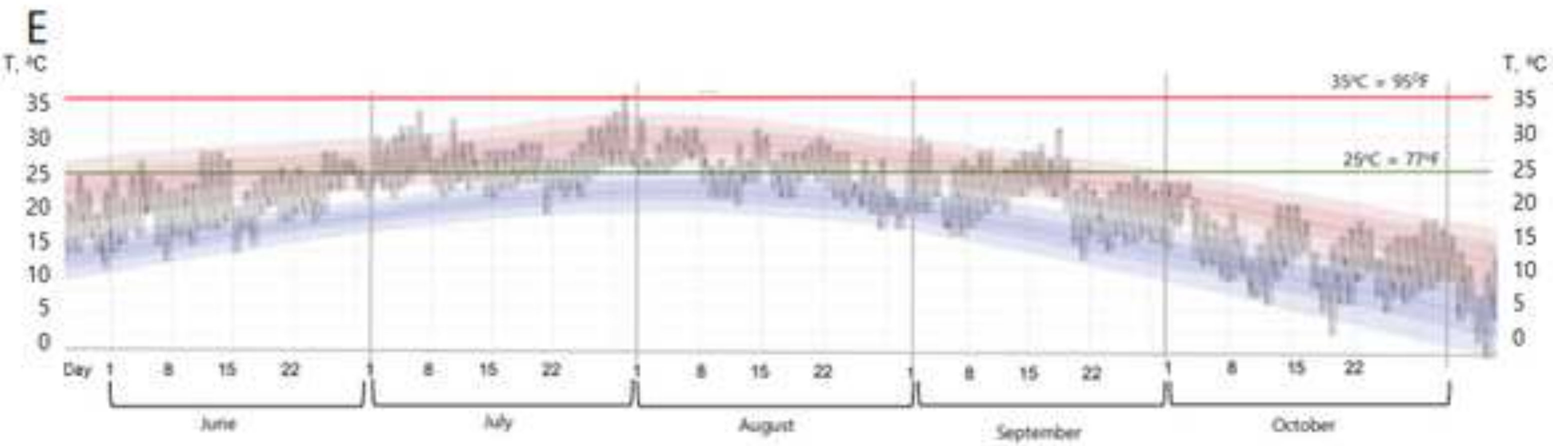

The daily range of reported temperatures (gray bars) and 24-hour highs (red ticks) and lows (blue ticks), placed over the daily average high (faint red line) and low (faint blue line) temperature, with 25th to 75th and 10th to 90th percentile bands.

Figure Click here to access/download;Figure;Fig 2 cor.TIF

Apis mellifera ligustica honey bees

Month of sampling		Group control (3 colonies)	Group 25 (3 colonies)	Group 35 (3 colonies)
Experimental condition (summer, September, and October)			T_{room} = 25 °C	T_{room} = 35 °C
**August September**	Data set 1 (brood microclimate and colony development)		Nest climate regulation	
	Statistics of data set 1		T-test ($p < 0.05$)	
**February**	Sampling of honey bees, n	(for physiology markers): 30+30+30<br>(for molecular markers): 10+10+10	30+30+30<br>10+10+10	30+30+30<br>10+10+10
	Data set 2	Two physiology markers (hypopharyngeal glands (HG, acinus size) and lipid content of fat body)<br>Molecular marker genes related to nutrition (*vg*, *ilp1*, *ilp2*, *TOR1*), development (*JHAMT*), and stress response (*HSP70*).		
	Statistics of data set 2	1. Discriminant analysis (define the variables with high discrimination score);<br>2. Agglomerative Hierarchical Clustering (check the natural groupings within dataset);<br>3. Elastic Net Regression (models the relationship between ambient temperatures, HG (acinus size) and lipid content of fat body with molecular markers);<br>4. ANOVA, Duncan's post-hoc test (comparison of significance within each variable, $p < 0.05$).		

 Click here to access/download;Figure;Fig 3 ABC cor.TIF

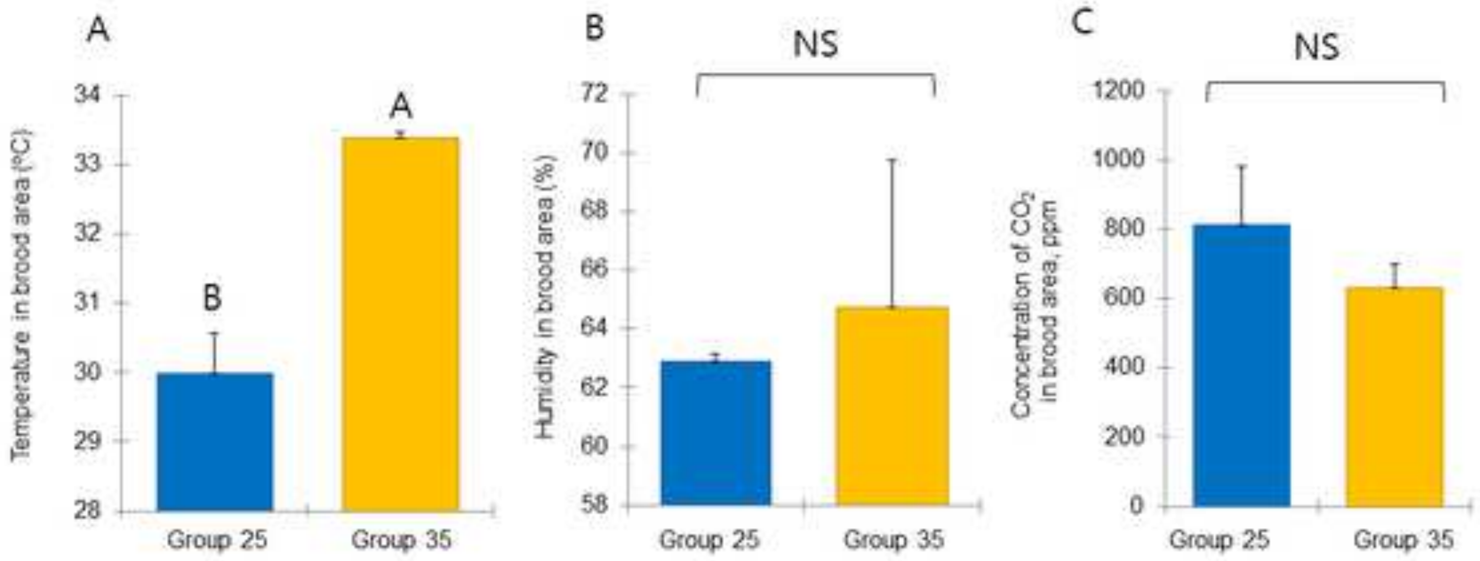

Click here to access/download;Figure;Fig 5 ABC cor.TIF

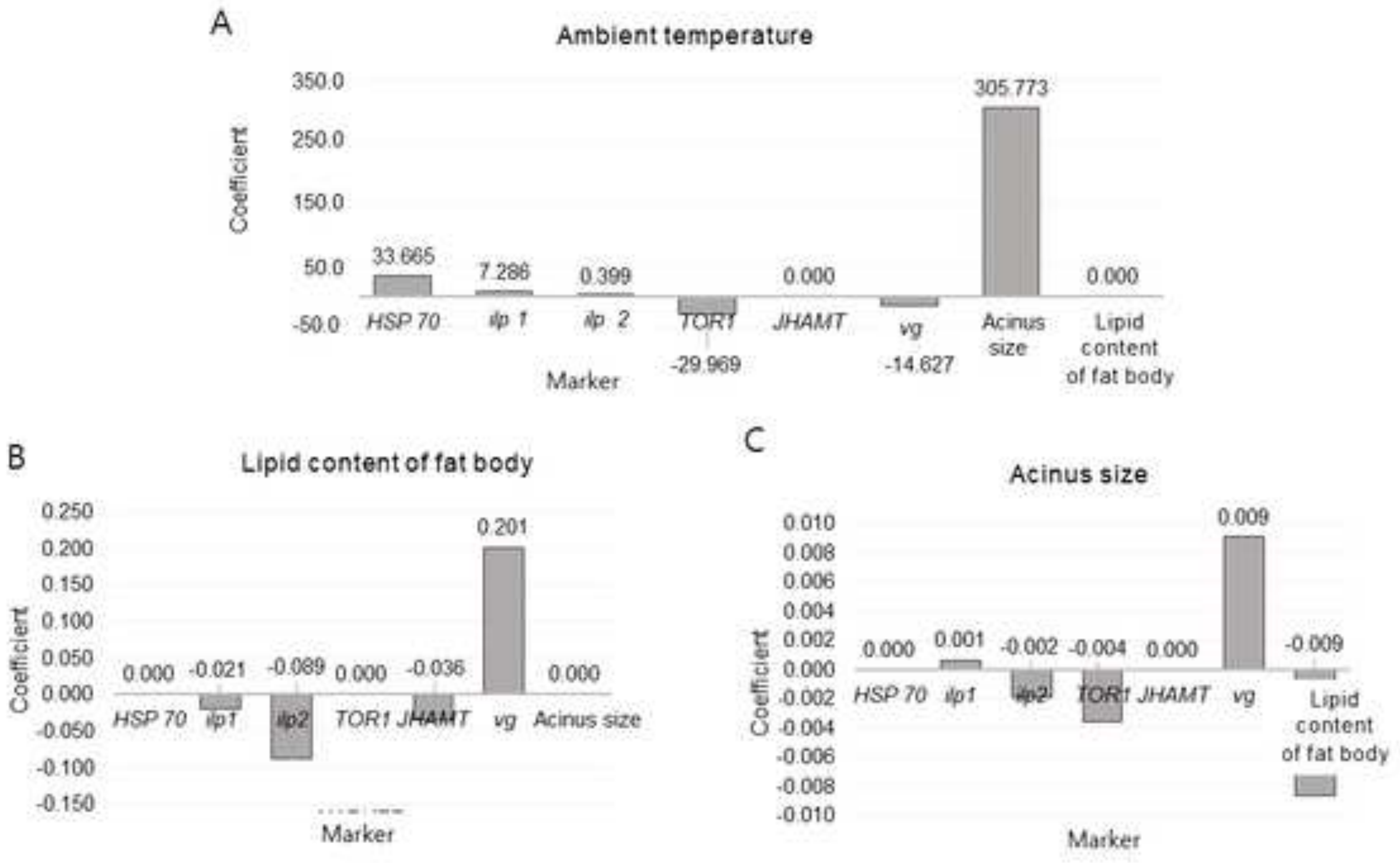

Click here to access/download;Figure;Fig 6 AB cor.TIF

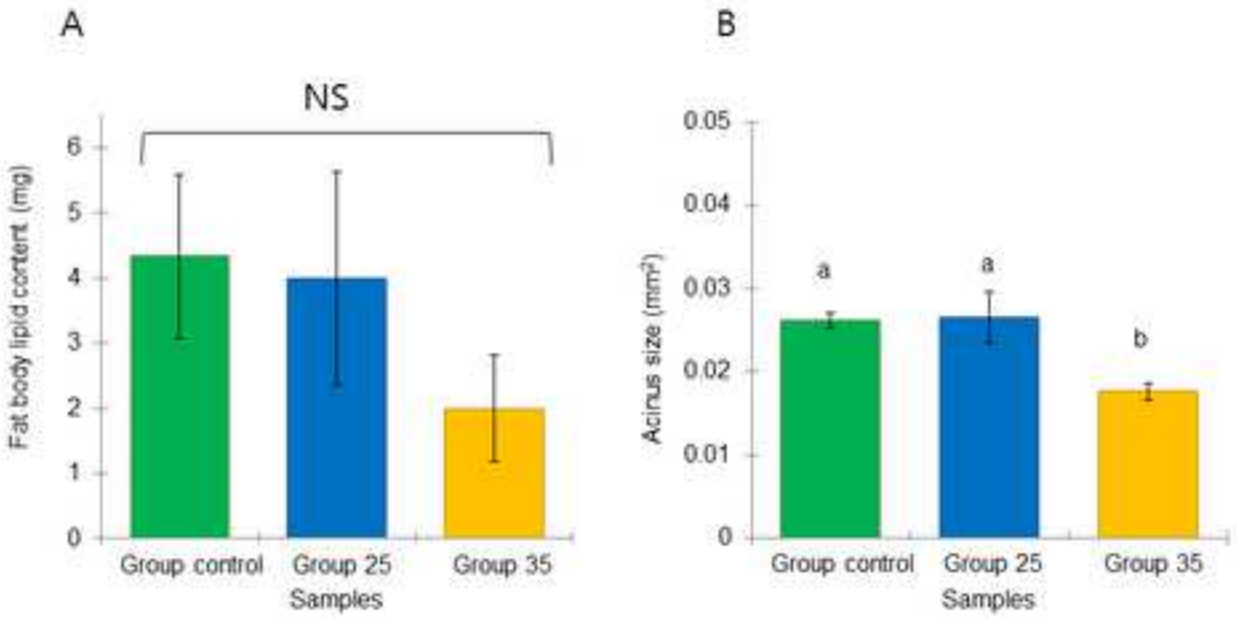

Click here to access/download
Supporting Information - Compressed/ZIP File Archive
Supporting information.zip

Click here to access/download
Supporting Information - Compressed/ZIP File Archive
Supporting information S5 S6.zip

Revised Manuscript with Track Changes

Research article

The effect of seasonal temperatures on the ~~aging~~physiology of the overwintered honey bee

Olga Frunze[1¶], Yumi Yun[1¶], Hyunjee Kim[1], Ravil R. Garafutdinov[2], Young-Eun Na[3]*, and Hyung-Wook Kwon[1]*

[1]Department of Life Sciences & Convergence Research Center for Insect Vectors (CRCIV), Incheon National University R&D Complex, 265~~119~~ Harmony-ro~~Academy-ro~~, Yeonsu-gu, Incheon, Republic of Korea 2201~~2~~4;

[2]Institute of Biochemistry and Genetics, Ufa Federal Research Center, Russian Academy of Sciences, 450054, prosp. Oktyabrya, 71, Ufa, Bashkortostan, Russian Federation

[3]Agro-materials Industry Division, Rural Development Administration (RDA), Wanju 55365, Republic of Korea

[¶]These authors contributed equally to this work

*Correspondence: hwkwon@inu.ac.kr (H.-W.K.) youngman99@korea.kr (Y.-E.N.)

Short title: **Physiology~~Aging~~ of honey bees at the end of winter**

Abstract

Honey bee physiology follows an annual cycle, with winter bees living ten times longer than summer bees. Their transition can be disrupted by climate change. Several climate factors, mainly temperature, may contribute to the global losses of winter bees. We simulated ~~climate~~global warming by maintaining constant temperatures of 25°C (Group 25) and 35°C (Group 35) in rooms around hives from June to October, while a Group control experienced natural conditions. Colony performance was assessed in August and September. In February, workers were examined for physiological traits (acin~~i~~us size~~ area~~ and lipid content in the fat body~~ mass~~) and molecular markers (*vg* and *JHAMT*), along with potential~~expected~~ markers (*ilp1, ilp2, TOR1*, and *HSP70*). Our findings suggest that temperature decreases around winter worker broods from Group 25 in the fall led to their different~~slower~~ physiological states~~aging~~ related to aging in winter compared to Group 35 workers. Changes in bees from Group 35 ~~, supported by markers. Furthermore,~~ the end of diapause~~, when aging speeds up,~~ were~~as~~ detected~~coupled~~ with an upregulation of *HSP70, ilp2,* and *TOR1* genes. ~~This research can predict t~~These signs of~~e~~ winter ~~status of~~ bees in response to summer global~~fall~~ ~~climate~~ warming ~~–~~could lead to the development of strategies ~~and help beekeepers implement strategies~~ to prevent bee losses and ~~. Additionally, it could~~improve ~~enhance~~ the ~~molecular aging~~ identification of physiological states in

insect models~~, advancing research on climate warming effects~~.

Introduction

Honey bees (below bees) play a crucial role in human life as pollinators on agricultural farms, producers of honey, royal jelly, and other products, which are valued for their health and medicinal benefits, and contributors to ecosystems worldwide. However, the loss of bees has been reported over the last two decades, particularly at the end of the overwintering period [1-3].

Several factors arising during the year can contribute to this [4]. For example, climate warming or cooling [5] impacts the lifespan in flies [6], fish [7], ~~honey~~ bees [8], and rodents [9]. Other factors related to climate change include the quality of forage [10, 11], beekeeper management practices [12, 13], and pesticide exposure from land use [11]. Although we can try to avoid some of these factors, no effective methods to mitigate the impacts of climate change and prevent ~~honey~~ bee winter mortality have been reported~~are currently lacking~~.

To advance in this direction, it is necessary to clarify the general processes occurring in late summer and fall that are involved in the transition from short-lived to

long-lived workers [14, 15], as these processes may be disrupted by climate change. This transition has been associated with external (environmental) factors that influence internal (metabolic) processes.

Known external trigger factors include a decrease in pollen availability [16, 17] and cessation of brood rearing [15, 18]. Ambient temperature, as factor of climate change, can significantly alter brood temperature in summer [19], despite ~~honey~~ bees` efforts to maintain a consistent brood microclimate [20-22].

With the onset of fall, external trigger factors initiate the transition of bees through internal processes, leading to the development of winter workers with prolonged longevity, which can last up to 8 months [1]. Briefly, this transition exhibits the classic insect diapause phenotype, characterized by reduced metabolic activity and altered hormonal profiles [14, 23-26]. Unlike other insects, the reduced metabolic activity of ~~honey~~ bees during diapause [17] includes vibrating their wing muscles to generate heat and maintain warmth within the winter cluster. This process depends on a constant energy source and requires a pure carbohydrate diet [27], which is not necessary for other diapausing insects ~~and impacts internal processes~~.

In winter bees, the physiology of internal processes related to this hormone-regulated phenomenon is well-described. It manifests as hypertrophied secretory vesicles and increased acini diameter in the hypopharyngeal gland (HG) compared to newly emerged and forager summer bees [28]. HG development is known to be positively correlated with vitellogenin (*vg*) throughout a worker's lifespan. However,

vg is synthesized in the fat body [29], which is more developed in winter bees compared to summer bees [27]. The fat body mass results from the accumulation of nutrients [30], w~~h~~ith~~ch~~ lipids constituting over >90 % of the mass in the form of triglycerides. These triglycerides are synthesized from dietary carbohydrates and fatty acids and are used not only for *vg* synthesis [30] but also for producing pheromones, phospholipids, and wax [31].

Significant differences between ~~honey~~ bees and other insects have been observed in the mutually inhibitory interaction between vg (protein) and the juvenile hormone (JH) [32]. An increased level of vg (protein and gene), alongside decreased levels of the JH [1, 15, 33], has been noted, however, the molecular mechanism of this complex relationship remains unclear [34]. It is known that the JH biosynthetic pathway begins in the glands in the brain complex and responds to sensory inputs that underpin the division of labor [34, 35]. Furthermore, at the end of winter, diapause bees gradually transition to nursing brood care and foraging physiology [1]. The last transition is characterized by several physiological and molecular changes, including a decrease in acin~~i~~us ~~area~~ size [36] and fat body mass [37], as well as reductions in vg levels and increases in JH levels [15, 38]. These changes contribute to seasonal effects and behavior [39], leading to reduced longevity and increased aging compared to diapause conditions [40, 41].

Alongside with the well-known age-related marker gene *vg* [32, 42-44], this study examined other potential markers, including the genes *JHAMT, ilp1, ilp2, TOR1*, and

HSP70. The enzyme involved in JH biosynthesis, *Juvenile Hormone Acid Methyltransferase (JHAMT),* exhibits changes in expression similar to JH titer and can serve as an alternative marker to JH [45-47]. A positive correlation with JH was observed in the *insulin-like peptide, ilp1,* whose expression increases in the brains of foragers and decreases with continued foraging [48]. The *insulin-like peptide, ilp2*, gene may~~is expected to~~ have task-related expression as it is highly expressed in nurse tissue and plays a role in increasing cell size and number in individual organs [49]. Additionally, the *TOR1* gene, a nutrient-sensing kinase, along with the *ilp1* and *ilp2* genes, participates in the mobilization of nutritional resources (autophagy) from tissues such as the fat body and glands in *Drosophila* [50]. Moreover, the *TOR* pathway is implicated in disease and nutrition in insects [51-53], and mammals [54], and it also regulates longevity extension in social insects [25]. Lastly, the stress marker *heat shock protein 70 (HSP70)* was chosen because it is detected at low levels in nurses but shows increased expression in foragers [55]. While all these markers are expected to be important for identifying the ~~aging~~physiological state in overwintered honey bees, testing them together in the same experiment requires evaluating their responses to the same triggers.~~further investigation~~ ~~.~~

This research aimed to simulate the influence of ~~climate~~global warming on colony performance in ~~early fall~~previouse seasons and to compare the physiological state~~aging~~ of overwintered *Apis mellifera ligustica* ~~honey~~ bees. The experiment

involved maintaining colonies at ambient constant temperatures of 25°C (Group 25) and 35°C (Group 35) in controlled rooms, representing the average and high summer temperatures in Incheon (Republic of Korea), respectively. A Group control was kept under natural conditions. The temperature experiments continued throughout the ~~last~~ summer, September, and October, with the colonies overwintering under the same conditions. Colony performance, including the brood microclimate, was assessed in August and September, during the transition from summer to winter physiology in the Republic of Korea. In February, we compared ~~evaluated~~ the long-term effects of ambient temperature on the physiological and molecular state ~~aging~~ of the winter bees at the end of diapause (broodless period) using eight markers~~: two physiological markers and six molecular gene markers~~. The two physiological markers were acin~~i~~us size ~~area~~ and ~~mass of~~ lipid content in the fat body. The six molecular markers were for: tasks (*vg* and *JHAMT*), nutrition (*ilp1, ilp2*, and *TOR1*), and stress response (*HSP70*). ~~By linking climate change factors, such as fall temperature, to honey bee responses in winter, this research can inform conservation actions for beekeepers to prevent bee losses.~~ We believe this study can lead to further research ~~and the development of a test kit~~ to identify the environmental causes and prevent ~~of~~honey bees –losses~~aging~~ early.

Methods

Experimental bees

Formatted: Font: 16 pt

Western ~~honey~~ bees (*Apis mellifera ligustica*) were obtained from nine colonies kept at the Incheon National University apiary in the Republic of Korea **(Fig 1A, B, C, and D),** managed by a professional beekeeper following standard beekeeping practices [56]. The experimental colonies contained~~housed~~ first-year young sister queens, naturally mated which started to lay eggs~~and beginning egg-laying~~ in May 2022. Prior to the experiment in June, colonies were standardized in terms of the number of frames (total 4), brood population (3 frames), honey amount (1 frame), and the number of ~~honey~~ bees (around 3000~3500 bees). Colony strength was assessed through visual inspection of adult population and brood area ~~populations~~ both before and during the experiment [56]. Two rooms equipped with a climate-control system were established to maintain ~~honey~~ bee colonies at 25°C (Group 25) and 35°C (Group 35), simulating ~~climate~~global warming without day and night temperature differences.

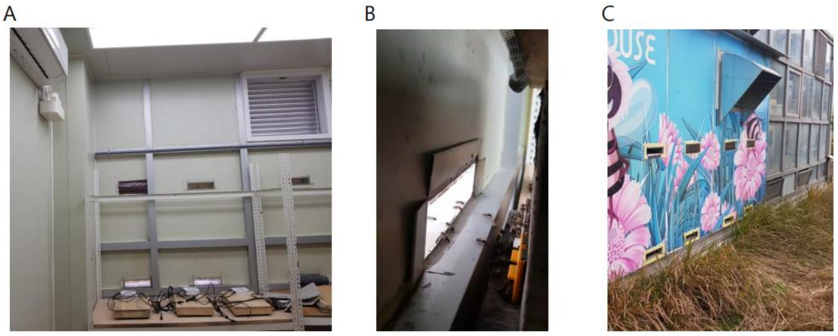

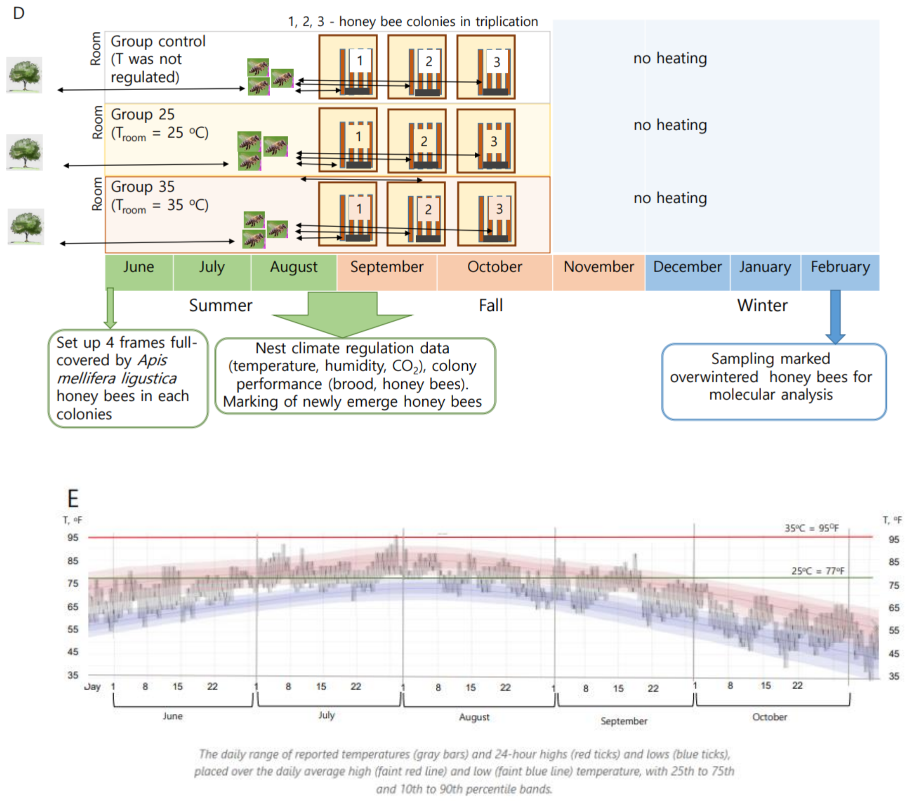

Fig 1. Overview of the experimental design (A) The room environment before the experiment. **(B)** The process of honey bees freely flying outside from the experimental room. **(C)** The view of the room and the entrance for honey bees outside. **(D)** Timeline of the experiment. **(E)** Ambient temperature of the Group control, Group 25 (green line), and Group 35 (red line) under the temperature experiment in 2022, Incheon, Republic of Korea. The data were obtained from https://weatherspark.com/h/m/142040/2022/10/Historical-Weather-in-October-2022-in-Incheon-South-Korea#Figures-Temperature.

These temperatures were selected to mimic the effects of globalclimate warming, with 25°C representing the summer average and 35°C being the highest recorded in

Incheon city under typical conditions. However, the highest temperature was not naturally reached during the summer of 2022 **(Fig 1E).** Other environmental conditions were not controlled. The ceiling light was used during colony inspections. In the third room~~,~~ housing ~~honey~~ bee colonies, the control group was provided with free ventilation without additional heating. All ~~honey~~ bees had equal opportunities to collect pollen and nectar around the apiary.

On September 18th and 25th, 2022, two combs with sealed brood were placed in an incubator set at 34°C. Upon hatching, around 200~~the~~ workers were individually marked with colored paint on their thoraxes and then reintroduced into their original colony. Throughout ~~the previous~~ summer, as well as in September and October of 2022, the honey bee colonies were subjected to the according~~varying room~~ ambient temperatures. Starting from November 2022, all colonies ~~Afterward, the controlled temperatures in the rooms~~ were kept ~~turned off, and the bee colonies were overwintered successfully **(S1 Table)** in the same rooms~~ under natural conditions and successfully overwintered. The success of colonies overwintering was monitored on February 5th and 27th, when we recorded whether the bees survived (+) or did not survive (-) **(S1 Table)**.

The overwintered marked ~~honey~~ bees, aged approximately 4.5 months, were sampled on February 5th, 2023, a time when queens had not yet begun laying eggs ~~and the overwintered bees were expected to have successfully survived the winter~~. Forty ~~honey~~ bees from each colony were sampled and individually placed in labeled

50 ml tubes, then stored at -80°C before analysis. The success of colonies overwintering was monitored on February 5th and 27th, when we recorded whether the honey bees survived (+) or did not survive (-) **(S1 Table).**

Brood microclimate measurements

Formatted: Font: 16 pt

The temperature, humidity (AM2320, BeeOnFarm, Korea), and CO_2 concentration (BO-100, BeeOnFarm, Korea) sensor systems were placed on the top and middle of each colony in August 2022. The data logging system was included in the sensor system because it provided a real-time clock. Data consisting of time (seconds), CO_2 (in ppm), temperature (in °C), and relative humidity (as a percentage) were saved, with approximately three measurements in minutes to approximately one file per day **(S1 File)**. The data from 6 honey bee colonies kept in each room (three at 25°C and three at 35°C) were collected over 15 days from 11 to 25 August. Average values for the day were used for statistical calculations.

Hypopharyngeal gland (HG) measurements

Formatted: Font: 16 pt

Acinus sizereas of ninety acini from the hypopharyngeal glands of ninety overwintered honey bees in each Group (control, 25°C, and 35°C) were measured to determine if there were differences based on the ambient temperature during the previous fall seasons. Thirty bees were collected from each colony, flash frozen in liquid nitrogen, and maintained at −80°C until their glands were measured **(S2 File)**.

For each bee, the HGs were dissected into 1× phosphate-buffered saline (PBS) with 0.1% Tween® 20 and visualized at 40× magnification. The ~~area~~size (mm^2) of at least ~~minimum of~~ ten randomly selected acini per bee was measured using a Nikon Eclipse E200 microscope (Nikon, Tokyo, Japan) equipped with Toup~~v~~View software (Touptek, Hangzhou, China) ~~software~~installed on a PC. Only acini with clearly defined borders were inclu~~s~~ded. Acinus~~i~~ size ~~areas~~ was~~ere~~ averaged within each individual~~s~~.

Formatted: Font: 16 pt

Fat body lipid content

The fat body lipid content in the abdomen of overwintered honey bees was studied in early February by the standard method [57, 58]. Ten ~~honey~~ bees per colony were collected ~~from each colony~~; the ninety ~~honey~~ bees were flash frozen in liquid nitrogen and maintained at −80°C until their abdomens were dissected and their guts were removed. Pools of ten abdomens per colony were weighed on a scale (HS220S, HANSANG Instrument Co., Ltd., Republic of Korea) before and after drying to a constant weight in a fume hood (SSFH-1000, Shinsaeng, Republic of Korea). The total lipid content was determined by measuring the decrease in weight following the removal of lipids from the dried tissue through extraction in 5 ml portions of diethyl ether (179272-1 L; Sigma–Aldrich, USA) **(S2 File).**

RNA Extraction and cDNA Synthesis

Formatted: Font: 16 pt

A total of ninety ~~honey~~ bees (ten winter bees from each colony) ~~*Apis mellifera ligustica* colonies~~ were sampled in labeled tubes and stored at -80°C. ~~Three honey bees from each colony were analyzed.~~ The brain and gut-free abdomen of five sampled ~~honey~~ bees from each colony (fifteen bees for treatment) were dissected on ice, and RNA was extracted directly. RNA was extracted from the brain for the analysis of *HSP70, JHAMT, ilp1, ilp2*, and *TOR1* expression, and from the abdomen for *vg* gene expression analysis, as this gene is expressed in the fat body located in the abdomen. Genes are measured in the brain to understand their roles in stress responses (*HSP70*), metabolic regulation (*JHAMT* and *TOR*), and neural function (*ilp1* and *ilp2*).

Total tissue RNA (brain or abdomen) was extracted ~~from fifteen randomly selected bees in each group~~ using a Qiagen RNeasy Mini Kit (#74,104; Qiagen, Valencia, CA, USA). ~~The tissues in each replicate were brain tissue for *TOR1, ilp1, ilp2, JHAMT*, and *HSP70* and abdomen for *vg* gene expression.~~ The total RNA concentration and purity were quantified using OD260/OD280 values between 1.8 and 2.0. Next, reverse transcription was performed using an RNA to cDNA EcoDryTM Premix (Oligo dT) kit (Takara, Japan). The reverse transcription reaction mixture included 50 ng/µl total RNA (with the clear volume calculated for each sample) and RNase-free water for a total volume of 20 µl. Reverse transcription was conducted at 42°C for 60 min, followed by heating at 70°C for 10 min.

Formatted: Indent: First line: 0 ch

Quantitative real-time PCR

Formatted: Font: 16 pt

The relative expression of six genes ~~related to nutrition~~ (*vg, ilp1, ilp2, TOR1*, ~~),~~ ~~development (~~*JHAMT*, and~~), and stress response (~~ *HSP70)* was measured. The housekeeping gene *RpL32* (Ribosomal protein L32*)* was used as an endogenous control. The PCR primer sequences are shown in **S2 Table.**

Formatted: Font: Not Italic

The reaction conditions were optimized. The qRT–PCR mixture had a volume of 20 µl which included 2 µl of template cDNA, 10 µl of Green qPCR Master Mix, 1 µl of upstream and downstream primers (5 pM/µl), and 6 µl of nuclease-free water (AM9930, Invitrogen, USA). Quantitative real-time PCR (qRT–PCR) was conducted using Brilliant III Ultra-Fast SYBR® Green qPCR Master Mix (600882, Agilent Technologies, USA) on an AriaMx Real-Time PCR System (Agilent Technologies LDA, Malaysia) with AriaMx 96 Well Plates, Skirted, LP (401490, Agilent Technologies, USA) covered by MicroAmp™ Optical Adhesive Film (4311971, Thermo Fisher Scientific, USA).

The qRT–PCR amplification procedure was as follows: initial denaturation at 95°C for 10 minutes; 40 cycles of denaturation at 95°C for 30 seconds, annealing at 60°C for 25 seconds, and extension at 72°C for 15 seconds. Each sample was ~~technically~~ replicated three times. Data analysis was performed using Agilent AriaMx version 2.0 analysis software. Relative gene expression data were analyzed using the 2^(-Delta Delta C(T)) method [59, 60] (**S3 File**).

Confirmation of amplicons in the RT–PCR products was performed by separating them through electrophoresis in a 2% agarose gel at 80 V for 40 minutes and

analyzing them using a gel documentation system, the Gerix 1010 transilluminator (Biostep GmbH, USA) (**S2 Fig**). A Dyne 50 bp DNA Ladder (Cat. No. A701, DYNEBIO, Korea) was used as a reference.

Statistical analysis

Formatted: Font: 16 pt

The statistical analysis, designed as illustrated in **Fig 2**, were~~as~~ conducted using Microsoft Excel and XLSTAT software (Addinsoft Pearson Edition 2014, Addinsoft, Paris, France). The characterization was performed using the following strategies [61]: (a) classification of the marker dataset to find a combination of features that best separates different groups (supervised approach, Discriminant Analysis (DA)); (b) clustering to discover natural groupings in the absence of labeled information (unsupervised approach, Agglomerative Hierarchical Clustering (AHC)); and (c) modeling the relationship between independent variables and a dependent variable and dealing with multicollinearity in the marker dataset to rank the markers by importance for identifying overwintered ~~honey~~ bee task development (supervised approach, Elastic Net Regression).

Specifically, the ~~honey~~ bees were categorized into groups, and the variables with the highest discrimination scores were selected using DA because it determines the linear combination of variables and provides maximal separation between groups. To discriminate the groups, Fisher distances were applied, which emphasize the linear combinations of variables that maximize the differences between groups.

AHC analysis provided a visual representation of the clustered dataset, revealing the arrangement of individual data points or observations based on their dissimilarity. The evaluation metric for clustering algorithms was the silhouette index. The score is bound~~ed~~ between (− 1) for incorrect clustering and (+ 1) for highly dense clustering.

Apis mellifera ligustica honey bees

<table>
<tr><td>Month of sampling</td><td></td><td>Group control
(3 colonies)</td><td>Group 25
(3 colonies)</td><td>Group 35
(3 colonies)</td></tr>
<tr><td colspan="2">Experimental condition
(summer, September, and October)</td><td></td><td>T_{room} = 25 °C</td><td>T_{room} = 35 °C</td></tr>
<tr><td>August
September</td><td>Data set 1
(brood microclimate and colony development)</td><td></td><td colspan="2">Nest climate regulation</td></tr>
<tr><td></td><td>Statistics of data set 1</td><td></td><td colspan="2">T-test ($p < 0.05$)</td></tr>
<tr><td>February</td><td>Sampling of honey bees, n</td><td>(for physiology markers): 30+30+30
(for molecular markers): 10+10+10</td><td>30+30+30
10+10+10</td><td>30+30+30
10+10+10</td></tr>
<tr><td></td><td>Data set 2</td><td colspan="3">Two physiology markers (hypopharengial glands (HG, acini area) and fat body mass)
Molecular marker genes related to nutrition (vg, ilp1, ilp2, TOR1), development (JHAMT), and stress response (HSP70).</td></tr>
<tr><td></td><td>Statistics of data set 2</td><td colspan="3">1. Discriminant analysis (define the variables with high discrimination score);
2. Agglomerative Hierarchical Clustering (check the natural groupings within dataset);
3. Elastic Net Regression (model the relationship between HG (acini area) and fat body mass with molecular markers);
4. ANOVA, Duncan's post-hoc test (comparison of significance within each variable, $p < 0.05$).</td></tr>
</table>

Fig 2. Experimental conditions, sampling schedule, data sets, and statistical tests used for analysis.

Elastic Net Regression analysis was used to test three~~wo~~ models of the relationship [61, 62] between temperatures, physiology and molecular markers. The dependent variables were ambient temperatures, acin~~i~~us size and lipid content in the fat body~~ mass~~. The seven independent variables were ranked by coefficient. Receiver operating characteristic (ROC) curves were generated to quantify how accurately regression analysis can discriminate between honey bee groups [63]. The ROC specificity and sensitivity plot demonstrated a perfectly fitted model linking physiology to molecular markers (gene expression profile) (**S1 Fig**). The area under the curve (AUC) can range

Formatted: Suppress line numbers

from 0.5 to 1.0, with a preference for higher scores, meaning that the model effectively distinguishes between the two classes.

The mean, standard deviation, and variance of each gene were calculated using descriptive statistics and visualized using the heat map module. For the gene expression analysis, ANOVA was used to test overall effects, followed by the Duncan's post hoc test ($p < 0.05$) for multiple comparisons and the t-test for differences of means between two groups of ~~honey~~ bees (**S4 File**). ~~The bar plots were generated using the online SRplot Official Website (https://www.bioinformatics.com.cn/en; accessed on 30 October 2023).~~

Results

The experiment was conducted on nine colonies of western honey bees (*Apis mellifera ligustica*). Each Group (control, 25°C, and 35°C) was represented by three colonies. The bees in~~from the~~ Groups 25 and 35 experienced constant diurnal ~~stable, controlled day and night~~ ambient temperatures (modeling ~~climate~~global warming) from June to October, while the room for the Group control was freely ventilated to allow for natural ambient temperatures. All bees experienced natural foraging outside without restrictions. Newly emerged ~~honey~~ bees from each Group were marked in September for the overwintering experiment in February.

Dynamics of ambient temperature in summer and fall months

The daily temperature in the first experimental room was set at 25°C for five months, and there were no significant differences compared to the natural daily average temperature in the summer months or September (t-test (ANOVA, Duncan's post hoc tests, $p > 0.05$). The difference in experimental conditions for the honey bees from between the Group control and the Group 25 was due to diurnal day-night temperature fluctuations, which were eliminated absent in the latter Group 25, where . Although warmer days and cooler nights did not affect the bees. In our experiment, this stable elevated night temperature in Group 25 simulated a global warming scenario. significant temperature differences were not detected, a stable elevated night temperature is one of the factors of climate warming that influences bees. HHowever, the ittemperature in room Group 25 was significantly highergreater than the average daily temperature in October 2022 (t-testsame tests, $p < 0.00015$). The daily temperature in the second room was set at 35°C for five months, which was significantly greater than the average diurnaldaily temperatures during the summer months, September, and October (same testst-test, $p < 0.00015$).

Brood microclimate and colony development in August and September

In this study, wWe hypothesized that the regulation of the nest climate by colonyinside colonies performance –influences the physiology of winter bees and the success of overwintering. we evaluated tThe performance of the colony using temperature,

humidity, CO_2, number of larvae, capped brood area, and honey bees' measurements from August 08 to September 18, 2022 were evaluated, when short-lived bees commenced rearing larvae that would develop into long-lived bees for the overwintering period. Since the expected ambient temperature in August and early September and the comparison of the colony performance of the Groups control and Group 25 on August 08 (**Fig 3 D, E, F**) showed no significant differences, sensors were not set up in the Group control. We hypothesized that the regulation of the nest climate inside colonies influences the physiology of winter bees and the success of overwintering.

Formatted: Centered

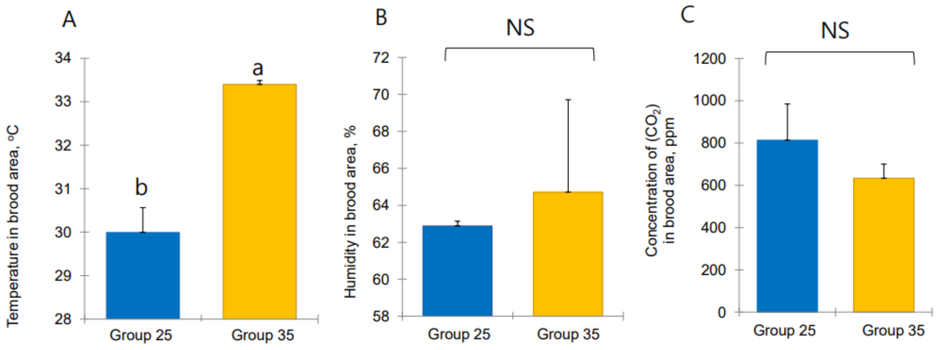

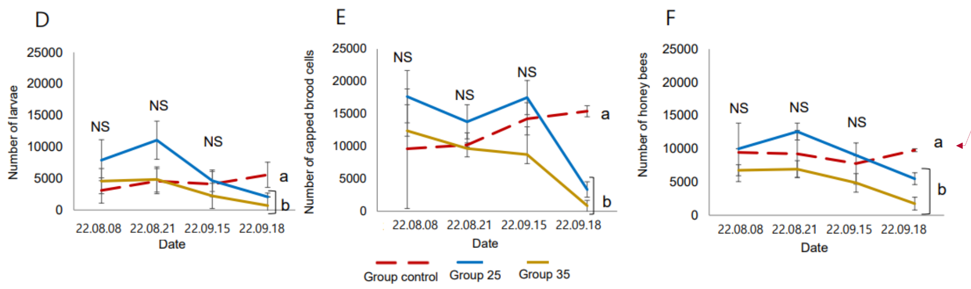

Formatted: Don't swap indents on facing pages

Fig 3. Colony performance in honey bees treated under different ambient temperatures (08 August – 18 September 2022). (A) Temperature in the brood area (°C). **(B)** Humidity in the brood area (%). **(C)** Concentration of CO_2 in the brood area (ppm). **(D)** Number of larvae. **(E)** Number of capped brood cells. **(F)** Number of ~~honey~~ bees. Statistical significance was determined using a t-test in **Fig 3A, B**, and **C** (p < 0.01 for 3A5, indicated by different letters above the columns) to compare the means of the two groups, one-way ANOVA, Duncan's post hoc tests (p < 0.05 for 3D and 3F, p < 0.01 for 3E, indicated by different letters above the columns) to compare the means of the three groups in **Fig 3D, E**, and **F**; bars in the graphs represent the mean ± SD.

Formatted: Font: Italic

SD – Standard Deviation, NS – No Significance. Honey bee colonies in Group 25 were maintained in room conditions at 25°C; colonies in Group 35 were kept in room conditions at 35°C;~~, while~~ colonies from Group control were maintained under natural conditions.

The mean and standard deviation of the brood temperature for bee colonies kept at 25°C and 35°C were 30.00 ± 0.982 and 33.40 ± 0.154 °C, respectively. Similarly, the brood ~~nest~~ humidities for these groups were 60.00 ± 0.435 and 64.72 ± 8.650 %, and the brood~~nest~~ CO_2 concentrations were 813.55 ± 296.80 and 633.30 ± 117.00

ppm, respectively. Importantly, the ~~nest~~brood temperature inside the colonies kept at 35°C was significantly greater than that inside the colonies kept at 25°C (t-test, $p <$ 0.0~~5~~1) (**Fig 3A**). However, ~~nest~~ brood humidity, CO_2 concentration, number of larvae, capped brood cells, and bees were not significantly differing **(Fig 3B, C**: t-test, $p >$ 0.05) (**Fig 3D, E, and F:** ANOVA, Duncan's post hoc tests $p > 0.05$). The control colonies~~ in the Group control~~, however, exhibited significantly (ANOVA, ~~Duncan's post hoc tests,~~ $p <$ 0.01~~5~~) larger capped brood area, more larvae, and a higher number of bees (ANOVA, $p < 0.05$) on September 18 compared to colonies in Groups 25 and 35.

Distinguishing ~~Analysis of the~~ honey bee groups through marker ranking

~~and the relationship between the markers~~

The overwintered bees~~relationship between molecular markers and the~~ were distinguished by their physiological state~~biological age of overwintered honey bees was analyzed~~ using a dataset comprising two established physiological markers (acinus size~~i~~ area and ~~mass of~~ lipid content in the fat body) and six molecular markers (*vg, TOR1, JHAMT*, *ilp1, ilp2,* and *HSP70*).

Data Mining methods, including Discriminant Analysis (DA), Agglomerative Hierarchical Clustering (AHC), and Elastic Net Regression, were employed in the analysis.

DA was performed to plot the Groups control, 25 and 35, and to identify the optimal combination of features that effectively distinguishes them. The Wilcoxon Lambda test within the DA yielded a statistically significant result (two-tailed test, $p < 0.05$), indicating that the model successfully differentiated between groups (**Fig 4A**). The observed Wilks' lambda value of 0.0~~00~~ signifies a robust separation of the Groups control, 25, and 35, demonstrating a high level of discriminative power. A comparison revealed significant differences between Group 25 and Group 35 ($F(2,8) = 19.37$; two-tailed test, $p < 0.05$; **Fig 4A**).

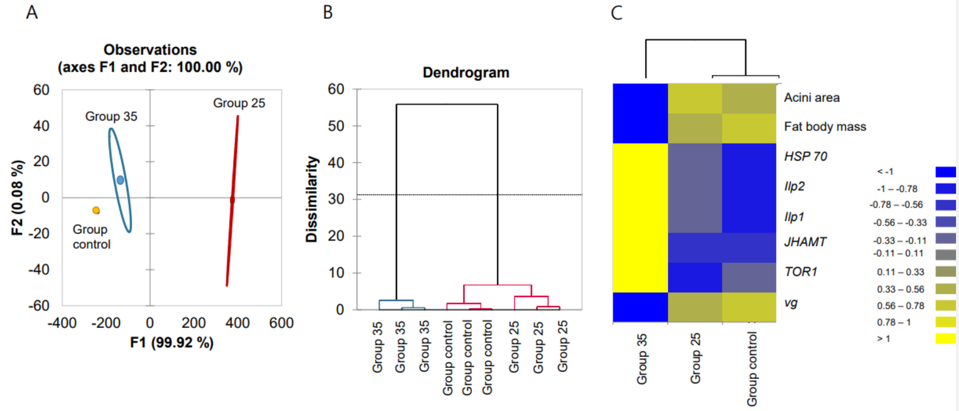

Fig 4. The discrimination of overwintered honey bees was performed using eight variables related to aging: two physiological variables (acinus size~~i area~~, n = 30; ~~mass of~~ lipid content in the fat body, n = 10) and six molecular variables (n = 10), including the expression levels of *HSP70, ilp1, ilp2, JHAMT, TOR1,* and *vg* genes. (A) Discriminant Analysis (DA). **(B)** Agglomerative Hierarchical Clustering (AHC). **(C)** Heatmap with cluster analysis based on ddCt values, acin~~i~~us size ~~area~~ (mm²), and the lipid content in the fat body ~~fat body mass~~ (mg).

Formatted: Indent: First line: 1 ch, Don't add space between paragraphs of the same style

Formatted: Font: Not Bold

The Fisher distances (FDs) in the DA between the Groups control, 25, and 35 were significantly different (FD = 27511~~.10~~; $p < 0.05$ and FD = 19131~~.16~~; $p < 0.05$, respectively). Moreover, the FD density was also significantly different between the Group control and Group 25 (FD = 784~~3.659~~; $p < 0.05$). The ~~honey~~ bees were separated along the primary discriminant axis F1 in 99.92% accuracy, where discriminant function coefficients for gene expressions *vg, HSP70*, and *JHAMT* were -20.09~~2~~, -14.96~~0~~, and 23.58~~76~~, respectively. These coefficients were the highest, providing significant role~~contributoins~~ to this separation.

Subsequently, AHC was employed to identify natural groupings without labeled information using a dissimilarity measure. The evaluation metric for clustering algorithms, the Silhouette index between clusters 1 and 2, was 0.657, indicating successful clustering, albeit with moderate density **(Fig 4B, C).**

Formatted: Suppress line numbers

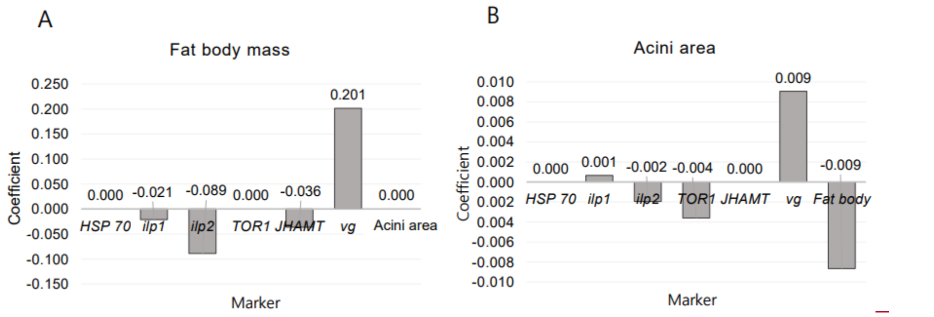

~~**Fig 5. Elastic Net Regression modeling the relationship beween dependent variables fat body mass (mass of lipid content in the fat body, A) and acini area (B) and the independent variables**~~

Formatted: Don't add space between paragraphs of the same style, Line spacing: Double, Don't suppress line numbers

~~**(*HSP70, ilp1, ilp2, TOR1, JHAMT, vg,* and acini area or fat body mass respectively) to rank the variables by importance for predicting dependent variables.**~~

Finally, Elastic Net Regression was used to rank the markers by importance by modeling the relationships between eight **(Fig 5A)** or seven **(Fig 5 B, C)** independent variables and one dependent variable ~~**(Fig 5A, B).**~~. This analysis was conducted separately for ambient temperature **(Fig 5A)**, fat body mass **(Fig 5~~A~~B)** and acinus size~~i area~~ **(Fig 5C~~B~~)**. The ambient temperature model ~~The fat body mass model~~ identified significant predictors, with variable acinus size ~~vg~~ having the highest positive coefficient (305.773~~0.201~~) and variable *TOR1*~~*ilp2*~~ and *vg* having the highest negative coefficients (-29~~0~~.969~~089~~) and (-14.627) respectively. In the acinus size~~i area~~ model, significant predictors were identified, with the variable *vg* having the highest positive coefficient (0.009) and the variable fat body mass having the highest negative coefficient (-0.009). In this all ~~both~~ models~~, only two genes~~ *vg* ~~and *ilp2*~~ showed high ~~positive and negative~~ coefficient values~~, respectively~~.

Fig 5. Elastic Net Regression modeling the relationship between dependent variables ambient temperatures (settings in summer and fall) (A), lipid content in the fat body (in February) (B), and acinus size (in February) (C) and the independent variables (*HSP70, ilp1, ilp2, TOR1, JHAMT, vg*, and acinus size or lipid content of fat body where appropriate, in February) to rank the variables by importance for predicting dependent variables.

~~Comparative analysis of the~~ Physiological markers ~~biological age~~ of

Formatted: Normal, Indent: First line: 0 ch, Don't add space between paragraphs of the same style

Formatted: Font: Bold

Formatted: Font: Bold

Formatted: Font: Bold

Formatted: Font: Not Bold

Formatted: Font: Italic

Formatted: Font: Italic

Formatted: Normal, Indent: First line: 0 ch, Don't add space between paragraphs of the same style, Suppress line numbers

~~**overwintered western**~~ **honey bees** ~~**through physiology markers**~~

These results are ~~the results~~ based on the two physiological markers, analysis of ~~the mass of~~ lipid content in the fat body ~~(fat body mass)~~ and the acinus size~~i area~~ of~~from~~ the hypopharyngeal glands (HGs) in overwintered bees. The mean and standard deviation of the lipid content ~~fat body mass~~ for the bees in the Groups control, 25, and 35 were 4.33 ± 1.25~~47~~, 4.00 ± 1.63~~3~~, and 2.00 ± 0.82~~16~~ mg/bee, respectively, with no significant differences (ANOVA, Duncan's post hoc, P = 0.22~~2~~, p ~~<~~> 0.05), even though the lipid content ~~fat body mass of the them~~ in Group 35 was slightly lower **(Fig 6A).**

Formatted: Font: Times New Roman

Similarly, the mean and standard deviation of the ~~area~~size of the bee acin~~i~~us from the Groups control, 25, and 35 were 0.026 ± 0.001, 0.027 ± 0.003, and 0.018 ± 0.001 mm², respectively. Notably, the acini~~i area~~ in bees from Group 35 were~~as~~ significantly smaller~~lower~~ (ANOVA~~same test~~, P = 0.006, p < 0.0~~5~~1) than in bees of the ~~that in the~~ Group control and 25 **(Fig 6B).** Importantly, both the lipid content~~fat body mass~~ and the acinus size~~i area~~ of the overwintered bees in Group 35 were lower than those in Groups control and 25 **(Fig 6)**, suggesting lower nutrition and different~~accelerated~~ physiological state~~aging~~ in bees from Group 35.

Formatted: Suppress line numbers

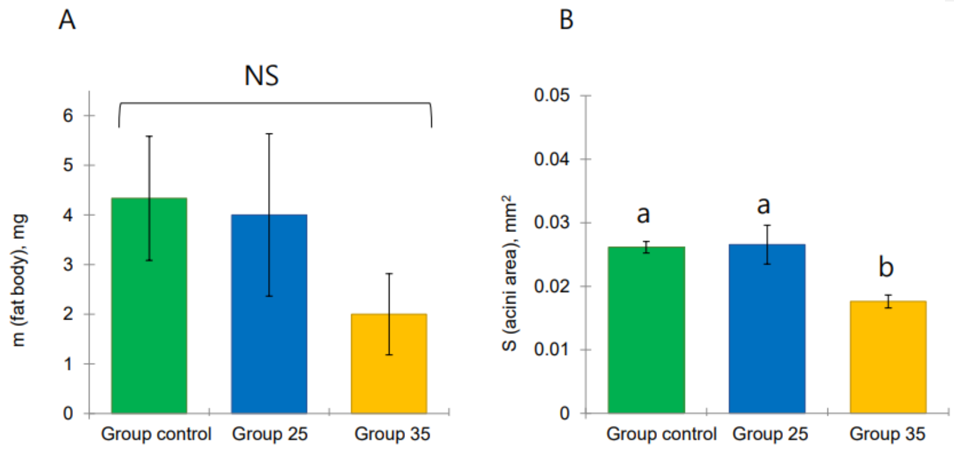

Fig 6. Physiological markers of long-lived honey bees at the end of winter (February) (A) The average of ~~fat body mass (mass of~~ lipid content in the fat body~~)~~, n = 10 (mean ± SD). **(B)** Average acinus size~~i area~~, n = 30 (mean ± SD). Statistical significance was determined using one-way ANOVA, Duncan's post hoc tests ($p < 0.05$, indicated by different letters above the columns).

Formatted: Indent: First line: 1 ch, Don't add space between paragraphs of the same style

Molecular markers ~~Comparative analysis of the biological age~~ of ~~overwintered western~~ honey bees ~~through molecular markers~~

These results ~~are the results based on molecular markers. They~~ include two previously defined task-related markers, *vg* and *JHAMT,* as well as four genes ~~expe~~predicted to respond to ~~aging~~physiology: *TOR1, ilp1, ilp2*, and *HSP70* **(Fig. 7).**

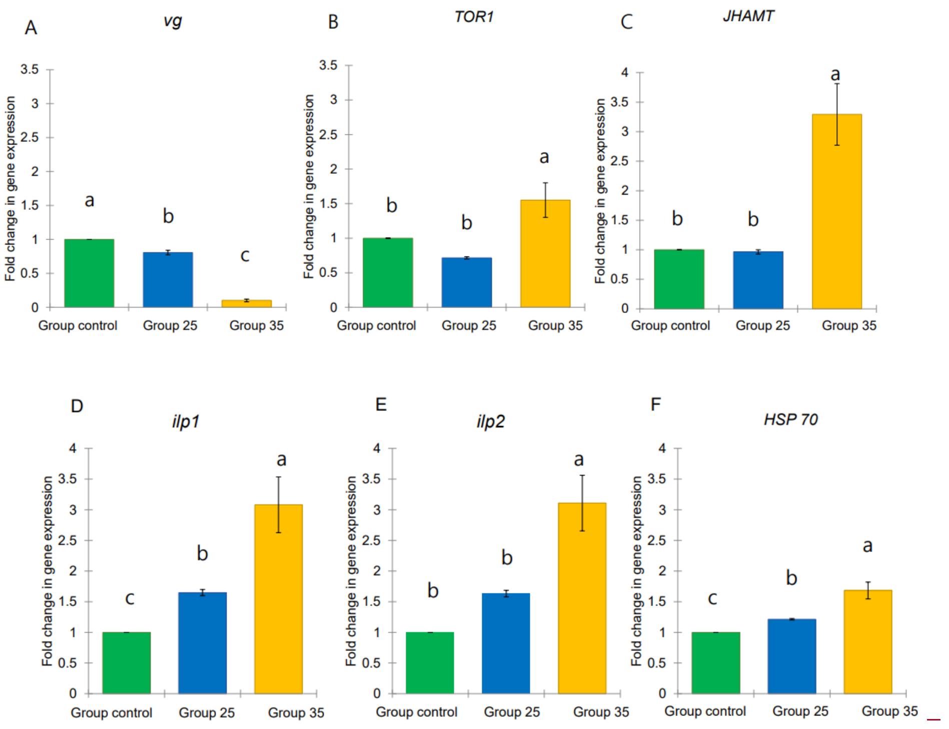

Fig 7. Gene expression analysis. The gene expression (ddCt) of *vg** **(A),** *TOR1*** **(B),** *JHAMT*** **(C),** *ilp1*** **(D),** *ilp2*** **(E),** and *HSP70*** **(F)** in long-lived honey bees at the end of overwintering (February) was evaluated. Statistical significance was determined using one-way ANOVA, Duncan's post hoc tests ($p < 0.05$, indicated by different lowercase letters above the columns). The bars in the graphs represent the mean ± SD. *n=9. **n=15.

Expression of *vg* gene was significantly upregulated (ANOVA, Duncan's post-hoc test, $p < 0.0$0015) in bees from Group control and 25 compared to those from Group 35. In contrast, the expression of the *TOR1, JHAMT, ilp1, ilp2*, and *HSP70* genes was significantly downregulated (same testANOVA, $p <$ 0.001 for *TOR1*; $p <$ 0.00010.05

Formatted: Normal, Centered, Don't add space between paragraphs of the same style, Don't swap indents on facing pages

Formatted: Font: Italic

Formatted: Font: Italic

for other listed genes) in the same bees, suggesting aging in bees from Group 35 compared to Groups control and 25 **(Fig. 7B, C, D, E, and F)**. However, there were no significant differences in the expression of *TOR1, JHAMT*, and *ilp2* (ANOVA~~same test~~, $p > 0.05$) between bees from Group control and Group 25. The expression levels of *ilp1* and *HSP70* were significantly lower (ANOVA~~same test~~, $p < 0.05$) in the ones from Group control than in Group 25.

Discussion

Key triggers influencing the transition of insects from summer to winter physiology include photoperiod, ambient temperature, brood microclimate, availability of pollen, and some yet-to-be-identified factors [25, 64, 65], which obviously have a particular influence on overwintering physiological state, and also linked to aging and subsequent winter loss in ~~honey~~ bees.

However, due to the complex and unclear interactions among all these triggers, we designed an experiment in which only one factor~~trigger~~ associated with ~~of climate~~global warming ~~,~~ ambient temperature (25, 35°C, and a control), was manipulated during the ~~previous~~ late summer, September, and October, and kept constant in experimental rooms. Colony development and brood~~nest~~ climate regulation were investigated in August and September, during which long-lived (winter) bees were reared and marked to study the physiological state~~biological age~~ of ~~honey~~ bees alongside their clear chronological age in February. Physiology~~Aging~~

Formatted: Indent: First line: 1 ch

was assessed using two physiological markers (~~acini~~acinus size ~~area~~ and ~~mass of~~ lipid content in the fat body) and six molecular markers (*vg, TOR, JHAMT, ilp1, ilp2*, and *HSP70*) at the beginning of February, during the expected diapause continuation.

Since other colony parameters (e.g., food availability, hive size, and environmental conditions) excluding ambient temperature were consistent during the experiment (in summer and fall), the observed colony development in the Groups is likely due to the altered thermal environments, which can be the effects of temperature values and the constant diurnal temperature regime. It is crucial during ambient temperature extremes and heat waves since honey bee colonies rely on precise temperature regulation to maintain the brood microclimate [64] and support metabolic activity [4].

It was found that ~~prolonged climate warming significantly weakened colony development in Groups 25 and 35 during the fall season (as of September 18) compared to the control group. This observation supports the importance of day-night and seasonal temperature fluctuations for natural colony development [66], as other colony parameters were the same.~~ constant diurnal and seasonal temperatures (25°C and 35°C) significantly impaired colony development in Groups 25 and 35 in the fall season (from September 18 onward), resulting in reduced brood and worker numbers compared to the Group control. Also, brood microclimate was effectively regulated by the bees. Despite a 10°C difference in the experimental ambient temperatures, their efforts reduced this difference to approximately 3°C (**Fig. 3**).

Formatted: Indent: Left 0 ch, First line: 0 ch

Formatted: Indent: First line: 1.5 ch

Formatted: Font: Bold

Bees in Group 25 raised the brood temperature to about 30°C, while those in Group 35 lowered the brood temperature to around 33°C, slightly below the typical brood temperature of around 34.5°C [67], reflecting well-documented microclimate regulation behaviors [21, 22].

Reduced brood and worker numbers, combined with challenges in brood temperature regulation, suggest that constant temperatures affect colony performance more than ambient temperature alone, as the average ambient temperatures for the Control and 25°C groups in summer showed no significant difference. However, the brood temperature responses in Groups 25 and 35 suggest different survival strategies for the colonies. ~~This colony development, as a response to ambient temperature, is closely related to honey bees' regulation of the nest microclimate. A temperature difference of 10°C between Groups 25 and 35 was assessed to study this. However, in August and September, bees adjusted this brood temperature difference to 3.403°C (**Fig. 3**). To achieve this, honey bees in Group 25 heated the brood area to a subnormal temperature of 30.00 ± 0.982 °C, while those in Group 35 cooled it to 33.398 ± 0.154 °C, which is slightly lower than the typical nest temperature of 34.5 ± 1.5 °C [67]. This well demonstrated known microclimate regulation [21, 22].~~ Although it is unclear~~we don't know if~~ whether the number of bees in colonies from Groups 25 or 35 was in~~may not have been~~ sufficient to heat or cool the hive~~more~~, ~~n~~or if the subnormal brood temperature in these Groups ~~25 and 35~~ were a strategy for ~~may be a mechanism to~~

Formatted: Indent: Left 0 ch, First line: 1.5 ch

rearing long-lived winter bees, these ~~brood~~ temperatures did not prove fatal for colony~~bee~~ survival during overwintering in our experiment (**S1 Table**). –
Furthermore, since Szentgyörgyi et al. [68] reported that short-lived ~~honey~~ bees tend to live longer after developing at a lower temperature (32°C), we favor this ~~preferred the latter~~ explanation. We ~~and~~ hypothesize~~d~~ that reduc~~t~~i~~o~~ng ~~of~~ brood temperature may~~could be~~ trigger ~~related to the development of~~ physiological changes associated with extended longevity (long-live) ~~and molecular markers~~ in winter bees compared to summer bees~~, which are responsible for the processes of aging and longevity~~ [69].

All these factors were expected to influence the physiological state of overwintered honey bees in diapause in February, which hatched under experimental temperatures.

~~Generally, the physiological markers of winter honey bees include hypertrophied HG compared to newly emerged and forager summer bees [28]. Another physiological marker is an enlarged fat body compared to summer bees [27], resulting from the accumulation of nutrients, particularly lipids [30].~~ The known physiological markers of winter bees physiology include hypertrophied hypopharyngeal glands (HG) compared to newly emerged and forager summer bees [28]. This is important because the enlarged HGs of overwintering honey bees secrete more royal jelly to adequately feed the first larvae after diapause [39]. Thus, the nutrition of winter bees includes both the bee's own nutritional needs and the nutritional needs of larvae cared for by nurse bees, which seem to stimulate each other. Another marker is an enlarged fat

Formatted: Indent: First line: 2 ch

body compared to summer bees [27], resulting from the accumulation of nutrients, particularly lipids [30]. These lipids support the production of hormones and molecular markers [40, 70, 71], as well as the synthesis of vg protein, which is later transported to the HG as a source of amino acids after diapause ends [72]. Based on this, larger acini in the HG and higher lipid content in the fat body of bees of the same chronological age may indicate a comparatively better physiological state. On February 5, winter bees from Group 25 and Group control, still in diapause, had the best physiological state compared to bees from Group 35, as In our research, the acinus sizei area in honey bees from Groups 25 and the Group control was significantly larger (ANOVA, Duncan's post hoc, $p < 0.05$) than in those from Group 35. A similar The same trend was observed in the mass of lipid content in the fat bodyfat body's lipid content, although the differences were not statistically significant.

The previously mentioned vg (gene and protein) is the most studied molecular marker in the dual suppression model in bees. Its elevation suppresses the levels of juvenile hormone (JH) and genes related to the JH pathway (*JHAMT*) [38]. This relationship has been Given that the sampling was conducted on February 5, these physiological differences were indicative of the honey bees' diapause condition. The honey bees Groups 25 and control exhibited a higher body nutrient stock compared to the honey bee Group 35.

All of this closely associated with molecular markers [40, 70, 71], especially vg

Formatted: Font: Italic

which is synthesized in the fat body [30]. Vg is then transferred to the HG and used as an amino acid donor for the synthesis of royal jelly to feed larvae after diapause ends [72]. Along with elevated vg levels, another molecular marker, suppressed juvenile hormone (JH) levels, is used to comparequantify the physiological statescondition of both winter andhoney bees compared to summer) honey bees [1, 33, 38]. These molecular levels remain stable during diapause and undergo a switch before the onset of brood rearing [38]. Notably, *vg* gene expression was significantly higher in honey bees from Group 25 and the control, whileereas *JHAMT* gene expression was significantly lower compared to Group 35. This indicates a , different physiological state and suggestsing an some changes in the aging process in the latter group of bees.

To utilize these well-known interactions, we built Elastic Net Regression models for ambient temperature, acinius size area and lipid content in the fat body mass **(Fig 5A, B and BC).** In this both models, only two genes — *vg* and *ilp2* — showed high negative (ambient temperature model) positive and positive (lipid content of fat body and acinus size models) negative coefficient values, respectively. This indicates new knowledge that thhigh ambient temperature is related to *vg* levels in reared honey bees **(Fig 5A)**. It was also shown that at during winter diapause, *vg* and *ilp2* gene expression highly related s are expected to be higher and lower, respectively, with largerto acinus size **(Fig 5C)i area** and lipid content **(Fig 5B)** atfat body mass in honey bees under normal conditions. This supports the well-known relationship between *vg*, – and acinus size, and lipid content of fat body [14, 15, 27, 28,

Formatted: Font: Not Bold

Formatted: Font: Italic

Formatted: Font: Bold

Formatted: Font: Bold

Formatted: Font: Bold

37]~~physiological markers, and suggests a new relationship between molecular marker *ilp2* and physiology markers~~. However, when each characteristic (*vg*, acinus size and lipid content of fat body) were analyzed separately, we found that in bees from Group control and Group 25, compared to Group 35, this relationship was reversed, suggesting potential ~~which also implied some~~ physiological ~~aging~~ issues in bees from Group 35.

To identify genes that can significantly discriminate between the three treatment ~~honey bee~~ groups in our study, Discriminant Analysis (DA) was implemented. Three genes were identified with high scores: —*JHAMT* and~~,~~ *vg*, which are well-known genes related to physiological states and are also suggested to be associated with aging, ~~,~~and the *HSP70* gene~~— were identified with high scores, with the first two being already well-known~~.

~~Even though the *HSP70* gene is synthesized in response to stress [73], Sarıoğlu-Bozkurt et al. [55] reported that its levels also increase in overwintered honey bees from fall to early spring and respond to different supplemental diets along with *vg*, demonstrating the complex regulation of this gene. We found that the highest *HSP70* gene expression was observed in the winter honey bees from Group 35, while the lowest was found in the Group control. B~~Because of ~~*ased on this*~~*HSP70* gene expression responded on stress [73] and diets along with *vg* in bees during winter [55], we suggest that highest *HSP70* gene expression in bees from Group 35,

Formatted: Font: Italic

Formatted: Font: Not Italic

Formatted: Font: Not Italic

Formatted: Font: Not Italic

Formatted: Font: Italic

while the lowest was found in the Group control, honey bees from Group 35 responded on some a stress, possibly related to their dietfferent nutritional needs, which may later stimulate the ending of diapause and the initiation of earlier colony development compared to honey bees from Groups control and 25. We can suggestAlso, the that this can be increaseingd nutritional needs at the end of diapause in of honey bees from Group 35, because are explained by the significant increase in the *TOR1* gene expression was observed there. T, because this evolutionarily conserved gene integrates signals from nutrients (amino acids and energy) and growth factors to regulate cell growth [50, 74, 75]. The nutritional needs of bees and the diet selection they consume from colony stock (bee bread and honey) are linked to their physiology. Winter bees in diapause require carbohydrate-rich food (honey) without a protein source, such as bee bread, to produce royal jelly related to *vg* metabolism, as there is no brood during diapause [17, 27]. This protein (vg) will be used after diapause ends, when the bees' HG increase in size to secrete royal jelly for feeding the larvae [1]. Additionally, significantly higher *HSP70* and *TOR1* gene expressions in honey bees from Group 35, compared to Group control and Group 25, were associated with a lowdecrease in fat body lipid contentweight, although itthis decrease was not yet statistically significant. This supports the aging hypothesis that for temperature-treated honey bees . from Group 35 experienced long-term effects of ambient temperature, which are reflected in physiological changes indicated by two new molecular markers (high expression of the *HSP70* and *TOR1* genes), suggesting

Formatted: Font: Italic

Formatted: Font: Italic

Formatted: Font: Italic

possible differences in diets and increased nutritional needs.However, *TOR1* gene was not selected by Data Mining methods as marker possibly because it had less fluctuation between Groups of honey bees compared to other genes.

The gene *ilp1*and *ilp2* did n'ot show a clear effect in our experiment to ambient temperature, which may be due to several factors. First, the interaction of this genes with nutrition, age, and season may involve more subtle or context-specific metabolic pathways and requires careful consideration due to its complex metabolic relationships influenced by nutrition, age, and season [76, 77]that were not fully captured under our experimental conditions [76]. Additionally, *ilp1*and *ilp2* could be influenced by other unmeasured variables that obscure its direct effects [77]. It is also possible that the gene's influence on these factors is non-linear or dependent on specific thresholds, meaning a wider range of experimental conditions may be required to detect its effect. Further investigation is needed to disentangle these complex relationships and better understand how *ilp1* and *ilp2* functions under varying biological contexts.

In conclusion, our results demonstrate that constant diurnal temperatures negatively impact colony development in summer and fall, reflecting broader issues related to global warming, such as reduced brood and worker numbers, increased brood temperature regulation demands. We hypothesize that reduced brood temperatures trigger physiological changes in bees that may be linked to extended

Formatted: Font: Not Italic

Formatted: Font: Italic

Formatted: Font: Italic

Formatted: Font: Not Italic

Formatted: Indent: Left 0 ch

longevity in winter. Notably, bees raised at the higher temperature (Group 35) exhibited distinct physiological states in bees at the end of diapause in February compared to those from Group 25 and the Group control. Comparative analysis revealed that overwintered bees from Group 35 had smaller acini, lower *vg*, and higher *JHAMT* gene expressions. Additionally, we identified two new molecular markers (high expression of *HSP70* and *TOR1* genes), which had increased expression in overwintered bees from Group 35 related to constant diurnal temperature in summer and fall.

While we cannot definitively conclude that bees from Group 35 faced suboptimal conditions, as the physiological changes did not result in fatal outcomes, further research is warranted. These findings enhance our understanding of how organisms respond to increased diurnal temperatures in summer and fall, providing valuable insights for apiculture practices and guidelines for winter management to prevent losses. Global warming during these seasons alters bee physiology for wintering, potentially threatening their longevity. Unlike bees experiencing typical fall conditions in Korea, which can adapt their colony development, those affected by global warming require assistance safeguarding their colonies at the end of winter and early spring.

~~In summary, our study found that exposure to constant day-night temperatures of 25°C and 35°C negatively impacted colony growth in the fall. The 25°C temperature~~

Formatted: Font: Italic

Formatted: Font: Italic

Formatted: Font: Italic

Formatted: Font: Italic

~~closely resembled natural fall conditions in Incheon, allowing honey bees to regulate the microclimate by lowering brood temperatures. This facilitated the development of winter bees with metabolic markers similar to those of the Group control. In contrast, the 35°C temperature hindered colony development, as honey bees in Group 35 were unable to reduce brood temperatures as effectively as those in Group 25, impairing winter bee development. In February, we observed increased levels of *JHAMT, TOR1*, and *ilp2*, and decreased levels of *vg*, which were associated with reduced nutrient stores in the fat body mass and a smaller acini area. However, these changes did not result in fatal outcomes for honey bee survival in our experiment. These results can be used to correlate climate change factors, such as fall temperatures, with bee responses in winter. Understanding how organisms respond to extreme heat can inform apiculture practices and help develop guidelines for managing honey bees in winter to prevent losses. We believe this research could lead to further studies and the development of a test kit to identify early causes of aging.By linking climate change factors, such as fall temperature, to honey bee responses in winter, this research can inform conservation actions for beekeepers to prevent bee losses.~~

Supporting information

S1 Table. Survival of honey bee colonies at the end of the overwintering (PDF)

S2 Table. PCR primer sequences. (PDF)

S1 Fig. The quality verification plot (specificity vs sensitivity) of Elastic Net Regression model demonstrated a perfectly fitted model linking physiology to molecular markers (gene expression profile). (.tif)

S1 Fig. Gel electrophoresis to verify the amplicons after qPCR. (.tif)

S1 File. Raw data from sensors and XLSTAT 2022.xlsx

S2 File. Raw data of acin~~i~~us size~~ area~~, lipid content of the f~~f~~at body~~ mass~~, and colony perfomance.xlsx

S3 File. Raw data qPCR.xlsx

S4 File. Stat analysis in XLSTAT.xlsx

S5 File. Raw data weather and statistics.xlsx

S6 File. Raw data and stat colony development.xlsx

Acknowledgments

The authors thank BeeOnfarm company (Republic of Korea) for providing the equipment to record the in-hive microclimate. Mention of trade names or commercial products in this publication is solely to provide specific information and does not imply recommendation or endorsement by the INU.

Author Contributions

Conceptualization: Yumi Yun, Hyung-Wook Kwon

Data curation: Yumi Yun

Formal analysis: Olga Frunze, Ravil R. Garafutdinov, Hyunjee Kim

Investigation: Hyung-Wook Kwon, Young-Eun Na

Methodology: Ravil R. Garafutdinov, Olga Frunze, Yumi Yun

Resources: Hyung-Wook Kwon, Young-Eun Na, Hyunjee Kim

Software: Olga Frunze, Ravil R. Garafutdinov

Supervision:, Hyung-Wook Kwon, Young-Eun Na

Visualization: Olga Frunze

Writing-original draft: Olga Frunze, Yumi Yun

Writing-review&editing: Olga Frunze, Yumi Yun, Hyunjee Kim, Hyung-Wook Kwon

Reference

Formatted: Font: 16 pt

1. Döke MA, Frazier M, Grozinger CM. Overwintering honey bees: biology and management. Curr Opin Insect Sci. 2015; 10:185-93.
2. Hristov P, Shumkova R, Palova N, Neov B. Factors associated with honey bee colony losses: a mini-review. Vet Sci. 2020; 7:166.
3. Gray A, Adjlane N, Arab A, Ballis A, Brusbardis V, Dougla AB. et al. Honey bee colony loss rates in 37 countries using the COLOSS survey for winter 2019–2020: the combined effects of operation size, migration and queen replacement. J Apic Res. 2023; 62:204-10.
4. Calovi M, Grozinger CM, Miller DA, Goslee SC. Summer weather conditions influence winter survival of honey bees (*Apis mellifera*) in the northeastern United

States. Sci Rep. 2021; 11: 1553.

5. Dosio A, Mentaschi L, Fischer EM, Wyser K. Extreme heat waves under 1.5ºC and 2ºC global warming. Environ Res. 2018; 13: 054006.
6. Lamb MJ. Temperature and lifespan in *Drosoph*. Nat. 1968; 220: 808–809.
7. Liu RK, Walford RL. Increased growth and lifespan with lowered ambient temperature in annual fish *Cynolebias adloffi*. Nature. 1966; 212: 1277–78.
8. Mishra DM, Bhunia P, Shubham S, Sen,R, Bhunia R, Gulia J, et. al. The impact of weather changes on honey bee populations and disease. J Adv Zool. 2023; 44: 180–90.
9. Conti NB, Sanchez-Alavez M, Winsky-Sommerer R, Morale MC, Lucero J, Brownell S, et al. Transgenic mice with a reduced core body temperature have an increased life span. Science. 2006; 314, 825-8.
10. Fahrenholz L, Lamprecht I, Schricker B. Thermal investigations of a honey bee colony: thermoregulation of the hive during summer and winter and heat production of members of different bee castes. J Comp Physiol. 1989; 159: 551-60.
11. Switanek M, Crailsheim K, Truhetz H, Brodschneider R. Modelling seasonal effects of temperature and precipitation on honey bee winter mortality in a temperate climate. Sci Total Environ. 2017; 579: 1581–7.
12. van Dooremalen C, Gerritsen L, Cornelissen B, van der Steen JJ, van Langevelde F., Blacquière T. Winter survival of individual honey bees and honey bee colonies depends on level of *Varroa destructor* infestation. PloS ONE. 2012; 7: e36285.

13. Morawetz L, Köglberger H, Griesbacher A, Derakhshifar I, Crailsheim K, Brodschneider R, et al. Health status of honey bee colonies (*Apis mellifera*) and disease-related risk factors for colony losses in Austria. PLoS ONE. 2019; 14: e0219293.

14. Fluri P, Lüscher M, Wille H, Gerig L. Changes in weight of the pharyngeal gland and hemolymph titers of juvenile hormone, protein and vitellogenin in worker honey bees. J Insect Physiol. 1982; 28:61-8.

15. Fluri P, Wille H, Gerig L, Luscher M. Juvenile hormone, vitellogenin and hemocyte composition in winter worker honeybees (*Apis mellifera*). Experientia 1977; 33: 1240–41.

16. Crailsheim K. Dependence of protein-metabolism on age and season in the honeybee (*Apis mellifica carnica* Pollm). J. Insect Physiol. 1986; 32:629–34.

17. Denlinger DL. Insect diapause: from a rich history to an exciting future. J Exp Biol. 2023; 226 (4): 245329.

18. Münch D, Kreibich CD, Amdam GV. Aging and its modulation in a long-lived worker caste of the honey bee. J Exp Biol. 2013; 216: 1638–49.

19. Coulibaly KA, Majeed MZ, Sayed SM, Yeo K. Simulated climate warming influenced colony microclimatic conditions and gut bacterial abundance of honeybee subspecies *Apis mellifera ligustica* and *A. mellifera sinisxinyuan*. J Apic Sci. 2022; 66: 15-27.

20. Simpson J. Nest Climate regulation in honey bee colonies. Science. 1961; 133:1327–

1333.

21. Abou-Shaara H, Owayss A, Ibrahim Y, Basuny N. A review of impacts of temperature and relative humidity on various activities of honey bees. Insectes Soc. 2017; 64: 455 – 63.
22. Bencsik M, McVeigh A, Tsakonas C, Kumar T, Chamberlain L, Newton MI. A Monitoring system for carbon dioxide in honeybee hives: An indicator of colony health sensors (Basel). 2023; 23(7):3588.
23. Winston ML. The Biology of the honey bee. Harvard University Press, Cambridge. 1987; 294.
24. Hartfelder K, Engels W. Social insect polymorphism: hormonal regulation of plasticity in development and reproduction in the honeybee. Curr Top Dev Biol. 1998; 40:45-77.
25. Rodrigues MA, Flatt T. Endocrine uncoupling of the trade-off between reproduction and somatic maintenance in eusocial insects. Curr Opin Insect Sci. 2016; 16:1e8.
26. Bresnahan ST, Döke MA, Giray T, Grozinger CM. Tissue-specific transcriptional patterns underlie seasonal phenotypes in honey bees (*Apis mellifera*). Mol Ecol. 2022; 31:174-84.
27. Brejcha M, Prušáková D, Sábová M, Peska V, Černý J, Kodrík D, et al. Seasonal changes in ultrastructure and gene expression in the fat body of worker honey bees. J Insect Physiol. 2023; 146:104504.
28. Deseyn J, Billen J. Age-dependent morphology and ultrastructure of the

hypopharyngeal gland of *Apis mellifera* workers (Hymenoptera, Apidae) Apidologie. 2005; 36: 49-57.

29. Dohanik VT, Medeiros-Santana L, Santos CG, Santana, WC, Serrão JE. Expression and function of the vitellogenin receptor in the hypopharyngeal glands of the honey bee *Apis mellifera* (Hymenoptera: Apidae) workers. Arch Insect Biochem Physiol. 2024; 116: e22120.
30. Smedal B, Brynem M, Kreibich C, Amdam G. Brood pheromone suppresses physiology of extreme longevity in honeybees (*Apis mellifera*). J Exp Biol. 2009; 212: 3795-801.
31. Arrese EL, Soulages JL. Insect fat body: energy, metabolism, and regulation. Annu Rev Entomol. 2010; 55: 207-25.
32. Amdam GV, Aase AL, Seehuus SC, Kim Fondrk M, Norberg K, Hartfelder K. Social reversal of immunosenescence in honey bee workers. Exp Ger Ontol. 2005; 40: 939-47.
33. Knoll S, Pinna W, Varcasia A, Scala A, Cappa MG. The honey bee (*Apis mellifera* L., 1758) and the seasonal adaptation of productions. Highlights on summer to winter transition and back to summer metabolic activity. A review. Livest Sci. 2020; 235: 104011.
34. Schilcher F, Scheine R. New insight into molecular mechanisms underlying division of labor in honeybees. Curr Opin Insect Sci. 2023; 59: 101080.
35. Scheiner R, Reim T, Søvik E, Entler BV, Barron AB, Thamm M. Learning, gustatory

responsiveness and tyramine differences across nurse and forager honeybees. J Exp Biol. 2017; 220:1443-50.

36. Corby-Harris V, Snyder LA. Measuring hypopharyngeal gland acinus size in honey bee (*Apis mellifera*) workers. J Vis Exp. 2018; 139:58261.

37. Corby-Harris V, Snyder L, Meador C. Fat body lipolysis connects poor nutrition to hypopharyngeal gland degradation in *Apis mellifera*. J Insect Physiol. 2019; 116:1-9.

38. Amdam GV, Omholt SW. The regulatory anatomy of honeybee lifespan. J Theor Biol. 2002; 216: 209-28.

39. Ali H, Alqarni AS, Iqbal J, Owayss AA, Raweh HS, Smith BH. Effect of season and behavioral activity on the hypopharyngeal glands of three honey bee *Apis mellifera* L. races under stressful climatic conditions of central Saudi Arabia. J Hymenopt Res. 2019; 68: 85-101.

40. Alaux C, Soubeyrand S, Prado A, Peruzzi M, Maisonnasse A, Vallon J et al. Measuring biological age to assess colony demographics in honeybees. PLoS ONE. 2018; 13(12): e0209192.

41. Diebel LWM, Rockwood K. Determination of biological age: geriatric assessment *vs* biological biomarkers. Curr Oncol Rep. 2021; 23: 104.

42. Elekonich MM, Schulz DJ, Bloch G, Robinson GE. Juvenile hormone levels in honey bee (*Apis mellifera* L.) foragers: foraging experience and diurnal variation. J Insect Physiol. 2001; 47:1119-25.

43. Guidugli KR, Nascimento AM, Amdam GV, Barchuk AR, Omholt S, Simoes ZL et al. 2005. Vitellogenin regulates hormonal dynamics in the worker caste of a eusocial insect. FEBS Lett. 2005; 579: 4961-65.
44. Amdam GV, Page RE. The developmental genetics and physiology of honeybee societies. Anim Behav. 2010;79(5): 973-80.
45. Goodman WG, Granger NA. The juvenile hormones. In: Lawrence IG, Kostas I, Sarjeet SG. Comprehensive Molecular Insect Science. Elselvier. 2005; pp. 319-408.
46. Li W, Huang ZY, Liu F, Li Z, Yan L, et al. Molecular cloning and characterization of juvenile hormone acid methyltransferase in the honey bee, *Apis mellifera*, and its differential expression during caste differentiation. PLoS ONE. 2013; 8:e68544.
47. Egi Y, Sakamoto K. Genome-wide screening of genes involved in programming diapause in the next generation in silkworm, *Bombyx mori* (Lepidoptera: Bombycidae). Eur J Entomol. 2022; 119:405-12.
48. Corona M, Velarde RA, Remolina S, Moran-Lauter A, Wang Y, Hughes KA et al. Vitellogenin, juvenile hormone, insulin signaling, and queen honey bee longevity. Proc Natl Acad Sci USA. 2007; 104: 7128-33.
49. Zhang W, Wang L, Zhao Y, Wang Y, Chen C, Hu Y, et al. Single-cell transcriptomic analysis of honeybee brains identifies vitellogenin as a caste differentiation-related factor. iScience. 2022; 25: 104643.
50. Barth JMI, Szabad J, Hafen E, Köhler K. Autophagy in *Drosophila* ovaries is induced by starvation and is required for oogenesis. Cell Death Differ. 2010; 18: 915.

51. Patel A, Fondrk MK, Kaftanoglu O, Emore C, Hunt G Frederick K, et al. The making of a queen: TOR pathway is a key player in diphenic caste development. PLoS ONE. 2007; 2: e509.

52. Laplante M, Sabatini DM. mTOR signaling in growth control and disease. Cell. 2012; 149:274-293.

53. Johnson SC, Rabinovitch PS, Kaeberlein M. mTOR is a key modulator of aging and age-related disease. Nature. 2013; 493:338-345.

54. Zoncu R, Efeyan A, Sabatini DM. mTOR: from growth signal integration to cancer, diabetes and aging. Nat Rev Mol Cell Biol. 2011; 12:21-35.

55. Sarioğlu-Bozkurt A, Topal E, Güneş N, Üçeş E, Cornea-Cipcigan M, Coşkun İ, et al. Changes in vitellogenin (*Vg*) and stress protein (*HSP 70*) in honey bee (*Apis mellifera anatoliaca*) groups under different diets linked with physico-chemical, antioxidant and fatty and amino acid profiles. Insects. 2022; 13: 985.

56. Delaplane KS, van der Steen J, Guzman-Novoa E. Standard methods for estimating strength parameters of *Apis mellifera* colonies. J Apic Res. 2013; 52: 1-12.

57. Martin JS. Lipid composition of fat body and its contribution to the maturing oöcytes in *Pyrrhocoris apterus*. J Insect Physiol. 1969; 15:1025-45.

58. Human H, Brodschneider R, Dietemann V, Dively JD, Ellis E, Forsgren I, et al. Miscellaneous standard methods for *Apis mellifera* research. J Apic Res. 2013; 52:1-53.

59. Livak KJ, Schmittgen TD. Analysis of relative gene expression data using real-time

quantitative PCR and the 2(-Delta Delta C(T)) Method. Methods. 2001; 25:402–408.
60. Bustin SA, Benes V, Garson JA, et al. The MIQE guidelines: minimum information for publication of quantitative real-time PCR experiments. Clin Chem. 2009; 55:611-22.
61. Moses AM. Statistical modeling and machine learning for molecular biology. CRC Press Taylor & Francis Group. 2017: 280 p.
62. Jaqaman K, Danuser G. Linking data to models: data regression. Nat Rev Mol Cell Biol. 2006; 7:813–819.
63. Hajian-Tilaki K. Receiver operating characteristic (ROC) curve analysis for medical diagnostic test evaluation. Caspian J Intern Med. 2013; 4: 627-35.
64. Medrzycki P, Sgolastra F, Bortolotti L, Bogo G, Tosi S, Padovani E, et al. Influence of brood rearing temperature on honey bee development and susceptibility to poisoning by pesticides. J Apic Res. 2010; 49:52-59.
65. Quinlan GM, Feuerborn C, Hines HM, Grozinger CM. Beat the heat: thermal respites and access to food associated with increased bumble bee heat tolerance. J Exp Biol. 2023; 226: jeb245924.
66. Stalidzans E, Berzonis A, Temperature changes above the upper hive body reveal the annual development periods of honey bee colonies. Comput Electron Agric. 2013; 90: 1-6.
67. Kovac H, Stabentheiner A, Brodschneider R. Contribution of honeybee drones of different age to colonial thermoregulation. Apidologie. 2009; 40:82-95.

68. Szentgyörgyi H, Czekońska K., Tofilski A. Honey bees are larger and live longer after developing at low temperature. J Therm Biol. 2018; 78:219-226.
69. Kovac H, Crailsheim K. Lifespan of *Apis mellifera carnica* Pollm. infested by *Varroa jacobsoni* Oud. in relation to season and extent of infestation. J Apic Res. 1988; 27: 230-8.
70. Kaur R, Singh D. Molecular markers a valuable tool for species identification of insects: A review. Ann Entomol. 2020; 38:1-20.
71. Daniels BC, Wang Y, Page RE Jr, Amdam GV. Identifying a developmental transition in honey bees using gene expression data. PLoS Comput Biol. 2023; 9(9):e1010704.
72. Ramanathan ANKG, Nair AJ, Sugunan VS. 2018. A review on royal jelly proteins and peptides. J Funct Foods. 2018; 44: 255-64.
73. Zhang Y, Liu Y, Zhang J, Guo Y, Ma E. Molecular cloning and mRNA expression of heat shock protein genes and their response to cadmium stress in the drasshopper *Oxya chinensis*. PLoS ONE. 2015; 10: e0131244.
74. Fingar D, Blenis J. Target of rapamycin (TOR): an integrator of nutrient and growth factor signals and coordinator of cell growth and cell cycle progression. Oncogene. 2004; 23: 3151–71.
75. Fahrbach SE, Nambu JR, Schwartz LM. Programmed cell death in insects. Insect Mol Biol Biochem. 2012; 12:419–449.
76. Nilsen KA, Ihle KE, Frederick K, Fondrk MK, Smedal B, Hartfelder K, et al.

Insulin-like peptide genes in honey bee fat body respond differently to manipulation of social behavioral physiology. J Exp Biol. 2011; 1:1488-97.

77. Chowański S, Walkowiak-Nowicka K, Winkiel M, Marciniak P, Urbański A, Pacholska-Bogalska J. Insulin-like peptides and cross-talk with other factors in the regulation of insect metabolism. Front Physiol. 2021; 12:701203.

Data Availability Statement: All relevant data are the manuscript and its Supporting information files.

Funding: This work was carried out with the support of the Cooperative Research Program for Agriculture Science & Technology Development (Project No. RS-2023-00232749) and the Priority Research Centers Program through the National Research Foundation of Korea (NRF) funded by the Ministry of Education (2020R1A6A1A03041954). The funders had no role in study design, data collection and analysis, decision to publish, or preparation of the manuscript.

Competing interests: The authors have declared that no competing interests exist.

Abbreviations: DA, discriminant analysis; AHC, Agglomerative Hierarchical Clustering; FD, Fisher distance; HG, hypopharyngeal glands; JH, juvenile hormone; *JHAMT, Juvenile Hormone Acid Methyltransferase; vg, vitellogenin; ilp1, insulin-like peptide 1; ilp2, insulin-like peptide 2; HSP70, heat shock protein 70; TOR1, target of rapamycin 1; RpL32, Ribosomal protein L32.*

Dear Editor-in-Chief, Reviewer 1 and Reviewer 2!

Thank you for the time and effort spent reviewing our manuscript number PONE-D-24-13353R1 (**The effect of seasonal temperatures on the physiology of the overwintered honey bee**) and suggesting some important points to consider. We corrected the manuscript according to the reviewers' comments. Our replies are marked by **blue** after the symbol **R** (reply) and **question numbers** for the reviewer's comments. Also, we copied these modifications in response for convenience when they were available.

Please, find below the reviewer comments **(black**) and our response (**blue**)

Reviewer #1: This version of the manuscript is greatly improved. I thank the authors for their extensive effort to revise it.

Having said that, there remain substantial issues that should be addressed.

1. There remain many wordings that are not clear, and minor technical issues. Some of these I commented on in the first draft but the same issues remain! For example, I commented on the overuse of "honey bee" when "bee" or "it" would be simpler. The new version is better, but the term "honey bee" remains used far too much.
Another example: I am confused by sample sizes in the paragraph in the Methods related to HP gland measurement. It is stated that 90 acini were measured and 10 acini/bee, which equals only 9 bees studied. But it also states that there were 30 bees/colony X 9 colonies = 270 bees! Please have an independent person read the manuscript for clarity.
As before, I have marked many issues and have suggested changes that I think will improve the manuscript. I hope the authors will take them to heart, as they surely want their manuscript to be as strong as possible. **R1.** Thank you for comment. We corrected the typo in the M@M chapter regarding the HG measurements description. It should state that there were 90 bees from one group, with 30 bees from one colony.

2. In the first draft, I took issue with the HP glands of winter bees being "hypertrophied". I was absolutely wrong on this, and appreciate the authors defending their wording. **R2.** Thank you.

3. In the original version, the authors had used "acinus" (singular) when in fact they meant to use "acini" (plural). It seems that in making corrections, they changed a correct term, "acinus size", to the incorrect term "acini area". Here, "acinus" (singular) is correct (just as one would write "We measured bee size", not "We measured bees size"). And I prefer "acinus size" rather than "acinus area", but that is up to the authors to decide on. **R3.** Corrected

4. Statistics: There is at least one instance where I believe average values were analyzed rather than the raw data. For example, when measuring acinus size, (line 185, revised doc), if I understand the methodology, the authors averaged the areas for the 10 bees from a colony, then conducted the analysis on the average values. Doing this will eliminate much of the variation upon which the analysis of variance is based. **R4.** Thank you for your comment. Our calculations followed the protocol published by Corby-Harris and Snyder (2018) (see screenshot below). Additionally, we applied the general technique of aggregating replicates for each acinus by computing the average expression for each raw data point within individuals to reduce noise and variability (Vaux et al., 2012). The average acinus size for each individual was used in the subsequent analysis. Data from **30** (not 10) individuals were used from each group.

jove Journal of Visualized Experiments www.jove.com

Video Article

Measuring Hypopharyngeal Gland Acinus Size in Honey Bee (*Apis mellifera*) Workers

Vanessa Corby-Harris[1], Lucy A. Snyder[1]

[1]USDA-ARS, Carl Hayden Bee Research Center

Correspondence to: Vanessa Corby-Harris at vanessa.corby@ars.usda.gov

URL: https://www.jove.com/video/58261
DOI: doi:10.3791/58261

Keywords: Environmental Sciences, Issue 139, *Apis mellifera*, honey bee, hypopharyngeal gland, acini, jelly, Giemsa stain, nutrition

Date Published: 9/14/2018

Citation: Corby-Harris, V., Snyder, L.A. Measuring Hypopharyngeal Gland Acinus Size in Honey Bee (*Apis mellifera*) Workers. *J. Vis. Exp.* (139), e58261, doi:10.3791/58261 (2018).

the hypopharyngeal gland acini of these bees were measured and compared (Figure 2). In each of the three hives, three bees were measured for each age x diet treatment combination. Ten acini were measured for each bee. The acini measurements were averaged for each bee to obtain an average acinus size per bee. These values were then averaged to obtain a value for the age x treatment combination for that hive. ANOVA analysis on the acinus measurements showed that diet ($F_{1,8}$= 2.65, p = 0.001), age ($F_{1,8}$ = 10.03, p = 0.013), and the interaction between diet x age ($F_{1,8}$= 0.02, p = 0.020) [illegible]

Vaux, D. L., Fidler, F., & Cumming, G. (2012). Replicates and repeats--what is the difference and is it significant? A brief discussion of statistics and experimental design. EMBO reports, 13(4), 291–296.

PDF questions

(comment in line 63) Vibrating vs contract flight muscles in bees for thermoregulation in the winter cluster. **R:** Thank you for comment. Vibrating muscles are specialized for rapid, high-frequency oscillations, enabling honey bees to generate heat for thermoregulation, even when their wings are not flapping. These muscles can contract rapidly without significant shortening, allowing for vibration instead of traditional movement. In the winter cluster, the vibrating thoracic muscles of honey bees play a crucial role in warming the cluster, helping to maintain a stable temperature necessary for survival during cold conditions (Reddy and Kavitha, 2016). In contrast, contractile muscles are primarily responsible for movement and do not contribute significantly to heat generation in this context. Therefore, vibrating muscles are essential for effective thermoregulation in honey bee clusters during winter.

Reference for response:

Current Biotica **10(1):89-101, 2016** **ISSN 0973-4031**

Thermoregulation in honey bees: Mechanism and Adaptations

P. V. Rami Reddy * **and** ***S. J. Kavitha***
Division of Entomology and Nematology, ICAR-Indian Institute of Horticultural Research
Hessaraghatta Lake P. O., Bengaluru – 560089, Karnataka, India
*E-mail: pvreddy2011@gmail.com

Current Biotica 10(1):89-101, 2016 ISSN 0973-4031

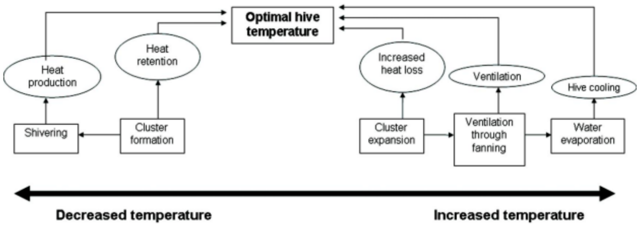

Fig. 1. ***Mechanisms of thermoregulation within a beehive***

(Source: Mathis and Tarpy, 2007)

The flight muscles are one of the sources of active heat production and are metabolically very active in the bee thorax which helps in thermogenesis at cool air temperatures. The muscles help in converting chemical energy to mechanical energy (Esch and Bastian, 1968; Josephson, 1981). By uncoupling these muscles from the wings with the help of steering muscles which contract and relax, metabolic rate of these muscles is increased; thus warmth is produced by this distinctive way. Muscles tremble and lead to vibrations as the antagonistic muscles work against one another. In workers of *A. mellifera*, the flight musculature makes up about 20 per cent of the total body mass. The metabolic rate in noted (Clark *et al*., 1973; Newsholme *et al*., 1972).

The nurse bees that are usually two day old act as endothermic frequently. Thorax acts as a central endothermic region in honey bees. For decreasing the heat dissipation produced by these nurse bees, other bees pack themselves around them on the comb. In young worker bees, the ability of active heat production by the thoracic muscles develops in their first two days of life (Vollmann *et al*., 2004). Heater bees sit completely motionless for about 30 minutes at the capped cells and press thoraces on to the cap cells. Antennal tips contain numerous heat sensitive receptors and help

(comment in line 184) "averaging the values within the 30 bees…" **R1, R4, R:** Thank you. We need to point out that, as mentioned in M@M (HG measurements), '10 randomly selected acini per bee' were measured, and this data was averaged. Therefore, we also accounted for within-colony variation. We corrected sentence.

with ToupView software (Touptek, Hangzhou, China) installed on a PC. Only acini with clearly defined borders were included. Acinus size was averaged within each individual.

SNBL_GY 삭제됨: s
SNBL_GY 삭제됨: i
SNBL_GY 삭제됨: areas

(comment in line 271) **R:** AUC is used to evaluate the performance of a statistical model. An AUC of up to 1.0 indicates good performance, meaning the model effectively distinguishes between the two classes. We corrected this sentence.

markers (gene expression profile) (**S1 Fig**). The area under the curve (AUC) can range from 0.5 to 1.0, with a preference for higher scores, meaning that the model effectively distinguishes between the two classes.

(comment in line 289) **R**: These graphs presented in Supplementary material, S5 File.

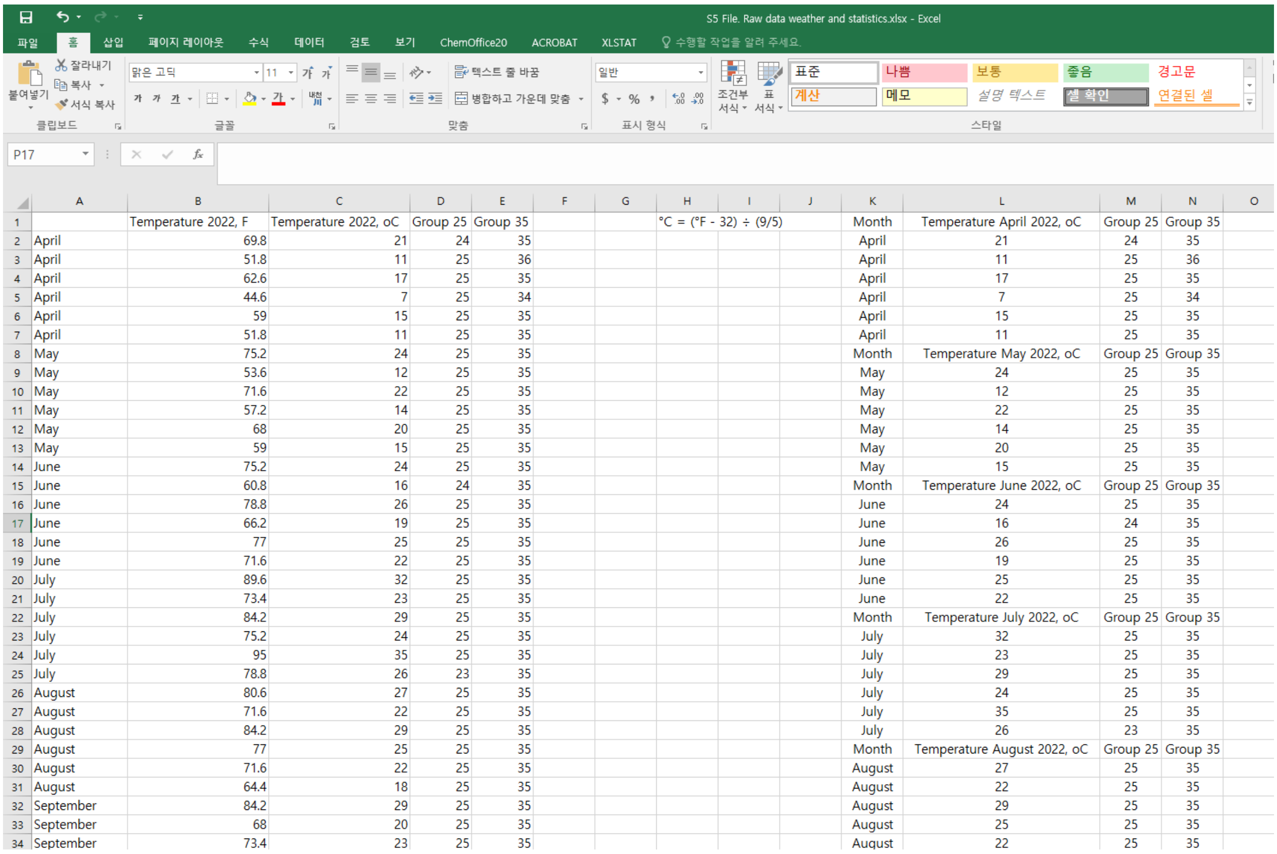

	A	B	C	D	E	H	K	L	M	N
1		Temperature 2022, F	Temperature 2022, oC	Group 25	Group 35	°C = (°F - 32) ÷ (9/5)	Month	Temperature April 2022, oC	Group 25	Group 35
2	April	69.8	21	24	35		April	21	24	35
3	April	51.8	11	25	36		April	11	25	36
4	April	62.6	17	25	35		April	17	25	35
5	April	44.6	7	25	34		April	7	25	34
6	April	59	15	25	35		April	15	25	35
7	April	51.8	11	25	35		April	11	25	35
8	May	75.2	24	25	35		Month	Temperature May 2022, oC	Group 25	Group 35
9	May	53.6	12	25	35		May	24	25	35
10	May	71.6	22	25	35		May	12	25	35
11	May	57.2	14	25	35		May	22	25	35
12	May	68	20	25	35		May	14	25	35
13	May	59	15	25	35		May	20	25	35
14	June	75.2	24	25	35		May	15	25	35
15	June	60.8	16	24	35		Month	Temperature June 2022, oC	Group 25	Group 35
16	June	78.8	26	25	35		June	24	25	35
17	June	66.2	19	25	35		June	16	24	35
18	June	77	25	25	35		June	26	25	35
19	June	71.6	22	25	35		June	19	25	35
20	July	89.6	32	25	35		June	25	25	35
21	July	73.4	23	25	35		June	22	25	35
22	July	84.2	29	25	35		Month	Temperature July 2022, oC	Group 25	Group 35
23	July	75.2	24	25	35		July	32	25	35
24	July	95	35	25	35		July	23	25	35
25	July	78.8	26	23	35		July	29	25	35
26	August	80.6	27	25	35		July	24	25	35
27	August	71.6	22	25	35		July	35	25	35
28	August	84.2	29	25	35		July	26	23	35
29	August	77	25	25	35		Month	Temperature August 2022, oC	Group 25	Group 35
30	August	71.6	22	25	35		August	27	25	35
31	August	64.4	18	25	35		August	22	25	35
32	September	84.2	29	25	35		August	29	25	35
33	September	68	20	25	35		August	25	25	35
34	September	73.4	23	25	35		August	22	25	35

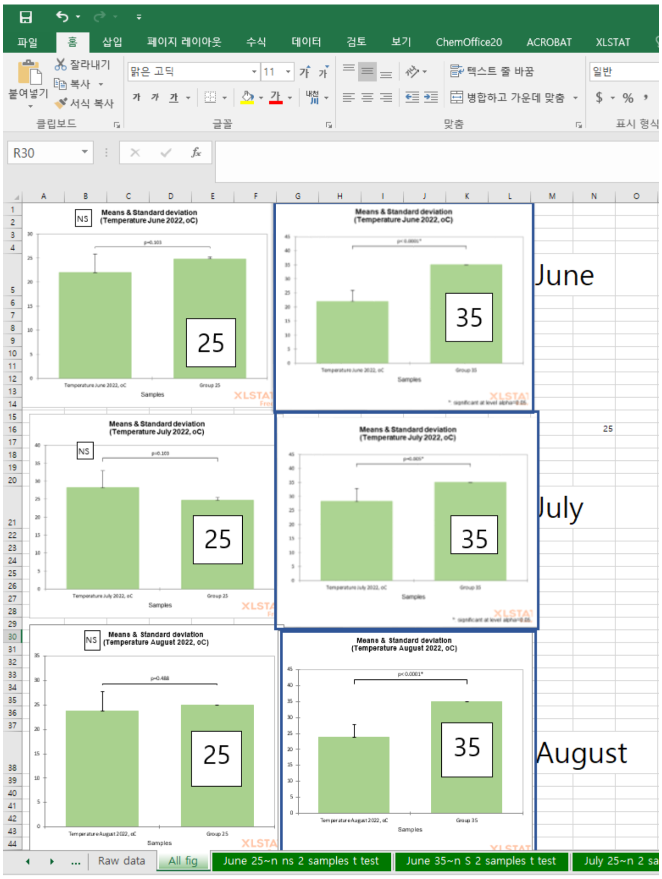

(comment from lines 371 and 377) **R**: Thank you for comment. The acinus size is very small, and when calculated in SI units, the standard deviation (SD) is also small.

Other comments in PDF about grammar were accepted and corrected in the manuscript.

Reviewer #2: The authors indeed improved the manuscript in comparison to the previous version. They incorporated many aspects as suggested. Many aspects and parts of the manuscript still need improvements and corrections.

Major comment

5. I suggest the authors again take their results and think deeply about their meaning in respect to temperature, nutrition and season. I advise to first stick to temperature, nutrition and season (the factors accounted for in the experiment) and only later in the discussion start to give a putative link to age. The authors should take a step back and consider how the results of the different physiological and molecular markers can be linked and interpreted, which picture does emerge. At the moment I find the results and especially the discussion highly confusing since the focus constantly shifts between treatment, age, nutrition, season,... their connection should become clearer. The clear link between the results found for the different factors is missing. E.g. do they hint in the same direction or not? I know that this is not easy because several factors are putatively associated with multiple effects, but what is the take home message? What are the results from this study and what kind of interpretation is possible? **R5.** Thank you for your comment. We examined the long-term effects of summer/fall temperatures (treatment) on honey bee physiology for bees that developed during that period and survived through the winter for 4.5 months. Although the bees are chronologically the same age, their comparative physiological state, as indicated by internal characteristics and reflecting senescence (aging), reveals some differences. We attribute these differences to the summer/fall temperatures, given the consistency of other factors. Nutrition and season were general factors for all honey bees and were not controlled. We agree that this design is weak in defining biological age and is more appropriately used for assessing **comparative physiological states** (instead of aging) related to the summer/fall experimental temperatures. We corrected this through manuscript.

Minor

Abstract

6. Line 35: what do you mean by "expected markers"? **R6.** Thank you for your comment. We refer to “expected markers” as potential indicators whose relationship with winter physiology and, possibly, aging has not been widely proven and still requires further clarification, unlike acinus size, vg gene expression, and JH hormone titer. Therefore, a comparatively large acinus size and high vg titer, along with a low JH titer, are physiological markers of younger bees. However, no studies have been conducted on the “expected markers” similar to our research.

7. Line 37: in your study, you simulate "climate warming". I wonder whether it wouldn't make more sense to report that increasing the temperature to 35C leads to faster physiological aging. Thus argument vice versa, since this is what you are actually interested in and what you want to show with this study, right? **R7.** Thank you for your comment. We need to clarify that we suggest that an ambient temperature of 35°C changed the physiological state in long-lived bees, particularly when honey bees are unable to effectively downregulate brood temperature in late summer or fall (due to factors such as insufficient numbers of bees). Additionally, brood temperatures that are typically maintained at normal levels may lead to the rearing of short-lived bees. So, this suggested mechanism depends on the response of the colony to the ambient temperature.

8. Line 39: add "physiological and molecular markers" instead of just markers **R8.** Corrected
9. Line 40: change to "with an upregulation" **R9.** Corrected

Introduction
10. Line 61: this should be "foraging" **R10.** Sorry, I'm confused about where this needs to be added. I’ve attached a screenshot of where I searched. We refer to bees as foragers if they collect water, nectar, or pollen from outside and store it in the combs. However, this activity is not possible in winter, so we cannot refer to bees in diapause as foragers.

maintain a consistent brood microclimate [20-22].

With the onset of fall, external trigger factors initiate the transition of bees through internal processes, leading to the development of winter workers with prolonged longevity, which can last up to 8 months [1]. Briefly, this transition exhibits the classic insect diapause phenotype, characterized by reduced metabolic activity and altered hormonal profiles [14, 23-26]. Unlike other insects, the reduced metabolic activity of honey bees during diapause [17] includes vibrating their wing muscles to generate

11. Line 60-61: are these studies specifically referring to climate warming? Expect for rodents these are all ectotherms and in all ectotherms lifespan depends on temperature... **R11.** Thank you for your comment. In this part of the introduction, we mention 'climate warming or **cooling**' (please see the screenshot below). Additionally, **endotherms** like rodents and pigs and **ectotherms** like *Drosophila* and honey bees serve as **models** for studying animal responses and regulation in reaction to stress triggers. In the manuscript, we discuss the **impact of temperature on mechanisms of longevity** and highlight relevant research in this area. We propose that decreased brood temperature in late summer and fall can trigger extended winter longevity in honey bees. Additionally, our research indicates that climate warming has disrupted the ability of honey bees in colonies from Group 35 to lower brood temperature, leading to changes in physiology. These changes may result in shortened longevity and subsequent winter honey bee loss in some environments.

Conti et al. (2006) reported that a modest, sustained **reduction of core body** temperature prolonged the life span of Hcrt-UCP2 transgenic mice, independent of altered diet or caloric restriction (CR), compared to their wild-type littermates (12% increase in males; 20% increase in females). Thus, the reduction of core body temperature has been proposed to contribute to the increased life span and anti-aging effects associated with CR.

climate warming or cooling [5] impacts the lifespan in flies [6], fish [7], honey bees [8], and rodents [9]. Other factors related to climate change include the quality of

12. Line 62: how do "beekeeper management practices [12, 13], and pesticide exposure from land use" link to climate change? These are factors that affect bees irrespective of climate change? Or do you want to say that the impact of these factors might become even stronger under climate change? **R12.** Thank you for your comment. Honey bee management must be flexible and account for weather conditions ("climate change") to protect bees from becoming weakened. Honey bee responses to pesticides depend on factors such as the type of pesticide, its concentration, the health condition of the bees, the season, the strength of the colony, and climate factors. This complexity is not the focus of our current research and requires further modeling to gain more detailed insights in the future.

13. Line 64: "mitigate the impacts of climate change and prevent honey bee winter mortality are currently lacking" this sounds like you are testing methods to prevent winter mortality? Please rephrase this sentence and don't rise wrong expectations. This may be your final goal, but it goes beyond this study. **R13.** Corrected.

14. Line 95: delete "and impacts internal processes" **R14.** Corrected.

15. Line 104: change "which" to "with" **R15.** Corrected.

16. Line 123: wouldn't "lipid content" be more appropriate? **R16.** Corrected

17. Line 152: "testing them together requires further investigation" what exactly do you mean here? Testing what together? And why further investigations? Do you mean that testing the combined effect of the genes is the aim of this study? **R17.** Thank you for comment. We corrected this sentence. Please, check the screenshot below.

nurses but shows increased expression in foragers [55]. While all these markers are expected to be important for identifying the physiological state in honey bees, testing them together in the same experiment requires evaluating their responses to the same triggers.

SNBL_GY
삭제됨: aging

SNBL_GY
삭제됨: further investigat

18. Line 162: delete "the last" **R18.** Corrected

19. Line 168: How about writing "the aging status by means of physiological and molecular markers"?

After reading the manuscript again I would suggest to write "and to compare the effect of summer temperature on the physiological and molecular state of winter bees". **R19** Thank you, we corrected this sentence. Please, check screenshot below:

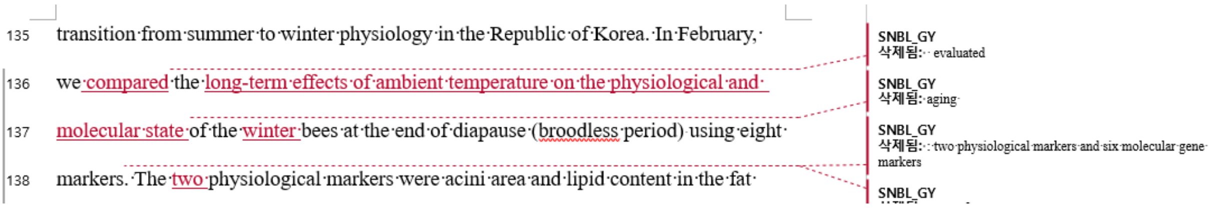
transition from summer to winter physiology in the Republic of Korea. In February, we compared the long-term effects of ambient temperature on the physiological and molecular state of the winter bees at the end of diapause (broodless period) using eight markers. The two physiological markers were acini area and lipid content in the fat

SNBL_GY
삭제됨: evaluated
SNBL_GY
삭제됨: aging
SNBL_GY
삭제됨: two physiological markers and six molecular gene markers
SNBL_GY

20. Line 176/177: this should be "early aging" and I find the conclusion here highly far fetched. I propose to delete this part.

You did not explicitely test whether the physiological and molecular you observe indeed result in differences in aging. I still miss the mentioning of the physiological cost of brood feeding which also may lead to a reduction in lifespan. This has not been taken into account in this study, e.g. brood area to number of bees.

To what extent would this lead to changes in the traits measured here? => all this would be necessary to take into account for a "testkit".

Let's assume I am a beekeeper and use the kit in February to measure the aging status of my bees. What measures can I take in February if I find out that my bees are in an advanced aging status? **R20.** Thank you for your comment. We modified and replaced this sentence with Introduction to Conclusion. We can address practical issues for beekeepers, based on laboratory analysis, and field practice. We found that extremely warm fall weather (climate warming) alters bee physiology, potentially may reducing winter bee longevity. This (longevity) needs to be study in the future research. If this is the case, feeding colonies with sugar/pollen patties on top of the frames starting in early February and covering them with polystyrene insulation are recommended to protect them from cooling in early spring when they may begin brood rearing, specifically for Korean beekeepers. In contrast, bees experiencing typical fall conditions in Korea will develop normally and can adjust their colony development to match the weather.

21. Line 183: change "housed" to "contained" **R21.** Corrected

22. Line 184: change "and beginning egg-laying" to "which started to lay eggs in " **R22.** Corrected

23. Line 186: number of bees missing **R23.** Corrected

24. Line 187: rephrase "populations" **R24.** Corrected

25. Line 200: add "per colony" **R25.** Corrected

26. Line 203: change "Throughout the previous summer" to "Througout summer" **R26.** Corrected

27. Line 204: change "varying room" to "the according" **R27.** Corrected

28. Line 205/206: Rephrase. E.g. "From ... onwards, all colonies were kept under natural conditions. **R28.** Corrected

29. Line 220: **A)** do you mean three measurements "per" minute? = 3x60x24= 4320 measuers/day? What do you mean by approximately one file per day? Is the file information relevant? Maybe just delete.

B) So it turns out that the microclimate was only monitored for 2 weeks in summer. This is super relevant information and should be stated in sections where these numbers are used.

Why was the microclimate only recorded in summer over 15 days and not throughout the experiment?

C) Having this information from two weeks in summer only does not allow us to draw sound conclusions about transcriptomes obtained from bees in February. Many temperature changes could have happened in the meantime... maybe the insulation of the three rooms differs,...?

R29. Thank you for this valuable remark. We divided the response into several parts. **A)** Deleted; **B)** Our research primarily focuses on the rearing and physiology of long-lived bees, particularly during the start transition at the end of summer until the end of diapause in early winter (in February). The microclimate was recorded for 15 days in August (at the end of summer), as this period was critical for assessing the initial conditions under which the last generation of overwintering bees began their development. This timeframe was chosen because August marks a crucial period in which bees are transitioning from summer activity to preparing for winter, even though other fall triggers (such as drops in ambient temperature, reduced nectar/pollen availability, and colder nights) had not yet fully manifested. Monitoring during this specific period allowed us to capture the initial environmental conditions influencing bee longevity before these fall triggers began. We believe this selective focus on the late summer period aligns with our research goals and provides valuable insight into the early stages of the bees' physiological adaptation. However, we acknowledge that a more extended monitoring period, including the fall, could provide a more comprehensive understanding of the microclimate's impact throughout the entire rearing period. This is an important consideration for future studies. **C)** We acknowledge that data from the two weeks in summer may have limitations regarding the conclusions drawn about the transcriptomes of bees sampled in February, especially if these bees had not been specifically marked. However, since honey bees reared from the August brood were marked and tracked specifically to assess

their physiology in February, and all other environmental conditions remained consistent across the groups, we believe that the primary variable influencing the observed physiological changes is temperature. The influence we observed likely follows this pathway: ambient temperature → brood temperature → honey bee physiology. Given that rooms were built within the same house, differences in insulation are not expected. Nevertheless, we recognize that other unmeasured factors might impact the results, and we agree that this is an area that would benefit from further investigation.

30. Line 247: change "bee" to plural "bees"; delete "Apis mellifera ligustica colonies" **R30.** Corrected

31. Line 256: "Total tissue RNA (brain or abdomen) was extracted from fifteen randomly selected" I don't understand this procedure. **A)** Why 15 random bees, and what do you mean by random? **B)** Could this result in 10 bees from a single colony and five bees from a second? You took 10 bees from each of nine colonies, why didn't you take one bee per colony for each of the measures? Or one bee per colony for RNA tissue extraction of the brain and fat body and another bee for the lipid content? **R31.** Thank you for your comment. The number of sampled honey bees is detailed in Figure 2. **A)** Corrected in manuscript. The term "randomly" means that the bees were selected without bias to ensure that the sample was representative of the colony.

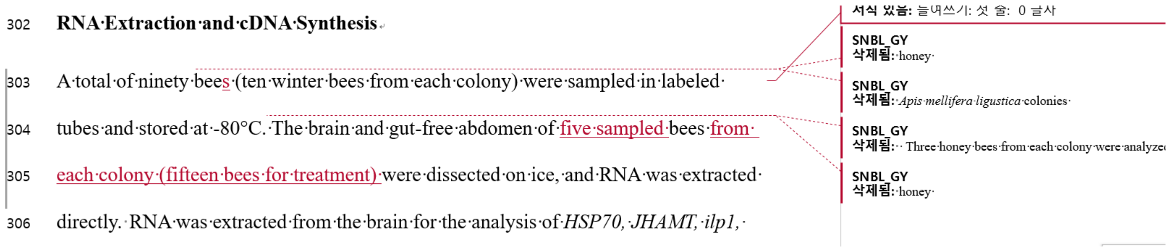

B) The necessary sample size depends on the context and goals of the research (Kadam and Bhalerao, 2010). The sample size of 30 (10*3 colony) and 90 (30*3 colony) bees for molecular and physiology analysis respectively was determined based on the need to obtain reliable data, with a 95% confidence level (as shown in the attached screenshots). For molecular analysis, specifically RNA extraction, pooling samples is a standard practice because the RNA yield from individual bee brains or abdomens is typically low and may not be sufficient for robust analysis. By pooling tissues, we ensure that the RNA samples are representative of the

physiological state of the colony, minimizing variability and improving the accuracy of our results. This method also helps to reduce potential sampling errors.

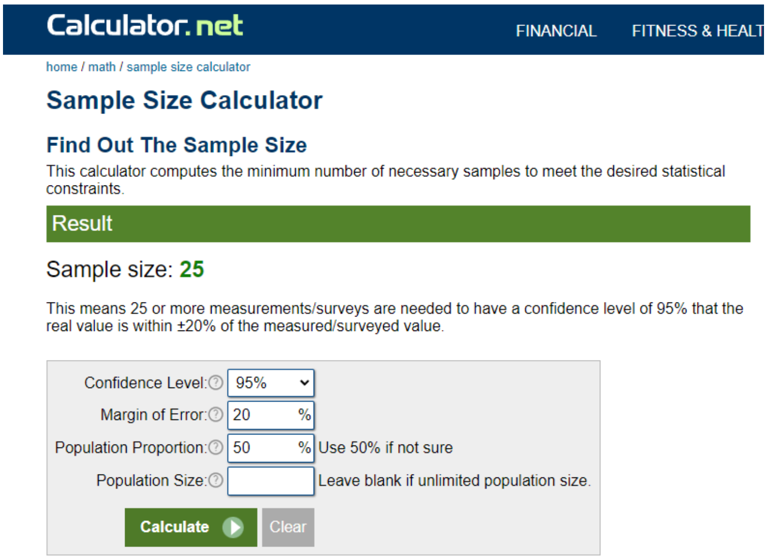

Kadam, P., & Bhalerao, S. (2010). Sample size calculation. International journal of Ayurveda research, 1(1), 55–57.

32. Line 258: this is redundant to line 254, delete. **R32.** Deleted

33. Line 311: "bounded" I guess this should be "bound" **R33.** Corrected

34. Line 313++: in my opinion it is not correct to perform this test here based on the data obtained from the experiment. To conduct such a test, you would first have to perform an experiment that would result in data suitable for this test. I.e. you would have to perform an experiment in which you explicitly investigate individuals with different acini sizes and lipid content, which are otherwise kept under the same conditions. Here in this experiment, you had 3 different temperature regimes and the question is whether these different treatments affect physiological and/or molecular markers. But in my point of view, your data does not allow testing for an effect of gene expression on the acinus size of lipid content. These could all be correlations due to your experimental setup. I suggest removing the ENR from M&M plus the results. **R34.** Thank you for your insightful comment. This point is critical for assessing the suitability of Elastic Net Regression (ENR) for our dataset. As per your advice, we assessed the ENR using 'ambient temperature' as the dependent variable, included this figure, and corrected the relevant text in the Results and Discussion chapters. For your convenience, you can see these modifications in the screenshots below.

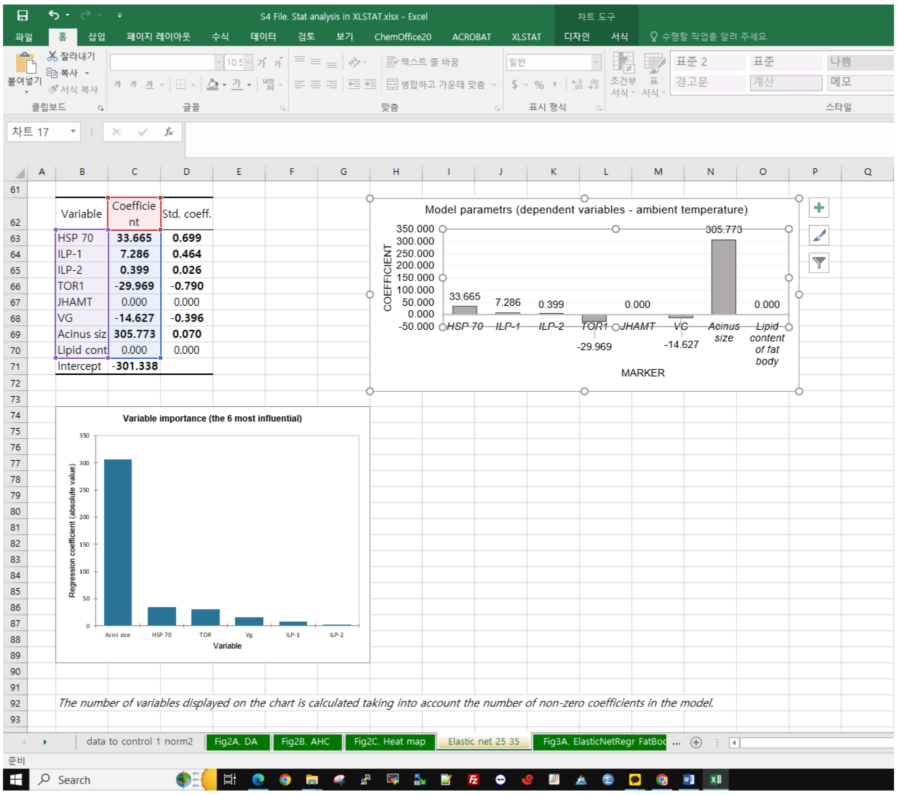

Results

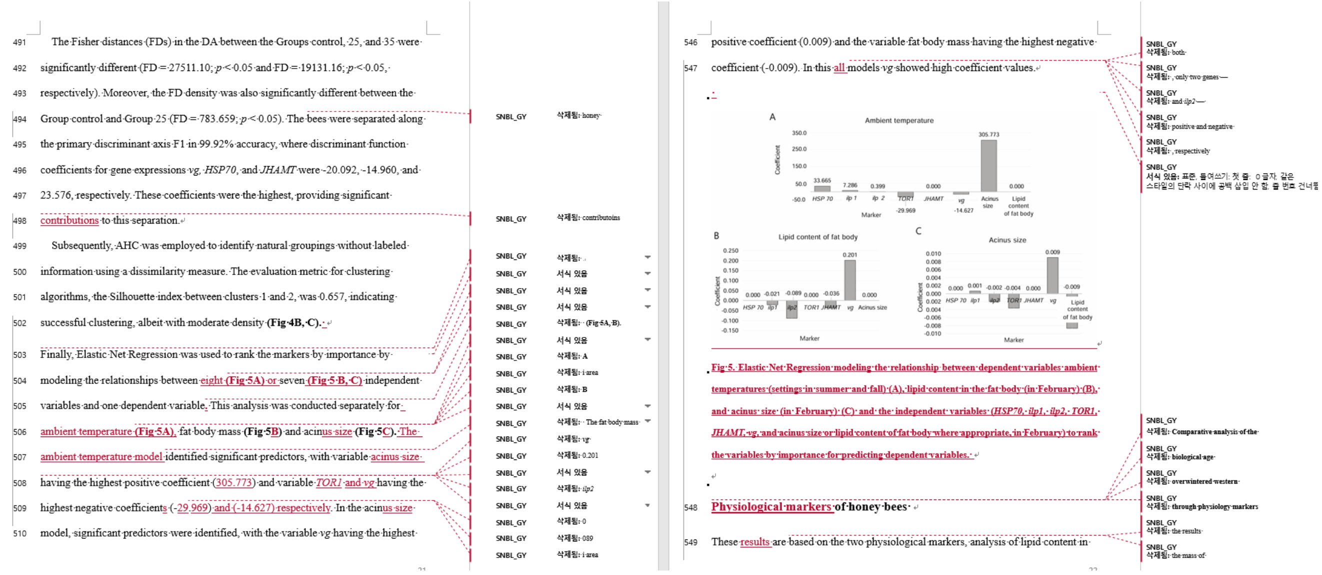

The Fisher distances (FDs) in the DA between the Groups control, 25, and 35 were significantly different (FD = 27511.10; $p < 0.05$ and FD = 19131.16; $p < 0.05$, respectively). Moreover, the FD density was also significantly different between the Group control and Group 25 (FD = 783.659; $p < 0.05$). The bees were separated along the primary discriminant axis F1 in 99.92% accuracy, where discriminant function coefficients for gene expressions *vg*, *HSP70*, and *JHAMT* were -20.092, -14.960, and 23.576, respectively. These coefficients were the highest, providing significant contributions to this separation.

Subsequently, AHC was employed to identify natural groupings without labeled information using a dissimilarity measure. The evaluation metric for clustering algorithms, the Silhouette index between clusters 1 and 2, was 0.657, indicating successful clustering, albeit with moderate density **(Fig 4B, C)**.

Finally, Elastic Net Regression was used to rank the markers by importance by modeling the relationships between eight **(Fig 5A)** or seven **(Fig 5 B, C)** independent variables and one dependent variable. This analysis was conducted separately for ambient temperature **(Fig 5A)**, fat body mass **(Fig 5B)** and acinus size **(Fig 5C)**. The ambient temperature model identified significant predictors, with variable acinus size having the highest positive coefficient (305.773) and variable *TOR1* and *vg* having the highest negative coefficients (-29.969) and (-14.627) respectively. In the acinus size model, significant predictors were identified, with the variable *vg* having the highest

positive coefficient (0.009) and the variable fat body mass having the highest negative coefficient (-0.009). In this all models *vg* showed high coefficient values.

Fig 5. Elastic Net Regression modeling the relationship between dependent variables ambient temperatures (settings in summer and fall) (A), lipid content in the fat body (in February) (B), and acinus size (in February) (C) and the independent variables (*HSP70, ilp1, ilp2, TOR1, JHAMT, vg*, and acinus size or lipid content of fat body where appropriate, in February) to rank the variables by importance for predicting dependent variables.

Physiological markers of honey bees

These results are based on the two physiological markers, analysis of lipid content in

Discussion

process in the latter group of bees.

To utilize these well-known interactions, we built Elastic Net Regression models for ambient temperature, acinus size and lipid content in the fat body **(Fig 5A, B and C)**. In this models *vg* showed high negative (ambient temperature model) and positive (lipid content of fat body and acinus size models) coefficient values. This indicates that high ambient temperature relate to *vg* level in reared honey bees **(Fig 5A)**. Also, during winter diapause, *vg* gene expression highly related to acinus size **(Fig 5C)** and lipid content **(Fig 5B)** at bees under normal conditions. This supports the well-known relationship between *vg*, acinus size, and lipid content of fat body. However, when each characteristic (*vg*, acinus size and lipid content of fat body) were analyzed separately, we found that in bees from Group control and Group 25, compared to Group 35, this relationship was reversed, suggesting potential physiological issues in bees from Group 35.

To identify genes that can significantly discriminate between bee groups,

SNBL_GY 삭제됨: i…s size area…and lip
SNBL_GY 서식 있음
SNBL_GY 삭제됨: at…uring winter
SNBL_GY 서식 있음
SNBL_GY 삭제됨: i area
SNBL_GY 서식 있음
SNBL_GY 삭제됨: fat body mass
SNBL_GY 서식 있음
SNBL_GY 삭제됨: which also implied
SNBL_GY 삭제됨:

Our experimental design included measurements of acinus size and lipid content of fat body in honey bees raised under controlled temperatures during the summer/fall seasons. These marked bees were then overwintered, and by February, all groups experienced identical conditions, eliminating direct temperature effects from the experiment. Therefore, the acinus size and lipid content reflected the initial experimental temperatures, while gene expression responded to the current overwintering environment and physiological state. Because acini size and lipid content still corresponded to the experimental conditions, we included them as dependent variables in the ENR model. ENR is particularly effective in managing correlated features and addressing multicollinearity, making it a suitable tool for our analysis. Although we are cautious not to interpret these findings over, we believe they add valuable insights and should be included in the results.

Results

35. Line 353: "Although significant temperature differences were not 353 detected, a stable elevated night temperature is one of the factors of climate warming 354 that influences bees." I don't understand what the authors want to convey here? Please clarify. **R35.** Thank you for your comment. The previous sentence discusses the comparison of ambient temperatures between Group control and Group 25. Although the average day and night temperatures were consistent, natural fluctuations, where days might be warmer and nights cooler, also influence

bees. In our experiment, these natural temperature variations were controlled for and eliminated as a factor.

We have revised this and other sentences in the manuscript. For your reference, we have attached a screenshot below.

and there were no significant differences compared to the natural daily average temperature in the summer months or September (ANOVA, Duncan's post hoc tests, $p > 0.05$). The difference in experimental conditions between the Group control and the Group 25 was due to day-night temperature fluctuations, which were eliminated in Group 25. Although the average day and night temperatures were consistent, natural fluctuations—where days might be warmer and nights cooler—also affect bees. In our experiment, these natural temperature variations were controlled to focus on the impact of a stable elevated night temperature, which is a factor of climate warming influencing bees. However, the temperature in room Group 25 was significantly higher than the average daily temperature in October 2022 (same tests, $p < 0.05$). The daily

36. Lines 355 and 358: change "same tests" to "ANOVA".
Plus I still have the same comments and questions as in the previous round of review concerning the <0.05 values. And this accounts for all "p < 0.05" throughout the results. It is not sufficient to state p < 0.05 if your p is smaller than alpha 0.05. You need to be more precise!! I don't believe that all p-values you obtained range between 0.01-0.05 which would be the margin represented by 0.05. You must have some values which are smaller than that which should either be indicated by the exact p-value or by 0.01 (if between 0.001-0.01) or p<0.001.

R36. Thank you for your comment. We have accepted your notes and corrected the *p*-values to reflect their specific ranges, rather than simply indicating they are below 0.05, as was done in the previous version of the manuscript. We have attached a screenshot of the summary table from the t-test or ANOVA and Duncan post-hoc tests. The calculations are also presented in the "S4 File. Stat analysis in XLSTAT.xlsx," under the sheet named "ANOVA Duncan norm1", "S5 File. Raw data weather and statistics.xlsx"

Summary (LS means) - T:

Category	Vg	Tor	Jhamt	ILP1	HSP 70	Fat body	ILP2	Acini size
35	-5.913 c	-3.837 a	-4.247 a	-3.303 a	-2.173 a	2.000 a	-3.290 a	0.018 b
25	-1.493 a	-4.933 c	-6.000 b	-4.190 b	-2.640 b	4.000 a	-4.203 b	0.027 a
control	-2.603 b	-4.450 b	-5.947 b	-4.910 c	-2.920 c	4.333 a	-4.910 c	0.026 a
Pr > F(Model)	<0.0001	0.001	<0.0001	<0.0001	0.000	0.222	<0.0001	0.006
Significant	Yes	Yes	Yes	Yes	Yes	No	Yes	Yes
Pr > F(T)	<0.0001	0.001	<0.0001	<0.0001	0.000	0.222	<0.0001	0.006
Significant	Yes	Yes	Yes	Yes	Yes	No	Yes	Yes

37. Line 362: here you start writing bees instead of "honey bees" but up to here always "honey bees". Writing bees is fine, just use it consistent throughout the manuscript, i.e. change above. **R37.** Corrected

38. Line 358: you should reference to Fig3 here. **R38.** Sorry, I was confused about the place where I can input references. Figure 3 content includes the sensor data and colony performance. Corrected manuscript after 1st revision:

second room was set at 35°C for five months, which was significantly greater than the average daily temperature during the summer months, September, and October (same tests, $p < 0.05$) ~~(Fig 6E)~~.

Brood microclimate and colony development in August and September

Manuscript without corrections after 1st revision:

having the highest negative coefficient (-0.089). In the acini area model, significant predictors were identified, with the variable *vg* having the highest positive coefficient (0.009) and the variable fat body mass having the highest negative coefficient (-

39. Line 361: I cannot find information in the M&M how you measured number of larvae and capped brood area? How was this measured, how many combs, which area? What do you mean by honey bees' measurements? **R39.** Thank you for your comment. We briefly mention colony performance measurements in lines 158-159 (see screenshot). We used the conventional visual assessment method for honey bee colonies, where each side of the frame was inspected for the percentage of capped area, larvae area, and honey bees (with 100% representing one side of a frame). These percentages were then converted into the number of capped cells, cells with

larvae, or bees. The process is well explained in the reference article (Delaplane et al., 2013).

honey amount (1 frame), and the number of bees (around 3000~3500 bees). Colony strength was assessed through visual inspection of adult population and brood area both before and during the experiment [56]. Two rooms equipped with a climate-[illegible] system were established to maintain bee colonies at 25°C (Group 25) and 35°C (Group 35), simulating climate warming without day and night temperature

SNBL_GY
삭제됨: honey

SNBL_GY Tuesday
삭제됨: populations

SNBL_GY
삭제됨: honey

56. Delaplane KS, van der Steen J, Guzman-Novoa E. Standard methods for estimating strength parameters of *Apis mellifera* colonies. J Apic Res. 2013; 52: 1-12.

Journal of Apicultural Research 52(1): (2013)
DOI 10.3896/IBRA.1.52.1.03

© IBRA 2013

REVIEW ARTICLE

IBRA INTERNATIONAL BEE RESEARCH ASSOCIATION

Standard methods for estimating strength parameters of *Apis mellifera* colonies

Keith S Delaplane[1*], Jozef van der Steen[2] and Ernesto Guzman-Novoa[3]

open-mated in the same vicinity. A more robust option is to

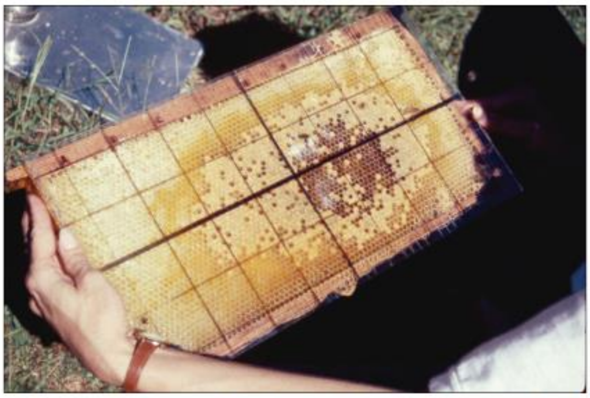

Fig. 2. A transparent plastic grid etched in square centimetres is used to visually sum the surface area of brood.

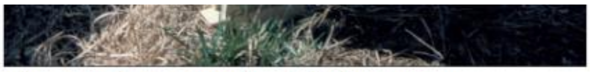

Fig. 3. A ventilated cage made to hold a large common heterogeneous mixture of bees for starting experiments.

7. In order to equalize initial colony populations, it is preferable to make colony-specific caged cohorts. Empty screened cages, ideally made to fit on top of an empty hive, are each pre-weighed or tared with a balance in the field. The large common cage is opened, the bees sprayed with water to reduce flight, then bees transferred from the common cage into the smaller colony-specific cages with the aid of cups or scoops (Fig. 4). Bees are added or removed from each colony-cage until the target weight is achieved and recorded, preferably ≥ 2 kg.

G2 | =(F2*880)

	A	B	C	D	E	F	G	H
1	colony	frame	side	observer 1	observer 2	mean of observers	cm2 covered by bees	bees per side
2	1	1	A	0.2	0.3	0.25	220	303.6
3	1	1	B	0.3	0.5	0.4	352	485.76
4	1	2	A	0.4	0.4	0.4	352	485.76
5	1	2	B	0.5	0.6	0.55	484	667.92
6	1	3	A	0.7	0.8	0.75	660	910.8
7	1	3	B	0.7	0.7	0.7	616	850.08
8	1	4	A	0.5	0.4	0.45	396	546.48
9	1	4	B	0.3	0.3	0.3	264	364.32
10	1	5	A	0.4	0.2	0.3	264	364.32
11	1	5	B	0.1	0	0.05	44	60.72
12							colony bee population	5039.76
13	2	1	A	0	0	0	0	0
14	2	1	B	0.2	0.3	0.25	220	303.6
15	2	2	A	0.4	0.4	0.4	352	485.76
16	2	2	B	0.5	0.6	0.55	484	667.92
17	2	3	A	1	0.9	0.95	836	1153.68
18	2	3	B	0.9	0.8	0.85	748	1032.24
19	2	4	A	0.7	0.8	0.75	660	910.8
20	2	4	B	0.5	0.5	0.5	440	607.2
21	2	5	A	0.3	0.4	0.35	308	425.04
22	2	5	B	0	0	0	0	0
23							colony bee population	5586.24

Fig. 8. Example Excel worksheet for converting raw observer data into colony bee population. See 4.2., paragraph 4.

then first measuring bees in honey supers, then bees in the brood chambers.

4. Raw data are converted into colony bee population using Table 2 and the methods given in Fig. 8.
5. An alternative to visually estimating bees on combs is to rapidly remove each frame and immediately shake the bees into a large plastic bag. The bag is weighed to determine total net weight (kg) of bees, then a fresh sample of *ca.* 20 g bees collected. The bees in the bag are returned to the colony and the sample taken to the lab where it is weighed, frozen, and the bees counted to determine g per bee. Dividing total net weight by g per bee gives colony bee population.

[illegible]

40. Line 366: "Groups control and 25 on August 08 (Fig 3 D, E, F) showed no significant differences,", why was August 8th chosen as the reference date to make the decision on putting loggers into hives or not.

Yes the referred numbers did not differ in August, but when we look at the last September measure the control group differs in all three measures from the 25 and 35 groups, which seem to have significantly lower numbers of larvae, capped brood cells, and bees.

Figure 3: A and B remove "," and put "(°C) and (%) **R40.** Thank you for your comment. We have corrected the y-axis labels in Fig. 3 (A, B). August 8th to September 19th was chosen as the reference period for deciding whether to place loggers in the hives because this is when the honey bee colonies begin rearing the brood that will become the main generation of long-lived bees for overwintering.

41. Line 389: throughout M&M you refer to the candidate genes as genes associated with "six genes related to nutrition (vg, ilp1, ilp2, TOR1),
development (JHAMT), and stress response (HSP70)" it comes out of the blue that these allow to investigate the "biological age". Please make sure that you introduce this link latest in the M&M or stick to the information given in M&M here in the results section. **R41.** Thank you for comment. We modified this. You can look at the changes on the screen shots below.

Analysis of the honey bee groups and the relationship between the markers

The relationship between molecular markers and the physiological state of overwintered bees was analyzed using a dataset comprising two established physiological markers (acini area and lipid content in the fat body) and six molecular markers (*vg*, *TOR1*, *JHAMT*, *ilp1*, *ilp2*, and *HSP70*). Data Mining methods, including

SNBL_GY
삭제됨: biological age

SNBL_GY
삭제됨: honey

SNBL_GY
삭제됨: mass of

19

during the previous summer, September, and October. Colony development and nest climate regulation were investigated in August and September, during which long-lived (winter) bees were reared and marked to study the physiological state of bees alongside their clear chronological age in February. Physiology was assessed using two physiological markers (acini area and mass of lipid content in the fat body) and six molecular markers (*vg*, *TOR*, *JHAMT*, *ilp1*, *ilp2*, and *HSP70*) at the beginning of February, during the expected diapause continuation.

SNBL_GY
삭제됨: biological age

SNBL_GY
삭제됨: honey

SNBL_GY
삭제됨: Aging

42. Line 392: remove "mass of" **R42**. Deleted

43. Fig4 caption: change "mass" to "lipid content" **R43.** Modified

44. Fig5 and according text: as mentioned above, I think the results obtained from this experiment/study do not allow to perform this analysis and the results should be removed. Wouldn't it be possible to perform this analysis with "treatment" as dependent variable to obtain information on the effect of temperature on each of the factors and their importance in desciminating between treatments? **R44.** Please, check response in **R34.**

45. Line 434: I suggest to find a new sub-header. First please make sure that the position and content of subheaders is logical. Currently the previous header fits for all results. You could specify in subheaders that you once look at physiological and then at the molecular markers to discriminate between treatment groups.

Second, I don't think that your experiments really allow to draw sound conclusions about the biological age of bees based on those markers. Feel free discuss this possiblily in the discussion, but your experimental data "only" allows to draw conclusions on the effect different temperature regimes on physiolocial traits and gene expression of candidate genes (which can be associated with...). This by itself is interesting!

To draw sound conclusions on the biological age of bees you would need to experiementally disentangle age from temperature by using different age cohorts, which you do not have. I.e. please be careful and rather remove this statement. Lipid content for example could be related to nutrition only, not age. Maybe bees at higher temperatures have a higher metabolism and thus use up their fat reserves quicker,...??? **R45.** Thank you for your feedback. We have accepted this point and changed “age” to “physiology”, which better aligns with our design. Also, we corrected sub-headers.

Distinguishing honey bee groups through marker ranking

The overwintered bees were distinguished by their physiological state using a dataset

SNBL_GY
삭제됨: and the relationship between the markers

SNBL_GY 2024년 September 06일
삭제됨: relationship between molecular markers and the

Physiological markers of honey bees

These results are based on the two physiological markers, analysis of lipid content in the fat body and the acini area from the hypopharyngeal glands (HGs) in overwintered

SNBL_GY
삭제됨: overwintered western

SNBL_GY
삭제됨: through physiology markers

SNBL_GY 8분 전
삭제됨: the results

SNBL_GY
삭제됨: the mass of

Molecular markers of honey bees

These results include two previously defined task-related markers, *vg* and *JHAMT*, as well as four genes expected to respond to physiology: *TOR1*, *ilp1*, *ilp2*, and *HSP70*

SNBL_GY
삭제됨: overwintered western

SNBL_GY
삭제됨: through molecular markers

SNBL_GY
삭제됨: are the results based on molecular markers. They

SNBL_GY
삭제됨: aging

46. Line 440: you did not measure fat body mass, you measured lipid content. Be specific here and in the following. **R46.** Thank you, corrected in manuscript.

47. Line 448: don't write "same test", be specific. Also in the following sections, i.e. throughout the manuscript. **R47.** Thank you, corrected in manuscript**.**

48. Line 452: "accelerated physiologicaly aging" see above comments concerning this interpretation. Change and downword. **R48.** Changed.

49. Figure 6: fat body mg should be "fat body lipid content (mg)", change other axis labels accordingly also in respect to format, and change figure caption accordingly. **R49**. Thank you for your comment. It has been corrected. Please check the screenshot below for convenience.

Importantly, both the lipid content and the acinus size of the overwintered bees in Group 35 were lower than those in Groups control and 25 **(Fig 6)**, suggesting lower nutrition and different physiological state in bees from Group 35.

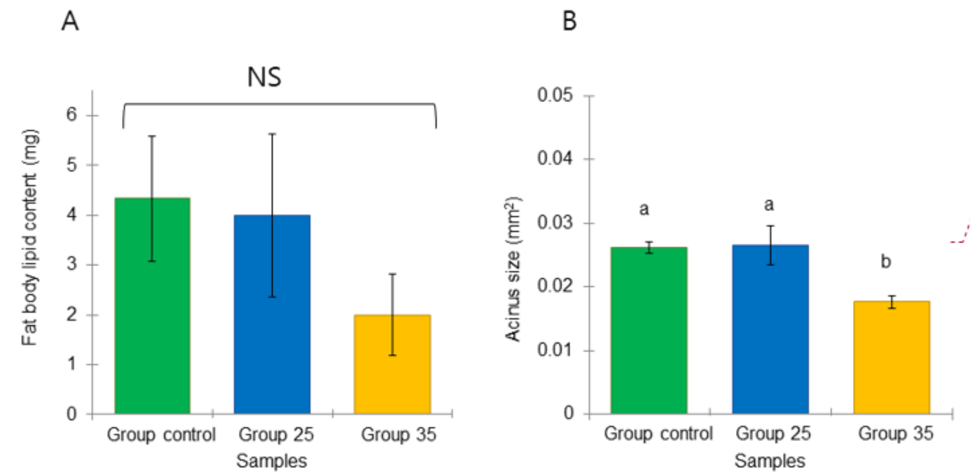

Fig 6. Physiological markers of long-lived honey bees at the end of winter (February) (A) The average of lipid content in the fat body, n = 10 (mean ± SD). **(B)** Average acinus size, n = 30 (mean ± SD). Statistical significance was determined using one-way ANOVA, Duncan's post hoc tests ($p < 0.05$, indicated by different letters above the columns).

50. Line 455: see comment to line 434 and other comments in respect to the use of "age". **R50.** Corrected.

51. Line 458: header says age and here you write task-related.... see above comments **R51.** Corrected.

Discussion

52. Line 489: "prolonged climate warming" I am confused. In the results you say that there are no differences between control and group25, and here you say that group 25 and 30 were warming conditions!? If G25 is similar to the control, then the fact that the brood parameters are lower in both G25 and G30 may be a treatment effect of either the insulated rooms or side effect constant temperatures during larval development, or ....

It might also be better to say "It was found that longer periods of higher tempartures/higher, constant temprатures, as expected under climate warming", as climate is measured over decades and centuries. **R52.** Thank you for your comment. To compare the average monthly temperatures, we analyzed the temperature dataset for the control, 25°C, and 35°C groups, and

found no significant difference using the t-test. This suggests that day/night temperature fluctuations were not sufficient to produce differences. However, as shown in Fig. 1E, the main temperature difference between the control and 25°C groups was in the daytime fluctuations, which also influence honey bee physiology. Additionally, the term 'global warming' was used in the manuscript to describe the unusual continuous day/night warm temperatures. We replaced “climate warming” with 'global warming' and removed the phrase “prolonged climate warming”.

53. Line 494: you actually mention tempearture fluctuations here yourself. Please use this information in the previous sentence and argument along those lines. **R53.** Thank you for comment. We modified this paragraph according to recommendations. Please check the screenshot below for convenience.

It was found that warming, along with constant diurnal and seasonal temperatures, significantly weakened colony development in Groups 25 and 35 during the fall season (as of September 18) compared to the control group. It is suggested that this weakening is attributed to the altered thermal environment, as honey bee colonies rely with temperature to regulate brood microclimate [64] and metabolic activity [4]. Without these, colonies in Groups 25 and 35 experienced disrupted physiological responses, leading to lower rates of brood rearing and overall development. Since other colony parameters (e.g., food availability, hive size, and environmental conditions) remained constant, the observed differences highlight the crucial role of diurnal temperature variations in supporting natural colony development. These findings align with previous studies [66], which emphasize the importance of maintaining a stable thermal regime for optimal colony health and development.

Although the experimental ambient temperatures were set with a 10°C difference, the bees' efforts to maintain optimal developmental temperatures reduced this difference to approximately 3°C (**Fig. 3**). Bees in Group 25 raised the brood

SNBL_GY
삭제됨: mass of

SNBL_GY
삭제됨: prolonged

SNBL_GY
삭제됨: significa
Groups 25 and 35
compared to the co
importance of day-
fluctuations for nat
colony parameters

26

54. Line 495++: rephrase. E.g. "While experimental ambient temperatures were set to 10°C difference, the bees effort to achieve optimal developmental temperatures for the bees reduced

this difference to about 3°C. Bees in Group 25 heated the brood to approximately 30°C, while the Group 35 bees cooled the hive to 33°C, following well demonstrated known microclimate regulation [21, 22]." **R54.** Thank you. Modified. Please check the screenshot below for convenience.

Although the experimental ambient temperatures were set with a 10°C difference, the bees' efforts to maintain optimal developmental temperatures reduced this difference to approximately 3°C (**Fig. 3**). Bees in Group 25 raised the brood temperature to about 30°C, while bees in Group 35 lowered the hive temperature to around 33°C, which is slightly below the typical brood temperature around 34.5°C [67], reflecting well-documented microclimate regulation behaviors [21, 22].

Although we don't know if the number of bees in colonies from Groups 25 or 35

55. Line 514: "related to the development of physiological and molecular markers in winter bees, which are responsible for the processes of aging and longevity [69]" rephrase. **R55.** Thank you. Modified. Please check the screenshot below for convenience.

overwintering in our experiment (**S1 Table**). Furthermore, since Szentgyörgyi et al. [68] reported that short-lived bees tend to live longer after developing at a lower temperature (32°C), we favor this explanation. We hypothesize that reducing brood temperature may trigger physiological conditions associated with extended longevity (long-live) in winter bees compared to summer bees [69].

56. Line 530: in line with comment to lipid content in the results section, acinus size has also been linked to nutritional state in nurse bees (e.g. doi:10.3791/58261). I.e. it is not exclusively an aging marker. This should be taken into consideration and clearly be mentioned in the discussion for both lipid content and acinus size. Or even better first be discussed in a way that it actually fits the result i.e. temperature treatment affected acinus size but not lipid content and then putative interpretations. Statistical results = p-values do not need to be repeated in the discussion. **R56.** Thank you for your question. We corrected this paragraph. Please check the screenshot below for convenience.

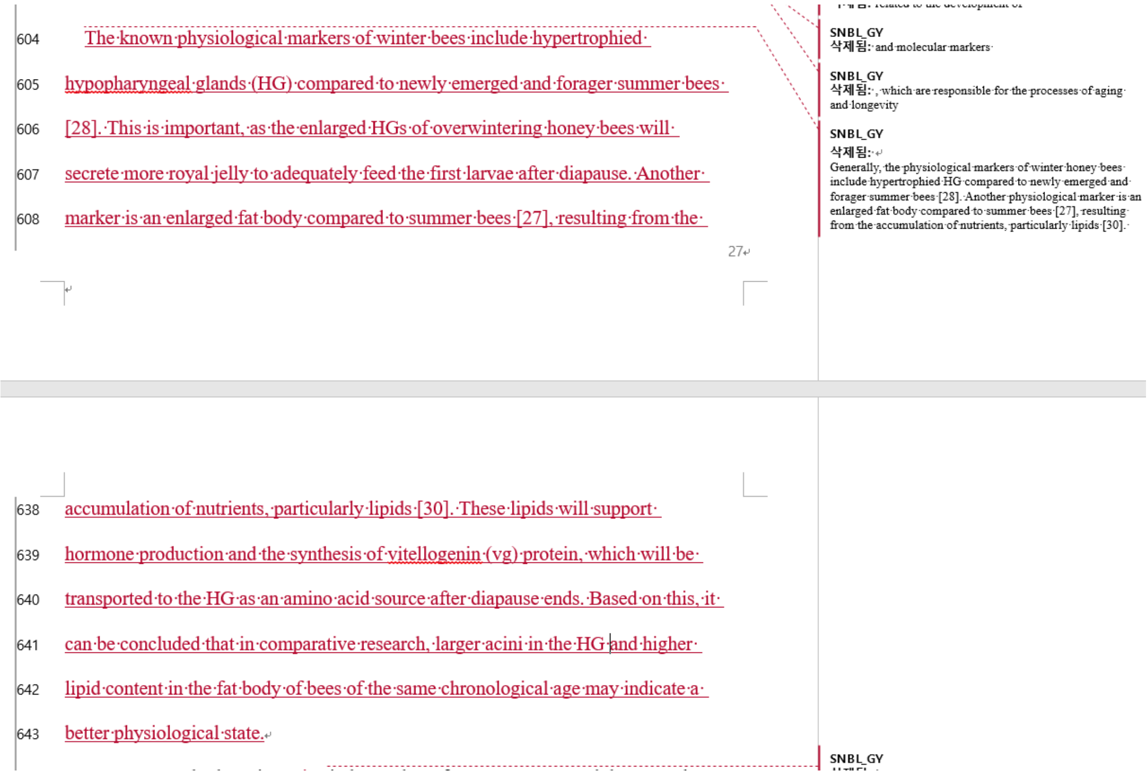
The known physiological markers of winter bees include hypertrophied hypopharyngeal glands (HG) compared to newly emerged and forager summer bees [28]. This is important, as the enlarged HGs of overwintering honey bees will secrete more royal jelly to adequately feed the first larvae after diapause. Another marker is an enlarged fat body compared to summer bees [27], resulting from the

27

accumulation of nutrients, particularly lipids [30]. These lipids will support hormone production and the synthesis of vitellogenin (vg) protein, which will be transported to the HG as an amino acid source after diapause ends. Based on this, it can be concluded that in comparative research, larger acini in the HG and higher lipid content in the fat body of bees of the same chronological age may indicate a better physiological state.

SNBL_GY
삭제됨: and molecular markers

SNBL_GY
삭제됨: , which are responsible for the processes of aging and longevity

SNBL_GY
삭제됨:
Generally, the physiological markers of winter honey bees include hypertrophied HG compared to newly emerged and forager summer bees [28]. Another physiological marker is an enlarged fat body compared to summer bees [27], resulting from the accumulation of nutrients, particularly lipids [30].

SNBL_GY

57. Line 535: why are physiological difference indicative of diapause conditions, and why only in two out of three? Please explain. **R57.** Thank you for your question. Please check the highlighted lines in the screenshot below for convenience.

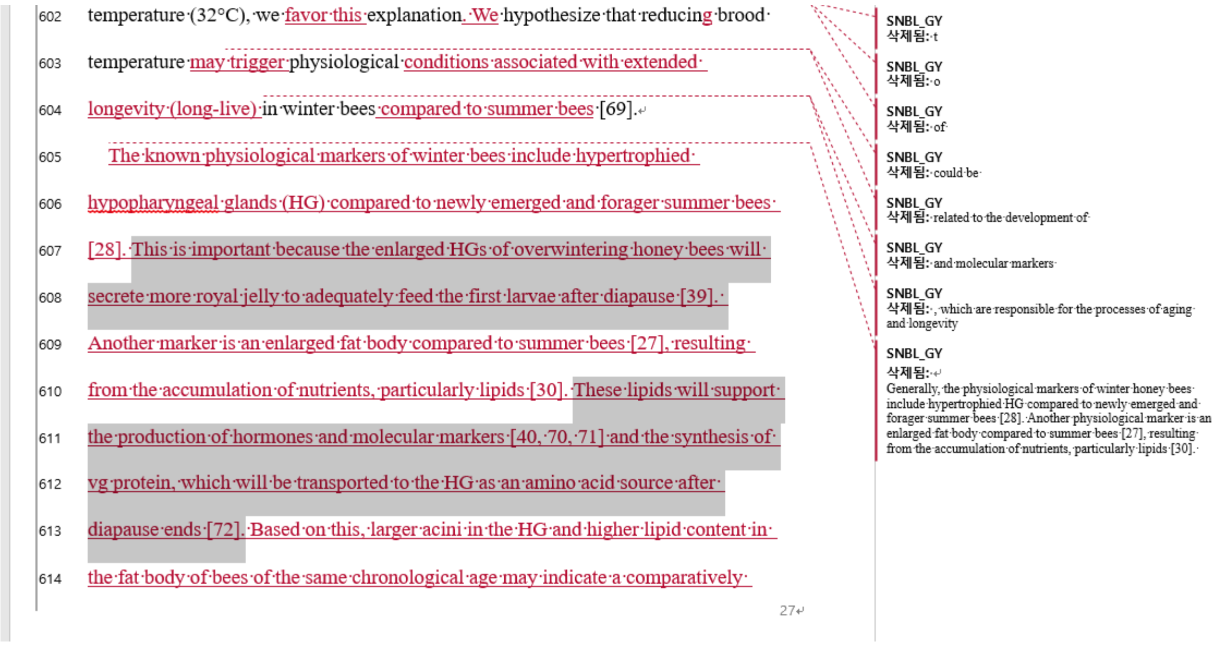
temperature (32°C), we favor this explanation. We hypothesize that reducing brood temperature may trigger physiological conditions associated with extended longevity (long-live) in winter bees compared to summer bees [69].

The known physiological markers of winter bees include hypertrophied hypopharyngeal glands (HG) compared to newly emerged and forager summer bees [28]. This is important because the enlarged HGs of overwintering honey bees will secrete more royal jelly to adequately feed the first larvae after diapause [39]. Another marker is an enlarged fat body compared to summer bees [27], resulting from the accumulation of nutrients, particularly lipids [30]. These lipids will support the production of hormones and molecular markers [40, 70, 71] and the synthesis of vg protein, which will be transported to the HG as an amino acid source after diapause ends [72]. Based on this, larger acini in the HG and higher lipid content in the fat body of bees of the same chronological age may indicate a comparatively

27

SNBL_GY
삭제됨: t

SNBL_GY
삭제됨: o

SNBL_GY
삭제됨: of

SNBL_GY
삭제됨: could be

SNBL_GY
삭제됨: related to the development of

SNBL_GY
삭제됨: and molecular markers

SNBL_GY
삭제됨: , which are responsible for the processes of aging and longevity

SNBL_GY
삭제됨:
Generally, the physiological markers of winter honey bees include hypertrophied HG compared to newly emerged and forager summer bees [28]. Another physiological marker is an enlarged fat body compared to summer bees [27], resulting from the accumulation of nutrients, particularly lipids [30].

58. Line 536: what is your explanation for this? => here in the discussion you refer all differences to nutrition but not age. This is not in line with what you write in the results. Also see my comments there. **R58.** Thank you for your question. We modified the focus from “aging”

to “different physiological states” (related to nutrition) and are suggesting this relationship, but we do not assert it.

59. Line 539: please rephrase this sentence, unclear what "all of this" refers to. **R59.** Thank you for your question. Rephrased.

60. Lines 541-544: These sentences are confusing. How does Vg which is transferred to the HG to be fed to larvae relate suppressed JH and conditions of winter bees? Rephrase and make clearer. **R60.** Thank you for your question. We have revised the sentences to remove any confusing expressions. Please refer to the screenshot below for convenience.

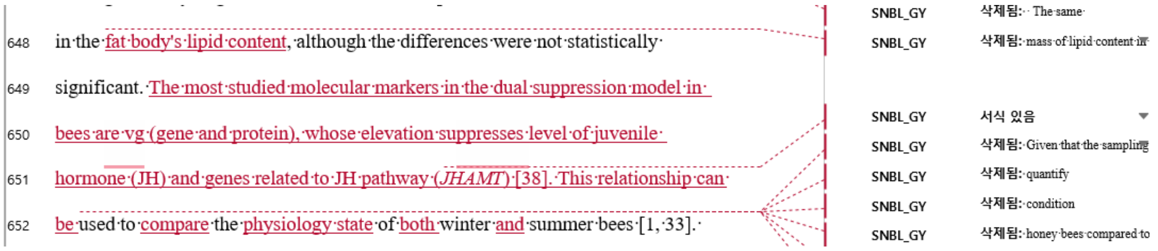
in the fat body's lipid content, although the differences were not statistically significant. The most studied molecular markers in the dual suppression model in bees are vg (gene and protein), whose elevation suppresses level of juvenile hormone (JH) and genes related to JH pathway (*JHAMT*) [38]. This relationship can be used to compare the physiology state of both winter and summer bees [1, 33].

SNBL_GY 삭제됨: The same
SNBL_GY 삭제됨: mass of lipid content in
SNBL_GY 서식 있음
SNBL_GY 삭제됨: Given that the sampling
SNBL_GY 삭제됨: quantify
SNBL_GY 삭제됨: condition
SNBL_GY 삭제됨: honey bees compared to

61. Line 551: vg expression also differs between nurses and foragers, does it differ in nutritioinal states? Nurses generally also have a higher fat content, right? Does it also differ in respect to the amount of brood in need of food? These could all be confounding factors, which at least need to be mentioned and discussed in addition to the aging process. **R61.** Thank you for your comment. The regulation of honey bee tasks is complex, and *vg* expression is indeed influenced by nutritional states. Nurses, with their highly developed hypopharyngeal glands and fat bodies, require *vg* to produce royal jelly. This suggests that both the nutrition of the bee itself and the need to feed larvae play a role in vg expression. Additionally, nurses generally have a higher fat content compared to foragers. This discussion was added. Please refer to the screenshot below for convenience.

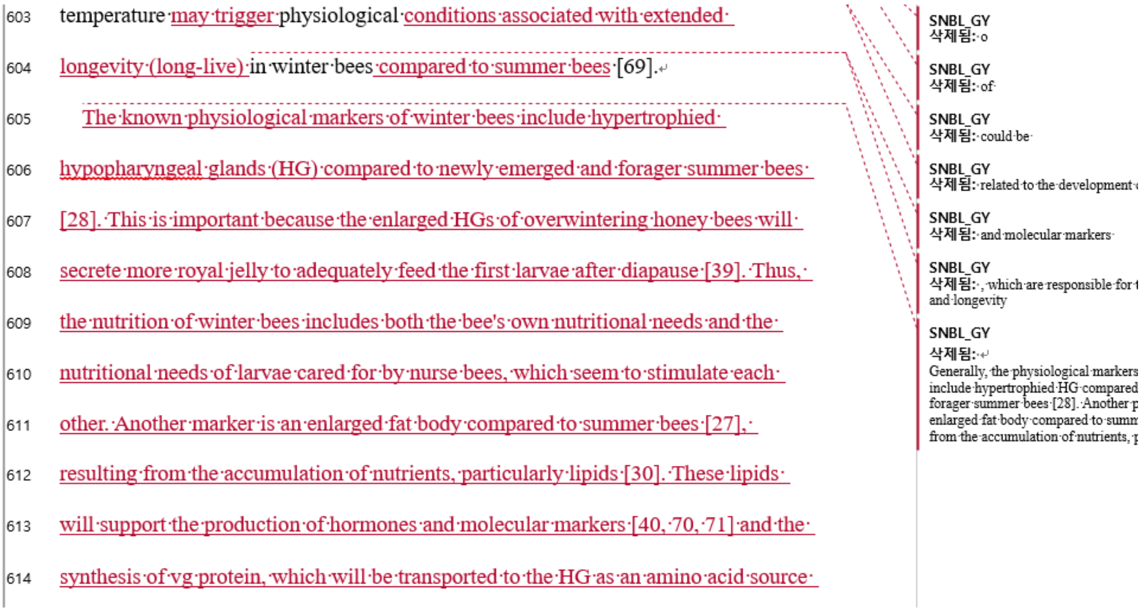
temperature may trigger physiological conditions associated with extended longevity (long-live) in winter bees compared to summer bees [69].

The known physiological markers of winter bees include hypertrophied hypopharyngeal glands (HG) compared to newly emerged and forager summer bees [28]. This is important because the enlarged HGs of overwintering honey bees will secrete more royal jelly to adequately feed the first larvae after diapause [39]. Thus, the nutrition of winter bees includes both the bee's own nutritional needs and the nutritional needs of larvae cared for by nurse bees, which seem to stimulate each other. Another marker is an enlarged fat body compared to summer bees [27], resulting from the accumulation of nutrients, particularly lipids [30]. These lipids will support the production of hormones and molecular markers [40, 70, 71] and the synthesis of vg protein, which will be transported to the HG as an amino acid source

SNBL_GY 삭제됨: o
SNBL_GY 삭제됨: of
SNBL_GY 삭제됨: could be
SNBL_GY 삭제됨: related to the development
SNBL_GY 삭제됨: and molecular markers
SNBL_GY 삭제됨: , which are responsible for and longevity
SNBL_GY 삭제됨: Generally, the physiological markers include hypertrophied HG compared forager summer bees [28]. Another enlarged fat body compared to summ from the accumulation of nutrients,

62. Line 554: see above comments to ENR **R62**. Please, check R34 response.

63. Line 564: this is interesting and relevant. Here you could spend some lines to give information on other studies showing this effect and especially in respect to "being already well-known". Well known for what? Be more specific. **R63.** Thank you for your comment. This expression was about the dual-mutual interaction between vg and JH, which has been corrected in this revision. Additionally, these sentences have been rewritten. Please refer to the screenshot below for convenience.

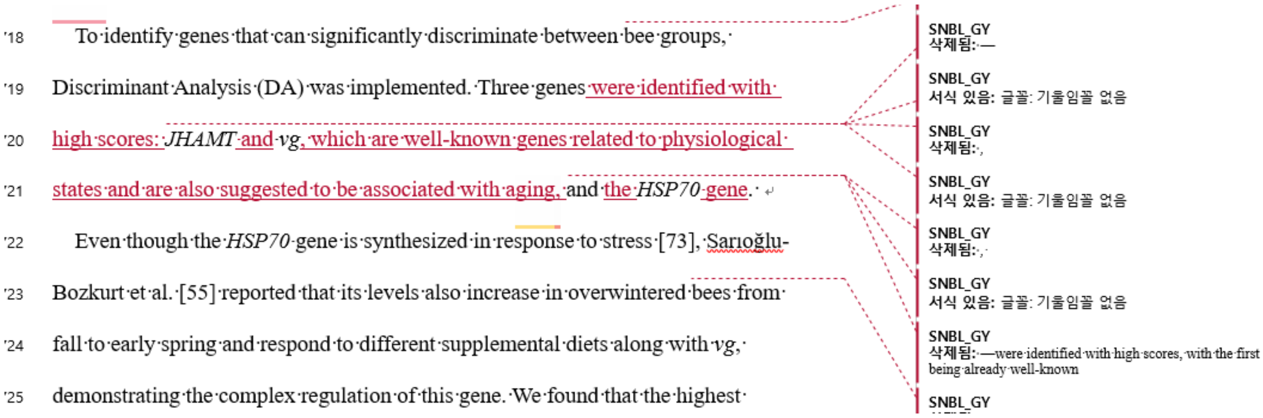
To identify genes that can significantly discriminate between bee groups, Discriminant Analysis (DA) was implemented. Three genes were identified with high scores: *JHAMT* and *vg*, which are well-known genes related to physiological states and are also suggested to be associated with aging, and the *HSP70* gene.

Even though the *HSP70* gene is synthesized in response to stress [73], Sarıoğlu-Bozkurt et al. [55] reported that its levels also increase in overwintered bees from fall to early spring and respond to different supplemental diets along with *vg*, demonstrating the complex regulation of this gene. We found that the highest

SNBL_GY 삭제됨: —
SNBL_GY 서식 있음: 글꼴: 기울임꼴 없음
SNBL_GY 삭제됨: ,
SNBL_GY 서식 있음: 글꼴: 기울임꼴 없음
SNBL_GY 삭제됨: ,
SNBL_GY 서식 있음: 글꼴: 기울임꼴 없음
SNBL_GY 삭제됨: —were identified with high scores, with the first being already well-known
SNBL_GY

64. Line 577-585: above you write that HSP70 can be stress-associated but it has also be shown to differ between overwintering bees from fall to spring. Here you write about putative stress and nutritional needs. Where do nutritional needs come from? This has not been mentioned before. Please write this section more clearly, and more in line with the other arguments,

currently I am lost. **R64.** Thank you for comment. We extended the explanation. Please refer to the screenshot below for convenience.

high scores: *JHAMT* and *vg*, which are well-known genes related to physiological states and are also suggested to be associated with aging, and the *HSP70* gene.

Even though the *HSP70* gene is synthesized in response to stress [73], Sarıoğlu-Bozkurt et al. [55] reported that its levels also increase in overwintered bees from fall to early spring and respond to different supplemental diets along with *vg*, demonstrating the complex regulation of this gene. Thus, stress and different diets are considered factors related to increased *HSP70* expression. The nutritional needs of bees are related to their function. Winter bees in diapause require carbohydrate-rich food without a protein source, such as pollen, for the secretion of royal jelly, as there is no brood during diapause [17, 27]. This will be needed at the end of diapause when the bees' hypopharyngeal glands increase in size to feed larvae [1]. We found that the highest *HSP70* gene expression was observed in the winter bees from Group 35, while the lowest was found in the Group control. Based on this, we

SNBL_GY 삭제됨: ,
SNBL_GY 서식 있음: 글꼴: 기울임꼴 없음
SNBL_GY 삭제됨: ,
SNBL_GY 서식 있음: 글꼴: 기울임꼴 없음
SNBL_GY 삭제됨: —were identified with high scores, with the firs being already well-known
SNBL_GY 삭제됨: honey
SNBL_GY 서식 있음: 글꼴: 기울임꼴
SNBL_GY 삭제됨: honey

65. Line 585: delete this sentence. **R65.** Deleted

66. Line 588: why, how so? **R66.**

67. Line 595: could you add some guesses why you do not find an effect if this gene has been associated to nutrition, age and season, all three factors seemingly relevant to this experiment? **R67.** Thank you for comment. We extended the explanation. Please refer to the screenshot below for convenience.

The gene *ilp1* did not show a clear effect in our experiment, as reported previously by other reseachers [76, 77], which may be due to several factors. First, the interaction of *ilp1* with nutrition, age, and season may involve more subtle or context-specific metabolic pathways that were not fully captured under our experimental conditions. Additionally, *ilp1* could be influenced by other unmeasured variables that obscure its direct effects. It is also possible that the gene's influence on these factors is non-linear or dependent on specific thresholds, meaning a wider range of experimental conditions may be required to detect its effect. Further investigation is needed to disentangle these complex relationships and better understand how ilp1 functions under varying biological contexts.

SNBL_GY 삭제됨: '
SNBL_GY 서식 있음: 글꼴: 기울임꼴
SNBL_GY 삭제됨: and requires careful consideration due to metabolic relationships influenced by nutrition, ag season [76, 77]
SNBL_GY 서식 있음: 글꼴: 기울임꼴

30

68. Line 600: all of a sudden you write about constant temperatures. I totally agree that this could be a very important factor, but this factor hardly finds any mention and discussion in the

discussion. Please add. **R68.** Thank you for comment. We corrected the mention of this point trough manuscript. Please refer to the screenshot below for convenience.

24 We simulated global warming by maintaining constant temperatures of 25°C (Group
25 25) and 35°C (Group 35) in rooms around hives from June to October, while a Group

SNBL_GY
삭제됨: climate

138 *mellifera ligustica* bees. The experiment involved maintaining colonies at ambient
139 constant temperatures of 25°C (Group 25) and 35°C (Group 35) in controlled rooms,

SNBL_GY
삭제됨: honey

550 ambient temperature (25, 35°C, and a control), was manipulated during the previous
551 summer, September, and October, and kept constant in experimental rooms. Colony

SNBL_GY
삭제됨: ,

581 Although the constant experimental ambient temperatures were set with a 10°C
582 difference, the bees' efforts to maintain optimal developmental temperatures

567 It was found that warming, along with constant diurnal and seasonal
568 temperatures, significantly weakened colony development in Groups 25 and 35
569 during the fall season (as of September 18) compared to the control group. It is

SNBL_GY
삭제됨: mass of

SNBL_GY
삭제됨: prolonged climate

SNBL_GY
삭제됨: significantly weakened col[illegible]

816 affect winter survival, possibly benefiting the longevity of winter bees. These
817 results highlight that both high (35°C) and moderate (25°C) constant temperatures
818 negatively impact colony development, reflecting broader issues related to global

SNBL_GY
서식 있음: 위 첨자

SNBL_GY
서식 있음: 위 첨자

69. Line 606: "In contrast, the 35°C temperature hindered colony development, as honey 606 bees in Group 35 were unable to reduce brood temperatures as effectively as those in 607 Group 25, impairing winter bee development. " this statement should be downworded. Since there are no measures for brood temperature in the control group, it is impossible to say whether the 35 group had suboptimal developmental conditions. Moreover the 25 and 35 group both have less brood and workers in comparison to the control. Thus in my opinion, the results do not support this strong statement and rather hint to suboptimal conditions in the 25 and 30C groups, e.g. because of constant temperatures? Maybe constant high temperatures require the bees to constantly ventilate the hive and thus use up many more resources than the control bees,

which in turn leaves them less resources for the brood? **R69.** Thank you for your comment. We agree with this discussion. We have corrected the mention of this point in the manuscript in two places. Please refer to the screenshots below for convenience.

Colony development impairment in temperatures Groups is likely due to the altered thermal environment, as honey bee colonies rely on temperature regulation for brood microclimate [64] and metabolic activity [4]. The reduced brood and worker numbers, along with potential issues in brood temperature regulation in both temperature groups, suggest that constant temperatures—whether high or moderate—had detrimental effects on colony development. This aligns with broader concerns about global warming, where constant elevated temperatures can induce thermal stress, increase the need for hive ventilation, and lead to greater resource expenditure, leaving fewer resources for brood care.

Since other colony parameters (e.g., food availability, hive size, and environmental conditions) were consistent, the results highlight the crucial role of diurnal temperature variations in supporting colony development. Persistent elevated temperatures, similar to those associated with global warming, can impair hive temperature regulation and further affect colony health and development. These findings are consistent with previous studies [66], which stress the

27

SNBL_GY
삭제됨: o

SNBL_GY
삭제됨: of

SNBL_GY
삭제됨: could be

SNBL_GY
삭제됨: related to the development of

SNBL_GY
삭제됨: and molecular markers

SNBL_GY
삭제됨: , which are responsible for the processes and longevity

SNBL_GY
서식 있음: 들여쓰기: 왼쪽 0 글자

In summary, constant temperatures of 25°C and 35°C impaired colony development in Groups 25 and 35 during the fall compared to the Group control, despite the 25°C temperature closely resembling natural conditions in Incheon. Although both temperature groups showed reduced brood and worker numbers, it is unclear if the Group 35 experienced suboptimal conditions due to the lack of direct brood temperature measurements in the Group control. The bees' efforts from Group 25 and 35 to regulate hive temperatures narrowed the difference to about 3°C, suggesting adaptive behavior. Lower brood temperatures in Group 25 did not

31

affect winter survival, possibly benefiting the longevity of winter bees. These results highlight that both high (35°C) and moderate (25°C) constant temperatures negatively impact colony development, reflecting broader issues related to global warming, such as thermal stress and resource management. Diurnal temperature variations remain crucial for optimal colony health and development.

This facilitated the development of winter bees with metabolic markers similar to

SNBL_GY
서식 있음: 위 첨자

SNBL_GY
서식 있음: 위 첨자

SNBL_GY
삭제됨: In summary, our stuc
constant day-night temperatu

70. Line 610: where are the numbers for honey bee survival? I did not see a section of measuring honey bee survival in M&M nor any results. this is actually a very good point. The authors marked 40 bees per colony, how many of those were still alive in February? **R70.** Thank you for your comment. As the honey bee colonies were kept under natural conditions during winter, the ambient daytime temperatures in February were often below 14°C (Screenshot 1), and there were instances of snow. This made it impractical to disturb the winter cluster by removing frames to count the marked bees, as exposing the bees to cold could have disrupted their ability to continue overwintering. Survival was checked in early spring (March), and the data was organized in Table S1 and mentioned in the Materials and Methods (Screenshot 2) and Discussion (Screenshot 3). We initially marked 200 bees in the fall, which was omitted in the Materials and Methods section, but we have corrected this (Screenshot 4). A sample of 40 marked bees was taken from the surface of the winter cluster for research (Screenshot 4). Please refer to the screenshots below for convenience.

Screenshot 1

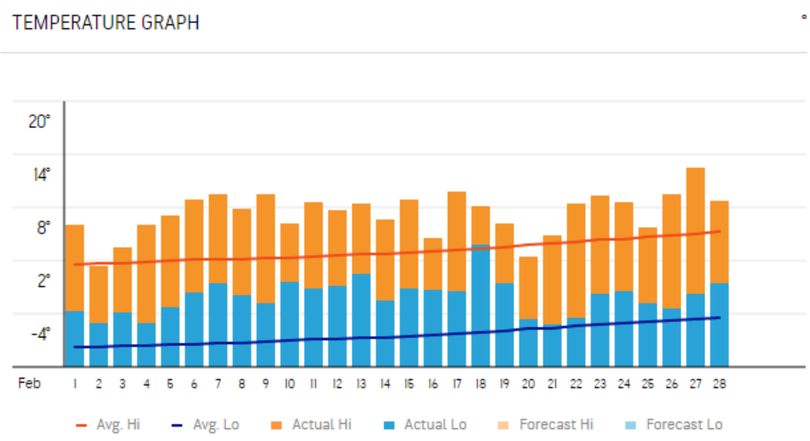

Screenshot 2. M&M

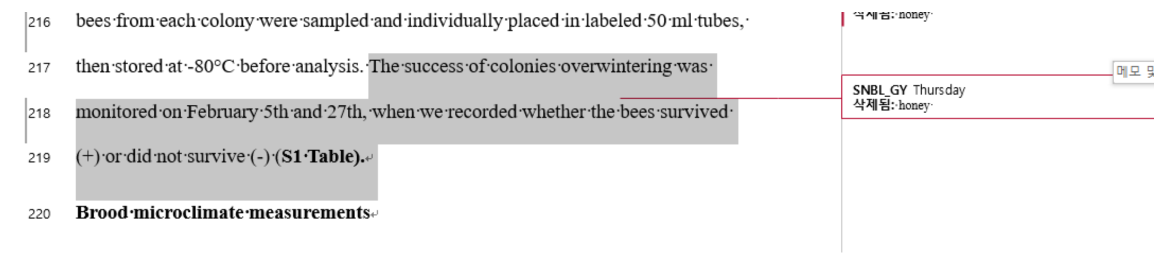

bees from each colony were sampled and individually placed in labeled 50 ml tubes, then stored at -80°C before analysis. The success of colonies overwintering was monitored on February 5th and 27th, when we recorded whether the bees survived (+) or did not survive (-) (**S1 Table**).

Brood microclimate measurements

Screenshot 3. Discussion

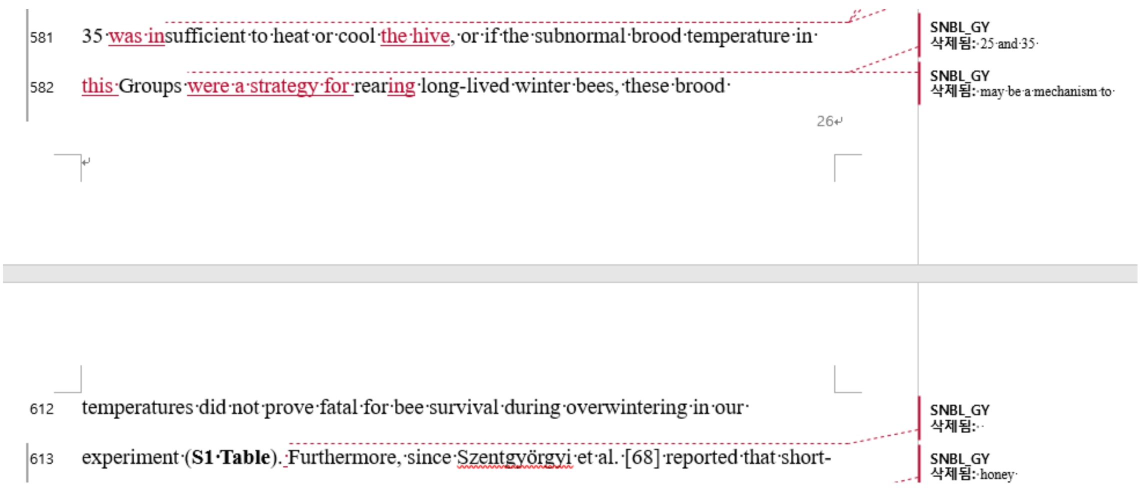

35 was insufficient to heat or cool the hive, or if the subnormal brood temperature in this Groups were a strategy for rearing long-lived winter bees, these brood

26

temperatures did not prove fatal for bee survival during overwintering in our experiment (**S1 Table**). Furthermore, since Szentgyörgyi et al. [68] reported that short-

Screenshot 4 M@M

incubator set at 34°C. Upon hatching, around 200 workers were individually marked with colored paint on their thoraxes and then reintroduced into their original colony. Throughout summer, as well as in September and October of 2022, the honey bee colonies were subjected to the according ambient temperatures. Starting from November 2022, all colonies were kept under natural conditions and successfully overwintered (**S1 Table**).

The overwintered marked bees, aged approximately 4.5 months, were sampled on February 5th, 2023, a time when queens had not yet begun laying eggs and the overwintered bees were expected to have successfully survived the winter. Forty bees from each colony were sampled and individually placed in labeled 50 ml tubes, t[illegible]red at -80°C before analysis. The success of colonies overwintering was monitored on February 5th and 27th, when we recorded whether the bees survived (+) or did not survive (-) (**S1 Table).**

SNBL_GY
삭제됨: the

SNBL_GY
삭제됨: the previous

SNBL_GY
삭제됨: varying room

SNBL_GY
삭제됨: Afterward, the controlled temperatures

SNBL_GY
삭제됨: turned off, and the bee colonies were o[illegible] successfully (**S1 Table**) in the same rooms

SNBL_GY
삭제됨: honey

SNBL_GY Thursday
삭제됨: honey

SNBL_GY
삭제됨: honey

71. Line 611: I guess you mean summer and not fall? At least you are writing about summer conditions throughout the manuscript... please be consistent. And you should be more specific and write "such as increased summer temperatures" **R71.** Thank you. We corrected. Please refer to the screenshot below for convenience.

These results can be used to correlate climate change factors, such as increased diurnal temperatures in summer and fall, with physiology responses in winter bees.

SNBL_GY
삭제됨:

SNBL_GY
삭제됨:

SNBL_GY

72. Line 613: Why "extreme heat", before it was higher temperatures...? Be consistent.
Unclear how "extreme heat" relates to management in winter? Be more specific.
What kind of management plans do you have in mind? Be more specific. **R72.** Thank you. We corrected this paragraph.

research is warranted. These findings enhance our understanding of how organisms respond to increased diurnal temperatures in summer and fall, providing valuable insights for apiculture practices and guidelines for winter management to prevent losses. Global warming during these seasons alters bee physiology for wintering, potentially threatening their longevity. Unlike bees experiencing typical fall conditions in Korea, which can adapt their colony development, those affected by global warming require assistance safeguarding their colonies at the end of winter and early spring.

SNBL_GY
삭제됨:
In summary, our study found that exposure to constant day-night temperatures of 25°C and 35°C negatively impacted colony growth in the fall. The 25°C temperature closely resembled natural fall conditions in Incheon, allowing honey bees to regulate the microclimate by lowering brood temperatures. This facilitated the development of winter bees with metabolic markers similar to those of the Group control. In contrast, the 35°C temperature hindered colony development, as honey bees in Group 35 were unable to reduce brood temperatures as effectively as those in Group 25, impairing winter bee development. In February, we observed increased levels of *JHAMT*, *TOR1*, and *ilp2*, and decreased levels of *vg*, which were associated with reduced

We are pleased to resubmit for publication the manuscript “The effect of seasonal temperatures on the aging of the overwintered honey bee”. We would like to thank the reviewers for their careful and thorough reading of this manuscript and for the thoughtful comments and constructive suggestions, which helped to improve the quality of this manuscript.

We look forward to hearing from you.

Sincerely,

Professor Hyung Wook Kwon

Department of Life Science,

Convergence Research Center for Insect Vectors

Incheon National University, R&D Complex,

265 Harmony-ro, Yeonsu-gu, Incheon, 22014, Republic of Korea;

+82-32-835-8090 (work);

+82-10-3379-6727 (mobile);

+82-32-835-0764 (fax).

Email: hwkwon@inu.ac.kr
