# Supplementary material for: The effect of seasonal temperatures on the physiology of the overwintered honey bee
Source: PLoS One. 2024 Dec 9;19(12):e0315062. doi: 10.1371/journal.pone.0315062 (PMC11627422; doi:10.1371/journal.pone.0315062)
Supplement: Attachment — Submitted filename: Response to Reviewers.pdf [file pone.0315062.s017.pdf]

Dear Editor-in-Chief, Reviewer 1 and Reviewer 2!

Thank you for the time and effort spent reviewing our manuscript number PONE-D-24-13353_R2-1 Nov (**The effect of seasonal temperatures on the physiology of the overwintered honey bee**) and suggesting very important points to consider. We corrected the manuscript according to the recommendations.

We have addressed all the points that you mark in PDF and made the necessary corrections in the manuscript.

Lines 56, 64, 70, 75, 85, 104, 112, 133, 150, 154, 157, 198, 297, Fig.3, 311, 337, 353, 354, 362, 389, 402, 440, 460, 463, 464, 482, 497, 501, 503, 505, 508, 514, 543, 561: **Response:** accepted and fixed in manuscript.
Line 475: **Response:** corrected.

All line numbers match those in the PDF from Reviewer 2. As all corrections include language improvements and we agreed with them, please note that each correction is highlighted in the file 'Reviewed Manuscript with Track Changes.doc.

Additionally, we reviewed all the references and made some adjustments.

We are pleased to resubmit for publication the manuscript. We would like to thank the reviewers for their careful and thorough reading of this manuscript and for the thoughtful comments and constructive suggestions, which helped to improve the quality of this manuscript.

We look forward to hearing from you.

Sincerely,

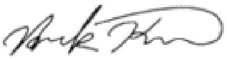

Professor Hyung Wook Kwon

College of Life Science and Bioengineering, Division of Life Science

Director of the Convergence Research Center for Insect Vectors

President of the Korean Society for Pestology and Disinfection (KSPD)

(new address, because the lab was moved to the new building): Incheon National University, R&D Complex, 265 Harmony-ro, Yeonsu-gu, Incheon, 22014, Republic of Korea

+82-32-835-0764 (fax); +82-10-3379-6727 (mobile); +82-32-835-8090 (work);

Email: hwkwon@inu.ac.kr
