# Supplementary material for: The effect of seasonal temperatures on the physiology of the overwintered honey bee
Source: PLoS One. 2024 Dec 9;19(12):e0315062. doi: 10.1371/journal.pone.0315062 (PMC11627422; doi:10.1371/journal.pone.0315062)
Supplement: Attachment — Submitted filename: Responce to reviwers rej.docx [file pone.0315062.s011.docx]

**Dear Editor-in-Chief, Reviewer 1 and Reviewer 2!**

Thank you for the time and effort spent reviewing our manuscript and suggesting some important points to consider.

Our replies are marked by **green color** after the symbol **R** (reply) and question numbers 1 (R1) – 4 (R4) for the Academic Editor, 5 (R5) – 20 (R20) for the first reviewer’s comments and (21 (R21) – 25 (R25)) for the second reviewer’s comments. Also, we copied these modifications in response for convenience when it was available.

Please, find below the reviewer comments (black) and our response (**green**)

**Academic Editor Comments**

Unfortunately,

1. the experimental design was not adequately explained throughout. For example, no plausible reason is given for the selection of the genes tested. As a result, the reader does not understand the purpose of the experiments. **R1:** Thank you for bringing this to our attention. Lines 80-99 in the Introduction provide explanations for each gene used, with the gray color highlighting the rationale behind their collective use. All of them can be seen in the screenshot below of the ORIGINAL manuscript (before current corrections).

If it doesn't seem sufficient, we revised the Introduction, Material and Method, and Discussion chapters to ensure greater clarity for readers, regardless of their level of expertise. You can find numerous corrections addressing this in the resubmitted manuscript.

2. Furthermore, it remains unclear why **no monitoring** **of the bee colonies** was carried out over the experimental period and why the **chronological age** of the overwintering bees was not stated or estimated. **R2:** We appreciate the editor's comment regarding **the monitoring of the bee colonies**. However, while the colony fitness was estimated, it was not included in the manuscript initially. Therefore, this data has been added to Figure 1 (D, E, and G). Additionally, an explanation of this data has been provided in the appropriate chapter adjacent to Figure 1.

The chronological age of the overwintering bees was mentioned, but we overlooked providing this explanation in the initial manuscript. Consequently, we have made modifications to the "Material and Methods" chapter under the section "Experimental bees". For your convenience, please find the corrections below in the screenshot.

3. In many cases, the references given are not appropriate. **R3:** Thank you for bringing this to our attention. We have rechecked each reference and made corrections where necessary. Please find the corrections throughout the entire manuscript.

4. Due to problems with English grammar and syntax, the article is difficult to read and does not correspond to standard English in its current state. **R4:** Thank you for bringing this to our attention. The English was corrected by native speakers before resubmission.

Despite all the criticism, however, some of the results seem very interesting. Therefore, you can understand my decision as an "open reject" decision. This means that if you are able to react adequately to the main points of criticism from the Reviewers, you can resubmit to PLOS One at any time. If resubmitted, the manuscript is considered a new submission. Thank you. Because of your suggestion, we resubmit to PLOS ONE.

**Reviewer #1:**

General comments.

5. There are many issues with English grammar, syntax, and scientific writing. The paper should be carefully reviewed and improved**. R5:** Thank you for bringing this to our attention. The English was corrected by native speakers before resubmission.

6. Many references are not directly related to the point in question. The authors should check carefully that suitable references are cited in the manuscript. **R6:** Thank you for bringing this to our attention. We have rechecked each reference and made corrections where necessary. Please find the corrections throughout the entire manuscript.

7. The overall experimental design needs to be adequately explained. For example: 1) Explain the rationale of the two temperatures selected. 2) Why did the authors control the temperature until October? I assume the authors want to manipulate the environmental signals before the colony starts overwintering, with the idea that these signals change the physiology of worker/ larvae and induce the subsequent development of winter bees. However, the rationale of the experiment needs to be carefully explained. **R7:** We appreciate the reviewer's comment regarding the experimental design. In response to point 1), we agree that the selection of two temperatures was omitted from the manuscript. We have now included this explanation. For your convenience, please find the corrections in the screenshot below.

In response to point 2), the reviewer's suggestion was correct; our intention was indeed to manipulate environmental signals before the colony enters the overwintering phase. We have now included this explanation in the manuscript.

8 One of the main objectives was to identify the expression patterns associated with overwintering bee physiology; the **design should have included comparisons between summer and winter bees**. **R8:** We appreciate the reviewer's comment on the experimental design. Our focus was specifically on the influence of outside temperature during the season-related transition of longevity, rather than comparing summer and winter bees. However, such a comparison could be explored in future research.

9. In addition to estimating the physiological or biological age with molecular markers, the authors should have also estimated the **chronological age** of the overwintering bees. **R9:** Thank you for bringing this to our attention. The chronological age of the overwintering bees was mentioned, but we overlooked providing this explanation in the initial manuscript. Consequently, we have made modifications to the "Material and Methods" chapter under the section "Experimental bees". For your convenience, please find the corrections below in the screenshot.

Specific points:

10. .Ln 21.“protecting nature” is a too general statement. Please use a more suitable wording. **R10:** Accepted and corrected.

11. Ln 23-26. The sentence is unclear. **R11:** Accepted and corrected.

12. Ln 27-28. Quote: ” aging was evaluated through qPCR” I suggest complete this idea, writing something similar to: “aging was evaluated through the measurement of the expression of biomarkers of age by qPCR”. **R12:** Accepted and corrected.

14. Ln 54. In addition to the reviews, the authors should cite the original studies where winter vg and JH were measured. For example, Fluri et al.., 1977 and 1982. **R14:** Accepted and corrected.

15. Ln 63 Not only in the “end of the winter”, but during the winter. **R15:** Accepted and corrected.

16. Ln-74-76. In this sentence, at least two references are not properly cited: Corona et al 2007, and Amdam and Page 2010. These articles are not related to diapause in winter bees. **R16:** Accepted and corrected.

17. Lines 77-80. I agree that, theoretically, vg should decrease, and JH should increase in the winter bees by the end of the winter. However, these changes should induce the transformation of the winter bees into spring foragers, not nurses. In any case, please add only the references directly supporting your argument: At least the three first references (Crailsheim, 1986, 1992; Nilsen et al., 2011) do not support it. **R17:** Accepted, rechecked, and corrected (citations). However, in beekeeping science and practice, the terms used to describe the overwintering condition of honey bees were not clearly defined compared to summer bees, where honey bees with different tasks and physiology were categorized into newly emerged, nurses, and foragers. When comparing summer and winter honey bees, it was found that the acinus, fat body, vg, and JH levels of winter bees were similar to those of nurses (Fluri et al., 1977). However, winter bees in diapause neither perform duties similar to those of summer nurses. Can we accurately refer to diapausing winter bees as "nurses" based solely on physiology and molecular analysis? This terminology requires further consideration and discussion. While I mentioned the duties of nurses after overwintering diapauses bees in the initial manuscript, there was no general term for this phenomenon. This issue has been addressed by refining the sentences and omitting the controversial point. The term "diapausing nurse honey bees" was used in the last edition of the manuscript.

18. Line 208-209. First, the authors should refer to relative levels (as indicated in Figure 6) rather than ddCT. Second, qPCR results are usually described, mentioning whether a comparison is significant and providing the p-value (rather than SD). I advise rewriting this section. Please compare as qPCR results are usually described in other studies. **R18:** Accepted, rewrote, and corrected

19. Line 310-322 This section needs a much better description of the colonies and how they were maintained during the experiment. For example, what was the hive size (one or two boxes)? One box What was the colony size (bee population)? Figure 1 (D, E, and H) visualized this data on new plots. Does the colonies were homogenized before the experiment? Yes. What was the age of the queen? 1 year Please also describe the rooms used in this experiment. We corrected Figure 6, and new photos and explanations in the chapter were done. For example: Were the rooms inside or outside the building in greenhouses? Could the bees fly out of the hive, or were they confined inside the hives? Could the bees fly out of the room? What were the light conditions used? **R19:** Thank you for bringing this to our attention. We revised the description of colonies to enhance clarity, and we made corrections to Figure 6 to improve the understanding of the experimental design. For your convenience, please find the corrections below in the screenshot.

20. Figure 6. The legend figure should explain the experimental design much better. For example, what was done differently between summer and fall? We corrected this point on Fig. 6D. What is the meaning of the three boxes depicted in September and October? Triplication of each treatment (three colonies). Are they colonies? What happened in November? Temperature control was off. **R20:** Thank you for bringing this to our attention. Accepted, rechecked, and corrected in the Materials and Methods chapter.

**Reviewer #2:**

The manuscript aims to investigate the impact of outside temperatures during summer on the physiological and molecular characteristics of overwintering generation of honeybee workers. The analysis was conducted in February of the subsequent year. The size of the hypopharyngeal glands and the mass of the fat body were selected as physiological markers, while the transcription of the genes *tor, vg, hsp70,* and *jhamt* are chosen as molecular markers. The objective of this study was sort of to establish a relationship between the temperature within the hives and the aging rate of the wintering generation workers, as well as the bees' capacity to endure the winter season.

21. The text lacks an explanation and justification for the selection of tested genes. The authors assert that these markers are employed for the purpose of detecting the process of aging in bees. **R21:** Thank you for bringing this to our attention. This response has already been addressed in the comment 1 to the editor above. Please find the full response there.

22. Regarding *hsp70*, I cannot concur as I am unaware of any published data regarding the function of *hsp70* as an indicator of age in bees. **R22:** We appreciate the reviewer's comment. Some published data regarding the function of *hsp70* as an indicator of age in bees can be found below. Tissue-*Hsp70* basal levels show age-related changes in mice (de Toda et al., 2016). Additionally, evidence suggests that serum concentration of *hsp70* decreases with age in a normal human population (Njemini et al., 2011). Furthermore, *Hsp70* treatment has been shown to extend mean and maximum lifespan, improve learning and memory in old animals, increase curiosity, decrease anxiety, and help maintain synaptic structures that degrade with age in mice (Bobkova et al., 2015).

Therefore, our choice of *hsp70* as the marker to test aging was not random. However, as you correctly pointed out, in honey bee science, this gene is not frequently used for aging tests.

**Reference for response:**

Njemini, R., Bautmans, I., Onyema, O.O. et al. Circulating Heat Shock Protein 70 in Health, Aging and Disease. BMC Immunol 12, 24 (2011).

Irene Martínez de Toda, Carmen Vida, Eduardo Ortega, Mónica De La Fuente, Hsp70 basal levels, a tissue marker of the rate of aging and longevity in mice, Experimental Gerontology, Volume 84, 2016,Pages 21-28.

Bobkova, N. V., Evgen'ev, M., Garbuz, D. G., Kulikov, A. M., Morozov, A., Samokhin, A., Velmeshev, D., Medvinskaya, N., Nesterova, I., Pollock, A., & Nudler, E. (2015). Exogenous Hsp70 delays senescence and improves cognitive function in aging mice. Proceedings of the National Academy of Sciences of the United States of America, 112(52), 16006–16011.

23. The authors' references on page 5 are completely irrelevant. Additionally, it should be noted that the Brejcha 2023 reference does not pertain to the facts provided. I neglected to consult further sources. **R23:** Thank you for bringing this to our attention. We have rechecked each reference and made corrections where necessary. Please find the corrections throughout the entire manuscript.

24.The transcription of the tested genes in the hive 35 exhibits notable increases or decreases as compared to the other two hives. While the data may appear intriguing first, its interpretation is very simplistic and deceptive, particularly in relation to Tor signaling. The authors make mention of alterations in the quantity of nutrients within the fat body tissue. However, I was unable to locate any data offered in the text. The only section that provides information is the description of lipid extraction in the Material and Methods section. **R24:** We appreciate the reviewer's comment. The fat body weight showed no significant differences, as presented in Figure 4A and mentioned in the chapter "Comparative Analysis of the Biological Age of Overwintered Western Honey Bees through Physiological Markers" in the initial manuscript as well. However, before these changes can become significant, certain metabolic processes must first be initiated, which we discuss in the explanation of the TOR pathway. Although significant differences in fat body weight were not reached during the 20 experimental days, molecular analysis detected some processes that had already begun.

25. Based on published data it is well known that the formation of the overwintering generation is typically influenced by photoperiod and temperature, as well as by a decrease in brooding activity. This leads to changes in the hormonal settings of bees. Furthermore, it is worth noting that winter bees do not only emerge in August, as authors sort of claim. Some sources suggest that they can be observed as early as July and continue throughout the winter, depending on brooding activities. **R25:** We appreciate the reviewer's comment. Our experiments were conducted in the Republic of Korea, and we discussed this matter with scientists who have extensive experience in beekeeping in both Europe and Asia. All of them agreed that July is not a typical month for rearing overwintered honey bees. We believe that further investigation through organizations like Coloss, where this situation can be scientifically studied across different countries, would provide valuable insights, rather than relying solely on private discussions.

Therefore, (1) some author’s claims seem to me too simplified, and also, (2) the samples of bees that were tested may not have been originated from the experimental period of June -August, when the temperature was altered. Furthermore, the authors should have to assessed the brooding activity rate and colony size throughout the experimental phase, as they are measurable criteria that may be used to determine the significant impact of temperature on the bee. Based on this, the quality of the study is not convincing to me.

We are pleased to resubmit for publication the revised version of the manuscript “**The effect of seasonal temperatures on the aging of the overwintered honey bee**”. We would like to thank the reviewers for their careful and thorough reading of this manuscript and for the thoughtful comments and constructive suggestions, which helped to improve the quality of this manuscript.

We look forward to hearing from you.

Sincerely,

Professor Hyung Wook Kwon

Department of Life Science,

Convergence Research Center for Insect Vectors

Incheon National University

119 Academy-ro, Yeonsu-gu, Incheon, 22012, Republic of Korea

+82-32-835-8090 (work);

+82-10-3379-6727 (mobile);

+82-32-835-0764 (fax).

Email: hwkwon@inu.ac.kr
