# Supplementary material for: The effect of seasonal temperatures on the physiology of the overwintered honey bee
Source: PLoS One. 2024 Dec 9;19(12):e0315062. doi: 10.1371/journal.pone.0315062 (PMC11627422; doi:10.1371/journal.pone.0315062)
Supplement: Attachment — Submitted filename: Response to Reviewers.docx [file pone.0315062.s013.docx]

Dear Editor-in-Chief, Reviewer 1 and Reviewer 2!

Thank you for the time and effort spent reviewing our manuscript number PONE-D-24-13353 (**The effect of seasonal temperatures on the aging of the overwintered honey bee**) and suggesting some important points to consider.

We corrected the manuscript according to the recommendations in the Submission Guidelines during the final step of our editing process (the Materials chapter was placed after the Introduction, citations in the text were numbered, and the References were updated). As a result, all figure numbers were changed before resubmission. However, we responded to the reviewer's comments using the previous numbering.

Our replies are marked by **green color** after the symbol **R** (reply) and **question numbers** for the reviewer’s comments. Also, we copied these modifications in response for convenience when they were available.

Please, find below the reviewer comments **(black**) and our response (**green**)

**Reviewer #1: Comments on PLOS ONE ms. “Winter honey bees”**

This manuscript describes an experiment designed to determine how elevated brood nest temperature affects the physiology and gene expression of wintering honey bees. I think the design of the experiment and statistical analyses were appropriate. However, I have substantial concerns about some of the wording and failure to address some of the implications of the results. Below I highlight some of my most important concerns. I have placed MANY comments and suggested changes to wording on the manuscript itself.
Despite my many comments, I enjoyed the study. I feel that the authors need to fix some of the glaring issues (i.e. HP glands are relatively undeveloped in wintering bees, not hypertrophied/well developed, as was mentioned at least 3 times), improve the wording, and better discuss the implications of their results with respect to our warming planet.

1. I anticipated that this study would lead to a discussion of how climate change, and specifically warming temperatures, may affect wintering bees. You mention this briefly in the last sentence of the Introduction. Therefore, I thought this would be the main point brought out in the Discussion. Instead, the Introduction and Discussion largely ignored the possible effects of climate warming on individual wintering bees. Why? **R1.** Thank you for pointing this out. We corrected some parts of the Introduction and Discussion.

Climate change is a complex set of factors that influences honey bee survival through temperature and the amount and quality of melliferous plants, pest and pathogen development, etc. Notably, the season of appearance and the duration of cooling or warming will have different impacts on honey bees and other plants and animals. This topic deserves several dedicated articles in the future.

Our field experiment focused on only one aspect of climate change: warming, characterized by stable, long-term high temperatures during the reported months, including summer. The effect on the overwintering of honey bees was estimated, as overwinter mortality continuously decreases the honey bee population worldwide. This experimental challenge was conducted solely on honey bee colonies to study their response, excluding the effects on plants and other environmental factors outside the rooms. For your convenience, you can review the corrections in the screenshots below.

Screenshot 1 (Introduction)

Screenshort 2 (Introduction)

Screenshot 3 (Results)

Screenshot 4 (Discussion)

Screenshot 5 (Material and Method)

2. Lines 126-132 verbally describe the ambient temperatures for the three experimental groups. **A)** The Control and 25-degree colonies experienced similar temperatures, but experienced significantly lower temperatures than the colonies in the room kept at 35 degrees. **B)** The authors refer to Fig. 6E for these results—but there is no Fig. 6E. Moreover, Fig. 6E is referred to before Fig 1 in the text—figures must be referred to sequentially (1, 2, 3, …). **C**) Related to this, the next paragraph describes how the brood nest temperature was significantly elevated in the 35 degree group compared to the 25 degree group. However, lines 141-149 of the Results fails to mention the control group! Figs. 1A 1B, 1C should include the control group. **D)** Figure 1E, that compares the numbers of capped brood cells over time, is therefore an enigma—as the 25 and 35 degree groups have similar numbers, but different from the Control, but the Control and 25 degree groups experienced similar temperatures in their experimental settings (except in the month of October). This odd result is never mentioned or discussed. **R2.** Thank you for this questions. We divided the points for the response into A) ~ D) for convenience. **A)** The difference in temperatures for the honey bees from the Control group and the 25°C group was due to day-night fluctuations, which were absent in the latter group. On average, significant temperature differences were not detected. However, a stable elevated night temperature is one of the factors of climate warming that was assessed in this research. We added this explanation to the paragraph “Dynamics of ambient temperature in summer and fall months”. **B)** This concerns the experimental design (Fig. 6E), which is detailed in the Materials and Methods section, following the Results section according to PLOS ONE guidelines. Due to the complexity of this numbering, we have removed this reference. **C)** Because of ambient temperature and colony performance (Fig 1 D, E, F) of honey bees from Group control and 25 at the beginning of the measurement (22.08.08) had no significant differences, sensors were not set up in honey bee colonies from Group control. Corrected in manuscript (see Screenshot). **D)** Honey bees, as social insects, have mechanisms for warming or cooling their nest to regulate the temperature around the brood, reducing the effect of ambient temperature on brood rearing. Despite the different ambient temperatures, similar numbers of capped brood areas in honey bee Groups control, 25 and 35 were observed in August and September 8. This indicates successful brood temperature regulation for colony development by the bees until September 18, when hidden problems from the summer were revealed through comparison with the control group. The only factor of climate warming considered here is temperature. Continuously stable ambient temperatures of 25°C and 35°C over 3 months suggest stress due to the lack of day-night temperature fluctuations and wind-cooling effects. This could weaken the colony in the fall, but it might not be a significant problem in the summer if food is sufficient. We added clarification to the Results.

3. Line 48-49: You state: “it is necessary to elucidate the processes involved
in the transition from short-lived workers to long-lived ones.” This is a valid point, but it is not the focus of your research! Therefore, it creates an expectation in the reader of what you investigated, but later you explain that you are really interested in factors operating in fall that affect the longevity of workers during winter. **R3.** Thank you for this question. Corrected.

4**.** It is not clear why you chose to capitalize “Acinus” and “Fat body” throughout the manuscript. I see no reason to do so. **R4.** Thank you for this question. Corrected.

5. The use of the term “honey bee” throughout the paper. It is clear from the Introduction (lines 103-104) of the paper that you studied honey bees, Apis mellifera. You may want to state that again at the beginning of the Methods and Results, but in many many locations you can replace “honey bee” with “bee” or “bees”, or even in some places to reduce repetition with “it” or “them” (singular/plural). **R5.** Thank you for this question. Corrected.

6. “Acinus” is singular. In honey bees, an acinus is a single globular structure of the HP gland. The hypopharyngeal gland is composed of many grape-line “acini”- plural. You need to check through the paper to determine which mentions of “Acinus” should remain “acinus” (singular), and which mentions refer to multiple structures and should be referred to as “acini” (plural). **R6**. Thank you for this question. Corrected.

7. Line 63-64: The authors have written that the winter bee phenotype is characterized by “increased Acinus size (hypopharyngeal gland, HP)”. This statement is false. Refer to Fig. 2 in Doke et al., a paper you cited. Similar incorrect statements about larger HP glands in winter bees appears in Line 249 and Line 256.
You may also want to cite:
Wang K., Liu Z.-G., Pang Q., Zhang W.-W., Chen X.-M., Fan R.-L., et al. (2018). Investigating the regulation of hypopharyngeal gland activity in honeybees (Apis mellifera carnica) under overwintering conditions via morphologic analysis combined with iTRAQ-Based comparative proteomics. Ann. Entomol. Soc. America 111 (3), 127–135. 10.1093/aesa/say012 **R7.** Thank you for this question. We fixed this inaccuracy and changed "increased" to "hypertrophied" (as in Reference 1) (see in Screenshot 1, 2 and 3), because it used in literature (Knoll et al., 2020). The measurement of the hypopharyngeal gland (HG) includes different parameters, such as the morphology of the HG (area, diameter, width, and length of acini) and the ultrastructure (development of secretory vesicles inside acini). In the HG of winter bees (sampled in November), secretory vesicles were visible, similar to those in 3-day-old bees, which were absent in newly emerged summer bees (Deseyn and Billen, 2005, Reference 2). They also found that the diameter of the acini in winter bees falls within the range of that in 3-day-old bees and was higher than in forager bees. Therefore, the term "hypertrophied" is more appropriate than "increased". As recommended in the article you suggested, Wang et al. (2018) reported the transition of HG development at the end of winter through ultrastructure research (development of secretory granules) by comparing honey bees sampled on February 15th and January 8th, without including data on summer honey bees. Consequently, this gland remains hypertrophied, as described by Cruz-Landim and Hadek (1969). However, the diameter of the acini in winter bees is not as large as in 6-12-day-old nurse summer bees (Deseyn and Billen, 2005).

Reference1

Knoll S, Pinna W, Varcasia A, Scala A, Cappa MG. The honey bee (*Apis mellifera* L., 1758) and the seasonal adaptation of productions. Highlights on summer to winter transition and back to summer metabolic activity. A review. *Livest Sci*. 2020; 235: 104011.

Reference 2

Deseyn J., Billen J. 2005. Age-dependent morphology and ultrastructure of the hypopharyngeal gland of *Apis mellifera* workers (Hymenoptera, Apidae). *Apidologie* 36: 49-57.

Screenshot 1: correction done

Screenshot 2 (line 249 in initial manuscript): the comparison is relevant to our research, where one area is larger than the other.

Screenshot 3 (line 256 in initial manuscript): we explained this point of view more deeply because it is a citation.

8. Lines 116-121: I feel that these sentences provide too much of the results. In the Introduction it is OK to briefly mention what the results are, in general terms, but this level of explanation of results seems excessive. **R8.** Thank you for this question. We reduced the explanation of the results there.

9. Statistical reporting: I have always referred to “Duncan’s post hoc test”. Not “Duncan”. I did an internet search and several sites also refer use the possessive noun: “Duncan’s”.
It is good to explain that SD refer to Standard Deviation the first time you use it in the text and in a figure legend. I do not think you need to write out “SD - Standard Deviation” repeatedly in the text and figure legends**. R9.** Thank you for this question. Corrected.

10. Number of significant digits to report: There are rules for how many significant digits one should report. It depends on the accuracy of the measurements as well as the number of possible values for the variable. Reporting mean temperature as 29.995 ± 0.982 °C is not correct. This should be either 30.0 °C or 30.00 °C. Please seek advice on significant digits and then check and correct all values reported in the manuscript. **R10.** Thank you for this question. We checked the SAMPL guidelines for general guidance on statistical reporting in PLOS ONE (screenshot attached). We corrected the issue. We used the 30.00 °C format in this chapter, with a total of 4 digits.

11. Check the definitions of “weight” vs. “mass”. I believe you will find that you should refer to “fat body mass”**. R11.** Thank you for this question. Corrected.

12. Lines 152-154: Interpretations of data belong in the Discussion—not in the Results. **R12.** Thank you for this question. Corrected.

13. Line 155: Heading: “Molecular marker recognition in overwintered western honey bees”. I believe you determined “gene expression of molecular markers” And acinus size and fat body mass are not “molecular markers”. You need to reword this heading. **R13.** Thank you for this question. Corrected. For convenience, you can review the corrections on the screenshot below

14. Figure 2: Because red-green color blindness is very common, particularly in men of European descent, one should never use red and green together in figures. This red and green graph will NOT be comprehensible by a number of readers. A different color scheme is needed. **R14.** Thank you for your question. Since this disability can affect the perception of red, green, and blue, we used yellow and blue scales to redraw the figure. We also corrected Figure 1 by changing the red line to a dashed line. For convenience, you can review the corrections in the screenshot below.

15. Line 247: You state that the bees in Groups 25 and 35 successfully overwintered. I have seen no data in the manuscript that supports that statement. There had to be some bee mortality over the 3.5 months. What percentage of marked bees survived to the date when they were sampled? Did you collect that data? (You may be referring here to colony survival, but that is not as relevant to your study as individual bee survival.) **R15.** Thank you for this question. Survival of honey bee colonies estimated as overwintering loss (YES\NO) in this case at the end of February and their developmental problem in spring (YES\NO). We added this table to the Supplementary material (S1 Table). All colonies got the “NO” that we reported in the manuscript because our target problem overwintering loss. Counting the percentage of marked bees in the middle and end of February under field conditions is not possible due to the low ambient temperatures in Korea, which can cause colonies to overcool and die. For convenience, you can review the corrections on the screenshot below

16. Lines 247-248: I have placed two important comments on the manuscript related to this sentence. **R16.** Thank you for this question. We corrected this part of discussion.

**Reviewer #2: Review "The effect of seasonal temperatures on the aging of the overwintered honey bee"**

Frunze et al. investigate the effect of winter temperature experienced by larvae on aging markers in workers. To do so, hives are kept at two different temperatures plus a control treatment (natural conditions) over summer and fall. In winter workers of the hives of the different treatments are collected, fatbody size, acinus size and the expression of several aging associated candidate genes are investigated to make inferences on the putative effect of climate warming on aging in winter bees. The aim and topic of this experiment are highly relevant and interesting. At the moment, there are several parts and aspects that need clarification and correction. The authors should also discuss the interplay and the meaning of the various factors, from treatment to measured variables, more clearly.

Major comments
17. -I understand that you collected several candidate genes that have been shown to be associated with aging and you test these in the fatbody. It does not become clear in the discussion whether all these candidates change their expression in the expected direction. Do they differ and if yes how, and what is your interpretation of that? **R17.** Thank you for this question. We added more discussion about this.

18. -Why did you choose to investigate Vg gene expression in the fatbody, and the expression of all other candidates in the head? **R18.** Thank you for this question. We corrected this part of the Introduction. Please, check lines 122-124, 743-745 on the screenshots below.

19. -Why did you measure fatbody and acinus size?
=> the latter three points could be made clear in the introduction, but latest in the results **R19.** Thank you for this question. We corrected this part of the Introduction. Please, check lines 114-116 on the screenshots below.

Screenshot

20. -What was the mean temperature of the control treatment in the hive, and how does it relate to the two treatment temperatures? **R20**. Thank you for this question. I believe you are referring to ambient temperature. The plot showing the dynamics of ambient temperatures in control and experimental colonies is presented in Figure 6E. Below on screenshot, we explained why these temperatures were chosen.

21. -The treatment temperatures differ by 10C, however, the in-hive difference is only 3C. This should be mentioned and discussed somewhere, also in respect to the control. This is especially relevant to be able to interpret why gene expression was only different to the 35C treatment. **R21.** Thank you for this question. Corrected.

22. -I find it highly interesting that the variables measured for Fig1D-F differ between the control and both treatments but later gene expression differs between 35C and the other two. Add this finding to the discussion and add your explanation for this pattern. **R22.** Thank you for this question. We added this points in Discussion.

23.-Results of the statistical test need to be better depicted and also mentioned in full in the text. **R23.** Thank you for this question. Corrected.

See more details below.


Minor comments

 **Abstract**
24. rephrase the first sentence. **R24.** We appreciate the reviewer's valuable comments and have rephrased the first sentence of the Abstract. For your convenience, you can review the corrected lines (21–23) in the screenshot below in the next response, R25.

25. Line 23: Be more concise. Temperature is not the only factor reported to affect winter bee longevity, it would thus be good to at least write that it is ONE factor affecting longevity and thereby that other factors may also play a role. Or also mention other factors putatively affecting longevity in bee workers. Be more specific in the abstract on what was in the treatment and who was tested later. **R25.** Thank you for bringing this to our attention. We fixed this inaccuracy in the sentence (lines 23 – 25). For convenience, you can review the corrections on the screenshot below

**Introduction**
26. Line 41: maybe put emphasis first on their role as pollinators and honey production and then production of health and medical products. **R26.** We appreciate the reviewer's valuable comments and have rephrased the first sentence of the Introduction. For your convenience, you can review the corrected lines (47–49) in the screenshot below.

27. Line 45: be more specific and add information on the "various factors" that may contribute to winter mortality. Plus maybe indicate that there are factors that contribute to strong or abnormal winter mortality. Because even under normal conditions winter bees will die and be replaced by spring workers...**R27.** Thank you for bringing this to our attention. Included to Introduction

28. Line 53: delete "seems" **R28.** Thank you, we deleted this word.

29. Line 66/67: what do you mean with "unlike"? Do other insects need diet at all or other dietary composition? **R29.** Thank you for pointing this out. Winter activity is a distinguishing feature of diapause honey bees compared to wasps, bumblebees (eusocial insects where only the queen overwinters), and other non-social insects that overwinter in the adult stage. Usually, diapause means metabolic suppression and insects (excluding honey bees) do not need to move or eat until spring (Denlinger, 2023). This is a special topic for discussion, and we do not have space to deeply explore diapause in insects generally. We have added some corrections to sentences for readers to easily follow the research. For your convenience, you can review the additions in the screenshot below.

Denlinger DL. Insect diapause: from a rich history to an exciting future. J Exp Biol. 2023; 226 (4): jeb245329.

30. Line 73/74: "Acinus" and "Fat" should be written in lower letters**.** **R30**. Thank you for this question. Corrected.

31. Line 77: what do you mean by "behavioral activities"? Why "inevitably" leading to reduced longevity and increased aging? **R31**. Thank you for this question. We believe "behavior" is a better term than "behavior activity," and "inevitably" was a superfluous word that was deleted during the correction. We added explanation briefly in six lines before this summarized sentence. Additionally, we avoided repetition and didn't extend the explanation to maintain the focus on the idea of the research, as this information is well-described for honey bees (Prado et al., 2020).

**Reference for response:**

Prado A, Requier F, Crauser D, Le Conte Y, Bretagnolle V, Alaux C. 2020 Honeybee lifespan: the critical role of pre-foraging stage. R. Soc. Open Sci. 7: 200998.

32. Please also add information on the roles and functions of JH and Vg as you do for the other candidate genes. **R32.** Thank you for this question. Added. For your convenience, you can review the additions in the screenshot below.

Introduction

Discussion

33. Line 96: "longevity" please be careful with using aging and longevity interchangeably. Longevity is basically the end result of the aging process; they are highly related but not the same. **R33.** Thank you for this question. We rechecked the manuscript for any mistakes in meaning, as we intended the same like you meaning for these words.

34. Line 102: you might want to add some information on why and how temperature may affect lifespan**. R34.** Thank you for this question. We have added this sentence to lines 57–60 and included the cited articles in the References. For your convenience, you can review the additions in the screenshot below.

35.Line 107: What exactly does "maintain" mean? Was the control group located outdoors or were outdoor conditions "simulated". **R35.** Thank you for this question. This sentence has been rephrased, and the word “maintain” has been deleted. For your convenience, you can review the correction in the screenshot below.

36.Line 117: "winter worker broods" unclear whether you investigated workers or brood. So far I understood that the aim was to investigate workers but here it sounds like it was brood? But then for Group35 you only talk about workers... Please phrase more clearly. => after reading the manuscript I now understand what you mean and it makes sense. Maybe consider writing "brood resulting in winter workers" or something like that? **R36.** Thank you for this question. These words (winter worker brood) have been replaced (brood resulting in winter workers) for clear meaning. For your convenience, you can review the correction in the screenshot below.

37. Line 120: HSP70 and TOR1 may serve as additional markers for what? Please clarify. And what kind of research is additionally needed? **R37.** Thank you for this question. We have added clarification to the sentence about this. For your convenience, you can review the correction in the screenshot below.

38. Line 122: I assume you hint towards faster aging under higher temperatures, but this is nowhere clearly stated. Please add this information and make the relationship easier understandable for the readers. **R38.** Thank you for this question. Added in Discussion.

Results

39. since M&M comes at the end of the manuscript it would be helpful to get some additional information in the results already. E.g. at which stage you put the frames of which staged brood in the experimental conditions, you marked workers and those were tested later, **R39**. Thank you for your question. We apologize for the oversight. According to PLOS ONE guidelines, the Materials and Methods chapter should precede the Results. We have corrected this, and the logical flow is now restored.

40. Line 128 and all following statistical results: please add test statistics in addition to the p-value. Currently unclear which test you conducted and its effect size. **R40.** Thank you for this question. We have addressed this omission and added the name of the statistical method to the text of the manuscript where it was used. For your convenience, you can review the corrections in the screenshot below.

41. Line 132 and 312: Why do you start with Figure 6? This should be Figure 1**. R41.** Thank you for this question. This concerns the experimental design (Fig. 6E), which is detailed in the Materials and Methods section, following the Results section according to PLOS ONE guidelines. Due to the complexity of this numbering, we have removed this reference.

42. A) Line 142: 29.995 ± 0.982 and 33.398 => the difference for the brood between the groups was only 3C instead of the 10C of the chambers. This should be mentioned much earlier. It basically means that the bees heat up the brood area to ~30C. Does 33C mean that the bees cool down the nest in the 35C treatment or are discrepancies in the ways nest and room temperatures are measured? B) What were the according measures in the control group? **R42.** A) Thank you for this question. It is well-known that honey bees maintain a consistent brood microclimate (Abou-Shaara et al., 2017; Bencsik et al., 2023) through cooling or heating, so a small difference in brood temperature of 3°C was expected even though the difference in ambient temperature was 10°C. The normal temperature range within a honey bee colony is from 33 to 36°C (Kovac et al., 2009) during spring and summer, with a more precise range of 34.5 ± 1.5°C (Jones et al., 2005), as mentioned in the Discussion. Additionally, Szentgyörgyi et al. (2018) reported that workers and drones developing at 32°C live longer than those developing at 35°C in the spring generation of bees. We observed lower brood temperatures in August and September, which is suggested to be related to the longer lifespan of honey bees reared for overwintering. B**)** We added this important point to the manuscript. Please, check the screenshot for convenience.

43. Line 156++: "The molecular markers recognition related to the biological age of long-lived honey bees, which were approximately 4.5 months old, was verified through a dataset comprising": sorry but I don't understand what you want to say here... E.g. what do the molecular markers "recognize"? And what was verified? **R43.** Thank you for this question. We have concisely rephrased the subtitle of this paragraph and the first sentence. In summary, comparing well-known and not-yet-studied markers allows us to analyze their relationship with aging. The word “verify” did not express idea the of “analyze the relationship” and has been replaced. For your convenience, you can review the corrections in the screenshot below.

44. Line 168:"A comparison revealed significant differences between Group 25 and Group 35" Please be more specific. What kind of comparison, which statistics are you talking about here?"**R44.** Thank you for this question. This paragraph describes the results of the Discriminant Analysis using Wilks' Lambda, which measures how well the discriminant functions differentiate between the groups. You can see this data in Screenshot 1 of the Excel sheet. Additionally, we have added the name of the statistic before the p-value. For your convenience, you can review the corrections in Screenshot 2 below.

Screenshot 1

Screenshot 2

45. Line 169: How many individuals were tested per colony. Based on Fig 2B I assume that 3 colonies per treatment were tested? One individual per colony or multiple? Please also add this information somewhere in the results, to give an idea of sample size. It would also be more telling to plot all the data points that went in the PCA (Fig 2A), which would also give an impression on the variation between samples. **R45.** Thank you for this question. We modified the title of Figure 2 by adding an explanation about used variables and sample size (Screenshot 1 below). Also, we created Figure 7, which includes the sample size for each data set (Screenshot 2 below).

Screenshot 1

Screenshot 2

46. Line 174+175: Please correct the interpretation of the DA results here: 99.98% is the percent variation explained along this axis. You could check based on the factor loadings which of the factors is the most telling here. How does this figure indicate that gene expression of ... contribute to this separation? Please clarify. **R46.** Thank you for this question. We sorry and fixed this inaccuracy in the text of manuscript and changed 99.98% to 99.92% as pointed in Figure 2A (Screenshot 1). When a higher Fisher's distance indicates better separation between the groups, the **discriminant function coefficients** contribute the value of each variable to this separation in XLSTAT soft**.** You can see these coefficients in the table in the Screenshot 2 below. Discriminant function coefficient in DA has the same meaning as factor loadings in Factor Analysis.

Screenshot 1

Screenshot 2

47. Fig3: maybe move panel A to supplement. Panel B, add "fatbody size" to header.
Currently it is not clear what exactly you are testing. I.e. instead of "dependent variable" specify that you are testing which of the gene expression levels correlates with fatbody and acinus size. Also add that you are testing. **R47**. Thank you for this question. We accepted the correction and removed Figure 3A, renaming Figure 3B to 3A and 3C to 3B. Additionally, we corrected "Fat body" to "Fat body weight." We also rewrote the footnotes of Figure 3 to include more details. For your convenience, you can review the corrections in the screenshot below.

48. I am actually wondering why you are conducting this analysis at all? If I understand correctly you are testing which of the variables, i.e. the expression of the different genes affects fatbody or acinus size, and whether fatbody size affects acinus size and vise versa. A) do you really expect that there is a causal relationship? B) I thought that the main aim was to test whether there are gene expression differences of these candidate genes between the different treatment groups, i.e. the results given in Fig 5. **R48.** Thank you for this question. Elastic Net regression, Discriminant Analysis, Agglomerative Hierarchical Clustering, and Correlation Analysis are part of Data mining. They are used to analyze large datasets to uncover patterns, relationships, and insights that cannot be found using only comparative analysis of variables one by one.

The strategy for choosing these methods is described in the Materials and Methods chapter. Briefly: (a) **Discriminant Analysis** (DA) classifies the marker dataset to find a combination of features that best separates groups. (b) To discover natural groupings in the absence of labeled information, **Agglomerative Hierarchical Clustering** (AHC) was used. (c) **Elastic Net Regression** models the relationship between the target variable and all other variables to rank them by importance for the target. (d) Moreover, **Correlation Analysis** is a fundamental tool in Data mining used to identify relationships between variables.

Addressing specific points: A) **Causal Relationships:** While these methods do not establish causality, they help identify potential relationships and patterns within the data. The goal is not necessarily to demonstrate a causal link but to explore the potential connections and variables that warrant further investigation. B) **Main Aim:** The primary aim is indeed to test gene expression differences between treatment groups, as illustrated in Figure 5. The additional analyses are intended to explore the relationships between gene expression, fat body size, and acinus size in greater depth. These analyses help to understand whether and how these variables might interact, which can provide a more comprehensive view of the data and guide future research directions.

In summary, while these methods may predict possible relationships, they are tools to broaden our understanding and to build hypotheses for future experiments on aging.

49. Fig 4: again it would be better to depict the data as boxplot with single data points visible.
Line 207: change to "physiological aging" **R49.** Thank you for this question. We corrected the mistyping in line 207. We aim to use a consistent plot style throughout the manuscript whenever possible, and we prefer to keep the same plot. The sample size, which you suggested be indicated with individual data points on another plot, is noted in the footnotes of the plots in Figure 4.

50. Line 209: I understand what you mean with "physiological age" above, and it makes sense. Here I am lost what you mean by "biological age"? Is this also supposed to mean "physiological age", because based on M&M all the tested workers should be 4.5 months old and age matched, right? **R50.** Thank you for this question. The terms "physiological age" and "biological age" are used interchangeably, as both express the of metabolic processes which speed and quality can be different at the person with same chronological age.

51. Fig5: which measure for "relative expression level" did you use, deltaCT? Add information to Y-axes. Add measures to the Y-axes. Again depict results as boxplots with data points. **R51.** Thank you for this missing clarification. Yes, the above-mentioned "relative expression" originates from the Ct data, which is used to calculate the fold differences between a target gene and a reference gene (delta Ct), and later, between a certain condition and control (2^−ΔΔCT^) (Livak and Schmittgen, 2001) (Screenshot 1). In the original article, the Y-axis is labeled as "Fold change in gene expression" (please see Fig. 3 in Screenshot 2), and we suggest using this instead of "Relative expression". For your convenience, you can review the corrections in the Screenshot 3 below.

Screenshot 1	Screenshot 2

Screenshot 3

52. General comment in respect to results. P-values are mostly > or <0.05. "<0.05 means between 0.01-0.05, is this always the case, are p-values always in this range? **R52.** Thank you for this question. Our reporting follows the standard scientific practice of providing more precise p-values when necessary to convey the statistical significance more accurately. Therefore, while some p-values might be reported as "<0.05", others are reported with more precision if they fall below commonly used thresholds (e.g., 0.01, 0.001). In summary, "<0.05" means the *p*-value is less than 0.05, but not necessarily restricted to the range of 0.01 to 0.05. We ensure to report *p*-values with appropriate precision to reflect the significance of the results accurately.

**Discussion**

53. Line 234: "during the previous summer" does this mean "during larval development"? Maybe specify this information for better understanding. **R53.** Thank you for this question. Honey bee colony development in spring, summer, and fall includes stages of open brood (eggs and larvae) and capped brood (pre-pupae and pupae), which are cared for by younger nurse-stage adults. Food carrying is provided by older adults in the forager stage, with the amount and numbers adjusted according to weather and colony conditions. When I mention "during the previous summer", I am referring to the influence of ambient temperature on the entire colony, including the larval stage, and how this necessitates a colony response to survive under experimental conditions and prepare for overwintering in the late summer and fall. Since 'during the previous summer' encompasses the meaning of 'during larval development,' we prefer to use the former expression.

54. Line 243: you need to give the actual mean temperatures here as well, not just the difference. Otherwise readers might not know and/or remember that they had 30 C. **R54.** Thank you for this question. We fixed it. For your convenience, you can review the corrections in the screenshot below.

55. Line 248: what do you mean by "initially had to decrease the nest temperature"? It sounds like both groups had to...? But the 25 C group actually needs to increase temperature to reach 30C. **R55.** Thank you for this question. We need to address some misunderstandings. We believed that “in nature” more clearly emphasized the natural condition as opposed to “initially” (i.e., without experiments) and made this change. We mean that the need to “decrease the nest temperature” is not yet a well-known adaptation for rearing long-lived honey bees, especially compared to the well-documented nest microclimate temperature in spring/ summer found in other research and in late summer/ early fall in our experiment with Group 35. It requires additional field experiments to deepen our understanding of the transformation related to longevity of honey bees.

56. Please clarify that Vg and JH expression are connected. Which way and in which bees/castes should become clearer and to what extent does it fit to your results...**R56.** Thank you for this question. Added to Introduction and Discussion.

57. Line 276: "relationship between vg gene expression and the hypopharyngeal gland (HG) and Fat body" I neither see anything about the hypopharyngeal gland in Fig3, nor anywhere in the results, nor any relationship of this gland and Vg and the fatbody. **R57.** Thank you for this question. We added this clarification. You can review the corrections in the screenshot below for your convenience.

58. Line 282: " it is associated with honey bee transitions in summer and aging" not sure what you mean here? Please be more specific and clarify. **R58.** Thank you for this question. We rewrite this sentence and related following discussion. For your convenience, you can review the corrections in the screenshot below.

59. Line 286: "The subsequent gene, TOR1, wasn't selected by Data Mining methods but can be considered aging-related (Barth et al., 2010; Fahrbach et al., 2012). " This statement needs to be reformulated. You do not find an association of TOR1 expression with physiological aging in your study. There are other studies which do. What could be the difference why they find it you don't? **R59.** Thank you for this question. We rewrite this discussion part. For your convenience, you can review the corrections in the screenshot below.

60. Line 288: "explaining the decrease in Fat body " this is not correct. You find a correlation of both not a causal relationship, please reformulate. **R60.** Thank you for this question. We rewrite this discussion part. For your convenience, you can review the corrections in the screenshot below.

61. Line 293: this summary sentence is rather confusing. **R61.** Thank you for this point. We rewrote this summary. For your convenience, you can review this in the screenshot below.

62 Line 295: why "conversely"? **R62.** Thank you for this point. We rewrote this and the "conversely" word was deleted. For your convenience, you can review this in the screenshot above in R61.

63. Did you continue observing those colonies? Did the differences in the 35C group lead to lower survival of the colonies, smaller colony sizes,... any fitness differences? **R63.** Thank you for this question. After detecting the overwintering success in mid-February, these colonies were given nutritional stimulation and involved in other experiments, making continued observation of the colonies impossible.

64. Figure 6D: change "no regulated" to "not regulated" **R64.** Thank you for this question. We corrected. Please, check Figure 6D.

65. Line 357: change "whether differed" to "whether it differed" **R65**. Thank you for this question. We corrected.

66. Line 372: "name, company, country" add according information
Are the results of the lipid content given in the results, or somewhere in the manuscript? => either add results or remove information from M&M **R66.** Thank you for your question. We apologize for the typographical error. We have added “name, company, country”. In this research, a method was used to analyze lipids in the fat body, as it is the most prominent component. This clarification has been added to the abstract and elsewhere in the manuscript. For your convenience, you can review this in the screenshots below.

Abstract:

Introduction:

Discussion:

Also, corrections were made in the Results chapter as well.

67. Line 377: add information on how and when the tissue(s) were dissected. Before freezing at -80C? Were they dissected and then directly isolated? Were samples dissected on ice? **R67.** Thank you for bringing up this point. We have clarified this step of the experiment. For your convenience, a screenshot is provided below for your review.

68. Line 435: change "group" to "groups"
Please indicate which tests were conducted using excel. **R68.** Thank you for bringing up this point. We have fixed the missing letter (Screenshot 1). This paragraph describes the results of the Discriminant Analysis (DA), which was fully spelled out in the paragraph above Figure 7 (Screenshot 2). For your convenience, a screenshots are provided below for your review.

Screenshot 1

Screenshot 2

69. Line 460: there should be additional supplementary tables with the raw data of all measures/data reported on. **R69**. Corrected.

PONE-D-24-13353

PLOS ONE

**We included when submitting your revised manuscript:**

•	A rebuttal letter that responds to each point raised by the academic editor and reviewer(s). You should upload this letter as a separate file labeled **'Response to Reviewers'**.

•	A marked-up copy of your manuscript that highlights changes made to the original version. You should upload this as a separate file labeled **'Revised Manuscript with Track Changes**'.

•	An unmarked version of your revised paper without tracked changes. You should upload this as a separate file labeled '**Manuscript**'.

If you would like to make changes to your financial disclosure, please include your updated statement in your cover letter. Guidelines for resubmitting your figure files are available below the reviewer comments at the end of this letter.

**When submitting your revision, we need you to address these additional requirements.**

1. Please ensure that your manuscript meets PLOS ONE's style requirements, including those **for file naming**. The PLOS ONE style templates can be found at

https://journals.plos.org/plosone/s/file?id=wjVg/PLOSOne_formatting_sample_main_body.pdf and

<https://journals.plos.org/plosone/s/file?id=ba62/PLOSOne_formatting_sample_title_authors_affiliations.pdf> **1R: Corrected**

2. We note that **the grant information** you provided in the ‘Funding Information’ and ‘Financial Disclosure’ sections do **not match**.

When you resubmit, please ensure that you provide the correct grant numbers for the awards you received for your study in the ‘Funding Information’ section. **2R: during resubmission will do**

3. Thank you for **stating the following financial disclosure**:

"Yes

Hyung Wook Kwon received.

This work was carried out with the support of the Cooperative Research Program for Agriculture Science & Technology Development (Project No. RS-2023-00232749) and the Priority Research Centers Program through the National Research Foundation of Korea (NRF) funded by the Ministry of Education (2020R1A6A1A03041954)."

Please state what role the funders took in the study. If the funders had no role, please state: "The funders had no role in study design, data collection and analysis, decision to publish, or preparation of the manuscript."

If this statement is not correct you must amend it as needed.

Please include this amended Role of Funder statement in your cover letter; we will change the online submission form on your behalf. **3R: Sorry, “no” is correct. We added the sentence in Manuscript "The funders had no role in study design, data collection and analysis, decision to publish, or preparation of the manuscript."**

We are pleased to resubmit for publication the manuscript “The effect of seasonal temperatures on the aging of the overwintered honey bee”. We would like to thank the reviewers for their careful and thorough reading of this manuscript and for the thoughtful comments and constructive suggestions, which helped to improve the quality of this manuscript.

We look forward to hearing from you.

Sincerely,

Professor Hyung Wook Kwon

Department of Life Science,

Convergence Research Center for Insect Vectors

Incheon National University

119 Academy-ro, Yeonsu-gu, Incheon, 22012, Republic of Korea

+82-32-835-8090 (work);

+82-10-3379-6727 (mobile);

+82-32-835-0764 (fax).

Email: hwkwon@inu.ac.kr
