# Supplementary material for: The effect of seasonal temperatures on the physiology of the overwintered honey bee
Source: PLoS One. 2024 Dec 9;19(12):e0315062. doi: 10.1371/journal.pone.0315062 (PMC11627422; doi:10.1371/journal.pone.0315062)
Supplement: Attachment — Submitted filename: Response to Reviewers2.docx [file pone.0315062.s015.docx]

Dear Editor-in-Chief, Reviewer 1 and Reviewer 2!

Thank you for the time and effort spent reviewing our manuscript number PONE-D-24-13353R1 (**The effect of seasonal temperatures on the physiology of the overwintered honey bee**) and suggesting some important points to consider. We corrected the manuscript according to the reviewers' comments. Our replies are marked by **blue** after the symbol **R** (reply) and **question numbers** for the reviewer’s comments. Also, we copied these modifications in response for convenience when they were available.

Please, find below the reviewer comments **(black**) and our response (**blue**)

**Reviewer #1**: This version of the manuscript is greatly improved. I thank the authors for their extensive effort to revise it.

Having said that, there remain substantial issues that should be addressed.

1. There remain many wordings that are not clear, and minor technical issues. Some of these I commented on in the first draft but the same issues remain! For example, I commented on the overuse of "honey bee" when "bee" or "it" would be simpler. The new version is better, but the term "honey bee" remains used far too much.
Another example: I am confused by sample sizes in the paragraph in the Methods related to HP gland measurement. It is stated that 90 acini were measured and 10 acini/bee, which equals only 9 bees studied. But it also states that there were 30 bees/colony X 9 colonies = 270 bees! Please have an independent person read the manuscript for clarity.
As before, I have marked many issues and have suggested changes that I think will improve the manuscript. I hope the authors will take them to heart, as they surely want their manuscript to be as strong as possible. **R1.** Thank you for comment. We corrected the typo in the M@M chapter regarding the HG measurements description. It should state that there were 90 bees from one group, with 30 bees from one colony.

2. In the first draft, I took issue with the HP glands of winter bees being "hypertrophied". I was absolutely wrong on this, and appreciate the authors defending their wording. **R2.** Thank you.

3. In the original version, the authors had used "acinus" (singular) when in fact they meant to use "acini" (plural). It seems that in making corrections, they changed a correct term, "acinus size", to the incorrect term "acini area". Here, "acinus" (singular) is correct (just as one would write "We measured bee size", not "We measured bees size"). And I prefer "acinus size" rather than "acinus area", but that is up to the authors to decide on. **R3.** Corrected

4. Statistics: There is at least one instance where I believe average values were analyzed rather than the raw data. For example, when measuring acinus size, (line 185, revised doc), if I understand the methodology, the authors averaged the areas for the 10 bees from a colony, then conducted the analysis on the average values. Doing this will eliminate much of the variation upon which the analysis of variance is based. **R4.** Thank you for your comment. Our calculations followed the protocol published by Corby-Harris and Snyder (2018) (see screenshot below). Additionally, we applied the general technique of aggregating replicates for each acinus by computing the average expression for each raw data point within individuals to reduce noise and variability (Vaux et al., 2012). The average acinus size for each individual was used in the subsequent analysis. Data from **30** (not 10) individuals were used from each group.

Vaux, D. L., Fidler, F., & Cumming, G. (2012). Replicates and repeats--what is the difference and is it significant? A brief discussion of statistics and experimental design. EMBO reports, 13(4), 291–296.

**PDF questions**

(comment in line 63) Vibrating vs contract flight muscles in bees for thermoregulation in the winter cluster. **R:** Thank you for comment. Vibrating muscles are specialized for rapid, high-frequency oscillations, enabling honey bees to generate heat for thermoregulation, even when their wings are not flapping. These muscles can contract rapidly without significant shortening, allowing for vibration instead of traditional movement. In the winter cluster, the vibrating thoracic muscles of honey bees play a crucial role in warming the cluster, helping to maintain a stable temperature necessary for survival during cold conditions (Reddy and Kavitha, 2016). In contrast, contractile muscles are primarily responsible for movement and do not contribute significantly to heat generation in this context. Therefore, vibrating muscles are essential for effective thermoregulation in honey bee clusters during winter.

Reference for response:

(comment in line 184) “averaging the values within the 30 bees…” **R1, R4,** **R:** Thank you. We need to point out that, as mentioned in M@M (HG measurements), '10 randomly selected acini per bee' were measured, and this data was averaged. Therefore, we also accounted for within-colony variation. We corrected sentence.

(comment in line 271) **R:** AUC is used to evaluate the performance of a statistical model. An AUC of up to 1.0 indicates good performance, meaning the model effectively distinguishes between the two classes. We corrected this sentence.

(comment in line 289) **R**: These graphs presented in Supplementary material, S5 File.

(comment from lines 371 and 377) **R**: Thank you for comment. The acinus size is very small, and when calculated in SI units, the standard deviation (SD) is also small.

Other comments in PDF about grammar were accepted and corrected in the manuscript.

**Reviewer #2:** The authors indeed improved the manuscript in comparison to the previous version. They incorporated many aspects as suggested. Many aspects and parts of the manuscript still need improvements and corrections.

Major comment
5. I suggest the authors again take their results and think deeply about their meaning in respect to temperature, nutrition and season. I advise to first stick to temperature, nutrition and season (the factors accounted for in the experiment) and only later in the discussion start to give a putative link to age. The authors should take a step back and consider how the results of the different physiological and molecular markers can be linked and interpreted, which picture does emerge. At the moment I find the results and especially the discussion highly confusing since the focus constantly shifts between treatment, age, nutrition, season,... their connection should become clearer. The clear link between the results found for the different factors is missing. E.g. do they hint in the same direction or not? I know that this is not easy because several factors are putatively associated with multiple effects, but what is the take home message? What are the results from this study and what kind of interpretation is possible? **R5.** Thank you for your comment. We examined the long-term effects of summer/fall temperatures (treatment) on honey bee physiology for bees that developed during that period and survived through the winter for 4.5 months. Although the bees are chronologically the same age, their comparative physiological state, as indicated by internal characteristics and reflecting senescence (aging), reveals some differences. We attribute these differences to the summer/fall temperatures, given the consistency of other factors. Nutrition and season were general factors for all honey bees and were not controlled. We agree that this design is weak in defining biological age and is more appropriately used for assessing **comparative physiological states** (instead of aging) related to the summer/fall experimental temperatures. We corrected this through manuscript.

Minor
Abstract
6. Line 35: what do you mean by "expected markers"? **R6.** Thank you for your comment. We refer to “expected markers” as potential indicators whose relationship with winter physiology and, possibly, aging has not been widely proven and still requires further clarification, unlike acinus size, vg gene expression, and JH hormone titer. Therefore, a comparatively large acinus size and high vg titer, along with a low JH titer, are physiological markers of younger bees. However, no studies have been conducted on the “expected markers” similar to our research.

7. Line 37: in your study, you simulate "climate warming". I wonder whether it wouldn't make more sense to report that increasing the temperature to 35C leads to faster physiological aging. Thus argument vice versa, since this is what you are actually interested in and what you want to show with this study, right? **R7.** Thank you for your comment. We need to clarify that we suggest that an ambient temperature of 35°C changed the physiological state in long-lived bees, particularly when honey bees are unable to effectively downregulate brood temperature in late summer or fall (due to factors such as insufficient numbers of bees). Additionally, brood temperatures that are typically maintained at normal levels may lead to the rearing of short-lived bees. So, this suggested mechanism depends on the response of the colony to the ambient temperature.

8. Line 39: add "physiological and molecular markers" instead of just markers **R8.** Corrected
9. Line 40: change to "with an upregulation" **R9.** Corrected

Introduction
10. Line 61: this should be "foraging" **R10.** Sorry, I'm confused about where this needs to be added. I’ve attached a screenshot of where I searched. We refer to bees as foragers if they collect water, nectar, or pollen from outside and store it in the combs. However, this activity is not possible in winter, so we cannot refer to bees in diapause as foragers.

11. Line 60-61: are these studies specifically referring to climate warming? Expect for rodents these are all ectotherms and in all ectotherms lifespan depends on temperature... **R11.** Thank you for your comment. In this part of the introduction, we mention 'climate warming or **cooling**' (please see the screenshot below). Additionally, **endotherms** like rodents and pigs and **ectotherms** like *Drosophila* and honey bees serve as **models** for studying animal responses and regulation in reaction to stress triggers. In the manuscript, we discuss the **impact of temperature on mechanisms of longevity** and highlight relevant research in this area. We propose that decreased brood temperature in late summer and fall can trigger extended winter longevity in honey bees. Additionally, our research indicates that climate warming has disrupted the ability of honey bees in colonies from Group 35 to lower brood temperature, leading to changes in physiology. These changes may result in shortened longevity and subsequent winter honey bee loss in some environments.

Conti et al. (2006) reported that a modest, sustained **reduction of core body** temperature prolonged the life span of Hcrt-UCP2 transgenic mice, independent of altered diet or caloric restriction (CR), compared to their wild-type littermates (12% increase in males; 20% increase in females). Thus, the reduction of core body temperature has been proposed to contribute to the increased life span and anti-aging effects associated with CR.

12. Line 62: how do "beekeeper management practices [12, 13], and pesticide exposure from land use" link to climate change? These are factors that affect bees irrespective of climate change? Or do you want to say that the impact of these factors might become even stronger under climate change? **R12.** Thank you for your comment. Honey bee management must be flexible and account for weather conditions (“climate change”) to protect bees from becoming weakened. Honey bee responses to pesticides depend on factors such as the type of pesticide, its concentration, the health condition of the bees, the season, the strength of the colony, and climate factors. This complexity is not the focus of our current research and requires further modeling to gain more detailed insights in the future.

13. Line 64: "mitigate the impacts of climate change and prevent honey bee winter mortality are currently lacking" this sounds like you are testing methods to prevent winter mortality? Please rephrase this sentence and don't rise wrong expectations. This may be your final goal, but it goes beyond this study. **R13.** Corrected.
14. Line 95: delete "and impacts internal processes" **R14.** Corrected.
15. Line 104: change "which" to "with" **R15.** Corrected.
16. Line 123: wouldn't "lipid content" be more appropriate? **R16.** Corrected
17. Line 152: "testing them together requires further investigation" what exactly do you mean here? Testing what together? And why further investigations? Do you mean that testing the combined effect of the genes is the aim of this study? **R17.** Thank you for comment. We corrected this sentence. Please, check the screenshot below.

18. Line 162: delete "the last" **R18.** Corrected
19. Line 168: How about writing "the aging status by means of physiological and molecular markers"?
After reading the manuscript again I would suggest to write "and to compare the effect of summer temperature on the physiological and molecular state of winter bees". **R19** Thank you, we corrected this sentence. Please, check screenshot below:

20. Line 176/177: this should be "early aging" and I find the conclusion here highly far fetched. I propose to delete this part.
You did not explicitely test whether the physiological and molecular you observe indeed result in differences in aging. I still miss the mentioning of the physiological cost of brood feeding which also may lead to a reduction in lifespan. This has not been taken into account in this study, e.g. brood area to number of bees.
To what extent would this lead to changes in the traits measured here? => all this would be necessary to take into account for a "testkit".
Let's assume I am a beekeeper and use the kit in February to measure the aging status of my bees. What measures can I take in February if I find out that my bees are in an advanced aging status? **R20.** Thank you for your comment. We modified and replaced this sentence with Introduction to Conclusion. We can address practical issues for beekeepers, based on laboratory analysis, and field practice. We found that extremely warm fall weather (climate warming) alters bee physiology, potentially may reducing winter bee longevity. This (longevity) needs to be study in the future research. If this is the case, feeding colonies with sugar/pollen patties on top of the frames starting in early February and covering them with polystyrene insulation are recommended to protect them from cooling in early spring when they may begin brood rearing, specifically for Korean beekeepers. In contrast, bees experiencing typical fall conditions in Korea will develop normally and can adjust their colony development to match the weather.

21. Line 183: change "housed" to "contained" **R21.** Corrected
22. Line 184: change "and beginning egg-laying" to "which started to lay eggs in " **R22.** Corrected
23. Line 186: number of bees missing **R23.** Corrected
24. Line 187: rephrase "populations" **R24.** Corrected
25. Line 200: add "per colony" **R25.** Corrected
26. Line 203: change "Throughout the previous summer" to "Througout summer" **R26.** Corrected

27. Line 204: change "varying room" to "the according" **R27.** Corrected

28. Line 205/206: Rephrase. E.g. "From ... onwards, all colonies were kept under natural conditions. **R28.** Corrected

29. Line 220: **A)** do you mean three measurements "per" minute? = 3x60x24= 4320 measuers/day? What do you mean by approximately one file per day? Is the file information relevant? Maybe just delete.
**B)** So it turns out that the microclimate was only monitored for 2 weeks in summer. This is super relevant information and should be stated in sections where these numbers are used.
Why was the microclimate only recorded in summer over 15 days and not throughout the experiment?
**C)** Having this information from two weeks in summer only does not allow us to draw sound conclusions about transcriptomes obtained from bees in February. Many temperature changes could have happened in the meantime... maybe the insulation of the three rooms differs,...? **R29.** Thank you for this valuable remark. We divided the response into several parts. **A)** Deleted; **B)** Our research primarily focuses on the rearing and physiology of long-lived bees, particularly during the start transition at the end of summer until the end of diapause in early winter (in February). The microclimate was recorded for 15 days in August (at the end of summer), as this period was critical for assessing the initial conditions under which the last generation of overwintering bees began their development. This timeframe was chosen because August marks a crucial period in which bees are transitioning from summer activity to preparing for winter, even though other fall triggers (such as drops in ambient temperature, reduced nectar/pollen availability, and colder nights) had not yet fully manifested. Monitoring during this specific period allowed us to capture the initial environmental conditions influencing bee longevity before these fall triggers began. We believe this selective focus on the late summer period aligns with our research goals and provides valuable insight into the early stages of the bees' physiological adaptation. However, we acknowledge that a more extended monitoring period, including the fall, could provide a more comprehensive understanding of the microclimate's impact throughout the entire rearing period. This is an important consideration for future studies. **C)** We acknowledge that data from the two weeks in summer may have limitations regarding the conclusions drawn about the transcriptomes of bees sampled in February, especially if these bees had not been specifically marked. However, since honey bees reared from the August brood were marked and tracked specifically to assess their physiology in February, and all other environmental conditions remained consistent across the groups, we believe that the primary variable influencing the observed physiological changes is temperature. The influence we observed likely follows this pathway: ambient temperature → brood temperature → honey bee physiology. Given that rooms were built within the same house, differences in insulation are not expected. Nevertheless, we recognize that other unmeasured factors might impact the results, and we agree that this is an area that would benefit from further investigation.

30. Line 247: change "bee" to plural "bees"; delete "Apis mellifera ligustica colonies" **R30.** Corrected

31. Line 256: "Total tissue RNA (brain or abdomen) was extracted from fifteen randomly selected" I don't understand this procedure. **A)** Why 15 random bees, and what do you mean by random? **B)** Could this result in 10 bees from a single colony and five bees from a second?
You took 10 bees from each of nine colonies, why didn't you take one bee per colony for each of the measures? Or one bee per colony for RNA tissue extraction of the brain and fat body and another bee for the lipid content? **R31.** Thank you for your comment. The number of sampled honey bees is detailed in Figure 2. **A)** Corrected in manuscript. The term "randomly" means that the bees were selected without bias to ensure that the sample was representative of the colony.

**B)** The necessary sample size depends on the context and goals of the research (Kadam and Bhalerao, 2010). The sample size of 30 (10*3 colony) and 90 (30*3 colony) bees for molecular and physiology analysis respectively was determined based on the need to obtain reliable data, with a 95% confidence level (as shown in the attached screenshots). For molecular analysis, specifically RNA extraction, pooling samples is a standard practice because the RNA yield from individual bee brains or abdomens is typically low and may not be sufficient for robust analysis. By pooling tissues, we ensure that the RNA samples are representative of the physiological state of the colony, minimizing variability and improving the accuracy of our results. This method also helps to reduce potential sampling errors.

Kadam, P., & Bhalerao, S. (2010). Sample size calculation. International journal of Ayurveda research, 1(1), 55–57.

32. Line 258: this is redundant to line 254, delete. **R32.** Deleted
33. Line 311: "bounded" I guess this should be "bound" **R33.** Corrected
34. Line 313++: in my opinion it is not correct to perform this test here based on the data obtained from the experiment. To conduct such a test, you would first have to perform an experiment that would result in data suitable for this test. I.e. you would have to perform an experiment in which you explicitly investigate individuals with different acini sizes and lipid content, which are otherwise kept under the same conditions. Here in this experiment, you had 3 different temperature regimes and the question is whether these different treatments affect physiological and/or molecular markers. But in my point of view, your data does not allow testing for an effect of gene expression on the acinus size of lipid content. These could all be correlations due to your experimental setup. I suggest removing the ENR from M&M plus the results. **R34.** Thank you for your insightful comment. This point is critical for assessing the suitability of Elastic Net Regression (ENR) for our dataset. As per your advice, we assessed the ENR using 'ambient temperature' as the dependent variable, included this figure, and corrected the relevant text in the Results and Discussion chapters. For your convenience, you can see these modifications in the screenshots below.

Results

Discussion

Our experimental design included measurements of acinus size and lipid content of fat body in honey bees raised under controlled temperatures during the summer/fall seasons. These marked bees were then overwintered, and by February, all groups experienced identical conditions, eliminating direct temperature effects from the experiment. Therefore, the acinus size and lipid content reflected the initial experimental temperatures, while gene expression responded to the current overwintering environment and physiological state. Because acini size and lipid content still corresponded to the experimental conditions, we included them as dependent variables in the ENR model. ENR is particularly effective in managing correlated features and addressing multicollinearity, making it a suitable tool for our analysis. Although we are cautious not to interpret these findings over, we believe they add valuable insights and should be included in the results.

Results
35. Line 353: "Although significant temperature differences were not 353 detected, a stable elevated night temperature is one of the factors of climate warming 354 that influences bees." I don't understand what the authors want to convey here? Please clarify. **R35.** Thank you for your comment. The previous sentence discusses the comparison of ambient temperatures between Group control and Group 25. Although the average day and night temperatures were consistent, natural fluctuations, where days might be warmer and nights cooler, also influence bees. In our experiment, these natural temperature variations were controlled for and eliminated as a factor.

We have revised this and other sentences in the manuscript. For your reference, we have attached a screenshot below.

36. Lines 355 and 358: change "same tests" to "ANOVA".
Plus I still have the same comments and questions as in the previous round of review concerning the <0.05 values. And this accounts for all "p < 0.05" throughout the results. It is not sufficient to state p < 0.05 if your p is smaller than alpha 0.05. You need to be more precise!! I don't believe that all p-values you obtained range between 0.01-0.05 which would be the margin represented by 0.05. You must have some values which are smaller than that which should either be indicated by the exact p-value or by 0.01 (if between 0.001-0.01) or p<0.001. **R36.** Thank you for your comment. We have accepted your notes and corrected the *p-*values to reflect their specific ranges, rather than simply indicating they are below 0.05, as was done in the previous version of the manuscript. We have attached a screenshot of the summary table from the t-test or ANOVA and Duncan post-hoc tests. The calculations are also presented in the “S4 File. Stat analysis in XLSTAT.xlsx,” under the sheet named “ANOVA Duncan norm1”, “S5 File. Raw data weather and statistics.xlsx”

37. Line 362: here you start writing bees instead of "honey bees" but up to here always "honey bees". Writing bees is fine, just use it consistent throughout the manuscript, i.e. change above. **R37.** Corrected
38. Line 358: you should reference to Fig3 here. **R38.** Sorry, I was confused about the place where I can input references. Figure 3 content includes the sensor data and colony performance.

Corrected manuscript after 1^st^ revision:

Manuscript without corrections after 1^st^ revision:

39. Line 361: I cannot find information in the M&M how you measured number of larvae and capped brood area? How was this measured, how many combs, which area? What do you mean by honey bees’ measurements? **R39.** Thank you for your comment. We briefly mention colony performance measurements in lines 158-159 (see screenshot). We used the conventional visual assessment method for honey bee colonies, where each side of the frame was inspected for the percentage of capped area, larvae area, and honey bees (with 100% representing one side of a frame). These percentages were then converted into the number of capped cells, cells with larvae, or bees. The process is well explained in the reference article (Delaplane et al., 2013).

40. Line 366: "Groups control and 25 on August 08 (Fig 3 D, E, F) showed no significant differences,", why was August 8th chosen as the reference date to make the decision on putting loggers into hives or not.
Yes the referred numbers did not differ in August, but when we look at the last September measure the control group differs in all three measures from the 25 and 35 groups, which seem to have significantly lower numbers of larvae, capped brood cells, and bees.
Figure 3: A and B remove "," and put "(°C) and (%) **R40.** Thank you for your comment. We have corrected the y-axis labels in Fig. 3 (A, B). August 8th to September 19th was chosen as the reference period for deciding whether to place loggers in the hives because this is when the honey bee colonies begin rearing the brood that will become the main generation of long-lived bees for overwintering.

41. Line 389: throughout M&M you refer to the candidate genes as genes associated with "six genes related to nutrition (vg, ilp1, ilp2, TOR1),
development (JHAMT), and stress response (HSP70)" it comes out of the blue that these allow to investigate the "biological age". Please make sure that you introduce this link latest in the M&M or stick to the information given in M&M here in the results section. **R41.** Thank you for comment. We modified this. You can look at the changes on the screen shots below.

42. Line 392: remove "mass of" **R42**. Deleted
43. Fig4 caption: change "mass" to "lipid content" **R43.** Modified
44. Fig5 and according text: as mentioned above, I think the results obtained from this experiment/study do not allow to perform this analysis and the results should be removed. Wouldn't it be possible to perform this analysis with "treatment" as dependent variable to obtain information on the effect of temperature on each of the factors and their importance in desciminating between treatments? **R44.** Please, check response in **R34**.

45. Line 434: I suggest to find a new sub-header. First please make sure that the position and content of subheaders is logical. Currently the previous header fits for all results. You could specify in subheaders that you once look at physiological and then at the molecular markers to discriminate between treatment groups.
Second, I don't think that your experiments really allow to draw sound conclusions about the biological age of bees based on those markers. Feel free discuss this possiblily in the discussion, but your experimental data "only" allows to draw conclusions on the effect different temperature regimes on physiolocial traits and gene expression of candidate genes (which can be associated with...). This by itself is interesting!
To draw sound conclusions on the biological age of bees you would need to experiementally disentangle age from temperature by using different age cohorts, which you do not have. I.e. please be careful and rather remove this statement. Lipid content for example could be related to nutrition only, not age. Maybe bees at higher temperatures have a higher metabolism and thus use up their fat reserves quicker,...??? **R45.** Thank you for your feedback. We have accepted this point and changed “age” to “physiology”, which better aligns with our design. Also, we corrected sub-headers.

46. Line 440: you did not measure fat body mass, you measured lipid content. Be specific here and in the following. **R46.** Thank you, corrected in manuscript.
47. Line 448: don't write "same test", be specific. Also in the following sections, i.e. throughout the manuscript. **R47.** Thank you, corrected in manuscript**.**
48. Line 452: "accelerated physiologicaly aging" see above comments concerning this interpretation. Change and downword. **R48.** Changed.
49. Figure 6: fat body mg should be "fat body lipid content (mg)", change other axis labels accordingly also in respect to format, and change figure caption accordingly. **R49**. Thank you for your comment. It has been corrected. Please check the screenshot below for convenience.

50. Line 455: see comment to line 434 and other comments in respect to the use of "age". **R50.** Corrected.
51. Line 458: header says age and here you write task-related.... see above comments **R51.** Corrected.

Discussion
52. Line 489: "prolonged climate warming" I am confused. In the results you say that there are no differences between control and group25, and here you say that group 25 and 30 were warming conditions!? If G25 is similar to the control, then the fact that the brood parameters are lower in both G25 and G30 may be a treatment effect of either the insulated rooms or side effect constant temperatures during larval development, or ....
It might also be better to say "It was found that longer periods of higher tempartures/higher, constant tempratures, as expected under climate warming", as climate is measured over decades and centuries. **R52.** Thank you for your comment. To compare the average monthly temperatures, we analyzed the temperature dataset for the control, 25°C, and 35°C groups, and found no significant difference using the t-test. This suggests that day/night temperature fluctuations were not sufficient to produce differences. However, as shown in Fig. 1E, the main temperature difference between the control and 25°C groups was in the daytime fluctuations, which also influence honey bee physiology. Additionally, the term 'global warming' was used in the manuscript to describe the unusual continuous day/night warm temperatures. We replaced “climate warming” with 'global warming' and removed the phrase “prolonged climate warming”.

53. Line 494: you actually mention tempearture fluctuations here yourself. Please use this information in the previous sentence and argument along those lines. **R53.** Thank you for comment. We modified this paragraph according to recommendations. Please check the screenshot below for convenience.

54. Line 495++: rephrase. E.g. "While experimental ambient temperatures were set to 10°C difference, the bees effort to achieve optimal developmental temperatures for the bees reduced this difference to about 3°C. Bees in Group 25 heated the brood to approximately 30°C, while the Group 35 bees cooled the hive to 33°C, following well demonstrated known microclimate regulation [21, 22]." **R54.** Thank you. Modified. Please check the screenshot below for convenience.

55. Line 514: "related to the development of physiological and molecular markers in winter bees, which are responsible for the processes of aging and longevity [69]" rephrase. **R55.** Thank you. Modified. Please check the screenshot below for convenience.

56. Line 530: in line with comment to lipid content in the results section, acinus size has also been linked to nutritional state in nurse bees (e.g. doi:10.3791/58261). I.e. it is not exclusively an aging marker. This should be taken into consideration and clearly be mentioned in the discussion for both lipid content and acinus size. Or even better first be discussed in a way that it actually fits the result i.e. temperature treatment affected acinus size but not lipid content and then putative interpretations. Statistical results = p-values do not need to be repeated in the discussion. **R56.** Thank you for your question. We corrected this paragraph. Please check the screenshot below for convenience.

57. Line 535: why are physiological difference indicative of diapause conditions, and why only in two out of three? Please explain. **R57.** Thank you for your question. Please check the highlighted lines in the screenshot below for convenience.

58. Line 536: what is your explanation for this? => here in the discussion you refer all differences to nutrition but not age. This is not in line with what you write in the results. Also see my comments there. **R58.** Thank you for your question. We modified the focus from “aging” to “different physiological states” (related to nutrition) and are suggesting this relationship, but we do not assert it.

59. Line 539: please rephrase this sentence, unclear what "all of this" refers to. **R59.** Thank you for your question. Rephrased.
60. Lines 541-544: These sentences are confusing. How does Vg which is transferred to the HG to be fed to larvae relate suppressed JH and conditions of winter bees? Rephrase and make clearer. **R60.** Thank you for your question. We have revised the sentences to remove any confusing expressions. Please refer to the screenshot below for convenience.

61. Line 551: vg expression also differs between nurses and foragers, does it differ in nutritioinal states? Nurses generally also have a higher fat content, right? Does it also differ in respect to the amount of brood in need of food? These could all be confounding factors, which at least need to be mentioned and discussed in addition to the aging process. **R61.** Thank you for your comment. The regulation of honey bee tasks is complex, and *vg* expression is indeed influenced by nutritional states. Nurses, with their highly developed hypopharyngeal glands and fat bodies, require *vg* to produce royal jelly. This suggests that both the nutrition of the bee itself and the need to feed larvae play a role in vg expression. Additionally, nurses generally have a higher fat content compared to foragers. This discussion was added. Please refer to the screenshot below for convenience.

62. Line 554: see above comments to ENR **R62**. Please, check R34 response.
63. Line 564: this is interesting and relevant. Here you could spend some lines to give information on other studies showing this effect and especially in respect to "being already well-known". Well known for what? Be more specific. **R63.** Thank you for your comment. This expression was about the dual-mutual interaction between vg and JH, which has been corrected in this revision. Additionally, these sentences have been rewritten. Please refer to the screenshot below for convenience.

64. Line 577-585: above you write that HSP70 can be stress-associated but it has also be shown to differ between overwintering bees from fall to spring. Here you write about putative stress and nutritional needs. Where do nutritional needs come from? This has not been mentioned before. Please write this section more clearly, and more in line with the other arguments, currently I am lost. **R64.** Thank you for comment. We extended the explanation. Please refer to the screenshot below for convenience.

65. Line 585: delete this sentence. **R65.** Deleted
66. Line 588: why, how so? **R66.**
67. Line 595: could you add some guesses why you do not find an effect if this gene has been associated to nutrition, age and season, all three factors seemingly relevant to this experiment? **R67.** Thank you for comment. We extended the explanation. Please refer to the screenshot below for convenience.

68. Line 600: all of a sudden you write about constant temperatures. I totally agree that this could be a very important factor, but this factor hardly finds any mention and discussion in the discussion. Please add. **R68.** Thank you for comment. We corrected the mention of this point trough manuscript. Please refer to the screenshot below for convenience.

69. Line 606: "In contrast, the 35°C temperature hindered colony development, as honey
606 bees in Group 35 were unable to reduce brood temperatures as effectively as those in
607 Group 25, impairing winter bee development. " this statement should be downworded. Since there are no measures for brood temperature in the control group, it is impossible to say whether the 35 group had suboptimal developmental conditions. Moreover the 25 and 35 group both have less brood and workers in comparison to the control. Thus in my opinion, the results do not support this strong statement and rather hint to suboptimal conditions in the 25 and 30C groups, e.g. because of constant temperatures? Maybe constant high temperatures require the bees to constantly ventilate the hive and thus use up many more resources than the control bees, which in turn leaves them less resources for the brood? **R69.** Thank you for your comment. We agree with this discussion. We have corrected the mention of this point in the manuscript in two places. Please refer to the screenshots below for convenience.

70. Line 610: where are the numbers for honey bee survival? I did not see a section of measuring honey bee survival in M&M nor any results. this is actually a very good point. The authors marked 40 bees per colony, how many of those were still alive in February? **R70.** Thank you for your comment. As the honey bee colonies were kept under natural conditions during winter, the ambient daytime temperatures in February were often below 14°C (Screenshot 1), and there were instances of snow. This made it impractical to disturb the winter cluster by removing frames to count the marked bees, as exposing the bees to cold could have disrupted their ability to continue overwintering. Survival was checked in early spring (March), and the data was organized in Table S1 and mentioned in the Materials and Methods (Screenshot 2) and Discussion (Screenshot 3). We initially marked 200 bees in the fall, which was omitted in the Materials and Methods section, but we have corrected this (Screenshot 4). A sample of 40 marked bees was taken from the surface of the winter cluster for research (Screenshot 4). Please refer to the screenshots below for convenience.

Screenshot 1

Screenshot 2. M&M

Screenshot 3. Discussion

Screenshot 4 M@M

71. Line 611: I guess you mean summer and not fall? At least you are writing about summer conditions throughout the manuscript... please be consistent. And you should be more specific and write "such as increased summer temperatures" **R71.** Thank you. We corrected. Please refer to the screenshot below for convenience.

72. Line 613: Why "extreme heat", before it was higher temperatures...? Be consistent.
Unclear how "extreme heat" relates to management in winter? Be more specific.
What kind of management plans do you have in mind? Be more specific. **R72.** Thank you. We corrected this paragraph.

We are pleased to resubmit for publication the manuscript “The effect of seasonal temperatures on the aging of the overwintered honey bee”. We would like to thank the reviewers for their careful and thorough reading of this manuscript and for the thoughtful comments and constructive suggestions, which helped to improve the quality of this manuscript.

We look forward to hearing from you.

Sincerely,

Professor Hyung Wook Kwon

Department of Life Science,

Convergence Research Center for Insect Vectors

Incheon National University, R&D Complex,

265 Harmony-ro, Yeonsu-gu, Incheon, 22014, Republic of Korea;

+82-32-835-8090 (work);

+82-10-3379-6727 (mobile);

+82-32-835-0764 (fax).

Email: hwkwon@inu.ac.kr
